# Monocyte-lineage tumor infiltration predicts immunoradiotherapy response in advanced pretreated soft-tissue sarcoma: phase 2 trial results

**DOI:** 10.1038/s41392-025-02173-3

**Published:** 2025-03-17

**Authors:** Antonin Levy, Daphné Morel, Matthieu Texier, Maria E. Rodriguez-Ruiz, Lisa Bouarroudj, Fanny Bouquet, Alberto Bustillos, Clément Quevrin, Céline Clémenson, Michele Mondini, Lydia Meziani, Roger Sun, Nadia Zaghdoud, Lambros Tselikas, Tarek Assi, Matthieu Faron, Charles Honoré, Carine Ngo, Benjamin Verret, Cécile Le Péchoux, Axel Le Cesne, Florent Ginhoux, Christophe Massard, Rastilav Bahleda, Eric Deutsch

**Affiliations:** 1 Department of Radiation Oncology, Gustave Roussy, Villejuif, France; 2 Gustave Roussy, Inserm U1030, Université Paris-Saclay, Villejuif, France; 3 Faculté de Médecine, Université Paris Saclay, Le Kremlin-Bicêtre, France; 4 Sarcoma unit, Gustave Roussy, Villejuif, France; 5 Biostatistics and Epidemiology Office, Gustave Roussy, Villejuif, France; 6 Oncostat 1018 Inserm, University Paris-Saclay, Villejuif, France; 7 Department of Radiation Oncology, Clínica Universidad de Navarra, Pamplona, Spain; 8 Bioinformatic platform, Gustave Roussy, Villejuif, France; 9 Product Development Medical Affairs, F Hoffmann-La Roche Ltd, Basel, Switzerland; 10 Department of Interventional Radiology, Gustave Roussy, Villejuif, France; 11 Gustave Roussy, Inserm U1015, Université Paris-Saclay, Villejuif, France; 12 Drug Development Department (DITEP) Gustave Roussy-Cancer Campus, Villejuif, France

**Keywords:** Sarcoma, Tumour immunology, Sarcoma

## Abstract

Immunoradiotherapy holds promise for improving outcomes in patients with advanced solid tumors, including in soft-tissue sarcoma (STS). However, the ideal combination of treatment modalities remains to be determined, and reliable biomarkers to predict which patients will benefit are lacking. Here, we report the results of the STS cohort of the SABR-PDL1 phase II trial that evaluated the anti-PDL1 atezolizumab combined with stereotactic body radiation therapy (SBRT) delivered concurrently with the 2nd cycle to at least one tumor site. Eligible patients received atezolizumab until progression or unmanageable toxicity, with SBRT at 45 Gy in 3 fractions). The primary endpoint was one-year progression-free survival (PFS) rate with success defined as 13 patients achieving 1-year PFS. Sixty-one heavily pretreated patients with STS (median 5 prior lines; 52% men; median age 54 years; 28% leiomyosarcoma) were enrolled across two centers (France, Spain). SBRT was delivered to 55 patients (90%), with the lung being the most commonly irradiated site (50%). After a median follow-up of 45 months, the one-year PFS rate was 8.3% [95% CI: 3.6–18.1]. Median PFS and overall survival were 2.5 and 8.6 months, respectively. Best responses included partial responses (5%) and stable disease (60%). Immune profiling revealed increased immunosuppressive tumor-associated macrophages (e.g., IL4I1, HES1) and monocyte-recruiting chemokines in non-responders. Higher monocyte/lymphocyte ratios (MonoLR) in tumor and blood correlated with progression. PD-L1 status, lymphoid infiltration, and tertiary-lymphoid structures were not predictive. Although the primary endpoint was not met, this study highlights MonoLR imbalance as a potential biomarker to identify STS patients likely to benefit from immunoradiotherapy. EudraCT No. 2015-005464-42; Clinicaltrial.gov number: NCT02992912.

## Introduction

Soft tissue sarcomas (STS) are a rare and heterogeneous group of malignancies that arise from connective tissues such as muscles, fat, nerves, and blood vessels. They account for <1% of all adult cancers but present a significant clinical challenge due to their complex biology and diverse histological subtypes. More than half of STS patients are diagnosed at advanced stages, with a high percentage presenting with metastatic or locally advanced disease. This late-stage diagnosis results in a generally poor prognosis, as effective systemic treatments for advanced STS remain limited. Conventional treatments such as chemotherapy have yielded modest results, with overall survival (OS) rates remaining low and objective response rates (ORR) varying significantly depending on the specific histological subtype.^1^ Recent developments in immunotherapy, particularly immune checkpoint blockade (ICB) using anti-PD1 and PD-L1 inhibitors, have raised hopes for better treatment outcomes in cancer therapy, including STS. However, despite some promising results in certain subtypes of STS, the overall efficacy of ICB in unselected pretreated STS populations has been underwhelming. Clinical trials have reported ORRs as low as 6%, with a median progression-free survival (PFS) of only around 3 months.^2–4^ Though durable responses to anti-PD1/PD-L1 therapies have been observed in ultra-rare STS subtypes^5,6^ and in patients selected based on specific tumor microenvironment (TME) characteristics, such as the presence of tertiary lymphoid structures (TLS), these findings underscore the complexity of effectively utilizing ICB in STS treatment.^7^

The significance of this topic is underscored by the need for improved therapies for advanced STS. The limited success of ICB in the broader STS population highlights the challenge of identifying which patients will benefit from these therapies.^2–4^ Given the rarity and diversity of STS, it is likely that the presence of specific TME features may play a critical role in determining response to treatment. Identifying these features and understanding how they influence tumor behavior is crucial for optimizing treatment strategies. In addition, the role of radiation therapy in combination with immunotherapy offers an exciting avenue for improving clinical outcomes. Preclinical studies have shown that it can stimulate immune responses by inducing tumor cell death, enhancing antigen presentation by promoting the release of tumor antigens, and activating immune pathways such as the cGAS-STING pathway.^8–10^ However, radiation therapy also carries potential immunosuppressive effects, such as upregulation of PD-L1 on tumor cells, recruitment of immunosuppressive cells, and radiation-induced lymphopenia (RIL), which could counteract the benefits of immunotherapy.^8–11^ Therefore, there is an urgent need to explore strategies that combine radiation with immunotherapy in a way that minimizes these immunosuppressive effects while maximizing the immune-activating potential of both modalities.

A promising approach to addressing this challenge is the combination of stereotactic body radiotherapy (SBRT) with immune checkpoint inhibitors. SBRT allows for highly precise delivery of radiation to tumor lesions, minimizing damage to surrounding healthy tissue and potentially reducing the immunosuppressive effects associated with conventional radiation therapy. Furthermore, SBRT’s ability to target specific tumor sites may improve the efficacy of immune checkpoint inhibitors as described in preliminary clinical studies.^12,13^ However, despite these theoretical benefits, the optimal way to combine SBRT with immunotherapy, including factors such as timing, dosage, and patient selection, remains unclear.^14^ There is a pressing need for clinical studies to investigate this combination therapy in greater detail, particularly in challenging cancers like STS. The goal is to identify the most effective strategies for combining these treatments to enhance the immune response while mitigating potential adverse effects.

In response to these gaps in knowledge, the current study presents the results of a non-randomized phase 2 trial assessing the tolerance, efficacy, and immune correlates of SBRT and anti-PD-L1 atezolizumab association for patients with metastatic pretreated cancers,^15^ including STS. This trial specifically aimed to determine whether this combination could improve clinical outcomes in this difficult-to-treat patient population. In addition, we used multi-feature translational data to characterize the disease profiles of patients who better benefited from the strategy. By focusing on the role of the TME and the interaction between radiation therapy and immunotherapy, this study aimed to uncover potential biomarkers that could guide patient selection and optimize treatment regimens. Preliminary findings from the STS cohort indicate that this combination therapy may offer promising therapeutic benefits, although further research is necessary to fully understand its mechanisms of action. The present paper reports the results of this study, with a particular focus on the STS cohort, and emphasizes the importance of continued investigation into the combination of SBRT and immunotherapy in improving treatment outcomes for patients with advanced STS. This research is critical not only for advancing treatment strategies in STS but also for providing insights that could be applied to other cancers that may benefit from similar approaches.

## Results

### Patients, treatments, safety and efficacy

**Fig. 1:**
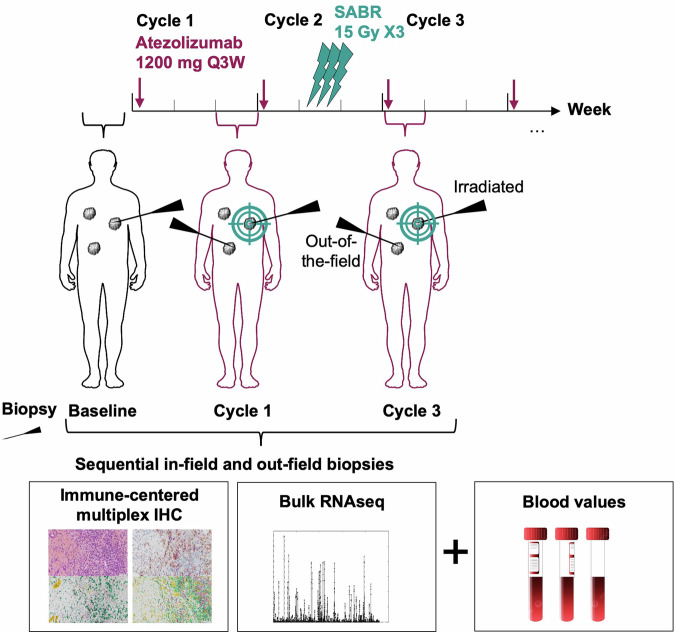
SABR-PDL1 trial design (NCT02992912)

**Fig. 2:**
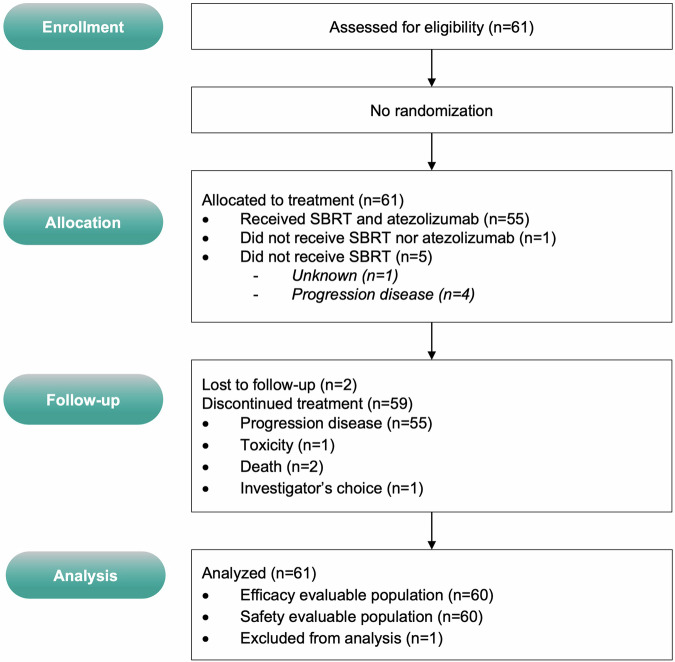
CONSORT diagram

From 03/2020 to 04/2021, 61 eligible patients with STS provided informed consent (Figs. 1 and 2, Supplementary Fig. 1) or trial participation at two European centers (France: *n* = 1, Spain: *n* = 1). Half of the patients were male (32 men [52%]), with a median age at inclusion of 54 years (range, 45–60 years). The most common histology was leiomyosarcoma (28%). Of these, 60/61 (98%) received atezolizumab treatment, and 55/61 (90%) underwent SBRT. Table 1 provides an overview of the baseline of the baseline participants’ characteristics. Given that patients underwent several chemotherapy lines (median number of prior systemic treatments: 5, range 0–9) and surgery, resulting in subgroup totals exceed the overall count for individual treatment types. In the 60/61 (98%) treated patients, the median number of atezolizumab cycles administered was 10 (range, 0–50). SBRT was delivered to 72 lesions (in 55 patients) at a median dose of 41 Gy (range 30–45 Gy; with a median dose of 42 Gy covering 95% of the planned tumor volume [PTV], range 33–44 Gy), administered in 3 fractions (55/55, 100%) over a median duration of 3 days (range 2–7 days). The median PTV volume was 40 cc (range, 25–54 cc). The main sites of irradiation were the lung (*n* = 36/72; 50%), bone (*n* = 9/72; 13%), and other locations (*n* = 27/72; 37%).

**Table 1:** Baseline clinical characteristics

Variable		Total	
		*N*	%
Age at inclusion (years)	Median (range)	54 (45–60)	
Gender	Male	32	52
	Female	29	48
ECOG PS	0	22	36
	1	39	64
Sarcomas histology	Leiomyosarcoma	17	28
	Liposarcoma	7	12
	Solitary fibrous tumor	7	12
	Others subtypes	30	49
Prior treatments^a^	Surgery	60	98
	Radiotherapy	44	72
	Chemotherapy/TT	60	98
	Median *N* lines (range)	5 (0–9)	
Tumor TLS	Yes	0	0
	No	35	57
	NA	26	43
Atezolizumab^b^	Median *N* cycles (range)	10 (0–50)	
SBRT (Gy)	Median dose (range)	41 (30–45)	
Irradiated sites (*n* = 72)	Lung	36	50
	Bone	9	13
	Others	27	37

*TT* targeted therapies, *TLS* tertiary lymphoid structures, *NA* not available

^a^Patients had several treatments so the sum of percentages is above 100

^b^One did not receive atezolizumab

The combination of atezolizumab and SBRT was well tolerated. There was no unexpected infield side-effects or immune-related adverse events (AEs; Supplementary Table 1). No grade 4 or 5 AEs attributable to the treatment were observed. One patient experienced a grade 3 atezolizumab-related AE (elevated CPK, *n* = 1; 2%), and no SBRT-related grade >3 toxicities were observed (Supplementary Table 1).

**Fig. 3:**
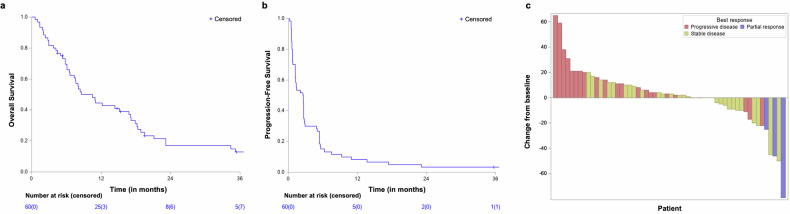
Clinical outcomes: **a** Overall survival. **b** Progression-free survival, and **c** Waterfall plot showing the best response and best change from baseline in size of target lesions in evaluable patients

The database was locked on June 6, 2024, and 60 patients were included in the efficacy analysis. After a median follow-up of 45.1 months (95% CI: 35.4–46.6 months), 5 (8%) patients were alive, and one had no evidence of disease. The median OS was 8.6 months (95% CI: 6.6–16.8 months, Fig. 3a). The one-year PFS rate and median PFS were 8.3% (95% CI: 3.6–18.1) and 2.5 months (95% CI: 1.2–2.6), respectively (Fig. 3b). Five “elite” responders (i.e., those on treatment for ≥1 year) had stable disease or partial response (SD or PR) for over one year after starting treatment, with a median time to progression of 23.1 months (95% CI: 13.7–NR). One of these patients is still receiving atezolizumab at the most recent follow-up. The trial did not meet its primary endpoint, as only 5/13 patients achieved 1-year PFS. Among the five elite patients, there were 2 out of 17 (11.8%) leiomyosarcoma, 1 out of 7 (14.3%) liposarcoma, 1 out of 1 epithelioid sarcoma, and 1 out of 4 (25%) had a malignant peripheral nerve sheath tumor (MPNST) (Supplementary Fig. 2). No significant impact of STS subtypes was observed on PFS (Supplementary Fig. 2). At 7 weeks, there was no observable objective response, however, stable disease (SD) was observed in 31 out of 60 patients (52%). Best overall responses included 3 objective responses (partial response [PR], 5%), and 36 (60%) patients had SD (Fig. 3c).

### Increased infiltration of CD68+/CD163+ tumor-associated macrophages (TAM) after SBRT is associated with progression

Patients were categorized into two groups based on their clinical response to treatment: ‘Progressive’ (*n* = 27) when tumor progression was confirmed within the 12 first weeks of treatment according to RECIST v1.1 and ‘Stable+Elite’ when the disease remained stable or responded to treatment for at least 12 weeks (*n* = 7 ‘Stable’; *n* = 3 ‘Elite”). Thirty-seven patients underwent at least one tumor biopsy for research purposes, with a median of two biopsies successfully collected and analyzed per patient (range 1–5). Among them, 36 (97.3%) had available matched blood values, and 35 (94.6%) had at least the baseline biopsy (Supplementary Fig. 1).

A total of 83 formalin-fixed paraffin-embedded (FFPE) biopsies (*n* = 64 [77.1%] ‘Progressive’ and *n* = 19 [22.9%] ‘Stable+Elite’; *n* = 33 baseline, *n* = 27 at Cycle 1 and *n* = 23 at Cycle 3) were analyzed by automatic quantification of immune cellular densities assessed by a series of multiplexed immunohistochemistry (IHC). The median surface of interpretable tumor tissue within FFPE biopsies, as contoured by an expert pathologist, was 1.89 mm^2^ (range: 0.056–23.6 mm^2^). Baseline PDL1 expression was similar between groups (mean PDL1+ cell density: 19.8 cells/mm^2^, 95%CI [0–46.1] in ‘Progressive’; 5.0 cells/mm^2^, 95%CI [0–14.7] in ‘Stable+Elite’; *P* = ns, Supplementary Fig. 3). Neither atezolizumab onset nor SBRT affected PDL1 expression assessed by IHC in this cohort. Lymphoid populations densities (CD3+, CD8+, CD20+, FOXP3+, and IRF1+ cells) were also not different between groups on FFPE biopsies (supplementary Fig. 4). All 83 pairs (*n* = 35 patients) of hematoxylin-eosin-saffron (HES) and CD3/CD20-co-labeled slides were reviewed by an expert pathologist and none of them contained TLS, including among responders (Supplementary Fig. 5).

**Fig. 4:**
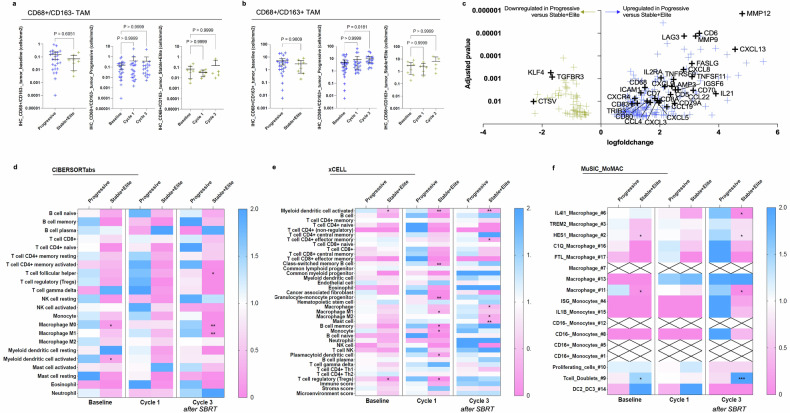
Tumor infiltration with immunosuppressive cells of the monocytic lineage after SBRT associates with progression. **a** and **b** CD68/CD163 co-labeling assessed by multiplexed immunohistochemistry on tumor FFPE biopsies. CD68+/CD163− (**a**) and immunosuppressive CD68+/CD163+ (**b**) cell densities were automatically quantified by the HALO® software on digitalized slides. Are displayed: cell density at baseline according to treatment response (left panel), cell density according to biopsy timepoint among patients with progressive disease (middle panel), and among patients with stable or responsive disease (right panel). Blue and yellow crosses refer, respectively, to patients with Progressive and Stable+Elite disease. Mean ± 95%CI. Kruskal–Wallis with Dunn’s multiple comparisons test. **c** Differential gene expression analysis of RNA-seq data comparing transcription profiles of tumor biopsies from patients who progressed with patients who showed stable disease or tumor response among 1775 selected genes. Genes differentially expressed between groups with adjusted *P*-value < 0.05 are indicated by yellow or blue crosses, respectively when downregulated or upregulated in progressive patients. Relevant genes implicated in immune regulation are depicted. **d–f** Heatmap depicting scores per immune cell type (y-axis) according to subgroups classified according to treatment response and biopsy timepoint (*x*-axis) using two independent published deconvolution tools, CIBERSORTabs (**f**) and xCELL (**e**). Scores are normalized per cell type based on the calculation: mean of the subgroup/total mean. Blue indicates higher values than the mean of samples, while pink indicates lower values. Stars indicate significant *P*-values obtained from the comparison of ’Progressive’ versus ‘Stable+Elite’ among samples obtained at the indicated timepoint, Kolmogorov–Smirnov test. **f** Idem, deconvolution of bulk RNA-seq data using the MoMac-VERSE obtained from single-cell RNA-seq description of 17 mononuclear cell types^16^. Scores are normalized per cell type based on the calculation: mean of the subgroup/total mean. Blue indicates higher values than the mean of samples, while pink indicates lower values. Stars indicate significant *P*-values obtained from the comparison of ’Progressive’ versus ‘Stable+Elite’ among samples obtained at the indicated timepoint, Kolmogorov–Smirnov test. Crosses indicate missing values. *P-value ≤ 0.05; **P-value ≤ 0.01; ***P-value ≤ 0.001

TAM infiltration was also estimated by automatic detection of CD68/CD163 co-labeling on FFPE biopsies, using the HALO® software on digitalized slides. This revealed an increased baseline infiltration trend with CD68+/CD163+ and CD68+/CD163− TAMs in tumors of patients with the progressive disease compared with tumors of patients with the stable or responsive disease, although it did not reach statistical significance (mean CD68+/CD163− cell density: 0.15 cells/mm^2^, 95%CI [0.06–0.24] in ‘Progressive’; 0.07 cells/mm^2^, 95%CI [0.001–0.13] in ‘Stable+Elite’, *P* = ns; mean CD68+/CD163+ cell density: 5.1 cells/mm^2^, 95%CI [2.4–7.7] in ‘Progressive’; 2.9 cells/mm^2^, 95%CI [0.54–5.3] in ‘Stable+Elite’, *P* = ns; Fig. 4a). Treatment did not affect CD68+/CD163− TAM infiltration, but a higher density of CD68+/CD163+ TAMs was observed after SBRT (at Cycle 3) in patients with progressive disease (mean CD68+/CD163+ cell density: 5.1 cells/mm², 95% CI [2.4–7.7] at baseline; 9.9 cells/mm², 95% CI [5.0–14.8] at Cycle 3; *P* = 0.018; Fig. 4b).

### Higher tumor infiltration of suppressive cells of monocytic lineage by RNA-seq analysis in non-responders

A total of 85 RNA-seq data points (*N* = 62 [72.9%] ‘Progressive’ and *N* = 23 [27.1%] ‘Stable+Elite’) of good quality were successfully obtained and included in the analysis. Firstly, we focused our attention on genes that are known to play a role in immunity and response to treatments (immunotherapy or SBRT). Among a dataset of 1775 genes of interest (supplementary Table 2), 269 (15.2%) were significantly dysregulated in progressive patients compared with biopsies of patients who had a clinical benefit (supplementary Table 3). CCL4 chemokine (also known as macrophage inflammatory protein, MIP-1β), implicated in the recruitment of peripheral monocytes to the tumor site, was upregulated in progressive patients as compared with ‘Stable+Elite’ patients (log(fold change [FC]) = +1.67; adjusted *P* = 0.011, Fig. 4c). In addition, transcripts of chemokines known to be secreted by macrophages were also significantly overrepresented in biopsies of unresponsive patients, including CXCL8 (log(FC) = +2.80; adjusted *P* = 0.0004), CCL22 (log(FC) = +2.53; adjusted *P* = 0.003), CXCL9 (log(fFC) = +2.17; adjusted *P* = 0.002), CXCL2(log(FC) = +1.38; adjusted *P* = 0.040) and CXCR4 (log(FC) = +1.15; adjusted *P* = 0.007). Gene set enrichment analysis suggested that pathways implicated in DNA repair, response to UV and response to IFN-γ, among others, were downregulated in biopsies of progressive patients (HALLMARK_DNA_REPAIR, NES = −2.97, adjusted *P* < 0.0001; HALLMARK_UV_RESPONSE_DN, NES = −2.28, adjusted *P* = 0.0013; HALLMARK_UV_RESPONSE_UP, NES = −1.81, adjusted *P* = 0.0085; HALLMARK_INTERFERON_GAMMA_RESPONSE, NES = 2.03, adjusted *P* = 0.0017; Supplementary Table 4, Supplementary Fig. 6).

We then intended to estimate the tumor infiltration with different immune subpopulations from bulk RNA-seq data using independent and previously published deconvolution algorithms. CIBERSORTabs, xCELL and EPIC were used to compare the abundance of specific cell populations across samples (Fig. 4d, e, Supplementary Figs. 7 and 8). When considering ‘Progressive’ versus ‘Stable+Elite’ samples regardless of the timepoint, all three tools indicated denser tumor infiltration with macrophages in unresponsive tumors (respectively, in ‘Progressive’ and ‘Stable+Elite’, median score for ‘macrophage’ by xCELL: 0.033 vs. 0.002, *P* = 0.0028; the median score for ‘macrophage’ by EPIC: 0.010 vs. 0.004, *P* = 0.0001) (Supplementary Fig. 7). Interestingly, CIBERSORTabs and xCELL scored higher for both ‘M1 macrophage’ and ‘M2 macrophage’ in biopsies from unresponsive patients compared to biopsies from patients of the ‘Stable+Elite’ group (EPIC algorithm provides an overall estimate of macrophage presence but lacks detailed subtyping; Supplementary Fig. 7). Scores estimating tumor infiltration with neutrophils were similar according to response groups with both xCELL and CIBERSORTabs (neutrophils are not provided by EPIC; Supplementary Fig. 7).

The CIBERSORTabs analysis of subgroups of biopsies considering both response and biopsy timepoint revealed a significantly lower infiltration with ‘M0 macrophage’ (*P* = 0.002) and ‘M1 macrophage’ (*P* = 0.003) after SBRT in patients who benefited from immunoradiotherapy (Fig. 4d). In contrast, the xCELL matrix rather indicated lower infiltration in responsive patients of ‘granulocyte–monocyte progenitor’ (*P* = 0.008) and ‘M1 macrophage’ (*P* = 0.020) after cycle 1 and of ‘M2 macrophage’ (*P* = 0.023) and ‘mast cells’ (*P* = 0.009) after SBRT, as compared with unresponsive patients at the same timepoint (Fig. 4e).

To deepen our understanding of treatment response according to TAM polarization, we took advantage of the MoMac signature,^16^ who computed single-cell RNA-seq analyses of 178,651 mononuclear cells to generate a monocyte–macrophage (MoMac) compendium that can be used to deconvolute signals from bulk RNA-seq data. We used the MuSiC tool^17^ to design our matrix capable of estimating specific cell type proportions while using the MoMac-VERSE as reference (Fig. 4f). We found that after SBRT and as compared to ‘Stable+Elite’, unresponsive patients showed higher tumor infiltration by ‘IL4I1 macrophages’ (*P* = 0.009), which are IFN responsive but lack IL12B (essential for antitumor activity), and are known to inhibit T cell proliferation.^16^ In addition, ‘HES1 macrophages’ (anti-inflammatory resident-tissue macrophages, mostly express M2-like genes and possibly implicated in epithelial–mesenchymal transition [EMT]) and ‘cluster 11 macrophage’ were higher at baseline and after SBRT in unresponsive patients.^16,18^ Conversely, the cluster referring to CD3E+CD7+CD3D+ ‘T cell doublets’ was more represented after SBRT in patients who responded to treatment (*P* = 0.006).

Altogether, this suggests that tumors of unresponsive patients had increased infiltration with immunosuppressive myeloid-derived cells (including specific TAMs (IL4I1, HES1) subtypes and mast cells), possibly exacerbated after SBRT.

### Blood monocyte/lymphocyte ratio mirrors tumor immune features

**Fig. 5:**
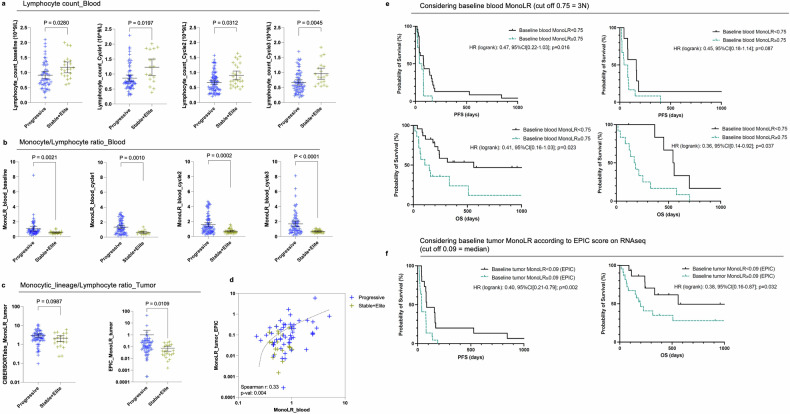
Higher tumor and blood monocyte/lymphocyte ratio in non-responders. **a** Peripheral lymphocyte counts from left to right at baseline, cycle 1, cycle 2, and cycle 3, according to the response group (blue: ‘Progressive’ group; yellow: ‘Stable+Elite’ group). Mean ± 95%CI, Kolmogorov–Smirnov test. **b** Monocyte/lymphocyte ratio (MonoLR) computed on blood values (monocyte absolute count/lymphocyte absolute count) from left to right at baseline, cycle 1, cycle 2, and cycle 3, according to the response group. Mean ± 95%CI, Kolmogorov–Smirnov test. **c** Tumor monocytic-lineage/lymphocyte ratio (MonoLR_tumor) obtained from RNAseq data with CIBERSORTabs and EPIC, according to the response group. Mean ± 95%CI, Kolmogorov–Smirnov test. **d** Correlation plot between blood MonoLR (*x*-axis) and tumor MonoLR values by EPIC (*y*-axis). Blue crosses refer to progressive patients while yellow crosses refer to patients with stable or responsive disease. The dotted line fits with the simple linear regression of equation *Y* = 0.3401*X−0.07988. **e** and **f** Kaplan–Meier curves of progression-free survival (PFS, **e**) and overall survival (**f**) from left to right: using baseline blood MonoLR in the training cohort, using baseline blood MonoLR in the validation cohort, and using baseline tumor MonoLR as estimated by the EPIC algorithm. Black lines refer to patients with baseline MonoLR < indicated threshold whereas green lines refer to patients with baseline MonoLR ≥ threshold. Logrank hazard ratio and *P*-values are depicted

Lymphopenia is associated with poorer outcomes and may be aggravated after radiation therapy.^11^ The group of Ghosh reported that a radio-induced aberrant production of myeloid-derived suppressive cells (MDSC) in the bone marrow might be the source of systemic lymphopenia.^19^ In our series, lower peripheral absolute lymphocyte counts were observed in patients who progressed as compared to their counterparts (stable or responsive disease), at baseline and after treatment onset (median baseline lymphocyte count: 0.78 vs. 1.10 × 10^9^/L, respectively in ‘Progressive’ and ‘Stable+Elite’; *P* = 0.028; median lymphocyte count at cycle 3: 0.56 vs. 0.85 × 10^9^/L, respectively in ‘Progressive’ and ‘Stable+Elite’; *P* = 0.0045; Fig. 5a). At baseline, peripheral monocyte and neutrophil counts were not different between response groups despite slightly higher values in ‘Progressive’ patients (median baseline monocyte count: 0.67 vs. 0.58 × 10^9^/L, respectively in ‘Progressive’ and ‘Stable+Elite’; *P* = ns; median baseline neutrophil count: 5.7 vs. 3.5 × 10^9^/L, respectively in ‘Progressive’ and ‘Stable+Elite’; *P* = ns; Supplementary Fig. 9).

As RNA-seq analysis indicated increased tumor infiltration with macrophages and possibly other cells derived from the monocyte compartment in our cohort, we sought to calculate the blood monocyte/lymphocyte ratio (MonoLR) both in the blood and a surrogate ratio of monocyte-derived cells (MDC) divided by lymphocytes in the tumor (MDCLR, considering monocytes + macrophages whatever their activation status + myeloid dendritic cells when available for MDC cells and the sum of lymphocytes subpopulations). We observed a significantly higher MonoLR in the blood of ‘Progressive’ patients compared to patients of the ‘Stable+Elite’ group throughout treatment, from baseline to cycle 3 (median baseline blood monocyte/lymphocyte ratio: 0.73 vs. 0.51 respectively in ‘Progressive’ and ‘Stable+Elite’; *P* = 0.0021; median blood MonoLR at cycle 3: 1.3 vs. 0.59, respectively, in ‘Progressive’ and ‘Stable+Elite’; *P* < 0.0001) (Fig. 5b). We found that post-SBRT MonoLR was a better biomarker predictive of response to immunoradiotherapy than the absolute lymphocyte count, with an area under the ROC curve (AUC) of 0.749 compared to 0.692 and 0.708 for lymphocyte counts, respectively, at baseline and post-SBRT (Supplementary Fig. 10).

Estimations of the MDC/lymphocyte ratio based on tumor RNA-seq deconvolution data also showed higher MDCLR in the tumors of unresponsive patients compared to patients with stable or responsive disease (Fig. 5c). Interestingly, tumor MDCLR scores estimated by EPIC on RNA-seq data correlated with matched blood MonoLR values in our cohort of patients with advanced STS (Spearman *r*: 0.33; *P:* 0.004; Fig. 5d). xCELL could not be used for MDCLR score calculation as it does not allow inter-cell type comparison within one sample. There was a consistent trend (but NS) of higher tumor MDCLR in unresponsive patients also when assessed in FFPE biopsies considering CD68+ for MDC and the sum of CD3+ (T lymphocytes) and CD20+ (B lymphocytes) for lymphocytes (Supplementary Fig. 11).

We investigated whether baseline MonoLR could serve as a predictive biomarker for survival outcomes in patients treated with atezolizumab plus SBRT. Our cohort was divided into two groups: a ‘training’ set (*N* = 37) comprising patients with available blood, tumor, and clinical data, and a ‘validation’ set (*N* = 19) consisting of patients with blood and clinical data but no tumor samples. Using the training set, we determined an optimal baseline MonoLR threshold of 0.75 based on the maximum likelihood ratio from the ROC curve (Supplementary Fig. 10). This threshold is approximately three times higher than the ‘healthy’ MonoLR value (~0.25). Patients with baseline MonoLR ≥0.75 exhibited significantly reduced PFS and OS (Fig. 5e). Specifically, the hazard ratios were as follows: PFS, HR 0.47 [95% CI: 0.22–1.03], *p* = 0.016; OS, HR 0.41 [95% CI: 0.16–1.03], *p* = 0.023. In the validation set, baseline MonoLR ≥0.75 was also significantly associated with reduced OS (HR 0.36 [95% CI: 0.14–0.92], *p* = 0.037). Although a similar trend was observed for PFS in the validation set, it did not reach statistical significance (HR 0.45 [95% CI: 0.18–1.14], *p* = 0.087). Additionally, a high baseline tumor MonoLR, calculated as the ratio of macrophage score to the sum of lymphocyte scores using the EPIC tool on RNAseq data, also significantly predicted reduced PFS and OS (Fig. 5f). The hazard ratios for tumor MonoLR were: PFS, HR 0.40 [95% CI: 0.21–0.79], *p* = 0.002; OS, HR 0.38 [95% CI: 0.16–0.87], *p* = 0.032.

Regarding the neutrophil/lymphocyte ratio (NLR), which is another known marker of inflammation that associates with poor prognosis in multiple diseases, we observed that patients with unresponsive disease had significantly elevated values of blood NLR compared to patients with stable or responsive disease, from baseline to cycle 3 (median baseline NLR: 5.7 vs. 3.5, respectively in ‘Progressive’ and ‘Stable+Elite’; *P* = 0.0019; median NLR at cycle 3: 9.2 vs. 4.9, respectively in ‘Progressive’ and ‘Stable+Elite’; *P* < 0.0001) (Supplementary Fig. 12). Tumor NLR on RNA-seq data could not be assessed using EPIC as neutrophils are not amongst the algorithm outputs. Estimation of tumor NLR by CIBERSORTabs showed no differences between ‘Progressive’ and ‘Stable+Elite’ groups (Supplementary Fig. 12).

### Effects of SBRT on tumor and blood from baseline

As SBRT was delivered concomitantly with the second cycle of atezolizumab, we compared RNA-seq data obtained during cycle 1 and during cycle 3 to assess the effect of radiation on tumor expression profiles. Among the 1775 genes of interest, 3 were found to be significantly upregulated (*NCS1, IGFBP2, CRLF1*), while 9 were significantly downregulated (*PGAM2, FCRL2, S100A12, FCGR3B, S100A1, MNDA, CSF3R, ACP5, BST1*) in tumors after radiation therapy (Supplementary Fig. 13). In addition, expression profiles slightly differed according to the irradiation status of the biopsy at cycle 3. *CD79B, AGR3, MROH7, IL12RB2, ENO3, SLC6A12, TNFSF11, FCRL2, NTRK1, S100A1, SLC7A10*, and *TNNI1* were significantly upregulated in out-of-the-field lesions after radiotherapy compared to lesions that locally received SBRT (Supplementary Fig. 13). Conversely, *HOPX, FBP1, AQP3*, and *STEAP4* were less expressed in lesions that did not locally receive SBRT compared to target lesions. Interestingly, several of these genes such as *S100A12, MNDA*, and *CSF3R* are implicated in immune regulation, including monocyte differentiation.^20^ SBRT led to a significant decrease of peripheral absolute lymphocyte values in patients with progressive disease but not in patients who presented with stable or responsive disease (Supplementary Fig. 13). Regarding myeloid cells, neither peripheral monocyte nor neutrophil counts were altered by SBRT from a quantitative point of view (Supplementary Figs. 12 and 13). However, blood MonoLR was significantly elevated after SBRT compared to baseline in ‘Progressive’ patients but not in ‘Stable+Elite’ patients (median blood monocyte/lymphocyte ratio in ‘Progressive’: 0.73 at baseline vs. 1.27 at cycle 3, *P* = 0.018) (Supplementary Fig. 13). A multivariate linear regression analysis was performed to evaluate the potential impact of clinical variables on the MonoLR score (Supplementary methods 1). The blood NLR, however, was not statistically significantly increased after SBRT in either group despite a trend in patients with progressive disease (Supplementary Fig. 12).

### Leiomyosarcoma subanalysis

We did not observe any significant impact of STS subtypes on PFS (Supplementary Fig. 2). Given that leiomyosarcoma (LMS) was the most frequent subtype in this study (*n* = 17, 28%) and is typically associated with low immune infiltration,^21^ we conducted additional analyses to account for STS subtype variability in immune response using translational data. Among the available data, RNAseq results were obtained from 11 patients with LMS and 26 patients with other STS subtypes. Deconvolution methods revealed that LMS generally exhibited lower immune infiltration, particularly in baseline biopsies (Supplementary Fig. 14). Notably, the xCELL algorithm estimated significantly lower infiltration with B cells (including ‘naïve’ and ‘class-switched memory’ B cells), lymphoid progenitors, mast cells, and CD4+ T cells (‘naïve’ and ‘effector memory’) in LMS biopsies. Conversely, LMS samples showed a higher presence of Th2 CD4+ T cells at baseline (*p* = 0.0001). This trend aligns with the known profile of LMS as a subtype associated with reduced immune engagement. However, these differences were not statistically significant when assessed using another method of immune infiltration estimation (CIBERSORTabs). IHC analyses (Supplementary Fig. 15) also demonstrated no significant differences in immune markers, including CD68/CD163 co-labeling, CD3, CD8, CD20, PDL1, and FOXP3, between LMS and other subtypes. Interestingly, we observed a significantly lower blood MonoLR in LMS patients during SBRT at cycle 2 (*p* = 0.0021; Supplementary Fig. 16). This may reflect a systemic immune shift induced by the combination of atezolizumab and SBRT. However, SBRT did not significantly influence MonoLR specifically within the LMS subgroup.

## Discussion

Immunotherapy has become the standard of care in several advanced tumor types, but prolonged responses to anti-PD(L)-1) have only been observed in ultra-rare STS subtypes (such as alveolar soft part sarcoma, chordoma, etc.).^2–6^ In a phase II trial evaluating atezolizumab alone in 44 pretreated STS patients, the PFS was 1.6 months, with no patients surviving beyond 1 year and no objective responses observed.^22^ The SABR-PDL1 trial was developed to evaluate whether SBRT could potentiate out-of-field immunotherapy responses in a cohort of patients with advanced STS. Despite a limited median PFS of 2.5 months and negative primary endpoint, five heavily pretreated patients showed long-lasting responses with a median time to progression of 23.1 months (95% CI: 13.7–NR), emphasizing the need for treatment personalization.

In advanced STS, early clinical reports linked “M2-like” TAMs and a lower PD-L1 expression with ICB resistance.^4,23^ However, baseline PD-L1 status has become controversial,^3^ and some other conventional markers (like TMB, mismatch repair deficiency, and neoantigens) are considered impractical for STS.^24,25^ Recent efforts in better selecting advanced STS patient candidates for immunotherapy have focused on the lymphoid compartment, specifically TLS. While TLS, composed mainly of B-cell follicles and follicular dendritic cells, may be observed in up to 25% of samples and may play a role in immunotherapy response,^7^ a high risk of false negatives persists when assessing TLS on small biopsies, and inter-lesion heterogeneity prevents robust prediction at the whole-disease level. A multicohort phase 2 study combining pembrolizumab with low-dose cyclophosphamide in patients with advanced STS found that the 30 TLS+ patients had better outcomes than the other group (*n* = 41 TLS negative patients).^7^ Agents that promote T-cell priming and T-cell therapy are being assessed.^26,27^ In our immunoradiotherapy study, PD-L1 status and TLS status did not correlate with response.

Our RNA-seq analysis revealed that tumor infiltration by immunosuppressive TAMs and monocyte-derived cells was associated with poorer outcomes after immunoradiotherapy. Preclinical studies have shown the immunosuppressive nature of tumor-infiltrating monocyte-derived cells after ionizing radiation, with some macrophage populations negatively influencing radiotherapy response.^8,28,29^ Two retrospective reports with a limited number of patients assessed the immune TME after preoperative radiotherapy in non-metastatic STS. Keung et al. observed an increased proportion of CD4+ T cells and PD-L1 expression after preoperative irradiation.^30^ Another team reported a rise in the percentage of monocytes, M2 macrophages, B-cells, and CD4+ T cells after neoadjuvant therapy.^31^ A first non-comparative phase 2 trial report using neoadjuvant nivolumab and/or ipilimumab ± irradiation in localized STS (*n* = 27 patients given early trial closure). Relapse-free survival benefits were mainly observed in patients receiving radiotherapy. In that study, baseline T-cell infiltration (lower T-reg and higher cytotoxic T lymphocytes), but not PD-L1 expression or macrophage, correlated with pathologic response.^32^ In our study, chemokines transcripts linked to macrophages and immunosuppressive TAMs were enriched in tumors of unresponsive patients. Other chemokines, such as CXCL13 and CCL5, are also expressed by stem-immunity hubs, which involve interactions between myeloid cells (e.g., CXCL10+ macrophages, mature regulatory DCs) and T cells (CD8+, T-regs), and are linked to favorable outcomes with PD-1 blockade therapy.^33^ Detailed signal analysis suggested that unresponsive patients had higher tumor infiltration with specific TAMs. IL4I1 macrophages, which share some IFN-responsive cells but lack antitumor activity. Instead, they inhibit T-cell proliferation and activation, while promoting regulatory T-cell (Treg) activity.^16^ HES1 TAM are anti-inflammatory resident-tissue macrophages, possibly implicated in epithelial–mesenchymal transition.^18^ Peripheral radio-induced lymphopenia has been linked to poor outcomes across multiple tumor types, including STS,^11^ and blood MonoLR has been associated with worsened OS in several malignancies, including STS.^34,35^ In the colorectal cancer cohort of the same SABR-PDL1 trial, we showed that SBRT combined with atezolizumab redirected immune cells toward tumor lesions and identified biomarkers like PD-L1 and IRF1 expression as predictors of response.^15^ This subsequent analysis is the first to pair blood MonoLR with tumor MDCLR estimates from RNA-seq data in advanced STS. Here, blood MonoLR correlated with tumor MDCLR (*p* = 0.004), and both were associated with immunoradiotherapy outcomes (Fig. 5), offering multiple potential explanations. First, the off-target irradiation of blood and surrounding healthy tissues can affect monocyte trafficking^36^ and circulating lymphocytes’ survival. Additionally, radiation therapy can activate the monocyte-derived type I interferon pathway, which promotes TAM accumulation.^16^ Patients with rapid disease progression exhibited a higher systemic inflammation burden, as indicated by elevated baseline MonoLR and NLR levels. This increased inflammatory state could stimulate the secretion of myelopoiesis-activating factors (e.g., IL-4, IL-6, and CSF-1), which may potentially be inhibited by targeted therapies, such as the IL-4Rα blocking antibody dupilumab.^37^ These factors drive the differentiation of hematopoietic stem cells towards myeloid phenotypes at the expense of lymphocyte proliferation. This would indicate that in patients with a high baseline inflammation burden, radiation therapy may feed a detrimental loop in which post-treatment immune regeneration is directed towards myeloid, and possibly pro-tumorigenic lineage. This aligns with the report of Ghosh et al. that described a radio-induced aberrant production of regulatory MDSC by the bone marrow following tumor irradiation, which caused secondary lymphocyte inactivation and lymphopenia, reversible by MDSC blockade in mice.^19^ Ongoing research is focusing on immune cell-sparing next-generation radiation therapy techniques (FLASH-RT, protons, etc.),^38^ refined volumes, and pharmacological support of the lymphocytic compartment to mitigate radio-induced loss and maintain an effective post-treatment immunosurveillance.^11^

Although this phase II clinical study included a robust translational research component, it is subject to certain limitations, which may be further compounded by the numerous subtypes of STS. We did not observe any significant impact of the STS subtype, and various subtypes were represented among the five patients with ‘elite’ (Supplementary Fig. 2). In addition, we found very few differences in the transcriptomic profiles of lesions biopsied post-radiotherapy according to their location (i.e., in or out of the radiation field) with only 16 transcripts (out of >32,500) being significantly differentially transcribed between irradiated and unirradiated tumors (Supplementary Fig. 13). This allowed us to pool the post-radiotherapy samples for other analyses with a low risk of introducing bias. Despite the limitations of our current dataset (small size), we found distinct immune features (Supplementary Fig. 14) and a significantly lower blood MonoLR (Supplementary Fig. 16) after treatment onset in LMS patients (most frequent STS subtype; *n* = 17, 28%) as compared to other patients. Other teams’ data have suggested that immunoradiotherapy combination efficacy may be increased by sequential administration (vs. concurrent), lower delivered doses, and in earlier stage disease,^9,14^ which is in line with a lower systemic inflammation burden. As a single-arm, prospective phase II trial utilizing concurrent immunotherapy and high-dose SBRT, the true effects of SBRT can only be conclusively evaluated in a randomized trial setting. Such trials are currently ongoing (NCT04498767, NCT03548428). Other groups have investigated systemic treatment intensification strategies such as upfront immunotherapy^39^ or combining anti-PD(L)-1 therapy with conventional chemotherapy^40^ or tyrosine kinase inhibitors (TKIs),^41,42^ yielding modest clinical benefits.

We conducted our correlative analyses by comparing PD to SD at 12 weeks as a surrogate for our study of features linked with the potential clinical benefit of the intervention, considering patients with PD at 12 weeks as “unresponders” and the others as patients having potentially benefitted from the combination. It is well described that RECIST criteria have certain limitations in the context of STS, including when evaluating responses to immunotherapy.^6,43^ Among the five patients with long-term response, only one achieved PR (Supplementary Fig. 2). We also analyzed small biopsy samples from metastases obtained at various timepoints and from different organs, which may not fully represent the disease’s immune infiltration heterogeneity, particularly regarding TLS status assessment. We observed a strong correlation between paired blood MonoLR and tumor MDCLR, although we acknowledge that the precise composition of subpopulations and their respective degree of activation may differ greatly between TME and circulation. We hypothesize that this correlation may be linked to the high inflammation burden in non-responsive patients, which likely favors TAM polarization, supporting the secretion of myeloid stimulating factors and fueling systemic myeloid/lymphoid imbalance. The role of M2-like TAM in ICB resistance was also highlighted by others after using a machine-learning framework by using bulk transcriptomics.^44^ Other teams are exploring competing endogenous RNA network^45^ or proteomics^46^ data to identify STS-related immune features or molecular subgroups. Others cellular crosstalk, e.g. of tumor-resident mast cells and cancer-associated fibroblasts (CAF), could play a role in response.^47,48^ Early clinical trials are assessing the impact of various anticancer drugs on STS TME and response.^43,47–49^

Overall, this study shows that immunoradiotherapy with atezolizumab and metastasis-directed concomitant SBRT during cycle 2 can trigger durable disease control in patients with heavily pretreated advanced STS, even though the trial did not meet its primary endpoint. Translational data suggest that tumor infiltration with specific immunosuppressive TAMs may impair the outcome. In addition, we uncovered that baseline blood MonoLR could be a reliable non-invasive biomarker predictive of response and survival in this setting. Altogether, our study provides an increased understanding of the TME that could lead to more personalized drug-radiotherapy combinations in advanced STS patients.

## Materials and methods

### Study design and procedures

The trial design has been previously described.^15^ In brief, this international, multicenter, single-arm phase 2 trial included several patient cohorts (such as colorectal and STS) who met the following eligibility criteria: (1) at least a lesion suitable for SBRT and (2) an unirradiated lesion measurable by RECIST v1.1 (Response Evaluation Criteria in Solid Tumors, version 1.1). For the STS cohort, patients were required to have experienced treatment failure according to current standard recommendations and must not have received prior ICB.

SBRT was administered concomitantly with the second cycle (week 6, Fig. 1) of atezolizumab (1200 mg IV, every 3 weeks, for up to 2 years) at an ablative dose (equivalent biologic dose (BED) >100 Gy) of 45 Gy in three fractions of 15 Gy. The protocol allowed for dose adjustments based on normal tissue tolerance constraints. The radiation dose was prescribed to the 90% isodose line to ensure that 95% of the planned dose was delivered to 95% of the PTV.

Scans to assess all earlier involved disease locations were conducted at weeks 4, 7, 13, and each 12 weeks thereafter, or as clinically indicated. Secondary endpoints comprised safety, measured by the toxicity profile defined by the Common Terminology Criteria for Adverse Events (CTCAE v4.03), OS, and ORRs for unirradiated targets based on RECIST v1.1. Patients who experienced clinical benefit and remained on atezolizumab for over a year were classified as “elite responders.”

### Blood analysis

Whole blood cell counts (including red blood cells, neutrophils, eosinophils, lymphocytes, monocytes, basophils, and platelet counts) were retrospectively extracted from the database over the period (day −7 to day +63 after Cycle 1, Day 1) for patients who agreed to participate to translational research. Data were split into two sets: a training set that corresponds to patients from whom we had clinical + blood counts + tumor biopsies and a validation set composed of patients with clinical and baseline blood data (no tumor). Treatment periods were split as follows: baseline (day −7 to day 0), cycle 1 (day +1 to day +21), cycle 2 (day +22 to day +42), and cycle 3 (day +43 to day +63). For blood-only analyses, all available measurements were considered. For correlations with tumor-based data, when more than one whole blood cell count was available, the median value per patient and per cycle was considered for baseline, cycle 1 and cycle 3 periods. MonoLR was obtained by (monocyte count [10^9^/L]/lymphocyte count [10^9^/L]) and blood neutrophil/lymphocyte ratio (NLR) was obtained by (neutrophil count [10^9^/L]/lymphocyte count [10^9^/L]). Except for Kaplan–Meier survival analyses, only the training set was used to describe the effect of treatment on blood counts. For Kaplan–Meier curves according to baseline MonoLR, a threshold was selected based on the maximum likelihood ratio obtained from the ROC curve done on data from the training dataset. The identified threshold was then applied to the validation dataset.

### Immunohistochemistry analysis

After providing specific informed consent for translational research, patients undertook sequential tumor biopsies at baseline, week 3 (Cycle 1, before SBRT), and week 7 (Cycle 3, after SBRT), from both irradiated and unirradiated sites, for biomarker analysis (Fig. 1 and Supplementary Fig. 1). Tumor samples were collected as FFPE and freshly frozen for IHC analysis and whole RNA extraction. A senior pathologist assessed tumor cellularity in each sample using an HES-stained slide from the FFPE-preserved biopsy, excluding samples without tumor cells from the analysis. FFPE blocks were sectioned into 4-μm slices for IHC multiplexing. An immune-targeting panel was developed at the Experimental and Translational Pathology (PETRA) platform of Gustave Roussy, utilizing the following chromogenic library: 2-Plex CD163/CD68 (anti-CD163, DBS catalog no. Mob460-05, clone 10D6; anti-CD68, DAKO catalog no. M0876, clone PG-M1) and 4-Plex CD8/PD-L1/FoxP3/cytokeratin (CK). (anti-CD8, Roche catalog no. 05937248001, clone SP57; anti-PD-L1, Roche catalog no. 7994190001, clone SP263; anti-FoxP3, Spring catalog no. ab99963, clone SP97; anti-CK, DBS catalog no. Mob190.05, clone AE1–AE3), and 4-Plex IRF1/CD20/CD3/CD68 (anti-IRF1, Cell Signaling catalog no. 8478, clone D5E4; anti-CD20, DAKO catalog no. M075501-2, clone L26; anti-CD3, DAKO catalog no. A0452, polyclonal; anti-CD68, DAKO catalog no. M0876, clone PG-M1). After staining, slides were digitalized at ×20 magnification using a VS120 scanner (Olympus Life Science) into .vsi files. The cellular densities of label-positive cells were automatically assessed using the HALO® image analysis software, in-situ hybridization module. The presence of TLS was evaluated by a senior pathologist (C.N.) on both HES slides and the chromogenic 4-Plex IRF1/CD20/CD3/CD68. Tumor IHC MonoLR was estimated by calculating the CD68+ density [cells/mm^2^] divided by the sum of CD3+ density [cells/mm^2^] + CD20+ density [cells/mm^2^].

### RNA-seq analysis

Tumor whole RNA was retrospectively extracted using the AllPrep RNA Mini Kit (Qiagen) according to the manufacturer’s instructions. The RNA samples underwent human mRNA sequencing at Novogene, conducted on an Illumina NovaSeq 6000 system following rRNA removal. The obtained files in FASTQ format were then subjected to quality assurance using Fastp^50^ v0.20. Samples were considered of good quality if Q20 > 90% and Q30 > 85%. This quality control involved trimming low-quality bases from the 3’ and 5’ ends of the reads, as well as filtering out bases with an average quality below the threshold of 10. Additionally, reads containing more than 50% low-quality bases, those with more than 7 undetermined bases, and reads shorter than 15 base pairs were removed. Subsequently, we performed further quality control using FastqScreen^51^ v0.14.0 to assess the sample origin by mapping against a wide range of potential target genomes with the Bowtie2 aligner v2.2.5. For downstream analysis, the cleaned reads were aligned to the hg38 human reference genome using Salmon^52^ v1.4.0 to estimate transcript abundance and normalize counts to Transcripts per Million. To assign reads to each transcript and import the count data into R, we utilized Tximport.^53^ Differential gene expression analysis was conducted using the R package DESeq2^53^ v1.34.0 on the count matrix, with an alpha threshold of adjusted *P*-values of 0.05 and a log2 foldchange threshold of 0.6. We compared samples from Progressive patients to those from Stable and Elite patients, both before and after SABR treatment and out-of-the-field lesions after radiotherapy with lesions that locally received SABR. The results of this analysis were filtered based on a previously defined list of 1775 genes relevant to our pathology context. This list includes genes from the LM22 list as well as genes from the Hallmark gene sets obtained from the MSigDB database.^54^ Gene Set Enrichment Analysis was performed with the R package fgsea^55^ v1.20.0 on Hallmark gene sets from the MSigDB database (h.all.v2023.2.Hs.symbols.gmt).

To estimate the cellular composition of the bulk sample, we performed deconvolution using three different tools: CIBERSORTabs,^56^ EPIC,^57^ xCell,^58^ and MuSiC_MoMAC.^17^ CIBERSORTabs and EPIC are deconvolution methods based on reconstructing data from expression profiles. CIBERSORTabs uses v-support vector regression, while EPIC uses least square regression to estimate cell fractions. In contrast, xCell is a deconvolution method based on marker genes, where an enrichment score is calculated to evaluate the expression of each gene for each cell type. This approach allows for the examination of cellular composition by identifying specific molecular signatures associated with each cell type. These methods enabled us to obtain an estimate of the cellular composition of the bulk sample, providing additional insights into our transcriptomic data. A surrogate “tumor MonoLR” score was calculated based on the following: tumor_MonoLR for CIBERSORTabs = sum of scores related to monocytes, macrophages, and myeloid dendritic cells divided by sum of scores related to T, B, and NK cells, and for EPIC = macrophage score divided by sum of (B cell, T cell CD4+, T cell CD8+, and NK cell).

In addition to the aforementioned deconvolution methods, we employed the Multi-Subject Single Cell deconvolution (MuSiC)^17^ tool for a more refined characterization of monocyte and macrophage populations in our bulk RNA-seq samples. To accomplish this, we utilized single-cell data from the human MoMac-VERSE,^16^ which comprises a comprehensive monocyte–macrophage (MoMac) compendium derived from single-cell RNA-seq analyses of 178,651 mononuclear cells. We designed a matrix capable of estimating specific cell type proportions using the MoMac-VERSE as a reference. For each macrophage and monocyte cluster identified in their study, we selected the 20 most significant genes based on the *P*-values from the differential expression analyses conducted by the authors (Supplementary methods 1). This approach allowed us to deconvolute signals from our bulk RNA-seq data and estimate the composition of specific macrophage subtypes.

### Statistical analyses

The primary efficacy endpoint was the one-year PFS rate, defined as the time from the initiation of atezolizumab treatment to the first documented disease progression or death from any cause. Each cohort (colorectal, STS, etc.) was analyzed individually. A Fleming 1-stage design was employed to demonstrate that the one-year PFS rate was not inferior to 15%, but could reach as high as 32%. To test the hypothesis in each cohort that the PFS rate was >*p*0 = 15% with an alpha level of 0.033 and a 90% power to detect activity >*p*1 = 32%, a total of 54 evaluable patients was required. If 13 or more patients in the cohort were alive and progression-free at one year, the combination of SBRT + atezolizumab would be considered a success for the respective histology. To account for up to 10% of non-evaluable patients, 60 patients were enrolled per cohort. A *P*-value of ≤0.05 was considered statistically significant. Statistical analyses were conducted using SAS software, version 9.4, or R software, version 3.0.2 (R Foundation for Statistical Computing).

Translational data were analyzed and plotted using R software v4.1.1 and Prism v10.1.0 (GraphPad, California, USA). Unless otherwise stated, multigroup comparisons were done according to a Kruskal–Wallis test followed by Dunn’s multiple comparisons test. For simple comparison analyses, the Kolmogorov–Smirnov test was used for non-parametric testing. Mean with 95% confidence interval (95%CI) as well as numeric *P* values are displayed in the figures. Differences with *P* values >0.05 were considered not statistically significant (ns).

## Ethics

The trial was approved by the appropriate ethics/institutional review board (SI-CPP reference: 16.00568.160322) and followed the Declaration of Helsinki, conducted in accordance with international good clinical practice standards. All patients provided written informed consent at enrollment. This trial is registered in the Clinical Trial Registry: EudraCT No. 2015-005464-42 and Clinicaltrial.gov number: NCT02992912.

## Supplementary information

Supplementary Materials

Trial Protocol

## Data Availability

The bulk RNA-seq raw data generated during this study have been deposited in the European Genome–phenome Archive (EGA) under accession number EGAD50000000958 (available at: https://ega-archive.org/datasets/EGAD50000000958).
