# Supplementary material for: Monocyte-lineage tumor infiltration predicts immunoradiotherapy response in advanced pretreated soft-tissue sarcoma: phase 2 trial results
Source: Signal Transduct Target Ther. 2025 Mar 17;10:103. doi: 10.1038/s41392-025-02173-3 (PMC11914280; doi:10.1038/s41392-025-02173-3)
Supplement: Supplementary file 1 — Supplementary Materials [file 41392_2025_2173_MOESM1_ESM.docx]

Supplementary Materials for

**Monocyte-lineage tumors infiltration predicts immunoradiotherapy response in advanced pretreated soft-tissue sarcoma: phase 2 trial results**

Antonin Levy, Daphné Morel, Matthieu Texier, Maria E. Rodriguez-Ruiz, Lisa Bouarroudj, Fanny Bouquet, Alberto Bustillos, Clément Quevrin, Céline Clémenson, Michele Mondini, Lydia Meziani, Roger Sun, Nadia Zaghdoud, Lambros Tselikas, Tarek Assi, Matthieu Faron, Charles Honoré, Carine Ngo, Benjamin Verret, Cécile Le Péchoux, Axel Le Cesne, Florent Ginhoux, Christophe Massard, Rastilav Bahleda, Eric Deutsch

Correspondence to: Antonin Levy ([antonin.levy@gustaveroussy.fr](mailto:antonin.levy@gustaveroussy.fr)) and Eric Deutsch ([eric.deutsch@gustaveroussy.fr](mailto:eric.deutsch@gustaveroussy.fr)),

**This PDF file includes:**

Figures. S1 to S16

Tables S1 to S4

Suppl Methods


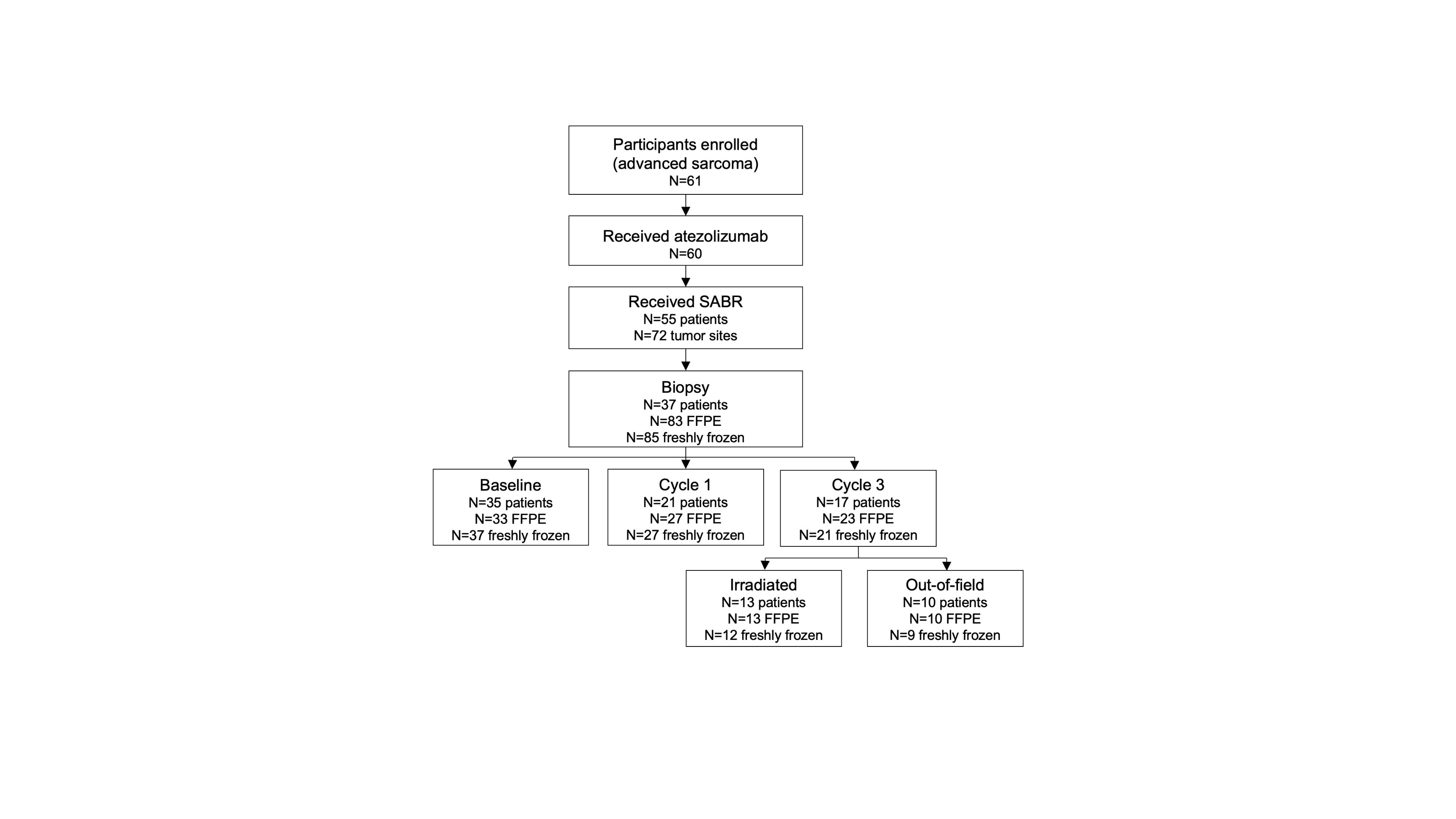


Figure. S1. Samples collection


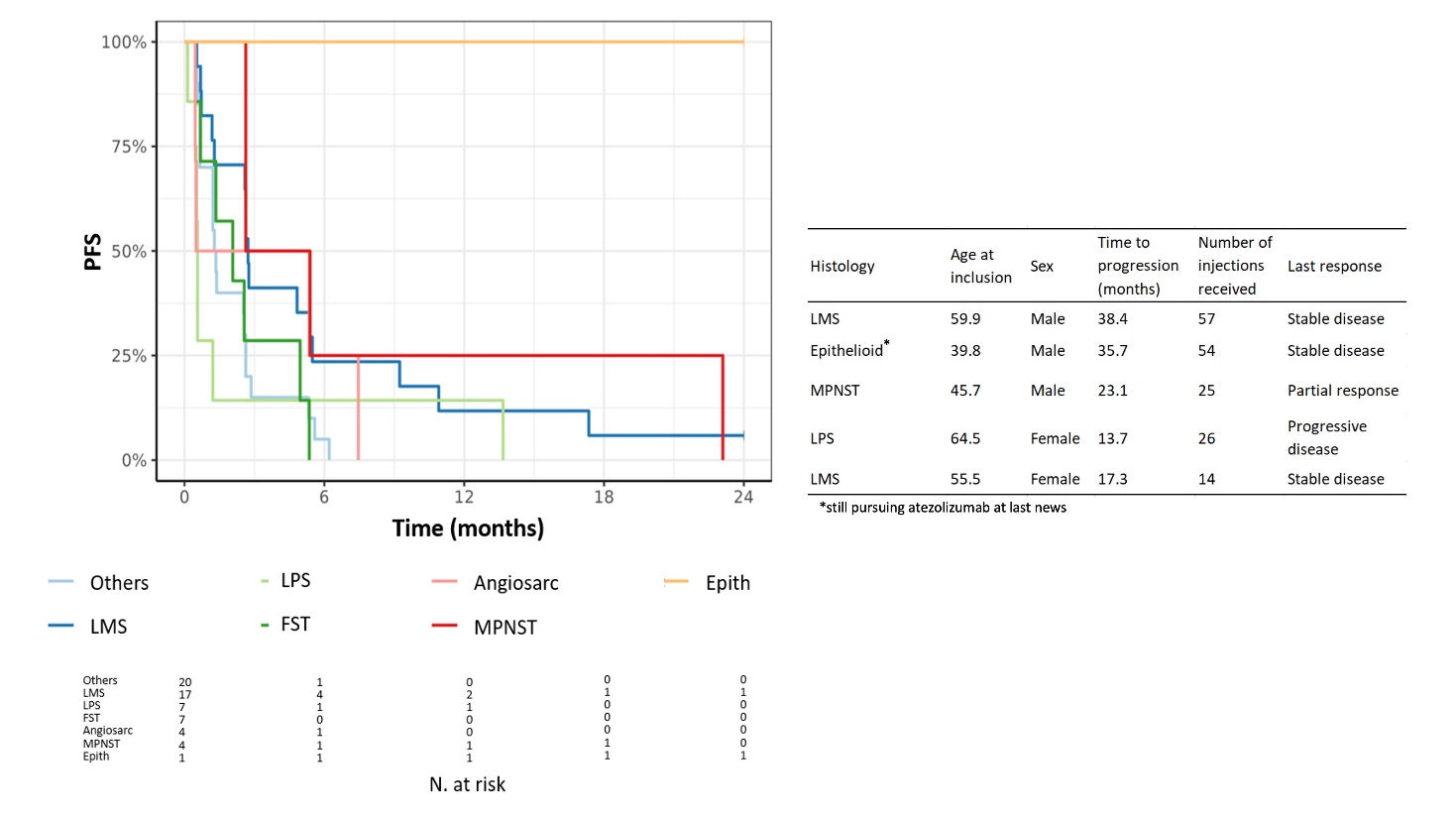


Figure. S2. *(left)* PFS according to sarcoma subtypes and *(right)* description of the five « elite » patients with PFS>1year.

LMS: Leiomyosarcoma, MPNST: Malignant Peripheral Nerve Sheath Tumor, LPS: Liposarcoma, FST: Fibrous solitary tumor, Angiosarc: Angiosarcoma, Epith: Epithelioid Sarcoma/


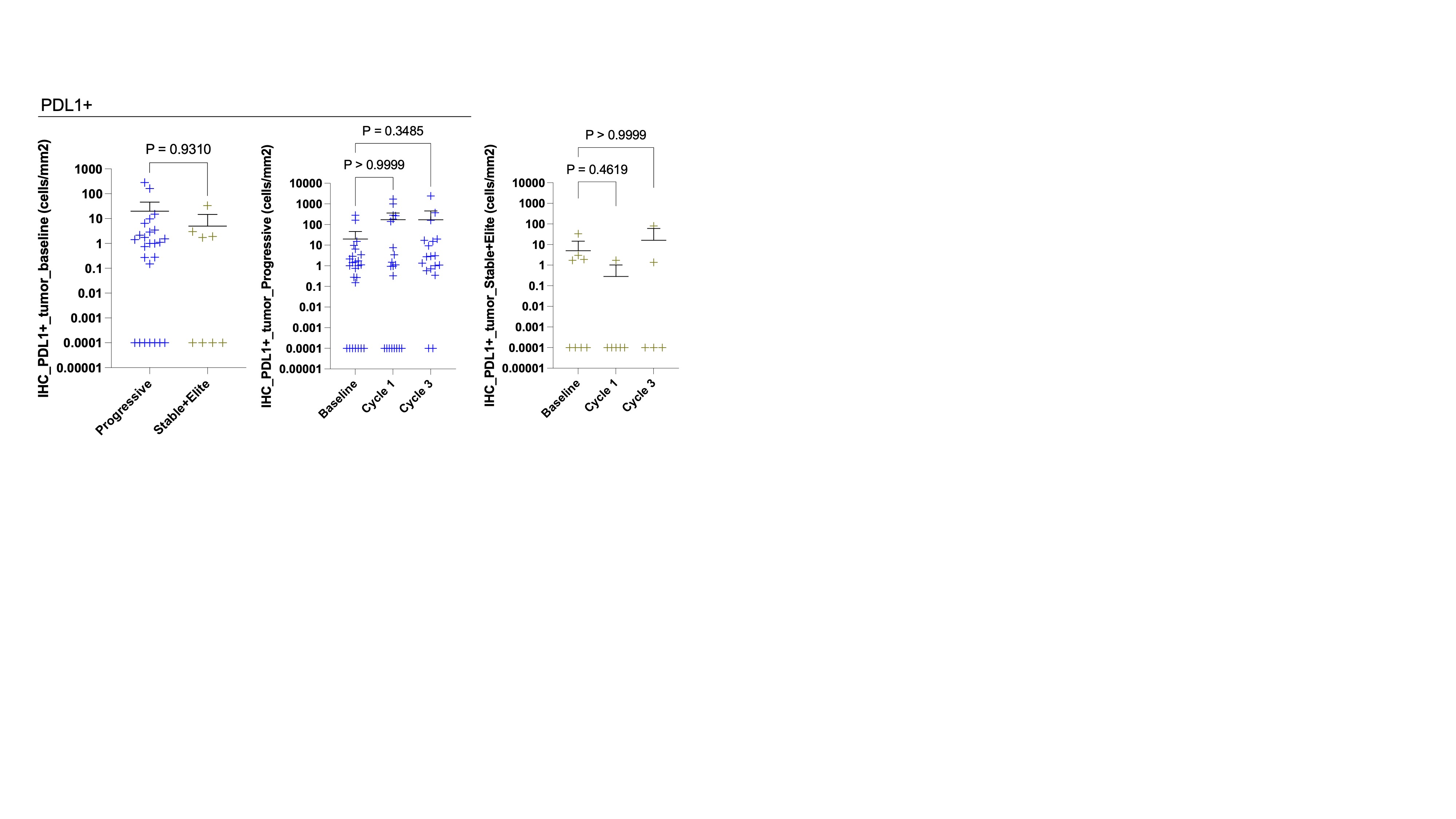


Figure. S3. Immune PDL1 expression assessed by multiplexed immunohistochemistry on tumor FFPE biopsies.

Cell densities were automatically quantified by the HALO® software on digitalized slides. Are displayed: cell density at baseline according to treatment response (left panel), cell density according to biopsy timepoint among patients with progressive disease (middle panel) and among patients with stable or responsive disease (right panel). Blue and yellow crosses refer respectively to patients with Progressive and Stable+Elite disease. Mean +/-95%CI. Kruskal-Wallis with Dunn’s multiple comparisons test.


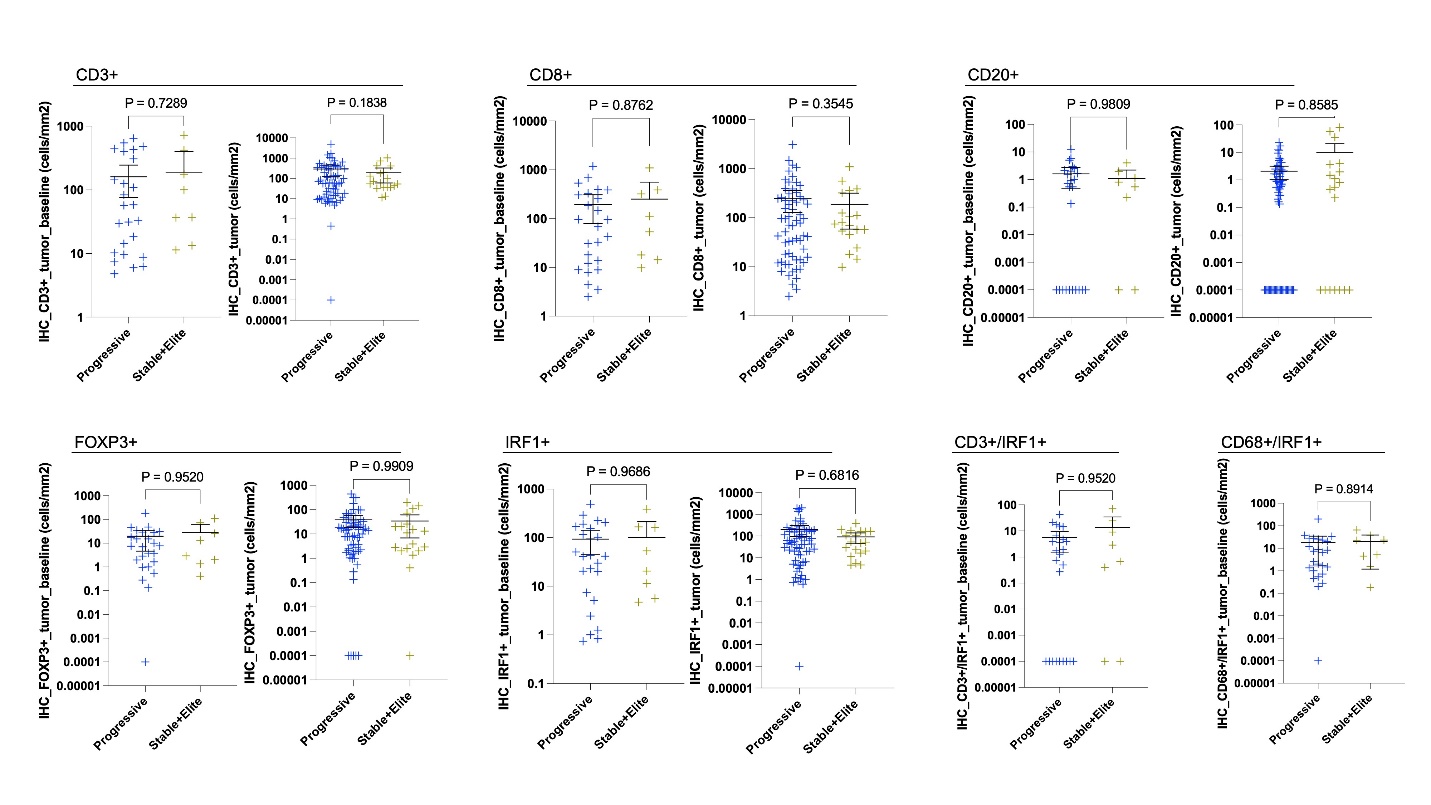


Figure. S4. Immune marker expressions assessed by multiplexed immunohistochemistry on tumor FFPE biopsies

CD3; CD8; CD20; FOXP3; IRF1; CD3/IRF1 co-labelling; CD69/IRF1 co-labelling. Cell densities were automatically quantified by the HALO® software on digitalized slides. Are displayed: cell density at baseline according to treatment response (left panel), cell density according to treatment response on all biopsies including baseline, cycle 1 and cycle 3 (right panel). Blue and yellow crosses refer respectively to biopsies of patients with Progressive and Stable+Elite disease. Mean +/-95%CI, Kolmogorov-Smirnov test.


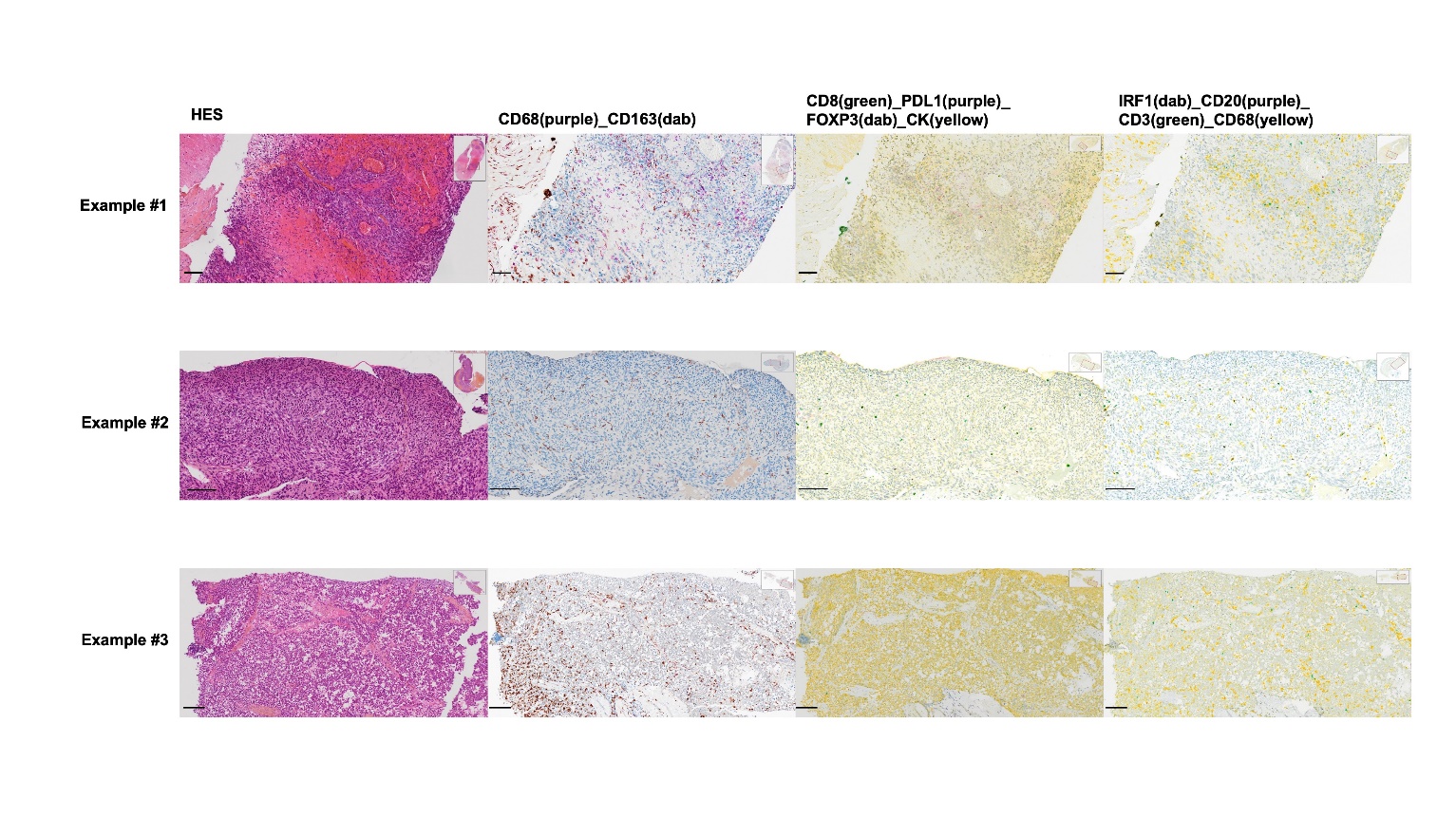


Figure. S5. Representative slides of IHC multiplexes

Digitalized at 20X. Three blocs from three distinct biopsies are shown. Black horizontal scale bares represent 100 μm.


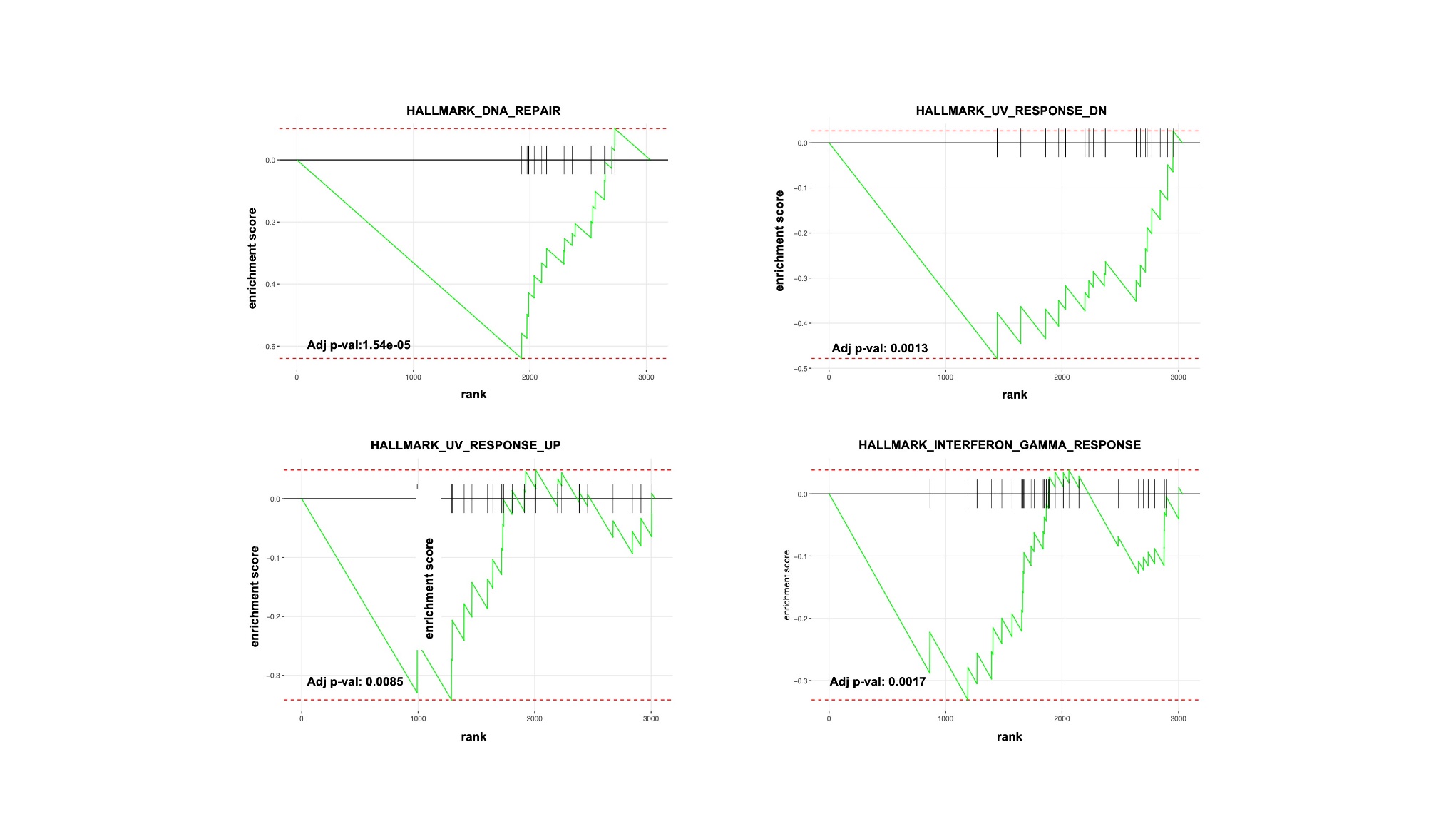


Figure. S6. Gene set enrichment analysis (GSEA) showing enrichment in pathways that were downregulated in tumor biopsies from patients who progressed compared to patients who showed stable disease or tumor response.


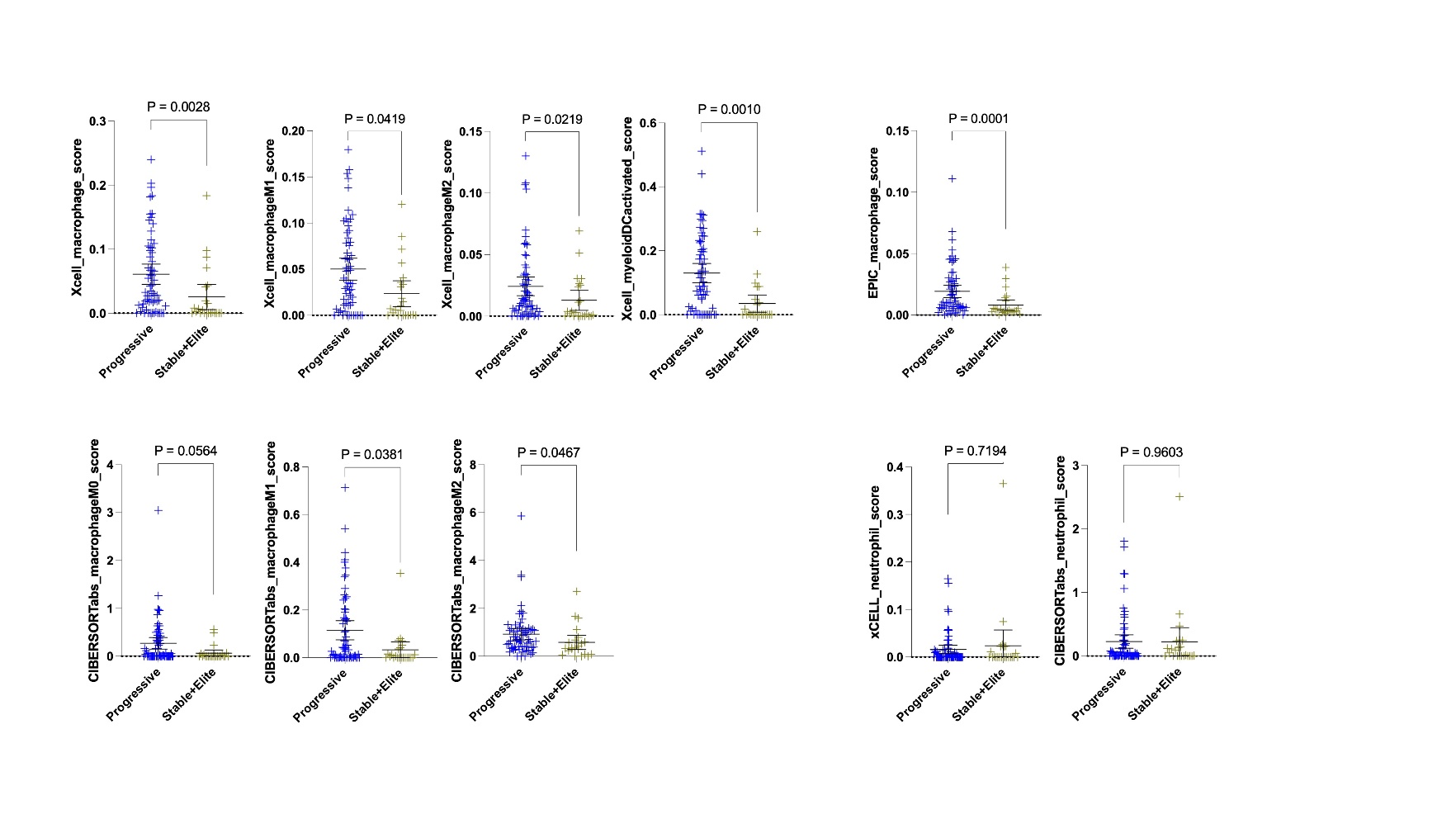


Figure. S7. Details of macrophage-related scores obtained by XCELL, an independent deconvolution tool that allows only inter-sample comparison, according to the response group.

Mean +/-95%CI, Kolmogorov-Smirnov test. Detail of the macrophage scores obtained by EPIC according to the response group. Mean +/-95%CI, Kolmogorov-Smirnov test. Detail of the macrophage-related scores obtained by CIBERSORTabs according to the response group. Mean +/-95%CI, Kolmogorov-Smirnov test. Detail of neutrophil scores obtained by xCELL and CIBERSORTabs according to the response group. Mean +/-95%CI, Kolmogorov-Smirnov test.


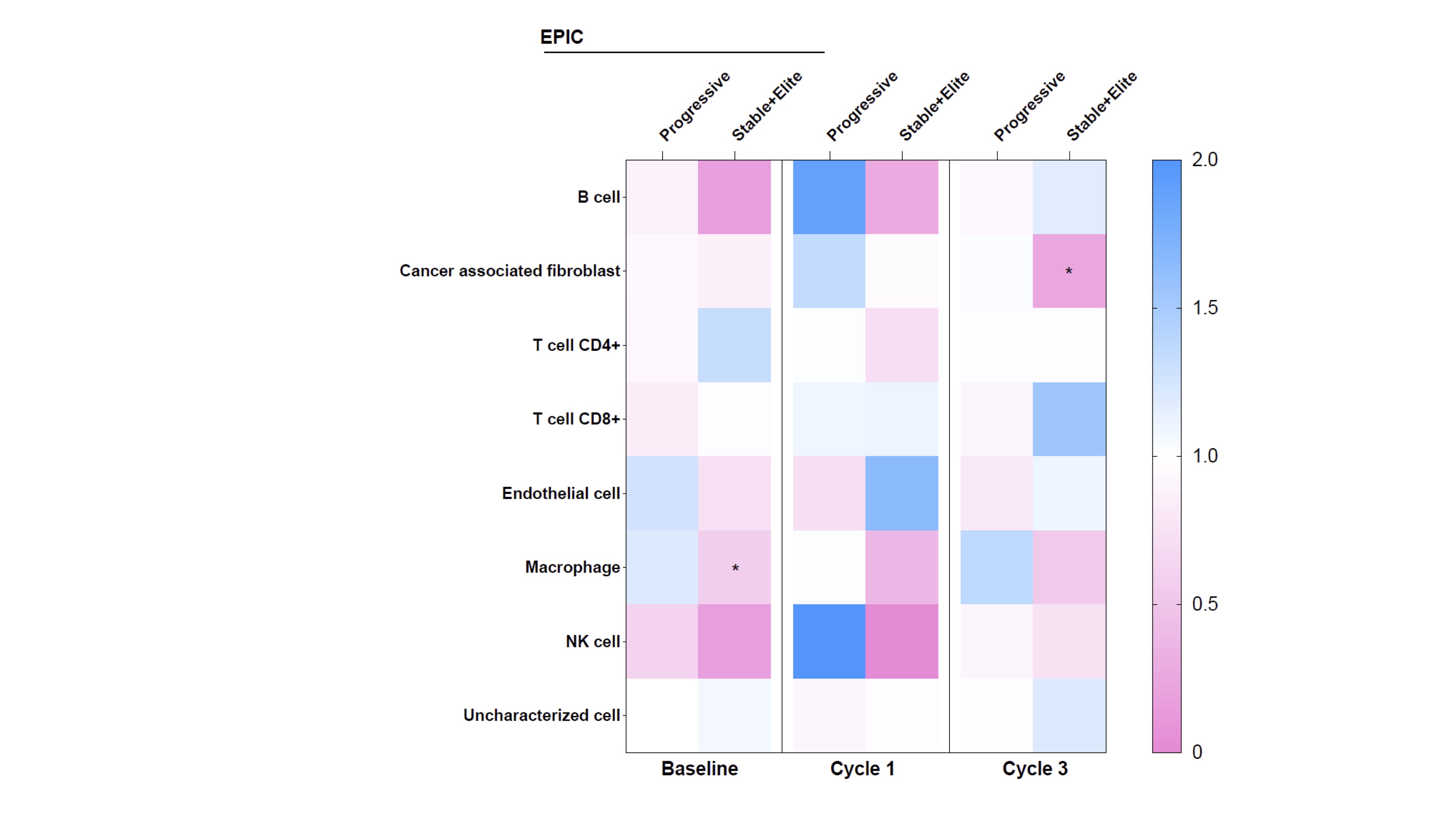


Figure. S8. Heatmap depicting scores per immune cell type (y-axis) according to subgroups classified according to treatment response and biopsy timepoint (x-axis) using EPIC.

Scores are normalized per cell type based on the calculation: mean of the subgroup / total mean. Stars indicate significant P-values obtained from the comparison of ’Progressive’ versus ‘Stable+Elite’ among samples obtained at the indicated timepoint, Kolmogorov-Smirnov test. *: P-value ≤ 0.05.


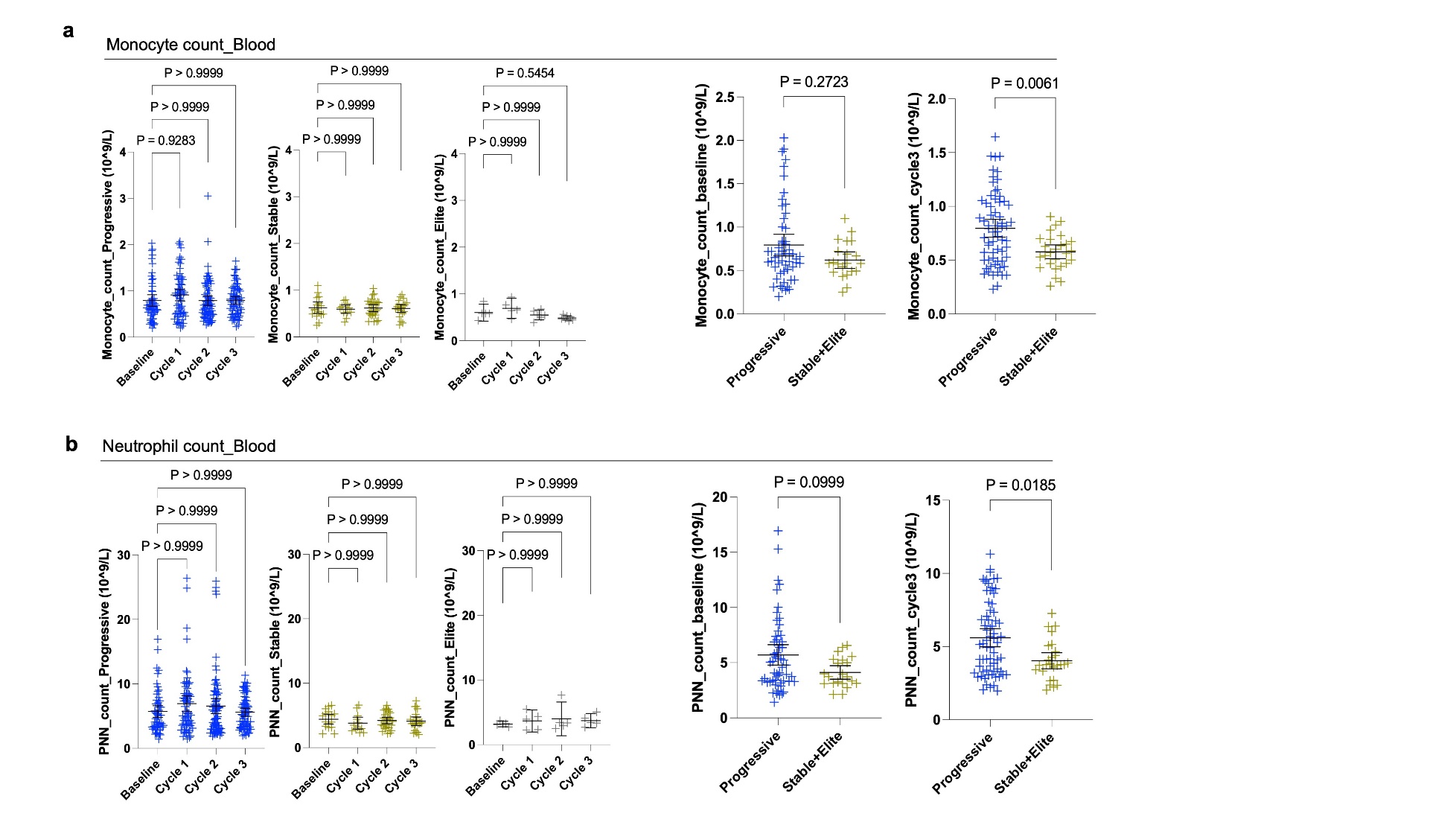


Figure. S9. Monocyte (a) and neutrophil (b) absolute counts according to the treatment cycle in patients with Progressive (blue, left panel), Stable (yellow, second panel) or Elite response (grey, third panel).

ROC curves and related metrics evaluating the predictive performance of absolute lymphocyte count (**a**) and monocyte/lymphocyte radio (**b**) to predict treament response (group ‘Progressive’ versus ‘Stable+Elite’). Baseline (left panels) and cycle 3 (right panels) data were evaluated.


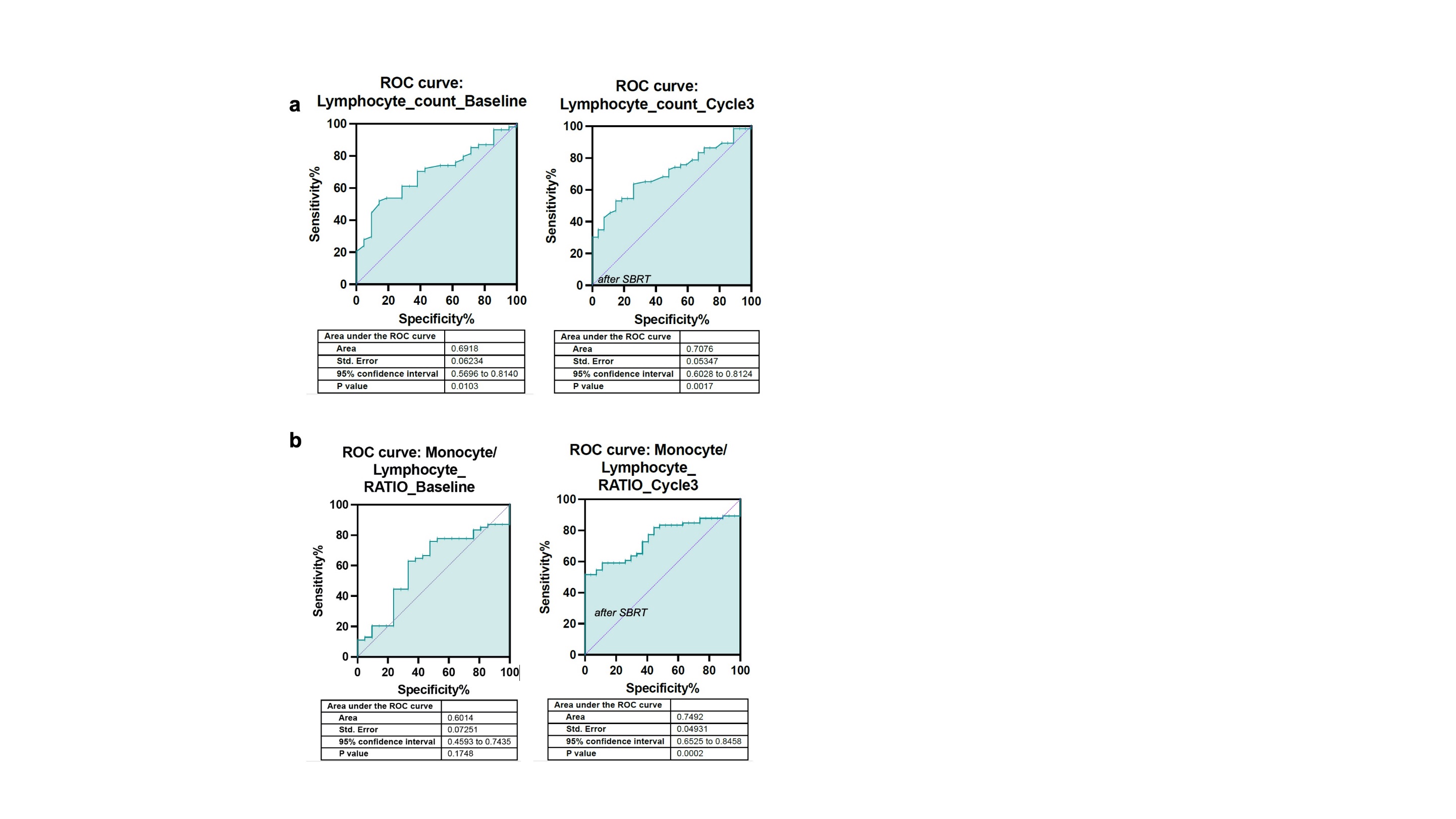


Figure. S10. ROC curves and related metrics evaluating the predictive performance of absolute lymphocyte count (a) and monocyte/lymphocyte radio (b) to predict treament response (group ‘Progressive’ versus ‘Stable+Elite’).

Baseline (left panels) and cycle 3 (right panels) data were evaluated.


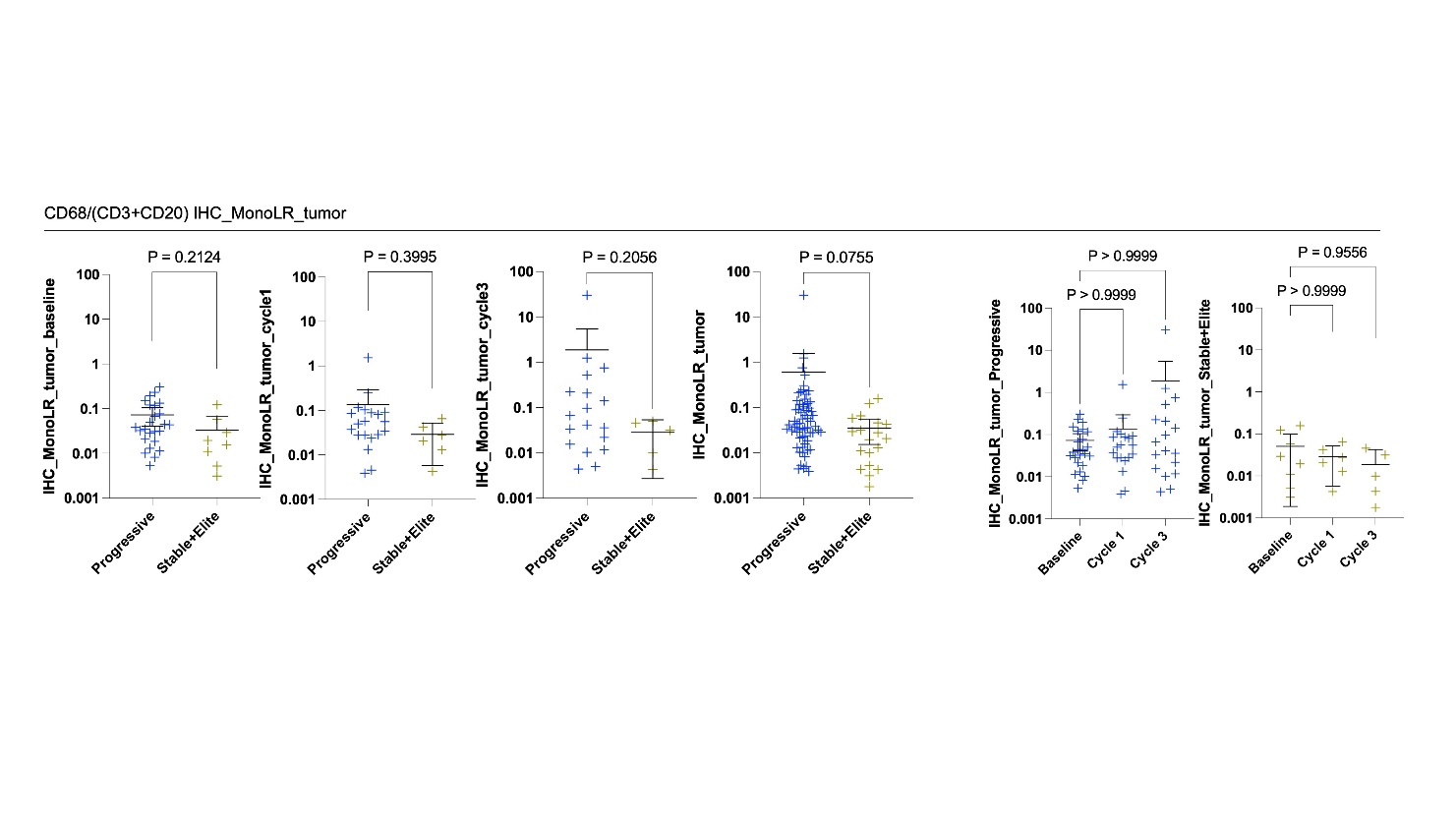


Figure. S11. Tumor monocytic-lineage/lymphocyte ratio (IHC_MonoLR_tumor) estimated from FFPE biopsies by immune multiplexing, according to the response group, from left to right at baseline, cycle 1, cycle 3 and considering all timepoints.

Right panels indicate IHC_MonoLR_tumor according to the treatment cycle among Progressive and Stable+Elite groups.


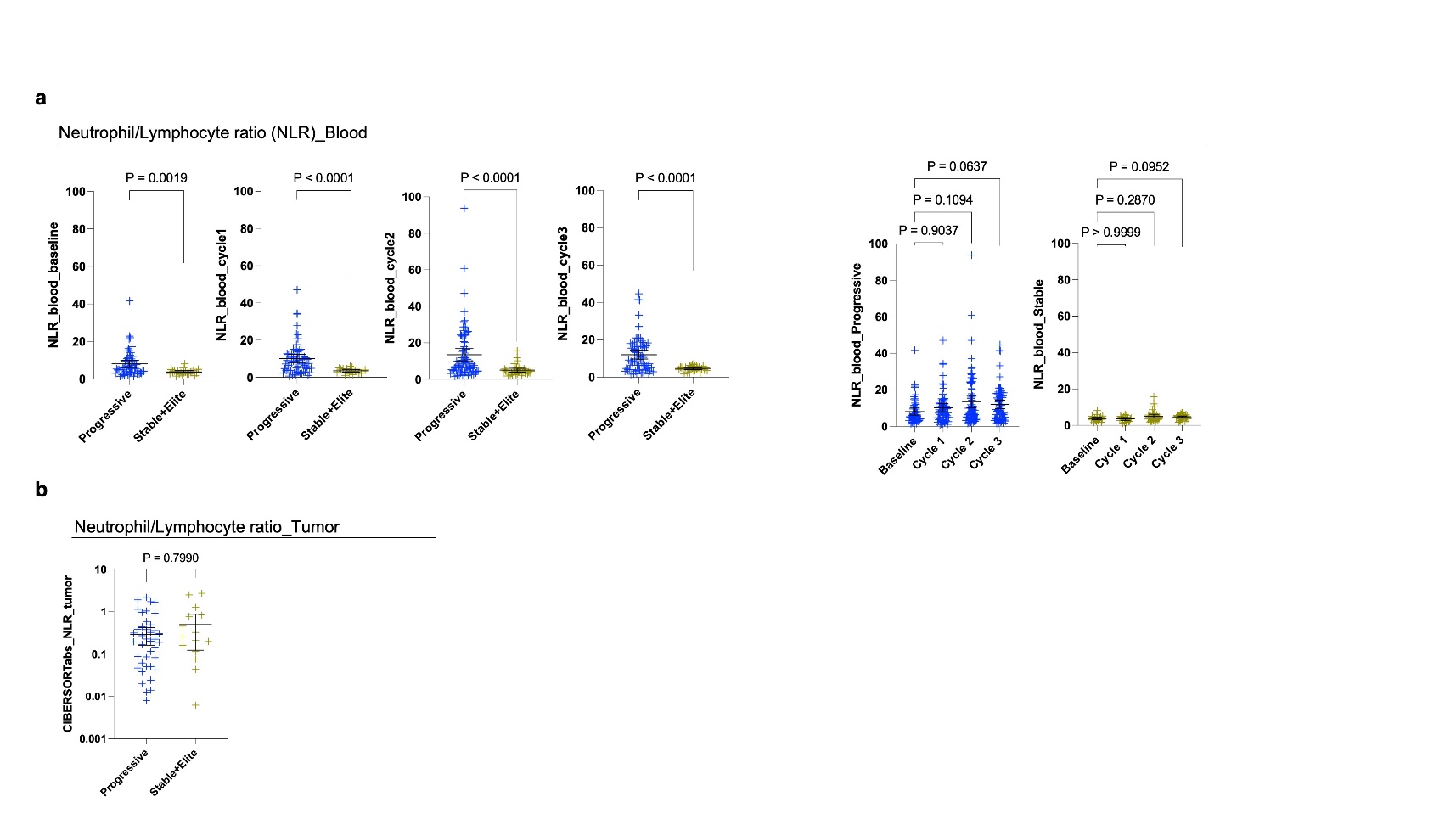


Figure. S12. NLR according to treatment response

Blood (a) and tumor (b) neutrophil/lymphocyte ratio (NLR) according to treatment response at (from left to right) baseline, cycle 1, cycle 2 and cycle 3. Right panels indicate NLR according to the treatment cycle among Progressive and Stable+Elite groups.

**
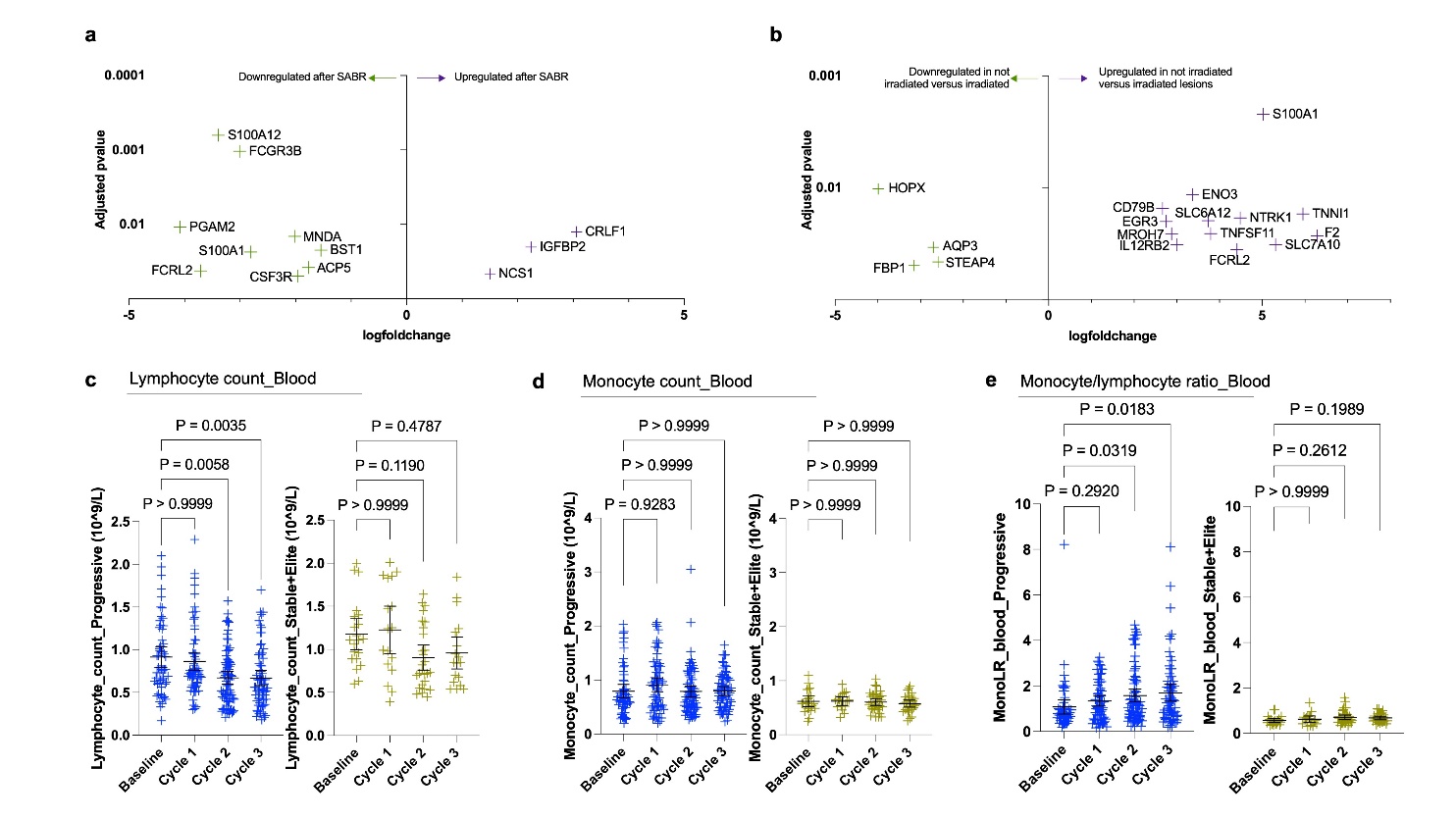
**

**Figure. S13. Effects of SBRT on tumor and blood from baseline**

**a,b**. Differential gene expression analysis of RNAseq data comparing transcription profiles of tumor biopsies obtained before (cycle 1) and after (cycle 3) SABR (**a**), and of tumor biopsies obtained at cycle 3 in irradiated lesions compared to out-of-the-field lesions (**b**), among 1775 selected genes. All genes differentially expressed between groups with adjusted p-value <0.05 are indicated by green or purple crosses, respectively when downregulated or upregulated after SABR therapy (**a**) or when downregulated or upregulated in out-of-the-field lesions compared to irradiated ones (**b**). **c,d**. Lymphocyte (**c**) and monocyte (**d**) absolute counts in the blood of patients with progressive (blue, left panel), stable or responsive disease (yellow, right panel), according to the treatment cycle, from baseline to cycle 3, each cycle corresponding to a 21-day period. Mean +/-95%CI, Kruskal-Wallis with Dunn’s multiple comparisons test. **e**. Blood monocyte/lymphocyte ratio in patients with progressive (blue, left panel) and stable or responsive disease (yellow, right panel), according to the treatment cycle, from baseline to cycle 3. Mean +/-95%CI, Kruskal-Wallis with Dunn’s multiple comparisons test.

Figure. S14. Leiomyosarcoma versus other subtypes: bulk RNAseq


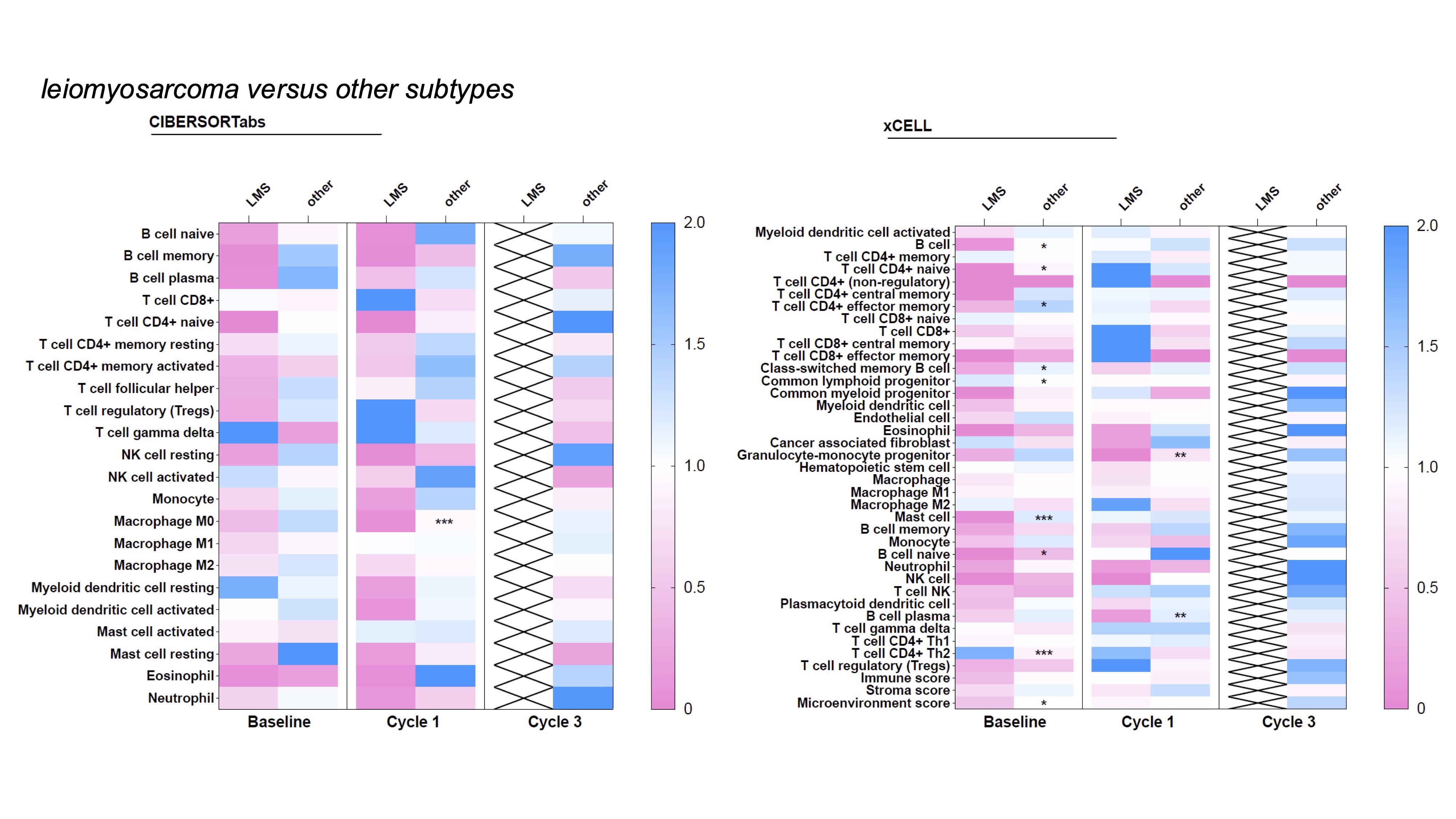


Heatmap depicting scores per immune cell type (y-axis) according to subgroups classified according to sarcoma subtype and biopsy timepoint (x-axis) using two independent published deconvolution tools, CIBERSORTabs (left panel) and xCELL (right panel). Scores are normalized per cell type based on the calculation: mean of the subgroup / total mean. Blue indicates higher values than the mean of samples, whereas pink indicates lower values. Stars indicate significant P-values obtained from the comparison of ’LMS’ versus ‘other subtypes’ among samples obtained at the indicated timepoint, Kolmogorov-Smirnov test. LMS: leiomyosarcoma.


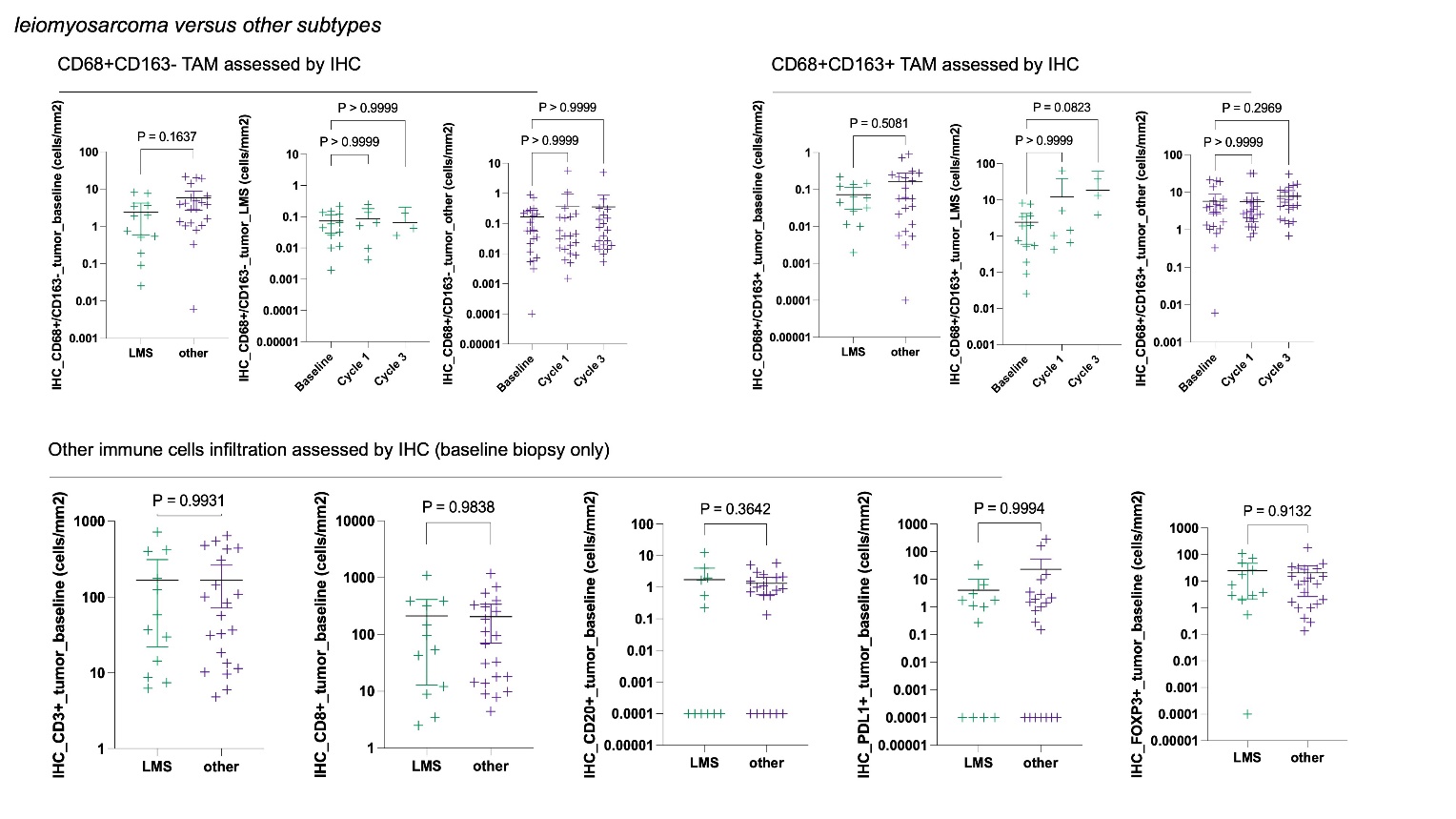


Figure. S15. Leiomyosarcoma versus other subtypes: immunohistochemistry

*(up) CD68/CD163 co-labelling assessed by multiplexed immunohistochemistry on tumor FFPE biopsies. CD68+/CD163- and immunosuppressive CD68+/CD163+ cell densities were automatically quantified by the HALO® software on digitalized slides. Are displayed: cell density at baseline according to sarcoma subtype (left panel), cell density according to biopsy timepoint among patients with LMS (middle panel) and among patients with another subtype of sarcoma (right panel). Green and purple crosses refer respectively to patients with LMS and with another sarcoma. Mean +/-95%CI. Kruskal-Wallis with Dunn’s multiple comparisons test. (bottom) Cell density of CD3+, CD8+, CD20+, PDL1+ and FOXP3+ cells at baseline according to sarcoma subtype.*


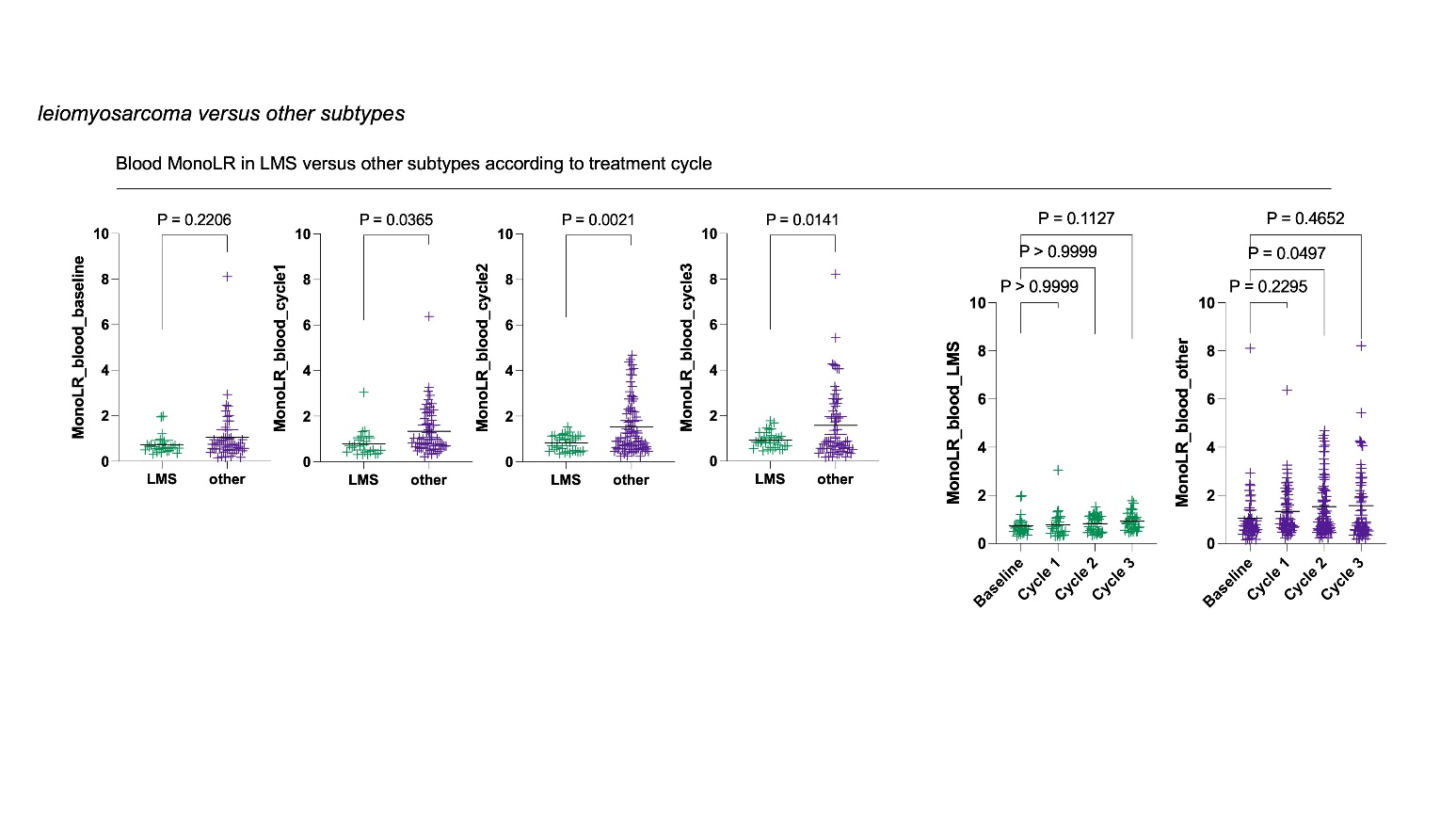


Figure. S16. Leiomyosarcoma versus other subtypes: blood values

(left) Monocyte/lymphocyte ratio (MonoLR) computed on blood values (monocyte absolute count / lymphocyte absolute count) from left to right: at baseline, cycle 1, cycle 2 and cycle 3, according to the sarcoma subtype (LMS versus another subtype). Mean +/-95%CI, Kolmogorov-Smirnov test. Green and purple crosses refer respectively to patients with LMS and with another sarcoma. (right) MonoLR according to treatment cycle among patients with LMS (green) and among patients with another sarcoma subtype.

**Table S1: Treatment-related (a: atezolizumab and b: SBRT) Adverse Events in Study Population per CTCAE**

| a |  |  |  |  |  |  |
| --- | --- | --- | --- | --- | --- | --- |
|  | | | | Grade | | |
| **SOC** | Prefered term |  | | 1 | 2 | 3 |
| **Blood and lymphatic system disorders** | Cholesterol Increased | . | . | 1 (2%) |  |  |
|  | CPK increased | . | . |  |  | 1 (2%) |
| **Eye disorders** | Conjunctivitis | . | . | 1 (2%) |  |  |
| **General disorders and administration site conditions** | Fever | . | . |  | 1 (2%) |  |
|  | Injection site reaction | . | . | 1 (2%) |  |  |
| **Hepatobiliary disorders** | Hepatic Cytolysis | . | . |  | 2 (3%) |  |
| **Investigations** | Alkaline phosphatase increased | . | . | 1 (2%) | 1 (2%) |  |
|  | Lipase increased | . | . | 1 (2%) | 1 (2%) |  |
| **Metabolism and nutrition disorders** | Anorexia | . | . | 2 (3%) |  |  |
| **Musculoskeletal and connective tissue disorders** | Arthralgia | . | . | 1 (2%) |  |  |
|  | Arthritis | . | . |  | 1 (2%) |  |
| **Nervous system disorders** | Paresthesia | . | . | 1 (2%) |  |  |
| **Skin and subcutaneous tissue disorders** | Rash maculo-papular | . | . | 1 (2%) |  |  |
|  |  |  |  |  |  |  |
| **b** |  |  |  |  |  |  |
|  | | | | Grade | |  |
| **SOC** | Prefered term |  | | 1 | 2 |  |
| **Metabolism and nutrition disorders** | Anorexia | . | . | 1 (2%) |  |  |
| **Musculoskeletal and connective tissue disorders** | Pain Flare | . | . | 1 (2%) |  |  |
| **Skin and subcutaneous tissue disorders** | Erythema | . | . | 1 (2%) |  |  |
|  | Oral Mucositis | . | . |  | 1 (2%) |  |

**Table S2: List of 1775 genes from the LM22 list as well as genes from the Hallmark gene sets obtained from the MSigDB database**.

Liberzon, A. et al. Molecular signatures database (MSigDB) 3.0. Bioinformatics **27**, 1739–1740 (2011)

| A2M |
| --- |
| AAAS |
| ABAT |
| ABCB1 |
| ABCB4 |
| ABCB9 |
| ABCC1 |
| ABCC5 |
| ABHD4 |
| ABI3BP |
| ACAA1 |
| ACAP1 |
| ACHE |
| ACKR3 |
| ACP5 |
| ACTA2 |
| ACVR1B |
| ACVR2A |
| ACVRL1 |
| ADA |
| ADAM12 |
| ADAM19 |
| ADAM28 |
| ADAMDEC1 |
| ADAMTS3 |
| ADCY6 |
| ADD1 |
| ADD3 |
| ADGRE1 |
| ADGRE2 |
| ADGRE3 |
| ADGRG3 |
| ADGRL2 |
| ADM |
| ADORA2B |
| ADRB2 |
| ADRM1 |
| AEN |
| AGER |
| AGGF1 |
| AGO2 |
| AGO4 |
| AHCY |
| AHNAK |
| AHR |
| AIF1 |
| AIFM3 |
| AIM2 |
| AK1 |
| AK3 |
| AK4 |
| AKAP12 |
| AKT3 |
| ALAS1 |
| ALCAM |
| ALDOA |
| ALDOB |
| ALDOC |
| ALOX15 |
| ALOX15B |
| ALOX5 |
| ALYREF |
| AMACR |
| AMD1 |
| AMPD1 |
| AMPD3 |
| AMPH |
| ANGPT4 |
| ANGPTL4 |
| ANKH |
| ANKRA2 |
| ANKRD55 |
| ANKZF1 |
| ANPEP |
| ANXA1 |
| ANXA2 |
| ANXA4 |
| AP2S1 |
| APAF1 |
| APBB2 |
| APLP1 |
| APOBEC3A |
| APOBEC3G |
| APOL3 |
| APOL6 |
| APOM |
| APP |
| APRT |
| AQP3 |
| AQP9 |
| AREG |
| ARHGAP22 |
| ARHGEF9 |
| ARL4A |
| ARL6IP1 |
| ARRB1 |
| ARRB2 |
| ASGR1 |
| ASGR2 |
| ASNS |
| ATF3 |
| ATOX1 |
| ATP2B1 |
| ATP2B4 |
| ATP2C1 |
| ATP6V1C1 |
| ATP6V1F |
| ATP7A |
| ATP8B4 |
| ATRN |
| ATRX |
| ATXN1 |
| ATXN8OS |
| AVPR1A |
| AZU1 |
| B3GALT6 |
| B4GALNT2 |
| BACH2 |
| BAIAP2 |
| BAK1 |
| BANK1 |
| BARX2 |
| BASP1 |
| BATF |
| BATF3 |
| BAX |
| BCAM |
| BCAN |
| BCAP31 |
| BCKDHB |
| BCL10 |
| BCL11B |
| BCL2 |
| BCL2A1 |
| BCL2L1 |
| BCL2L10 |
| BCL2L11 |
| BCL2L2 |
| BCL7A |
| BDNF |
| BEND5 |
| BFSP1 |
| BGN |
| BHLHE40 |
| BHLHE41 |
| BID |
| BIK |
| BIRC3 |
| BLCAP |
| BLK |
| BMF |
| BMP1 |
| BMP2 |
| BMP2K |
| BMPR1A |
| BMPR2 |
| BNIP3L |
| BOLA2 |
| BPI |
| BRAF |
| BRCA1 |
| BRF2 |
| BRS3 |
| BRSK2 |
| BSG |
| BST1 |
| BTG1 |
| BTG2 |
| BTG3 |
| BTNL8 |
| C11orf80 |
| C1orf54 |
| C3AR1 |
| C4BPB |
| C5AR1 |
| C5AR2 |
| CA12 |
| CA2 |
| CA8 |
| CACNA1A |
| CADM1 |
| CALD1 |
| CALU |
| CAMP |
| CANT1 |
| CAP2 |
| CAPG |
| CAPN3 |
| CASP1 |
| CASP2 |
| CASP3 |
| CASP4 |
| CASP5 |
| CASP6 |
| CASP7 |
| CASP8 |
| CASP9 |
| CAT |
| CAV1 |
| CAVIN1 |
| CAVIN3 |
| CBL |
| CCDC102B |
| CCK |
| CCL1 |
| CCL13 |
| CCL14 |
| CCL17 |
| CCL18 |
| CCL19 |
| CCL20 |
| CCL22 |
| CCL23 |
| CCL4 |
| CCL5 |
| CCL7 |
| CCL8 |
| CCN1 |
| CCN2 |
| CCN5 |
| CCNA1 |
| CCND1 |
| CCND2 |
| CCND3 |
| CCNE1 |
| CCNG1 |
| CCNG2 |
| CCNK |
| CCNO |
| CCP110 |
| CCR1 |
| CCR10 |
| CCR2 |
| CCR3 |
| CCR4 |
| CCR5 |
| CCR6 |
| CCR7 |
| CD14 |
| CD160 |
| CD180 |
| CD19 |
| CD1A |
| CD1B |
| CD1C |
| CD1D |
| CD1E |
| CD2 |
| CD209 |
| CD22 |
| CD244 |
| CD247 |
| CD27 |
| CD28 |
| CD300A |
| CD33 |
| CD36 |
| CD37 |
| CD38 |
| CD3D |
| CD3E |
| CD3G |
| CD4 |
| CD40 |
| CD40LG |
| CD44 |
| CD48 |
| CD5 |
| CD59 |
| CD6 |
| CD68 |
| CD69 |
| CD7 |
| CD70 |
| CD72 |
| CD79A |
| CD79B |
| CD80 |
| CD81 |
| CD82 |
| CD83 |
| CD86 |
| CD8A |
| CD8B |
| CD9 |
| CD96 |
| CDA |
| CDC25A |
| CDC25B |
| CDC34 |
| CDC42BPA |
| CDC42SE2 |
| CDC5L |
| CDC6 |
| CDCP1 |
| CDH11 |
| CDH12 |
| CDH13 |
| CDH2 |
| CDH6 |
| CDHR1 |
| CDK13 |
| CDK2 |
| CDK5R1 |
| CDK6 |
| CDKN1A |
| CDKN1B |
| CDKN1C |
| CDKN2A |
| CDKN2AIP |
| CDKN2B |
| CDKN2D |
| CDO1 |
| CDON |
| CEACAM3 |
| CEACAM8 |
| CEBPA |
| CEBPG |
| CELF2 |
| CEMP1 |
| CETN2 |
| CFLAR |
| CFP |
| CGRRF1 |
| CHI3L1 |
| CHI3L2 |
| CHKA |
| CHRNA5 |
| CHST15 |
| CHST2 |
| CHST3 |
| CHST7 |
| CISH |
| CITED2 |
| CKAP4 |
| CLC |
| CLCA2 |
| CLCA3P |
| CLCN2 |
| CLEC10A |
| CLEC2D |
| CLEC4A |
| CLEC7A |
| CLIC2 |
| CLP1 |
| CLTB |
| CLU |
| CMA1 |
| CMPK2 |
| CNP |
| CNTFR |
| COCH |
| COL11A1 |
| COL12A1 |
| COL16A1 |
| COL1A1 |
| COL1A2 |
| COL2A1 |
| COL3A1 |
| COL4A1 |
| COL4A2 |
| COL5A1 |
| COL5A2 |
| COL5A3 |
| COL6A1 |
| COL6A2 |
| COL6A3 |
| COL7A1 |
| COL8A2 |
| COLGALT1 |
| COLQ |
| COMP |
| COPA |
| COQ8A |
| COX17 |
| CP |
| CPA3 |
| CR2 |
| CREB5 |
| CREBBP |
| CREG1 |
| CRISP3 |
| CRLF1 |
| CRLF2 |
| CRTAM |
| CRYBB1 |
| CSF1 |
| CSF2 |
| CSF2RA |
| CSF2RB |
| CSF3R |
| CSRNP2 |
| CSRP2 |
| CST7 |
| CSTF3 |
| CTH |
| CTHRC1 |
| CTLA4 |
| CTNNB1 |
| CTSD |
| CTSF |
| CTSG |
| CTSV |
| CTSW |
| CTSZ |
| CXCL1 |
| CXCL10 |
| CXCL11 |
| CXCL12 |
| CXCL13 |
| CXCL2 |
| CXCL3 |
| CXCL5 |
| CXCL6 |
| CXCL8 |
| CXCL9 |
| CXCR1 |
| CXCR2 |
| CXCR4 |
| CXCR5 |
| CXCR6 |
| CYB5B |
| CYB5R1 |
| CYFIP1 |
| CYFIP2 |
| CYLD |
| CYP1A1 |
| CYP27A1 |
| CYP27B1 |
| DAB2 |
| DACH1 |
| DAD1 |
| DAP |
| DAP3 |
| DAPK2 |
| DBP |
| DCN |
| DCPS |
| DCSTAMP |
| DCTN4 |
| DCXR |
| DDAH1 |
| DDB1 |
| DDB2 |
| DDIT3 |
| DDIT4 |
| DDX21 |
| DEF6 |
| DEFA4 |
| DENND5A |
| DENND5B |
| DEPDC5 |
| DFFA |
| DGAT1 |
| DGCR8 |
| DGKA |
| DGUOK |
| DHRS11 |
| DHRS3 |
| DHX58 |
| DIABLO |
| DKK1 |
| DLC1 |
| DLG1 |
| DLG4 |
| DMAC2L |
| DNAJA1 |
| DNAJB1 |
| DNAJC3 |
| DNM1L |
| DNTT |
| DNTTIP2 |
| DPEP2 |
| DPP4 |
| DPYD |
| DPYSL3 |
| DPYSL4 |
| DRAM1 |
| DRC1 |
| DSC1 |
| DST |
| DTNA |
| DUSP1 |
| DUSP2 |
| DUT |
| DYRK1A |
| E2F5 |
| EAF2 |
| EBI3 |
| EBP |
| ECM1 |
| ECM2 |
| EDF1 |
| EDIL3 |
| EDN2 |
| EEF1AKMT1 |
| EFEMP1 |
| EFEMP2 |
| EFNA1 |
| EFNA3 |
| EFNA5 |
| EGFR |
| EGLN2 |
| EGR2 |
| EGR3 |
| EI24 |
| EIF1B |
| EIF2S3 |
| EIF5 |
| ELANE |
| ELL |
| ELN |
| ELOA |
| ELP1 |
| EMP1 |
| EMP3 |
| ENO1 |
| ENO2 |
| ENO3 |
| ENPP1 |
| EOMES |
| EPB41 |
| EPCAM |
| EPHA1 |
| EPHA2 |
| EPHX1 |
| EPN2 |
| EPS8L2 |
| ERBB2 |
| ERBB3 |
| ERCC1 |
| ERCC2 |
| ERCC3 |
| ERCC4 |
| ERCC5 |
| ERCC8 |
| EREG |
| ERO1A |
| ERRFI1 |
| ETF1 |
| ETFBKMT |
| ETS1 |
| ETV3 |
| ETV4 |
| EXT1 |
| F2 |
| F2R |
| F2RL2 |
| F3 |
| FAH |
| FAM124B |
| FAM162A |
| FAM174B |
| FAP |
| FAS |
| FASLG |
| FBLN1 |
| FBLN2 |
| FBLN5 |
| FBN1 |
| FBN2 |
| FBP1 |
| FBXL8 |
| FBXW7 |
| FCER1A |
| FCER2 |
| FCGR2B |
| FCGR3B |
| FCMR |
| FCN1 |
| FCRL2 |
| FDXR |
| FEN1 |
| FERMT2 |
| FES |
| FEZ1 |
| FFAR2 |
| FGF13 |
| FGF18 |
| FGF2 |
| FGL2 |
| FHL2 |
| FKBP4 |
| FLJ13197 |
| FLNA |
| FLT3LG |
| FLVCR2 |
| FMO1 |
| FMOD |
| FN1 |
| FOS |
| FOSB |
| FOSL2 |
| FOXC2 |
| FOXO3 |
| FOXP3 |
| FPR1 |
| FPR2 |
| FPR3 |
| FRK |
| FRMD4A |
| FRMD8 |
| FSTL1 |
| FSTL3 |
| FTL |
| FUCA1 |
| FURIN |
| FYN |
| FZD2 |
| FZD3 |
| FZD8 |
| G6PD |
| GAA |
| GABARAPL1 |
| GADD45A |
| GADD45B |
| GAL |
| GAL3ST4 |
| GALK1 |
| GALM |
| GALR1 |
| GAPDH |
| GAPDHS |
| GAS1 |
| GASK1B |
| GATA1 |
| GBE1 |
| GBP4 |
| GCH1 |
| GCK |
| GCLC |
| GCLM |
| GCNT1 |
| GCNT2 |
| GEM |
| GFI1 |
| GGH |
| GGT5 |
| GIPR |
| GJA1 |
| GLIPR1 |
| GLIPR2 |
| GLRX |
| GLRX2 |
| GLS |
| GLS2 |
| GM2A |
| GMPR2 |
| GNA15 |
| GNG7 |
| GNLY |
| GPC1 |
| GPC3 |
| GPC4 |
| GPI |
| GPR1 |
| GPR171 |
| GPR18 |
| GPR183 |
| GPR19 |
| GPR25 |
| GPR65 |
| GPR83 |
| GPX1 |
| GPX2 |
| GPX3 |
| GPX4 |
| GPX7 |
| GRAP2 |
| GRB2 |
| GREM1 |
| GRHPR |
| GRINA |
| GRK5 |
| GRPEL1 |
| GSDME |
| GSN |
| GSR |
| GSTM1 |
| GSTO1 |
| GSTT1 |
| GTF2A2 |
| GTF2B |
| GTF2F1 |
| GTF2H1 |
| GTF2H3 |
| GTF2H5 |
| GTF3C5 |
| GUCY1B1 |
| GUCY2D |
| GUK1 |
| GUSBP11 |
| GYPE |
| GYS1 |
| GZMA |
| GZMB |
| GZMH |
| GZMK |
| GZMM |
| H1-0 |
| H1-2 |
| H2AC25 |
| H2AC8 |
| H2AJ |
| H2AX |
| H2BC8 |
| HAL |
| HAS1 |
| HAS2 |
| HAX1 |
| HBEGF |
| HCK |
| HCLS1 |
| HDAC3 |
| HDC |
| HDLBP |
| HESX1 |
| HEXA |
| HEXIM1 |
| HGF |
| HHEX |
| HIC1 |
| HINT1 |
| HIPK2 |
| HK1 |
| HK2 |
| HK3 |
| HLA-DOB |
| HLA-DQA1 |
| HLA-F |
| HMGB2 |
| HMGB3P30 |
| HMOX1 |
| HMOX2 |
| HNMT |
| HNRNPU |
| HOPX |
| HOXA1 |
| HOXB9 |
| HPGDS |
| HPRT1 |
| HPSE |
| HRAS |
| HRH1 |
| HS3ST1 |
| HSPA13 |
| HSPA2 |
| HSPA4L |
| HSPA5 |
| HSPA6 |
| HSPB1 |
| HTR2B |
| HTR7 |
| HTRA1 |
| HUWE1 |
| HYAL2 |
| HYCC2 |
| ICA1 |
| ICAM1 |
| ICOS |
| ID1 |
| ID2 |
| IDO1 |
| IDS |
| IER3 |
| IER5 |
| IFI30 |
| IFI44L |
| IFITM3 |
| IFNA10 |
| IFNAR1 |
| IFNB1 |
| IFNG |
| IFNGR1 |
| IFNGR2 |
| IGF1R |
| IGF2R |
| IGFBP1 |
| IGFBP2 |
| IGFBP3 |
| IGFBP4 |
| IGFBP5 |
| IGFBP6 |
| IGHD |
| IGHE |
| IGHM |
| IGKC |
| IGLL3P |
| IGSF6 |
| IKZF2 |
| IKZF4 |
| IL10 |
| IL10RA |
| IL10RB |
| IL12B |
| IL12RB1 |
| IL12RB2 |
| IL13 |
| IL13RA1 |
| IL15 |
| IL15RA |
| IL17A |
| IL17RA |
| IL17RB |
| IL18 |
| IL18R1 |
| IL18RAP |
| IL1A |
| IL1B |
| IL1R1 |
| IL1R2 |
| IL1RL1 |
| IL21 |
| IL26 |
| IL2RA |
| IL2RB |
| IL2RG |
| IL3 |
| IL32 |
| IL3RA |
| IL4 |
| IL4R |
| IL5 |
| IL5RA |
| IL6 |
| IL6ST |
| IL7 |
| IL7R |
| IL9 |
| IL9R |
| ILVBL |
| IMPDH2 |
| INHA |
| INHBA |
| INHBB |
| INHBE |
| INKA2 |
| INPP4B |
| INSIG1 |
| IP6K2 |
| IPCEF1 |
| IRAG2 |
| IRAK1 |
| IRF1 |
| IRF4 |
| IRF6 |
| IRF8 |
| IRF9 |
| IRS1 |
| IRS2 |
| ISCU |
| ISG20 |
| ITGA2 |
| ITGA4 |
| ITGA5 |
| ITGA6 |
| ITGAE |
| ITGAV |
| ITGB1 |
| ITGB3 |
| ITGB4 |
| ITGB5 |
| ITIH5 |
| ITK |
| ITPA |
| JAG2 |
| JMJD6 |
| JUN |
| JUNB |
| KALRN |
| KCNA3 |
| KCNG2 |
| KCNH2 |
| KCNMA1 |
| KDELR3 |
| KDM3A |
| KIF13B |
| KIF5A |
| KIR2DL1 |
| KIR2DL4 |
| KIR2DS4 |
| KIR3DL2 |
| KIRREL1 |
| KIT |
| KLF4 |
| KLF6 |
| KLF7 |
| KLHDC3 |
| KLHL24 |
| KLK8 |
| KLRB1 |
| KLRC3 |
| KLRC4 |
| KLRD1 |
| KLRF1 |
| KLRG1 |
| KLRK1 |
| KRT17 |
| KRT18 |
| KRT18P50 |
| KYNU |
| LAG3 |
| LAIR2 |
| LALBA |
| LAMA1 |
| LAMA2 |
| LAMA3 |
| LAMC1 |
| LAMC2 |
| LAMP3 |
| LAMTOR5 |
| LARGE1 |
| LAT |
| LCK |
| LCLAT1 |
| LDHA |
| LDHB |
| LDHC |
| LDLR |
| LEF1 |
| LEPR |
| LGALS1 |
| LGALS3 |
| LHCGR |
| LHX2 |
| LIF |
| LIG1 |
| LILRA2 |
| LILRA3 |
| LILRA4 |
| LILRB2 |
| LIME1 |
| LINC00597 |
| LINC00921 |
| LMNA |
| LOC100130100 |
| LOC126987 |
| LOX |
| LOXL1 |
| LOXL2 |
| LPAR1 |
| LRIG1 |
| LRMP |
| LRP1 |
| LRRC15 |
| LRRC8C |
| LSP1 |
| LST1 |
| LTA |
| LTB |
| LTBP1 |
| LTBR |
| LTC4S |
| LUM |
| LXN |
| LY86 |
| LY9 |
| LYN |
| MACF1 |
| MADD |
| MAFF |
| MAGEA11 |
| MAGEE1 |
| MAGI2 |
| MAK |
| MAN1A1 |
| MANEA |
| MAOA |
| MAP1B |
| MAP2K5 |
| MAP3K1 |
| MAP3K13 |
| MAP3K8 |
| MAP4K1 |
| MAP4K2 |
| MAP6 |
| MAP9 |
| MAPK14 |
| MAPK8IP2 |
| MAPKAPK2 |
| MAPKAPK3 |
| MARCHF3 |
| MARCO |
| MARK2 |
| MAST1 |
| MATN2 |
| MATN3 |
| MBL2 |
| MBP |
| MCL1 |
| MCM7 |
| MDM2 |
| MEFV |
| MEP1A |
| MEST |
| MET |
| MFAP5 |
| MGAM |
| MGAT1 |
| MGLL |
| MGMT |
| MGP |
| MGST1 |
| MICAL3 |
| MIF |
| MIOS |
| MKNK2 |
| MMP1 |
| MMP12 |
| MMP14 |
| MMP16 |
| MMP2 |
| MMP25 |
| MMP3 |
| MMP9 |
| MNDA |
| MPC2 |
| MPG |
| MPO |
| MROH7 |
| MRPL23 |
| MRPL40 |
| MRPS31 |
| MS4A1 |
| MS4A2 |
| MS4A3 |
| MS4A6A |
| MSC |
| MSRA |
| MSX1 |
| MT1E |
| MT2A |
| MTA1 |
| MUC1 |
| MXD1 |
| MXD4 |
| MXI1 |
| MXRA5 |
| MYB |
| MYC |
| MYD88 |
| MYH9 |
| MYL9 |
| MYLK |
| MYO1C |
| MYO1E |
| MZB1 |
| NAALADL1 |
| NAGK |
| NAT1 |
| NCAN |
| NCBP2 |
| NCF2 |
| NCOA3 |
| NCR3 |
| NCS1 |
| NDRG1 |
| NDST1 |
| NDST2 |
| NDUFA6 |
| NDUFB4 |
| NDUFS2 |
| NEDD4L |
| NEDD9 |
| NEFH |
| NEK7 |
| NELFB |
| NELFCD |
| NELFE |
| NFE2 |
| NFIB |
| NFIL3 |
| NFKB1 |
| NFKBIA |
| NFKBIZ |
| NFX1 |
| NHLH2 |
| NID2 |
| NINJ1 |
| NIPBL |
| NIPSNAP3B |
| NKG7 |
| NKX2-5 |
| NLRP3 |
| NMBR |
| NME1 |
| NME3 |
| NME4 |
| NME8 |
| NNMT |
| NOCT |
| NOD2 |
| NOL8 |
| NOP2 |
| NOTCH1 |
| NOTCH2 |
| NOX3 |
| NPAS1 |
| NPIPB15 |
| NPL |
| NPR2 |
| NPTX2 |
| NPTXR |
| NQO1 |
| NR1D2 |
| NR3C1 |
| NR4A1 |
| NR4A3 |
| NRP1 |
| NT5C |
| NT5C3A |
| NT5E |
| NTM |
| NTN3 |
| NTRK1 |
| NTRK3 |
| NUDT15 |
| NUDT21 |
| NUDT9 |
| NUP58 |
| NUPR1 |
| NXF1 |
| ODC1 |
| OLFM1 |
| ONECUT1 |
| ORC1 |
| OSGIN1 |
| OSM |
| OSMR |
| OXSR1 |
| OXTR |
| P2RX1 |
| P2RX4 |
| P2RX5 |
| P2RY10 |
| P2RY13 |
| P2RY14 |
| P2RY2 |
| P3H1 |
| P4HA1 |
| P4HA2 |
| PADI4 |
| PAK1 |
| PAM |
| PAQR5 |
| PARP2 |
| PASK |
| PAX7 |
| PBXIP1 |
| PCDHA5 |
| PCK1 |
| PCNA |
| PCOLCE |
| PCOLCE2 |
| PDAP1 |
| PDCD1 |
| PDCD1LG2 |
| PDCD2L |
| PDCD4 |
| PDE4B |
| PDE6C |
| PDE6G |
| PDGFA |
| PDGFB |
| PDGFC |
| PDGFRB |
| PDK1 |
| PDK3 |
| PDLIM1 |
| PDLIM3 |
| PDLIM4 |
| PDLIM5 |
| PEA15 |
| PENK |
| PERP |
| PEX14 |
| PF4 |
| PFKFB3 |
| PFKL |
| PFKP |
| PFN2 |
| PGAM2 |
| PGF |
| PGGHG |
| PGK1 |
| PGLYRP1 |
| PGM1 |
| PGM2 |
| PHF3 |
| PHKG1 |
| PHLDA1 |
| PHLDA3 |
| PHTF2 |
| PIAS3 |
| PIDD1 |
| PIK3CD |
| PIK3IP1 |
| PIK3R3 |
| PIK3R5 |
| PIM1 |
| PITPNC1 |
| PKD2L2 |
| PKLR |
| PKP1 |
| PLA1A |
| PLA2G2A |
| PLA2G7 |
| PLAC8 |
| PLAGL1 |
| PLAT |
| PLAUR |
| PLCB2 |
| PLCB4 |
| PLCH2 |
| PLCL1 |
| PLEC |
| PLEKHF1 |
| PLEKHG3 |
| PLIN2 |
| PLK2 |
| PLK3 |
| PLOD1 |
| PLOD2 |
| PLOD3 |
| PLPP1 |
| PLPP3 |
| PLPPR4 |
| PLSCR1 |
| PLXNB2 |
| PMAIP1 |
| PMCH |
| PMEPA1 |
| PMM1 |
| PMP22 |
| PNOC |
| PNP |
| PNRC1 |
| POLA1 |
| POLA2 |
| POLB |
| POLD1 |
| POLD3 |
| POLD4 |
| POLE3 |
| POLE4 |
| POLG2 |
| POLH |
| POLL |
| POLR1C |
| POLR1D |
| POLR1H |
| POLR2A |
| POLR2C |
| POLR2D |
| POLR2E |
| POLR2F |
| POLR2G |
| POLR2H |
| POLR2I |
| POLR2J |
| POLR2K |
| POLR3C |
| POLR3GL |
| POM121 |
| POSTN |
| POU2F1 |
| PPARG |
| PPARGC1A |
| PPAT |
| PPBP |
| PPFIA4 |
| PPFIBP1 |
| PPIB |
| PPIF |
| PPM1D |
| PPP1R15A |
| PPP1R2 |
| PPP1R3C |
| PPP2R5B |
| PPP3R1 |
| PPT1 |
| PRAF2 |
| PRDM2 |
| PRDX1 |
| PRDX2 |
| PRDX4 |
| PRDX5 |
| PRDX6 |
| PRF1 |
| PRG2 |
| PRIM1 |
| PRKAB1 |
| PRKACA |
| PRKAR2B |
| PRKCA |
| PRKCD |
| PRKCE |
| PRKCH |
| PRMT2 |
| PRNP |
| PROCR |
| PRPF3 |
| PRR5L |
| PRRX1 |
| PRSS2 |
| PSEN1 |
| PSEN2 |
| PSG2 |
| PSMC3 |
| PTCH1 |
| PTEN |
| PTGDR |
| PTGER2 |
| PTGFR |
| PTGIR |
| PTH1R |
| PTHLH |
| PTK2 |
| PTPA |
| PTPN1 |
| PTPN11 |
| PTPN14 |
| PTPN2 |
| PTPN21 |
| PTPRCAP |
| PTPRD |
| PTPRE |
| PTPRG |
| PTPRM |
| PTRH2 |
| PTX3 |
| PUS1 |
| PVR |
| PVRIG |
| PVT1 |
| PYGM |
| QPCT |
| QSOX1 |
| RAB27A |
| RAB27B |
| RAB40C |
| RABGAP1L |
| RACK1 |
| RAD51 |
| RAD51C |
| RAD52 |
| RAD9A |
| RADX |
| RAE1 |
| RALA |
| RALGDS |
| RALGPS2 |
| RAP2B |
| RARA |
| RASA2 |
| RASA3 |
| RASGRP1 |
| RASGRP2 |
| RASGRP3 |
| RASSF4 |
| RB1 |
| RBPJ |
| RBPMS |
| RBX1 |
| RCAN3 |
| RCHY1 |
| REG1A |
| RELA |
| REN |
| RENBP |
| REPS2 |
| RET |
| RETSAT |
| REV3L |
| RFC2 |
| RFC3 |
| RFC4 |
| RFC5 |
| RGS1 |
| RGS13 |
| RGS16 |
| RGS4 |
| RHBDF2 |
| RHOB |
| RHOH |
| RHOT2 |
| RIPOR2 |
| RNASE2 |
| RNASE6 |
| RNASEL |
| RND3 |
| RNF19B |
| RNH1 |
| RNMT |
| ROCK1 |
| RORA |
| RPA2 |
| RPA3 |
| RPL10L |
| RPL18 |
| RPL36 |
| RPL3P7 |
| RPN1 |
| RPS12 |
| RPS27L |
| RRAD |
| RRAGD |
| RRM2B |
| RRP12 |
| RRP8 |
| RRP9 |
| RSAD2 |
| RUBCNL |
| RUNX1 |
| RXRA |
| RXRB |
| RYR1 |
| S100A1 |
| S100A10 |
| S100A12 |
| S100A4 |
| S1PR5 |
| SAC3D1 |
| SAMSN1 |
| SAP30 |
| SAT1 |
| SATB1 |
| SBNO2 |
| SC5D |
| SCAF4 |
| SCAF8 |
| SCARB1 |
| SCG2 |
| SCHIP1 |
| SCN8A |
| SCN9A |
| SDC1 |
| SDC2 |
| SDC3 |
| SDC4 |
| SDCBP |
| SEC31B |
| SEC61A1 |
| SELENBP1 |
| SELENOS |
| SELENOW |
| SELL |
| SELP |
| SERGEF |
| SERPINB5 |
| SERPINB6 |
| SERPINC1 |
| SERPINE1 |
| SERPINE2 |
| SERPINH1 |
| SERTAD3 |
| SESN1 |
| SF3A3 |
| SFMBT1 |
| SFN |
| SFRP1 |
| SFRP4 |
| SGCB |
| SGCD |
| SGCG |
| SH2D1A |
| SH3BGRL2 |
| SHE |
| SHOX2 |
| SIAH2 |
| SIGLEC1 |
| SIGMAR1 |
| SIK1 |
| SIPA1L1 |
| SIRPG |
| SKA1 |
| SKAP1 |
| SLAMF1 |
| SLAMF8 |
| SLC12A1 |
| SLC12A8 |
| SLC15A3 |
| SLC19A2 |
| SLC1A5 |
| SLC20A1 |
| SLC22A18 |
| SLC25A1 |
| SLC25A4 |
| SLC29A2 |
| SLC2A1 |
| SLC2A3 |
| SLC2A5 |
| SLC2A6 |
| SLC35D1 |
| SLC37A4 |
| SLC39A8 |
| SLC3A2 |
| SLC6A12 |
| SLC6A6 |
| SLC6A8 |
| SLC7A1 |
| SLC7A10 |
| SLC7A11 |
| SLCO5A1 |
| SLIT2 |
| SLIT3 |
| SMAD3 |
| SMAD5 |
| SMAD7 |
| SMPD3 |
| SMPDL3A |
| SMPDL3B |
| SNAI2 |
| SNAPC4 |
| SNAPC5 |
| SNTB1 |
| SNX14 |
| SNX9 |
| SOCS1 |
| SOCS2 |
| SOCS3 |
| SOD1 |
| SOD2 |
| SP1 |
| SP140 |
| SPAG4 |
| SPARC |
| SPHK1 |
| SPIB |
| SPOCK1 |
| SPOCK2 |
| SPOP |
| SPP1 |
| SPR |
| SPRED2 |
| SPRY4 |
| SPTAN1 |
| SQSTM1 |
| SRI |
| SRPX |
| SRSF6 |
| SRXN1 |
| SSRP1 |
| SSX1 |
| ST14 |
| ST3GAL4 |
| ST3GAL6 |
| ST6GALNAC4 |
| ST8SIA1 |
| STAM2 |
| STAP1 |
| STARD3 |
| STAT1 |
| STAT2 |
| STAT3 |
| STBD1 |
| STC1 |
| STC2 |
| STEAP3 |
| STEAP4 |
| STIP1 |
| STK25 |
| STOM |
| STX3 |
| STXBP6 |
| SULT1A1 |
| SULT2B1 |
| SUPT4H1 |
| SUPT5H |
| SURF1 |
| SWAP70 |
| SYNE1 |
| SYNGR2 |
| SYNJ2 |
| SYT11 |
| TACR3 |
| TAF10 |
| TAF12 |
| TAF13 |
| TAF1C |
| TAF6 |
| TAF9 |
| TAGLN |
| TAP1 |
| TARBP2 |
| TARDBPP1 |
| TARS1 |
| TAX1BP3 |
| TBX21 |
| TCF7 |
| TCHH |
| TCL1A |
| TCN2 |
| TEC |
| TENT4A |
| TEP1 |
| TES |
| TFPI |
| TFPI2 |
| TFRC |
| TGFA |
| TGFB1 |
| TGFB2 |
| TGFB3 |
| TGFBI |
| TGFBR2 |
| TGFBR3 |
| TGFBRAP1 |
| TGM2 |
| TGM5 |
| THBS1 |
| THBS2 |
| THY1 |
| TIAM1 |
| TIMP1 |
| TIMP2 |
| TIMP3 |
| TIPARP |
| TJP1 |
| TK2 |
| TKTL1 |
| TLR2 |
| TLR7 |
| TLR8 |
| TM4SF1 |
| TM7SF3 |
| TMBIM6 |
| TMED2 |
| TMEM156 |
| TMEM255A |
| TMEM45A |
| TNC |
| TNF |
| TNFAIP3 |
| TNFAIP6 |
| TNFRSF10C |
| TNFRSF11A |
| TNFRSF11B |
| TNFRSF12A |
| TNFRSF13B |
| TNFRSF17 |
| TNFRSF18 |
| TNFRSF1A |
| TNFRSF1B |
| TNFRSF21 |
| TNFRSF4 |
| TNFRSF8 |
| TNFRSF9 |
| TNFSF10 |
| TNFSF11 |
| TNFSF14 |
| TNFSF9 |
| TNIP3 |
| TNNI1 |
| TOB1 |
| TOGARAM1 |
| TOP2A |
| TP53 |
| TP63 |
| TPBG |
| TPD52 |
| TPD52L1 |
| TPI1 |
| TPM1 |
| TPM2 |
| TPM4 |
| TPRKB |
| TPSAB1 |
| TPST2 |
| TRAC |
| TRAF1 |
| TRAF4 |
| TRAFD1 |
| TRAT1 |
| TRAV12-2 |
| TRAV13-1 |
| TRAV13-2 |
| TRAV21 |
| TRAV8-6 |
| TRAV9-2 |
| TRBC1 |
| TRDC |
| TREM1 |
| TREM2 |
| TREML2 |
| TRIAP1 |
| TRIB2 |
| TRIB3 |
| TRPM4 |
| TRPM6 |
| TSC22D1 |
| TSG101 |
| TSHR |
| TSPO |
| TSPYL2 |
| TST |
| TTC38 |
| TTC39B |
| TUBA4A |
| TWSG1 |
| TXK |
| TXN |
| TXNIP |
| TXNRD1 |
| TXNRD2 |
| TYK2 |
| TYMS |
| TYR |
| TYRO3 |
| UBASH3A |
| UCK2 |
| UGP2 |
| UGT1A8 |
| UGT2B17 |
| UMPS |
| UPF3B |
| UPK3A |
| UPP1 |
| UROD |
| USP11 |
| VAMP8 |
| VAV2 |
| VCAM1 |
| VCAN |
| VDAC2 |
| VDR |
| VEGFA |
| VEGFC |
| VHL |
| VILL |
| VIM |
| VLDLR |
| VPREB3 |
| VPS28 |
| VPS37B |
| VPS37D |
| VWA5A |
| WDR37 |
| WEE1 |
| WIPF1 |
| WIZ |
| WLS |
| WNT5A |
| WNT5B |
| WNT7A |
| WRAP73 |
| WSB1 |
| WWP1 |
| XBP1 |
| XIAP |
| XPC |
| XPNPEP1 |
| YKT6 |
| YTHDC1 |
| ZAP70 |
| ZBP1 |
| ZBTB10 |
| ZBTB16 |
| ZBTB32 |
| ZFP36 |
| ZFP36L1 |
| ZFP36L2 |
| ZMAT3 |
| ZMIZ1 |
| ZNF135 |
| ZNF165 |
| ZNF204P |
| ZNF222 |
| ZNF286A |
| ZNF292 |
| ZNF324 |
| ZNF365 |
| ZNF442 |
| ZNF707 |
| ZWINT |
|  |
|  |
|  |
|  |
|  |
|  |

**Table S3: List of 269/1775 genes dysregulated in progressive patients compared with biopsies of patients who had a clinical benefit**

| GENE | Log_foldchange | Adj_Pval |
| --- | --- | --- |
| MMP12 | 4,798250802 | 1,37E-06 |
| RASSF4 | 2,110818069 | 3,46E-06 |
| CD6 | 3,344796126 | 1,00E-05 |
| MMP9 | 3,217275112 | 1,33E-05 |
| LAG3 | 2,826927395 | 1,33E-05 |
| ADAMDEC1 | 3,382239717 | 1,33E-05 |
| ABCB9 | 2,250678681 | 2,86E-05 |
| ASGR2 | 3,897454992 | 4,60E-05 |
| FBP1 | 2,82613767 | 5,01E-05 |
| CXCL13 | 4,558128215 | 5,07E-05 |
| ASGR1 | 3,390162036 | 5,07E-05 |
| CD72 | 1,929350759 | 8,18E-05 |
| PTX3 | 3,056442709 | 8,18E-05 |
| PNOC | 4,285242719 | 8,70E-05 |
| MUC1 | 2,962816108 | 0,00011611 |
| GCLM | -1,280012779 | 0,000123192 |
| SSX1 | 5,51553326 | 0,00017642 |
| IGFBP1 | 4,936464842 | 0,000203424 |
| FASLG | 3,090720368 | 0,000215578 |
| NR1D2 | -0,91982568 | 0,000314841 |
| IDO1 | 2,909384099 | 0,000364389 |
| CXCL8 | 2,804966483 | 0,000397761 |
| ERO1A | 1,011879878 | 0,00047326 |
| SLC2A5 | 1,877443424 | 0,000477109 |
| KLF4 | -1,70271674 | 0,000545293 |
| TNIP3 | 3,186605483 | 0,000556431 |
| COLGALT1 | 1,110023262 | 0,000691117 |
| FCER2 | 4,662649007 | 0,000709809 |
| SLAMF8 | 1,921036368 | 0,000780266 |
| TNFSF11 | 3,093820128 | 0,000821516 |
| SPP1 | 2,126763559 | 0,000829861 |
| EDN2 | 4,309582693 | 0,000848826 |
| TGFBR3 | -1,655019455 | 0,000850673 |
| IL2RA | 2,042473736 | 0,000923663 |
| CD1A | 3,995927822 | 0,000931343 |
| PLOD1 | 1,156842548 | 0,000962634 |
| RNASE2 | 2,399333581 | 0,001047146 |
| MSC | 1,927223037 | 0,001062167 |
| TNFRSF9 | 2,466805654 | 0,001084378 |
| ARL4A | -1,253718097 | 0,001158371 |
| POLH | -0,943232678 | 0,001187811 |
| IL9R | 3,372086395 | 0,001247717 |
| SERPINE2 | 2,165681794 | 0,001247717 |
| SLC2A3 | 1,397799817 | 0,001257956 |
| PLEKHF1 | 1,386930077 | 0,001257956 |
| QSOX1 | 1,27398947 | 0,001271427 |
| CST7 | 1,849076141 | 0,001271427 |
| PIK3CD | 1,840362952 | 0,001307425 |
| VPS37B | 1,002533123 | 0,001330138 |
| HSPA4L | -1,188778763 | 0,001491912 |
| ALOX15B | 2,974475711 | 0,001710782 |
| GTF2B | -0,828177601 | 0,001719141 |
| CP | 2,648233815 | 0,002004922 |
| LAMP3 | 2,41017452 | 0,002204333 |
| CXCL9 | 2,169280102 | 0,002247933 |
| ITGAE | -1,206576279 | 0,002252932 |
| FOSB | 2,125678077 | 0,002442904 |
| CD68 | 1,4927073 | 0,002448192 |
| EPN2 | -1,15599587 | 0,002448686 |
| PDLIM5 | -1,155791792 | 0,002766379 |
| PLAUR | 1,529584703 | 0,002766379 |
| CD40LG | 2,782039086 | 0,00284002 |
| CA2 | 1,815244648 | 0,003037317 |
| IGSF6 | 2,620597958 | 0,003091141 |
| CCL22 | 2,530968216 | 0,003128092 |
| F2R | -1,414247236 | 0,003245262 |
| KIRREL1 | 0,987378169 | 0,003246478 |
| CD70 | 3,136259407 | 0,00327254 |
| TGFB3 | 1,506430276 | 0,003425484 |
| SLC2A6 | 1,740194486 | 0,003583971 |
| SPHK1 | 1,62256396 | 0,003632889 |
| ZBTB32 | 3,220873452 | 0,003819309 |
| GPR18 | 2,294821291 | 0,003909988 |
| SLC15A3 | 1,294465455 | 0,004039322 |
| ICAM1 | 1,357091526 | 0,004215638 |
| GCNT1 | 1,482660585 | 0,004280269 |
| KIF5A | 2,874747039 | 0,004398367 |
| MT2A | 1,432040357 | 0,004398367 |
| CDH12 | 2,83770899 | 0,004398367 |
| IL21 | 3,902914215 | 0,00461256 |
| LEPR | -1,54255579 | 0,004665443 |
| TNNI1 | 3,251519241 | 0,004675915 |
| RORA | -1,189608081 | 0,004701153 |
| DCN | -2,150004339 | 0,004937279 |
| CD5 | 2,364191567 | 0,005024599 |
| PLPP3 | -1,01312233 | 0,00510387 |
| PLEKHG3 | -1,355336745 | 0,0052654 |
| NDRG1 | 1,380192019 | 0,005323755 |
| PROCR | -1,433991899 | 0,005449248 |
| REV3L | -0,821140508 | 0,005455675 |
| SLC25A4 | -1,442418627 | 0,005464096 |
| EXT1 | 0,910273833 | 0,005725874 |
| SPIB | 2,707750773 | 0,005825686 |
| PTPN1 | 0,905421928 | 0,005970968 |
| PLPPR4 | 1,757245342 | 0,006019424 |
| NR3C1 | -0,846185036 | 0,006163013 |
| CD3G | 1,959696931 | 0,006392749 |
| EPCAM | 2,663588154 | 0,006426565 |
| S100A4 | -1,660032974 | 0,006671349 |
| HAX1 | -0,793172631 | 0,006671349 |
| LIME1 | 1,706299339 | 0,006688777 |
| CD40 | 1,198267936 | 0,006780877 |
| HIPK2 | 0,826838008 | 0,006987641 |
| SFRP1 | -1,812721595 | 0,00707199 |
| ITGA6 | -0,922765103 | 0,007262542 |
| CXCR4 | 1,152817204 | 0,007443529 |
| CD8A | 1,833134071 | 0,007480542 |
| MATN3 | 1,961418963 | 0,007879497 |
| DSC1 | 3,124850946 | 0,008008311 |
| CTH | 1,38691413 | 0,008151667 |
| JUNB | 1,096036521 | 0,008173251 |
| RPL3P7 | 4,295266961 | 0,008924108 |
| CHI3L1 | 2,045225267 | 0,009037287 |
| S100A1 | -2,184335214 | 0,009089224 |
| IFI30 | 1,453188113 | 0,009089224 |
| CD7 | 1,917852004 | 0,009175423 |
| MAP4K1 | 1,844822123 | 0,009195853 |
| EBI3 | 1,684941782 | 0,009638608 |
| CXCL3 | 2,007770646 | 0,009663804 |
| CD80 | 1,569866739 | 0,009696452 |
| ANKRD55 | 3,091314886 | 0,009711729 |
| PMEPA1 | 0,998811794 | 0,009792115 |
| ELL | 1,020277445 | 0,009956126 |
| CTSV | -2,290400746 | 0,010217542 |
| PTPN14 | -0,841527795 | 0,010300404 |
| FRMD4A | 1,060195735 | 0,010736283 |
| PTH1R | 1,820057225 | 0,010748577 |
| P4HA2 | 0,924133334 | 0,010880301 |
| ADCY6 | 1,083489897 | 0,011005774 |
| CCL4 | 1,674059317 | 0,011212201 |
| CXCL5 | 1,968393892 | 0,01160621 |
| CDK6 | 1,134813515 | 0,01160621 |
| MMP14 | 1,241793595 | 0,01160621 |
| SLC12A8 | 1,538985906 | 0,011706898 |
| CTHRC1 | 1,387560067 | 0,012114417 |
| TRIB3 | 1,233600221 | 0,012202753 |
| IGFBP3 | 1,104573925 | 0,012202753 |
| CD79A | 2,503847476 | 0,012245255 |
| ANPEP | 1,541638067 | 0,012728532 |
| CD83 | 1,051880468 | 0,012746885 |
| FN1 | 1,329471412 | 0,012966924 |
| MGAT1 | 0,665402704 | 0,013482393 |
| EEF1AKMT1 | -0,941625542 | 0,013842408 |
| SBNO2 | 1,056768263 | 0,014319103 |
| MEFV | 1,614521535 | 0,014706764 |
| PLOD3 | 0,687187491 | 0,014829063 |
| ETS1 | 0,76982251 | 0,014943066 |
| GREM1 | 2,155585645 | 0,01518917 |
| RRM2B | -0,697859609 | 0,015514469 |
| SYNJ2 | 1,02735378 | 0,015548918 |
| TPRKB | -0,782068813 | 0,015968094 |
| NT5C3A | -0,499727619 | 0,016362421 |
| MS4A3 | 3,555358872 | 0,016727189 |
| CCL19 | 2,239454922 | 0,017326546 |
| DCSTAMP | 2,295021917 | 0,017543784 |
| ARHGAP22 | 1,571227163 | 0,017543784 |
| NAALADL1 | 1,261355738 | 0,017705075 |
| DDIT3 | 1,287039639 | 0,018125955 |
| SAMSN1 | 1,409853047 | 0,018275032 |
| RGS1 | 1,534251758 | 0,018846661 |
| GALM | 0,868539177 | 0,018875807 |
| IGFBP2 | 1,55108597 | 0,018875807 |
| SLAMF1 | 2,09324712 | 0,018932825 |
| MRPL40 | -0,737925372 | 0,01970901 |
| CTLA4 | 2,237271062 | 0,020034428 |
| LDHC | 2,492980789 | 0,020529173 |
| PEX14 | 0,950650536 | 0,021055201 |
| CLU | 1,441620661 | 0,021246759 |
| CAP2 | -1,426520178 | 0,021626841 |
| RALGDS | 0,91840283 | 0,021809101 |
| SPOCK2 | 1,497187335 | 0,021844696 |
| ADAM12 | 1,163123227 | 0,02207967 |
| NPTXR | 1,490240991 | 0,022139141 |
| UGP2 | -0,9222945 | 0,022672805 |
| SOD2 | 1,919009283 | 0,022900607 |
| TMEM156 | 1,700174627 | 0,023584173 |
| FBLN5 | 1,177910741 | 0,024029462 |
| FAH | 1,148450727 | 0,024039836 |
| COL5A1 | 1,262348751 | 0,024077746 |
| GPX3 | 1,364447454 | 0,024565087 |
| SURF1 | -0,888554508 | 0,024799274 |
| P2RX4 | 0,888105948 | 0,024896205 |
| ST3GAL6 | -0,945354247 | 0,025515761 |
| PASK | 1,11954065 | 0,025684405 |
| ILVBL | 0,771769444 | 0,026411489 |
| PKP1 | 2,12232162 | 0,026489246 |
| DGAT1 | 0,725262023 | 0,026884391 |
| BGN | 1,24783178 | 0,027127296 |
| CLTB | -0,848666964 | 0,027204911 |
| CANT1 | 0,566782858 | 0,027567618 |
| GAA | 0,811151012 | 0,028077744 |
| DLG4 | 1,110305148 | 0,028361628 |
| ABCC1 | -0,814892898 | 0,028505655 |
| 1,00 AMD | -0,610728673 | 0,028505655 |
| BMF | 0,922721261 | 0,028619311 |
| MPO | 2,413390284 | 0,028630526 |
| POLD4 | 0,806276594 | 0,028738285 |
| ENO1 | 0,878005158 | 0,028770888 |
| VDAC2 | -0,682817436 | 0,029171034 |
| HCLS1 | 0,944592321 | 0,029281207 |
| CTSD | 1,070540478 | 0,029769531 |
| SCG2 | 1,707699399 | 0,029942195 |
| LOXL1 | 1,230513732 | 0,030046528 |
| BCL2A1 | 1,873963731 | 0,030268142 |
| BMP1 | 1,007744958 | 0,030419414 |
| ISCU | -0,562768919 | 0,03043441 |
| GTF2A2 | -0,72130443 | 0,031650121 |
| RAD51C | -0,797816354 | 0,03172271 |
| 1,00 DKK | 1,870056487 | 0,03222436 |
| PPP1R3C | -1,20149696 | 0,032470747 |
| SPTAN1 | -0,565809035 | 0,032506564 |
| TNFAIP3 | 1,148685618 | 0,032670684 |
| CDKN2AIP | -0,434965864 | 0,032921249 |
| IRF1 | 0,971995191 | 0,03329069 |
| CLCA2 | -2,239527296 | 0,034235314 |
| FYN | 0,671544778 | 0,034478385 |
| MGAM | 1,761714332 | 0,034707494 |
| TUBA4A | -1,144133115 | 0,034778789 |
| GPR183 | 1,296887287 | 0,034870354 |
| COQ8A | -0,889064432 | 0,035692992 |
| UPK3A | 2,466538122 | 0,036011898 |
| SLC1A5 | 0,785724213 | 0,036012312 |
| SPARC | 1,09662188 | 0,036300426 |
| OXSR1 | 0,453181251 | 0,03676546 |
| TAF1C | 0,860575716 | 0,036844123 |
| POLB | -0,546275821 | 0,036924758 |
| ITGA2 | -1,054628617 | 0,036924758 |
| PPP1R2 | -0,537048882 | 0,037152359 |
| CCR7 | 1,816377001 | 0,037839291 |
| WNT5A | 1,299078577 | 0,03800861 |
| IL2RB | 1,32528046 | 0,038143185 |
| CYP27B1 | 1,878038746 | 0,038451235 |
| PRG2 | 3,021893433 | 0,038471482 |
| ST3GAL4 | -0,829553601 | 0,038619566 |
| NDST2 | 0,882008015 | 0,038654587 |
| LTB | 2,211833792 | 0,038749305 |
| CD22 | 1,845959443 | 0,039132506 |
| SIPA1L1 | 0,605458319 | 0,039137775 |
| STC2 | 1,194795725 | 0,039605854 |
| EREG | 2,06271886 | 0,03971971 |
| MRPS31 | -0,503252545 | 0,039808148 |
| CXCL2 | 1,376756065 | 0,040007497 |
| REG1A | 3,773228238 | 0,040219364 |
| SDC1 | 1,293437191 | 0,040235298 |
| NIPBL | -0,51849053 | 0,040235298 |
| CR2 | 2,827580403 | 0,040412449 |
| P2RY10 | 1,497460709 | 0,040738892 |
| GAL | 2,136454311 | 0,040884323 |
| LHCGR | 2,293741361 | 0,040909005 |
| SERPINH1 | 0,793604845 | 0,041345897 |
| NEFH | 1,531565686 | 0,041542596 |
| ABAT | 1,251683876 | 0,042985791 |
| EOMES | 1,543776524 | 0,043208006 |
| MMP3 | 2,546007634 | 0,043210163 |
| HINT1 | -0,797516233 | 0,043443299 |
| TRPM4 | 1,144912659 | 0,043556144 |
| PTGER2 | 1,222654442 | 0,043972134 |
| RHBDF2 | 1,060382198 | 0,044312186 |
| GPR65 | 0,976546309 | 0,044829729 |
| MTA1 | 0,725208929 | 0,044833343 |
| SEC61A1 | 0,548280138 | 0,044898028 |
| DNAJB1 | 0,724333638 | 0,045202908 |
| TRBC1 | 1,537160303 | 0,045616697 |
| TOB1 | -0,704127004 | 0,047396941 |
| IL12RB1 | 1,453509552 | 0,047526554 |
| PMP22 | -0,948144709 | 0,048059464 |
| PTGDR | 1,414522452 | 0,048212896 |
| MAK | 1,330354869 | 0,048701525 |
| MAP3K1 | -0,647034654 | 0,049242212 |

**Table S4: Gene set enrichment analysis (GSEA) showing enrichment in pathways that were downregulated in tumor biopsies from patients who progressed compared to patients who showed stable disease or tumor response.**

| pathway | pval | padj | log2err | ES | NES | size | leadingEdge |
| --- | --- | --- | --- | --- | --- | --- | --- |
| HALLMARK_ADIPOGENESIS | 0.00891181988742964 | 0.0202001250781739 | 0.335347956526587 | -0.321408173244985 | -1.74906654992517 | 24 | PTGER3; ARL4A; ME1; SLC27A1; COQ3; LIFR; COX7B; ATP5PO; GPD2; SOWAHC; TOB1; HIBCH; ARAF; DGAT1; SLC1A5; ABCB8; ABCA1; PEX14; ADCY6; FAH; GPX3; C3; APOE |
| HALLMARK_ALLOGRAFT_REJECTION | 0.814281274748629 | 0.996919793847641 | 0.00219332500538299 | 0.252771829023555 | 0.813591369177941 | 44 | CXCL13; IL11; PRKCG; MMP9; FASLG; CD40LG; IGSF6; SIT1; CCL22; CD79A; CCL19; LTB; CXCL9; EREG; IL2RA; IL12A; HLA-DOA; CD3G; CD7; MAP4K1; CD8A; CCL4; CD80; FGR; GCNT1; CD74; IL12RB1; IL27RA; WAS; ICAM1; IL2RB |
| HALLMARK_APICAL_JUNCTION | 0.995965423680946 | 0.996919793847641 | 0.000514946030192017 | 0.134521305434599 | 0.417619210216036 | 32 | CLDN14; MADCAM1; CLDN6; MMP9; DSC1; ALOX15B; CLDN18; NRAP; COL9A1; NLGN3; CD274; CADM3; ADRA1B; FSCN1; ICAM1; MDK; EVL; SYK; CERCAM; BMP1; TMEM8B; SIRPA |
| HALLMARK_APOPTOSIS | 0.0488858572078217 | 0.0977717144156435 | 0.098399800470183 | -0.316231180084846 | -1.46786353114037 | 17 | DCN; TGFBR3; F2R; VDAC2; SPTAN1; BMF; IRF1; BGN; DDIT3; GPX3; CTH; CLU; NEFH; PLPPR4; SOD2; EREG |
| HALLMARK_BILE_ACID_METABOLISM | 0.90024454438066 | 0.996919793847641 | 0.00177303501712793 | 0.232231370223121 | 0.667033053396018 | 19 | HAO1; ABCG4; SERPINA6; SLC23A1; NR1I2; APOA1; CYP7A1; ABCA3 |
| HALLMARK_COAGULATION | 0.516000400761447 | 0.762783201125617 | 0.00442720779127663 | 0.307841642171854 | 0.995534720610749 | 46 | C8B; FGG; C8A; CPN1; CPB2; C9; FGA; ITIH1; CTSE; MMP10; PLG; PROC; MMP9; MMP8; F12; HPN; SERPINA1; MASP2; TMPRSS6; MMP3; APOA1; GP1BA |
| HALLMARK_COMPLEMENT | 0.920969874208797 | 0.996919793847641 | 0.00134933028402854 | 0.21832778702045 | 0.708881923573525 | 48 | MMP12; C9; ITIH1; PLG; MMP13; MMP8; ADRA2B; CR2; SERPINA1; CD40LG; CP; TMPRSS6; GATA3; SCG3; PCSK9; GP1BA; F5; GNGT2; APOC1; CA2; C1QC; CFB; OLR1; C1QA; C3; PLAUR; SPOCK2; CLU; WAS; FN1; MMP14; CEBPB; DOCK10; TNFAIP3; CTSD; CTSB; LTA4H; IRF1 |
| HALLMARK_DNA_REPAIR | 9.07573979096731e-07 | 1.54287576446444e-05 | 0.659444398037935 | -0.639588859416446 | -2.96880643271537 | 17 | POLH; SURF1; GTF2B; REV3L; MRPL40; GTF2A2; RRM2B; POLB; NT5C3A; SEC61A1; CANT1; POLD4; TAF1C; HCLS1; VPS37B; ELL; ADCY6 |
| HALLMARK_E2F_TARGETS | 0.000390500269128023 | 0.0016596261437941 | 0.49849310876659 | -0.481763925729443 | -2.23622381894009 | 17 | NAA38; SHMT1; BARD1; NUP205; DCK; RAD51C; DONSON; MSH2; ASF1A; ZW10; ING3; RAD50; JPT1; DCLRE1B; MYBL2; NUP107; ESPL1 |
| HALLMARK_EPITHELIAL_MESENCHYMAL_TRANSITION | 0.00360608492720661 | 0.00875763482321606 | 0.431707695803346 | -0.276735671021512 | -1.8707282252697 | 38 | DCN; SFRP1; TGFBR3; CAP2; ITGA2; PMP22; PLOD3; SERPINH1; PMEPA1; BMP1; SPARC; IGFBP3; COLGALT1; TNFAIP3; PLOD1; ADAM12; FBLN5; LOXL1; MMP14; BGN; COL5A1; QSOX1; SDC1; WNT5A; FN1; CTHRC1; PLAUR; ANPEP; IGFBP2; SCG2; DKK1; MATN3; SPP1; GREM1; SERPINE2 |
| HALLMARK_ESTROGEN_RESPONSE_EARLY | 0.991904974982712 | 0.996919793847641 | 0.000725271409442706 | 0.147456068467902 | 0.445223556488104 | 26 | KRT19; TJP3; KRT13; SOX3; MUC1; SYT12; KCNK15; ABCA3; TBC1D30; CALCR; SLC7A5; CYP26B1; ABAT; TGIF2; STC2 |
| HALLMARK_ESTROGEN_RESPONSE_LATE | 0.982578326669029 | 0.996919793847641 | 0.000793592313579855 | 0.165601390839438 | 0.507713765283014 | 29 | TH; KRT19; TJP3; KRT13; SOX3; SERPINA1; SERPINA5; ABCA3; PKP3; GAL; CA2; CALCR; SLC7A5; CYP26B1 |
| HALLMARK_FATTY_ACID_METABOLISM | 0.0883955600403633 | 0.166969391187353 | 0.0688229551583881 | -0.303281405369572 | -1.36696612507384 | 16 | ME1; ALDH3A2; GPD2; PTS; MDH1; SDHD; CBR1; ALDH9A1; HIBCH; ALAD; ACSS1; TDO2; CA2; ACSM3; IL4I1; HAO2 |
| HALLMARK_G2M_CHECKPOINT | 0.000666982300720779 | 0.00226773982245065 | 0.477270815362862 | -0.437956204379562 | -2.14171105728594 | 19 | MAP3K20; SLC12A2; BARD1; CUL5; AMD1; CUL4A; HSPA8; ORC5; ABL1; UPF1; JPT1; CCNF; MYBL2; RAD54L; MT2A; SLC7A5; E2F2; ESPL1; ATF5 |
| HALLMARK_GLYCOLYSIS | 8.7720773527937e-05 | 0.000497084383324977 | 0.538434096309916 | -0.365450084581156 | -2.24436259202187 | 32 | DCN; RARS1; COG2; ME1; AGL; UGP2; HAX1; SRD5A3; MDH1; ALDH9A1; HS2ST1; PHKA2; B4GALT1; ENO1; CHPF2; EXT1; P4HA2; PYGL; ERO1A; IGFBP3; CXCR4; PLOD1; IDUA; STC2; COL5A1; QSOX1; SDC1; CHPF; CTH; CHST1 |
| HALLMARK_HEME_METABOLISM | 0.104010577346849 | 0.18612419104173 | 0.0939002554813741 | -0.248168280825168 | -1.31227819591357 | 23 | GCLM; ABCG2; ENDOD1; NR3C1; NUDT4; CIR1; ARHGEF12; HEBP1; LRP10; KHNYN; ALAD; PIGQ; SLC66A2; P4HA2; CTSB; C3; E2F2; ACSL6; CA2; SMOX; KEL; SPTA1 |
| HALLMARK_HYPOXIA | 0.996919793847641 | 0.996919793847641 | 0.00044986971981105 | 0.130062501964199 | 0.4082010433222 | 35 | IGFBP1; EDN2; KIF5A; FBP1; CP; LDHC; PKP1; SLC2A5; PLAUR; TGFB3; MT2A; SLC2A3; NDRG1; DDIT3; COL5A1; BGN; STC2; CXCR4; TNFAIP3; IGFBP3; ERO1A; P4HA2; EXT1; NDST2; ENO1; GAA; ILVBL; ETS1 |
| HALLMARK_IL2_STAT5_SIGNALING | 0.0353327855382087 | 0.0750821692686935 | 0.221247181222857 | -0.254993342210386 | -1.50512238111971 | 29 | S100A1; ARL4A; ITGAE; RORA; EEF1AKMT1; ITGA6; ST3GAL4; SLC1A5; HIPK2; GALM; P2RX4; GPR65; CD83; FAH; PTGER2; IL2RB; NDRG1; SLC2A3; EOMES; CA2; PTH1R; CST7; IL2RA; SPP1; LTB; CTLA4; TNFRSF9; MUC1; TNFSF11 |
| HALLMARK_INFLAMMATORY_RESPONSE | 0.578558652706043 | 0.819624758000228 | 0.00391612575707247 | 0.303068519273939 | 0.960319600889964 | 38 | CSF3; ROS1; BDKRB1; CD70; HPN; CXCL8; SLC28A2; CCL22; TNFRSF9; LAMP3; GP1BA; CXCL9; SLAMF1; EREG; GPR132; NDP; CCR7; PDPN; OLR1; SLC1A2; EBI3; SPHK1; MEFV; CCRL2; RGS1; PLAUR; ICAM1; IL2RB; GPR183; MMP14; PTGER2; CD40 |
| HALLMARK_INTERFERON_GAMMA_RESPONSE | 0.000454693728437722 | 0.00171773186298695 | 0.49849310876659 | -0.331017872741847 | -2.03290178937549 | 32 | RTP4; ISOC1; CFH; PLA2G4A; ARL4A; MX1; LYSMD2; IFI44; PSMA3; PSMA2; ST8SIA4; PTPN1; IRF1; CCL2; IRF5; TNFAIP3; CIITA; FCGR1A; CD40; IL2RB; ICAM1; MT2A; IFI30; ITGB7; CD74; SLAMF7; CFB; CD274; SOD2; CXCL9; GPR18 |
| HALLMARK_KRAS_SIGNALING_DN | 0.210615165095762 | 0.358045780662795 | 0.00885949989224668 | 0.371460257598028 | 1.17702942944261 | 38 | CALML5; SPRR3; CKM; EDN2; NPHS1; HNF1A; SERPINA10; ABCG4; KRT4; KRT13; UPK3B; GPR3; SLC12A3; MYOT; CD40LG; CACNA1F; HTR1B; SIDT1; GP1BA; SLC38A3; CD207; PKP1; LYPD3; STAG3; HSD11B2; MEFV; CD80; IGFBP2; MACROH2A2 |
| HALLMARK_KRAS_SIGNALING_UP | 0.972959665588135 | 0.996919793847641 | 0.000825999525059636 | 0.180024905845366 | 0.570436831069909 | 38 | CFHR2; MMP10; ADAMDEC1; MMP9; SCG5; HKDC1; HOXD11; SCG3; SPP1; EREG; TMEM176B; TSPAN1; MAP4K1; CA2; CFB; MMP11; TMEM176A; EPHB2; PLAUR; ACE; CBX8; ADAM8; LAPTM5; CXCR4; TNFAIP3; IGFBP3; ITGB2; ERO1A |
| HALLMARK_MITOTIC_SPINDLE | 8.74922862673463e-06 | 7.43684433272443e-05 | 0.593325476396405 | -0.482884679295447 | -2.62780324314665 | 24 | PDLIM5; TUBA4A; MYH10; ARHGAP29; PALLD; UXT; PCNT; EPB41L2; RANBP9; SPTAN1; ARHGEF12; ABL1; RHOF; STAU1; PLEKHG2; MYO9B; CDC42EP1; CENPJ; SHROOM1; KIF3C; KPTN; FSCN1; PIF1; ESPL1 |
| HALLMARK_MTORC1_SIGNALING | 2.17306177572637e-06 | 2.46280334582322e-05 | 0.627256739718528 | -0.525249169435216 | -2.77744210573442 | 23 | TUBA4A; ME1; NUP205; PSMC2; PSMA3; PSMA4; UFM1; ACACA; SLC1A5; SERPINH1; CALR; ENO1; CCNF; LTA4H; ERO1A; ITGB2; CXCR4; TRIB3; DDIT3; CTH; SLC2A3; IFI30; SLC7A5 |
| HALLMARK_MYOGENESIS | 0.00158151985245705 | 0.0048883340894127 | 0.45505986738723 | -0.304131728736881 | -1.91566025410814 | 34 | MYH1; MYL2; CFD; FHL1; KLF5; CRYAB; COX7A1; NCAM1; PPP1R3C; PFKM |
| HALLMARK_OXIDATIVE_PHOSPHORYLATION | 3.69316604241518e-10 | 1.25567645442116e-08 | 0.814035837847908 | -0.732464302992348 | -3.58192641432793 | 19 | SLC25A4; NDUFB1; COX6C; COX7B; ATP5PO; SURF1; ACAT1; NDUFA4; NDUFV2; TIMM8B; MDH1; SDHD; VDAC2; NDUFB5; ETFA; ATP5F1C; SLC25A12; ISCU |
| HALLMARK_P53_PATHWAY | 0.001799959248526 | 0.00509988453749035 | 0.45505986738723 | -0.342591624779236 | -1.99960169326772 | 28 | CLCA2; KLF4; S100A4; PROCR; F2R; HSPA4L; POLH; COQ8A; PTPN14; RAD51C; HINT1; TPRKB; TOB1; ISCU; CDKN2AIP; SEC61A1; RALGDS; RHBDF2; CTSD; TRIB3; ABAT; DDIT3; SDC1; NDRG1; IFI30; SPHK1 |
| HALLMARK_PEROXISOME | 0.495288147879667 | 0.762783201125617 | 0.0272193881356601 | -0.221338634857522 | -0.967496006514951 | 15 | SLC25A4; ISOC1; PEX11A; FIS1; ALDH9A1; MSH2; IDE; PEX14; DLG4; HSD11B2; SOD2; ABCB9; NR1I2; HAO2; SERPINA6 |
| HALLMARK_SPERMATOGENESIS | 0.963065846832506 | 0.996919793847641 | 0.00129287878777204 | 0.194113356466821 | 0.552044616827545 | 18 | MTNR1A; MEP1B; TEKT2; SCG5; LDHC; SCG3; SLC2A5; NEFH; ACE; SEPTIN4 |
| HALLMARK_TNFA_SIGNALING_VIA_NFKB | 2.26455303954616e-05 | 0.000153989606689139 | 0.575610261071129 | -0.336483554725114 | -2.32247378532619 | 41 | KLF4; KLF2; F2RL1; PDLIM5; PLPP3; NFE2L2; B4GALT1; IER2; ABCA1; EHD1; IRF1; RCAN1; PMEPA1; SPSB1; CD83; CCL2; JUNB; TNFAIP3; CEBPB; GPR183; CLCF1; ICAM1; CXCL2; SLC2A3; NFKBIE; RELB; PLAUR; FOSL1; CD80; CCRL2; SPHK1; CCL4; OLR1; SLC2A6; BCL2A1; SOD2; MSC; CXCL3; FOSB |
| HALLMARK_UNFOLDED_PROTEIN_RESPONSE | 0.913943101477499 | 0.996919793847641 | 0.0018137210875538 | 0.232321555302362 | 0.63847905601806 | 15 | IGFBP1; CHAC1; SLC7A5; CEBPB; STC2; CCL2; ERO1A; CALR; EIF4EBP1; XPOT; TATDN2; DCTN1 |
| HALLMARK_UV_RESPONSE_DN | 0.000266437804935531 | 0.00129412648111543 | 0.49849310876659 | -0.478275290215589 | -2.28247434637043 | 18 | TGFBR3; CAP2; PDLIM5; PLPP3; PMP22; NR1D2; NR3C1; ABCC1; NIPBL; MRPS31; SIPA1L1; FYN; MTA1; PEX14; SYNJ2; FBLN5; GCNT1; PIK3CD |
| HALLMARK_UV_RESPONSE_UP | 0.00325646440388248 | 0.00851690690246188 | 0.431707695803346 | -0.341561102785734 | -1.80612601363701 | 23 | CTSV; SLC25A4; TUBA4A; CLTB; AMD1; PPP1R2; MGAT1; DNAJB1; DGAT1; IRF1; JUNB; DLG4; MMP14; ICAM1; GPX3; CXCL2; NPTXR; IGFBP2; CA2; SOD2; FOSB; GAL |
| HALLMARK_XENOBIOTIC_METABOLISM | 0.430934897690631 | 0.697704120070545 | 0.00526343416315213 | 0.327880954806856 | 1.04185242824362 | 39 | FETUB; IGFBP1; VTN; ITIH1; HGFAC; REG1A; PLG; GCKR; CES1; SERPINA6; DDC; FBP1; SLC22A1; PTGDS; CYP2S1; DDAH2; TMEM176B; HSD17B2; LEAP2; SMOX; CA2; CFB; APOE; CYP2E1; TDO2; PYCR1; ABCC3; MT2A |

Suppl Methods 1: Multivariate linear regression analysis: clinical variables influencing MLR score

Multivariate linear regression analysis: clinical variables influencing MLR score

*Methods*

To explore the relationship between the blood monocyte/lymphocyte ratio (MLR) and various clinical variables (e.g., age, gender, ECOG performance status, and radiotherapy parameters such as dose and isodose), a linear regression analysis was conducted. Due to the varying number of blood samples among patients, we used the average MLR scores for each patient, calculated separately for the periods before and after the irradiation date.

To satisfy the assumption of normality for the dependent variable required in linear regression, a logarithmic transformation of the MLR was applied. This transformation improved the distribution of the MLR, making it more closely approximate a normal distribution, as confirmed by the Shapiro-Wilk test. Other linear regression assumptions, such as homoscedasticity and the absence of multicollinearity, were also checked.

Initially, we constructed a comprehensive linear regression model including all potentially relevant clinical variables, with log(MLR) as the dependent variable. To achieve a more parsimonious model, a stepwise variable selection based on the Akaike Information Criterion (AIC) was employed. This method allowed us to identify the most influential variables for MLR, retaining only those that significantly enhanced the model fit according to the AIC criterion.

*Results*

A **multivariate linear regression analysis** was conducted to assess the influence of clinical variables on the MLR score. The analysis included **90 observations (MLR averages before and after radiotherapy)** from **49 patients**, of whom **45 received radiotherapy**. Given the limited sample size, results should be interpreted with caution.

The initial model (**Table 1**) showed an **adjusted R² of 0.0915** (**p = 0.053**), indicating that **9.15% of the variability** in the MLR score was explained by all included variables. After stepwise selection, the optimized model (**Table 2**) presented a slightly higher **adjusted R² of 0.1266** (**p = 0.006**), meaning that **12.66% of the MLR score variability** was explained by the retained variables.

**Only the ECOG performance status was significantly associated** with the MLR score (**p = 0.004 for ECOG 2 and p = 0.005 for ECOG 3**, compared to ECOG 1), while **other variables**, including those related to radiotherapy, **showed no significant association.**

Although these **low R² values** indicate that the models **explain only a limited portion** of the MLR score variability (**87.34% of the variability remaining unexplained in the optimized model**), the statistical significance of the performance status nevertheless **suggests a potential link** between the patient's general condition and this biomarker. These results highlight the **complexity of factors influencing** the MLR score and the need to explore other **potentially explanatory variables** not included in this analysis.

**Table 1 Linear regression model of log(Mean of MLR) with clinical variables before stepwise selection**

| **Variable** | **Estimate** | **Std. Error** | **t value** | **Pr(>\|t\|)** |
| --- | --- | --- | --- | --- |
| **(Intercept)** | 1.0615561 | 1.0787845 | 0.984 | 0.3281 |
| **Age** | 0.0023861 | 0.0062912 | 0.379 | 0.7055 |
| **Gender (Male vs Female)** | -0.0477170 | 0.1508162 | -0.316 | 0.7526 |
| **PS (ECOG = 2) (vs ECOG = 1)** | 0.4098154 | 0.1566023 | 2.617 | 0.0107* |
| **PS (ECOG = 3) (vs ECOG = 1)** | 0.9043642 | 0.3947699 | 2.291 | 0.0247* |
| **RT Timing (Before vs After)** | -0.2146617 | 0.1403433 | -1.530 | 0.1302 |
| **Dose** | -0.0094706 | 0.0113512 | -0.834 | 0.4066 |
| **Prescription (%) (to isodose)** | -0.0137768 | 0.0091486 | -1.506 | 0.1361 |
| **Number of beams, number of arcs** | -0.0009402 | 0.0021567 | -0.436 | 0.6641 |
| **PTV cc** | 0.0010130 | 0.0014579 | 0.695 | 0.4892 |

Notes:

- Dependent variable: log(Mean of MLR)
- *P < 0.05
- R² = 0.1855, Adjusted R² = 0.09147
- p-value = 0.05367

**Table 2 Optimized linear regression model of log(Mean of MLR) after stepwise selection**

| **Variable** | **Estimate** | **Std. Error** | **t value** | **Pr(>\|t\|)** |
| --- | --- | --- | --- | --- |
| **(Intercept)** | 0.978895 | 0.903254 | 1.084 | 0.28166 |
| **PS (ECOG = 2) (vs ECOG = 1)** | 0.432955 | 0.144823 | 2.990 | 0.00369** |
| **PS (ECOG = 3) (vs ECOG = 1)** | 0.993840 | 0.344760 | 2.883 | 0.00503** |
| **RT Timing (Before vs After)** | 0.214662 | 0.137600 | 1.560 | 0.12260 |
| **Dose** | -0.013690 | 0.009630 | -1.422 | 0.15893 |
| **Prescription (%) (to isodose)** | -0.012103 | 0.007722 | -1.567 | 0.12086 |

Notes:

- Dependent variable: log(Mean of MLR)
- **P < 0.01
- R² = 0.1768, Adjusted R² = 0.1266
- p-value = 0.006185

**Supplementary methods 2: Table of the top 20 genes from the MoMac clusters**

From Mulder, K. *et al.* Cross-tissue single-cell landscape of human monocytes and macrophages in health and disease. *Immunity* **54**, 1883–1900.e5 (2021).

| gene | cluster | p_val |  | p_val_adj | avg_logFC |
| --- | --- | --- | --- | --- | --- |
| LST1 | 1 | 0 |  | 0 | 1.5318446990769 |
| FCGR3A | 1 | 0 |  | 0 | 1.52133484975644 |
| IFITM2 | 1 | 0 |  | 0 | 1.47137121850356 |
| RHOC | 1 | 0 |  | 0 | 1.35961192076776 |
| CD52 | 1 | 0 |  | 0 | 1.32449805284263 |
| S100A4 | 1 | 0 |  | 0 | 1.1025158010331 |
| CDKN1C | 1 | 0 |  | 0 | 1.07109349242119 |
| IFITM3 | 1 | 0 |  | 0 | 1.06505842726049 |
| NAP1L1 | 1 | 0 |  | 0 | 1.05646091956748 |
| SERPINA1 | 1 | 0 |  | 0 | 1.03597974105744 |
| COTL1 | 1 | 0 |  | 0 | 1.01349073470749 |
| STXBP2 | 1 | 0 |  | 0 | 1.0120438649559 |
| LIMD2 | 1 | 0 |  | 0 | 1.00245518166332 |
| CD79B | 1 | 0 |  | 0 | 0.940232774876724 |
| ICAM3 | 1 | 0 |  | 0 | 0.900795347745315 |
| KLF2 | 1 | 0 |  | 0 | 0.885255115313424 |
| LTB | 1 | 0 |  | 0 | 0.881679270254375 |
| CORO1A | 1 | 0 |  | 0 | 0.868856226596445 |
| CD37 | 1 | 0 |  | 0 | 0.868747106991283 |
| POU2F2 | 1 | 0 |  | 0 | 0.864556824482441 |
| SLC40A1 | 2 | 0 |  | 0 | 1.28716664195542 |
| RNASE1 | 2 | 0 |  | 0 | 0.926894549009641 |
| LGMN | 2 | 0 |  | 0 | 0.900602426107437 |
| C1QA | 2 | 0 |  | 0 | 0.897506520428977 |
| C1QB | 2 | 0 |  | 0 | 0.834922591417669 |
| STAB1 | 2 | 0 |  | 0 | 0.81705629745735 |
| DAB2 | 2 | 0 |  | 0 | 0.807456313777782 |
| F13A1 | 2 | 0 |  | 0 | 0.793637187198959 |
| A2M | 2 | 0 |  | 0 | 0.77407110026494 |
| MAF | 2 | 0 |  | 0 | 0.773017370528374 |
| HES1 | 2 | 0 |  | 0 | 0.751056935923201 |
| FUCA1 | 2 | 0 |  | 0 | 0.740180351480661 |
| IGF1 | 2 | 0 |  | 0 | 0.734085319043604 |
| EGR1 | 2 | 0 |  | 0 | 0.733253697475972 |
| HSPA1A | 2 | 0 |  | 0 | 0.725025734781714 |
| C1QC | 2 | 0 |  | 0 | 0.711942725790145 |
| CCL3 | 2 | 0 |  | 0 | 0.691928943177155 |
| NR4A2 | 2 | 0 |  | 0 | 0.675105591684081 |
| CCL4 | 2 | 0 |  | 0 | 0.668523180474939 |
| CTSC | 2 | 0 |  | 0 | 0.667294983474607 |
| APOC1 | 3 | 0 |  | 0 | 1.10440064483456 |
| APOE | 3 | 0 |  | 0 | 1.02602113551782 |
| ACP5 | 3 | 0 |  | 0 | 0.881924836303141 |
| SPP1 | 3 | 0 |  | 0 | 0.86012614828256 |
| CTSD | 3 | 0 |  | 0 | 0.826177409123766 |
| GPNMB | 3 | 0 |  | 0 | 0.811833579413564 |
| FABP5 | 3 | 0 |  | 0 | 0.801342394766562 |
| LGALS3 | 3 | 0 |  | 0 | 0.774052770367081 |
| CTSB | 3 | 0 |  | 0 | 0.733096768763451 |
| PLA2G7 | 3 | 0 |  | 0 | 0.636336651352475 |
| CD63 | 3 | 0 |  | 0 | 0.614678718834523 |
| CD9 | 3 | 0 |  | 0 | 0.606951062496381 |
| PLD3 | 3 | 0 |  | 0 | 0.597475288123541 |
| LIPA | 3 | 0 |  | 0 | 0.593241395119637 |
| LGMN | 3 | 0 |  | 0 | 0.579269070716498 |
| TREM2 | 3 | 0 |  | 0 | 0.562086435689214 |
| MMP9 | 3 | 0 |  | 0 | 0.510902541724924 |
| SDS | 3 | 0 |  | 0 | 0.484100511297752 |
| C1QB | 3 | 0 |  | 0 | 0.483213589004217 |
| CCL18 | 3 | 0 |  | 0 | 0.480409946585179 |
| CXCL10 | 4 | 0 |  | 0 | 1.85735552517853 |
| ISG15 | 4 | 0 |  | 0 | 1.66882269474859 |
| IFIT1 | 4 | 0 |  | 0 | 1.38214865467022 |
| IFIT2 | 4 | 0 |  | 0 | 1.31472562839925 |
| IFIT3 | 4 | 0 |  | 0 | 1.2020067408931 |
| RSAD2 | 4 | 0 |  | 0 | 1.15677355829656 |
| TNFSF10 | 4 | 0 |  | 0 | 1.01793643916288 |
| ISG20 | 4 | 0 |  | 0 | 1.0150933929878 |
| IFI44L | 4 | 0 |  | 0 | 0.936043386679855 |
| IFITM3 | 4 | 0 |  | 0 | 0.872058532326733 |
| IFI6 | 4 | 0 |  | 0 | 0.838232930094802 |
| LY6E | 4 | 0 |  | 0 | 0.738067042654269 |
| EPSTI1 | 4 | 0 |  | 0 | 0.633210684305302 |
| GBP1 | 4 | 2.97335046648921e-299 |  | 3.13272205149303e-295 | 0.813215253166583 |
| IFI44 | 4 | 6.07834368579851e-281 |  | 6.40414290735731e-277 | 0.718295919685888 |
| PARP14 | 4 | 8.46812564713062e-277 |  | 8.92201718181682e-273 | 0.71351028739184 |
| TNFSF13B | 4 | 2.12152482946763e-261 |  | 2.2352385603271e-257 | 0.515681259774583 |
| IRF7 | 4 | 5.87223352115511e-257 |  | 6.18698523788902e-253 | 0.569737501177206 |
| IFI35 | 4 | 1.18282282395412e-255 |  | 1.24622212731806e-251 | 0.623002857747832 |
| APOBEC3A | 4 | 2.0177244541682e-255 |  | 2.12587448491162e-251 | 0.879624510460664 |
| CD52 | 5 | 0 |  | 0 | 0.589649985381878 |
| S100A4 | 5 | 0 |  | 0 | 0.494556622388314 |
| PLAC8 | 5 | 0 |  | 0 | 0.432876091645317 |
| S100A6 | 5 | 0 |  | 0 | 0.417093848233564 |
| CFP | 5 | 0 |  | 0 | 0.414284342293457 |
| LST1 | 5 | 0 |  | 0 | 0.39285114707664 |
| SERPINA1 | 5 | 0 |  | 0 | 0.368248079573332 |
| SH3BGRL3 | 5 | 0 |  | 0 | 0.340748732953239 |
| CORO1A | 5 | 0 |  | 0 | 0.333218624100762 |
| AP1S2 | 5 | 0 |  | 0 | 0.329205281805012 |
| LIMD2 | 5 | 0 |  | 0 | 0.327635468204736 |
| COTL1 | 5 | 0 |  | 0 | 0.319209212979249 |
| IFITM2 | 5 | 0 |  | 0 | 0.314771094227961 |
| STXBP2 | 5 | 0 |  | 0 | 0.310652471508446 |
| EMP3 | 5 | 0 |  | 0 | 0.298336900094363 |
| TSPO | 5 | 0 |  | 0 | 0.296869375342408 |
| IFITM3 | 5 | 0 |  | 0 | 0.291776559567736 |
| TXNIP | 5 | 0 |  | 0 | 0.277473541567619 |
| PRELID1 | 5 | 0 |  | 0 | 0.277383677796767 |
| RPL18A | 5 | 0 |  | 0 | 0.266050705957594 |
| CXCL10 | 6 | 0 |  | 0 | 2.14870584086474 |
| CXCL9 | 6 | 0 |  | 0 | 1.64967997654194 |
| GBP1 | 6 | 0 |  | 0 | 1.1469407386352 |
| IDO1 | 6 | 0 |  | 0 | 0.79417456159861 |
| STAT1 | 6 | 0 |  | 0 | 0.700435430144445 |
| IL4I1 | 6 | 0 |  | 0 | 0.693279006849693 |
| GBP5 | 6 | 0 |  | 0 | 0.687250592400941 |
| WARS | 6 | 0 |  | 0 | 0.667115737733975 |
| MMP9 | 6 | 0 |  | 0 | 0.642747218570518 |
| CD40 | 6 | 0 |  | 0 | 0.638280407543393 |
| PPA1 | 6 | 0 |  | 0 | 0.618068670602649 |
| SLAMF7 | 6 | 0 |  | 0 | 0.591100994786765 |
| ANKRD22 | 6 | 0 |  | 0 | 0.588062106193499 |
| SOD2 | 6 | 0 |  | 0 | 0.583231900606239 |
| VAMP5 | 6 | 0 |  | 0 | 0.571632100323912 |
| TAP1 | 6 | 0 |  | 0 | 0.565167848302016 |
| CCL8 | 6 | 0 |  | 0 | 0.553867622975208 |
| TXN | 6 | 0 |  | 0 | 0.549262259021698 |
| LAP3 | 6 | 0 |  | 0 | 0.535084522209708 |
| PSME2 | 6 | 0 |  | 0 | 0.526483340314646 |
| RGS1 | 7 | 0 |  | 0 | 0.572746613965835 |
| HLA-DPB1 | 7 | 0 |  | 0 | 0.543224030022006 |
| HLA-DRB1 | 7 | 0 |  | 0 | 0.535066194580743 |
| CD74 | 7 | 0 |  | 0 | 0.499566290783568 |
| HLA-DQA1 | 7 | 0 |  | 0 | 0.488175194904801 |
| HLA-DPA1 | 7 | 0 |  | 0 | 0.479187819426652 |
| HLA-DRA | 7 | 0 |  | 0 | 0.477304899229142 |
| HLA-DQB1 | 7 | 0 |  | 0 | 0.460575214736493 |
| CLEC10A | 7 | 0 |  | 0 | 0.449066681675752 |
| HES1 | 7 | 0 |  | 0 | 0.430817354677013 |
| HLA-DRB5 | 7 | 0 |  | 0 | 0.416903106689325 |
| HLA-DMA | 7 | 0 |  | 0 | 0.410624519036548 |
| GPR183 | 7 | 0 |  | 0 | 0.402668318863445 |
| ZNF331 | 7 | 0 |  | 0 | 0.396692153468338 |
| C1QC | 7 | 0 |  | 0 | 0.377964107726162 |
| DUSP2 | 7 | 0 |  | 0 | 0.351709368300539 |
| C1QA | 7 | 0 |  | 0 | 0.347482236769324 |
| HSPA1A | 7 | 0 |  | 0 | 0.337041013021239 |
| IL1B | 7 | 0 |  | 0 | 0.322456797773639 |
| CST3 | 7 | 0 |  | 0 | 0.322091763209447 |
| S100A8 | 8 | 0 |  | 0 | 2.68993700129629 |
| S100A9 | 8 | 0 |  | 0 | 2.35591821413858 |
| S100A12 | 8 | 0 |  | 0 | 2.06782032086531 |
| VCAN | 8 | 0 |  | 0 | 1.44401796553865 |
| S100A6 | 8 | 0 |  | 0 | 1.19364230552597 |
| MNDA | 8 | 0 |  | 0 | 1.18971965984266 |
| FOS | 8 | 0 |  | 0 | 1.17292954976049 |
| CSTA | 8 | 0 |  | 0 | 1.13111194076956 |
| S100A4 | 8 | 0 |  | 0 | 1.08183326519662 |
| LYZ | 8 | 0 |  | 0 | 1.00036078951205 |
| SELL | 8 | 0 |  | 0 | 0.989549423157614 |
| AP1S2 | 8 | 0 |  | 0 | 0.892865924799868 |
| RBP7 | 8 | 0 |  | 0 | 0.838927859954472 |
| MALAT1 | 8 | 0 |  | 0 | 0.810617785881381 |
| STXBP2 | 8 | 0 |  | 0 | 0.776476165932692 |
| RPL39 | 8 | 0 |  | 0 | 0.774926098127803 |
| CSF3R | 8 | 0 |  | 0 | 0.764543072398225 |
| H1FX | 8 | 0 |  | 0 | 0.748275197954888 |
| MGST1 | 8 | 0 |  | 0 | 0.720017948294833 |
| TSPO | 8 | 0 |  | 0 | 0.712056050104694 |
| CCL5 | 9 | 0 |  | 0 | 1.48273050921558 |
| IL32 | 9 | 0 |  | 0 | 1.45099785753882 |
| NKG7 | 9 | 0 |  | 0 | 1.26391202958587 |
| KLRB1 | 9 | 0 |  | 0 | 1.15972887893724 |
| CD3D | 9 | 0 |  | 0 | 1.10800015526034 |
| GZMA | 9 | 0 |  | 0 | 1.08700463128963 |
| CD7 | 9 | 0 |  | 0 | 0.942912224761931 |
| CD2 | 9 | 0 |  | 0 | 0.925433847268039 |
| CD3E | 9 | 0 |  | 0 | 0.868505577914483 |
| LTB | 9 | 0 |  | 0 | 0.825005168050922 |
| CST7 | 9 | 0 |  | 0 | 0.814542733822264 |
| CD69 | 9 | 0 |  | 0 | 0.768590479390803 |
| PRF1 | 9 | 0 |  | 0 | 0.701070623350183 |
| GZMM | 9 | 0 |  | 0 | 0.569750312313097 |
| IFITM1 | 9 | 0 |  | 0 | 0.439471652639198 |
| BTG1 | 9 | 0 |  | 0 | 0.366587377114639 |
| RPS27 | 9 | 0 |  | 0 | 0.333127236495975 |
| KLRG1 | 9 | 0 |  | 0 | 0.294045198077027 |
| CXCR4 | 9 | 1.72537967474261e-292 |  | 1.81786002530881e-288 | 0.448361525288421 |
| CD3G | 9 | 4.97471721849876e-288 |  | 5.2413620614103e-284 | 0.660232344950564 |
| STMN1 | 10 | 0 |  | 0 | 1.52589061990186 |
| HIST1H4C | 10 | 0 |  | 0 | 1.32057513571035 |
| TUBB | 10 | 0 |  | 0 | 1.29520874210408 |
| H2AFZ | 10 | 0 |  | 0 | 1.25018145721845 |
| UBE2C | 10 | 0 |  | 0 | 1.1675265889894 |
| TUBA1B | 10 | 0 |  | 0 | 1.09407110342258 |
| TOP2A | 10 | 0 |  | 0 | 1.04818382598233 |
| HMGB2 | 10 | 0 |  | 0 | 1.03319741649252 |
| TK1 | 10 | 0 |  | 0 | 1.02213602302111 |
| HMGN2 | 10 | 0 |  | 0 | 0.969218316504443 |
| PTTG1 | 10 | 0 |  | 0 | 0.956016995960646 |
| CDK1 | 10 | 0 |  | 0 | 0.93433534767333 |
| MKI67 | 10 | 0 |  | 0 | 0.914139985181612 |
| TYMS | 10 | 0 |  | 0 | 0.902193295014589 |
| HMGB1 | 10 | 0 |  | 0 | 0.889701630515451 |
| PCNA | 10 | 0 |  | 0 | 0.887566408633081 |
| RRM2 | 10 | 0 |  | 0 | 0.887156106719141 |
| BIRC5 | 10 | 0 |  | 0 | 0.811640159510046 |
| DUT | 10 | 0 |  | 0 | 0.79615920235143 |
| NUSAP1 | 10 | 0 |  | 0 | 0.794303182117352 |
| MT1G | 11 | 0 |  | 0 | 2.62612642477654 |
| MT1X | 11 | 0 |  | 0 | 2.21691995311464 |
| MT1E | 11 | 0 |  | 0 | 1.69682674999159 |
| MT2A | 11 | 0 |  | 0 | 1.62998792449477 |
| MT1F | 11 | 0 |  | 0 | 1.2512357674505 |
| FTL | 11 | 1.04061818599082e-288 |  | 1.09639532075992e-284 | 0.33517172826417 |
| MT1M | 11 | 1.29616182705176e-272 |  | 1.36563610098174e-268 | 1.34487240290888 |
| FUCA1 | 11 | 9.09781606954483e-197 |  | 9.58545901087243e-193 | 0.357638750162155 |
| CTSB | 11 | 4.25433785877558e-190 |  | 4.48237036800595e-186 | 0.417285486893875 |
| LGMN | 11 | 5.7009542213652e-185 |  | 6.00652536763038e-181 | 0.432725827609959 |
| CTSD | 11 | 8.85443502566584e-184 |  | 9.32903274304153e-180 | 0.462864320186819 |
| GLUL | 11 | 8.66693936007293e-183 |  | 9.13148730977284e-179 | 0.384851571191175 |
| APOC1 | 11 | 3.97623000449614e-148 |  | 4.18935593273713e-144 | 0.609725456984056 |
| GPNMB | 11 | 2.7573030657368e-133 |  | 2.90509451006029e-129 | 0.321919949836359 |
| SLC40A1 | 11 | 1.50414398106024e-132 |  | 1.58476609844507e-128 | 0.281805848306926 |
| SDS | 11 | 3.53048246594132e-113 |  | 3.71971632611578e-109 | 0.366184372189065 |
| LGALS3 | 11 | 4.00395836938466e-108 |  | 4.21857053798368e-104 | 0.328569577340824 |
| YWHAH | 11 | 8.82234509892504e-103 |  | 9.29522279622742e-99 | 0.289561282253897 |
| ABCA1 | 11 | 9.4060288751474e-100 |  | 9.91019202285531e-96 | 0.318277477098499 |
| IL6 | 11 | 7.55681682249267e-93 |  | 7.96186220417827e-89 | 0.298660535849389 |
| HSPA1B | 12 | 0 |  | 0 | 1.22066224966354 |
| HSPH1 | 12 | 0 |  | 0 | 1.21837887566676 |
| DNAJB1 | 12 | 0 |  | 0 | 1.12744861308227 |
| HSPA1A | 12 | 0 |  | 0 | 1.11115371016702 |
| HSP90AA1 | 12 | 0 |  | 0 | 0.840254522354249 |
| HSPE1 | 12 | 2.71106820232937e-282 |  | 2.85638145797423e-278 | 0.794405266722731 |
| HSPB1 | 12 | 3.45328158922581e-216 |  | 3.63837748240832e-212 | 0.839333757381825 |
| HSPD1 | 12 | 1.81033699338783e-209 |  | 1.90737105623342e-205 | 0.718726072184924 |
| BAG3 | 12 | 7.40496997329277e-204 |  | 7.80187636386127e-200 | 1.042339858915 |
| APOBEC3A | 12 | 7.62959128852524e-161 |  | 8.03853738159019e-157 | 0.517626428645795 |
| HSPA6 | 12 | 1.18651527574812e-159 |  | 1.25011249452822e-155 | 1.16975650709502 |
| BCL2A1 | 12 | 5.88369838344171e-113 |  | 6.19906461679418e-109 | 0.452784925034443 |
| RPL39 | 12 | 4.46095831070543e-108 |  | 4.70006567615924e-104 | 0.264004538137725 |
| ZFAND2A | 12 | 4.48944424681333e-85 |  | 4.73007845844252e-81 | 0.847393120542319 |
| SOD2 | 12 | 1.96906073032249e-79 |  | 2.07460238546778e-75 | 0.310954598882702 |
| IER5 | 12 | 8.68462864996575e-79 |  | 9.15012474560391e-75 | 0.688424265048201 |
| DNAJA1 | 12 | 1.08579939166276e-75 |  | 1.14399823905588e-71 | 0.44168523260897 |
| HSP90AB1 | 12 | 2.49882489835442e-74 |  | 2.63276191290622e-70 | 0.348370705003621 |
| NEAT1 | 12 | 6.76087994488474e-72 |  | 7.12326310993056e-68 | 0.297277355617116 |
| DNAJA4 | 12 | 1.57631526595766e-69 |  | 1.66080576421299e-65 | 0.881643172673858 |
| IGFBP7 | 13 | 2.1858268235685e-106 |  | 2.30298714131177e-102 | 1.02344729796569 |
| TIMP3 | 13 | 4.17061253005762e-103 |  | 4.39415736166871e-99 | 0.732697538145236 |
| CXCL14 | 13 | 4.51348816687502e-96 |  | 4.75541113261952e-92 | 0.987702457422788 |
| COL1A1 | 13 | 2.19832783993497e-95 |  | 2.31615821215548e-91 | 0.714491285650589 |
| CALD1 | 13 | 1.02383158743507e-91 |  | 1.07870896052159e-87 | 0.864690912955604 |
| COL3A1 | 13 | 1.04660234480305e-91 |  | 1.10270023048449e-87 | 0.778881444508227 |
| COL1A2 | 13 | 2.41552221462899e-91 |  | 2.54499420533311e-87 | 0.778572112315179 |
| IGFBP4 | 13 | 2.86931181933606e-87 |  | 3.02310693285247e-83 | 0.56260043081775 |
| RARRES2 | 13 | 8.59437024405551e-83 |  | 9.05502848913689e-79 | 0.375964435499437 |
| MYL9 | 13 | 1.51688263540361e-75 |  | 1.59818754466124e-71 | 0.641323053227197 |
| TM4SF1 | 13 | 1.84028065733358e-72 |  | 1.93891970056666e-68 | 0.674838554626767 |
| SPARC | 13 | 3.10948802272105e-71 |  | 3.2761565807389e-67 | 0.765480246715913 |
| DCN | 13 | 1.86820533418447e-70 |  | 1.96834114009676e-66 | 0.762345310951451 |
| DSTN | 13 | 6.05426968975145e-64 |  | 6.37877854512213e-60 | 0.380189114054557 |
| LMNA | 13 | 5.69891681626533e-63 |  | 6.00437875761715e-59 | 0.358308584428006 |
| MGP | 13 | 2.25891234044177e-61 |  | 2.37999004188945e-57 | 0.840951915481855 |
| CD59 | 13 | 2.65188248757217e-55 |  | 2.79402338890604e-51 | 0.381384624703975 |
| TPM2 | 13 | 2.05736318412162e-53 |  | 2.16763785079054e-49 | 0.504051086879815 |
| SPTBN1 | 13 | 2.4420033840382e-52 |  | 2.57289476542265e-48 | 0.487610172688378 |
| HSPB1 | 13 | 9.33222180420472e-52 |  | 9.83242889291009e-48 | 0.285369392120541 |
| FCER1A | 14 | 0 |  | 0 | 1.29492158139115 |
| CLEC10A | 14 | 0 |  | 0 | 1.15833814615982 |
| CD1C | 14 | 0 |  | 0 | 1.00166121312267 |
| CD1E | 14 | 0 |  | 0 | 0.855915054971146 |
| HLA-DQB1 | 14 | 0 |  | 0 | 0.777402408701058 |
| HLA-DPB1 | 14 | 0 |  | 0 | 0.763311104758521 |
| HLA-DQA1 | 14 | 0 |  | 0 | 0.714663535044314 |
| GPR183 | 14 | 0 |  | 0 | 0.665715776441298 |
| HLA-DRB5 | 14 | 0 |  | 0 | 0.621640543919049 |
| HLA-DRB1 | 14 | 0 |  | 0 | 0.600249444948845 |
| PPA1 | 14 | 0 |  | 0 | 0.590463200470238 |
| DUSP2 | 14 | 0 |  | 0 | 0.587182025020953 |
| PKIB | 14 | 0 |  | 0 | 0.574270456913914 |
| CD74 | 14 | 0 |  | 0 | 0.574040283621171 |
| HLA-DRA | 14 | 0 |  | 0 | 0.573874425515949 |
| CST7 | 14 | 0 |  | 0 | 0.551332968884238 |
| LGALS2 | 14 | 0 |  | 0 | 0.537791338709719 |
| HLA-DPA1 | 14 | 0 |  | 0 | 0.530195002740452 |
| CXCR4 | 14 | 0 |  | 0 | 0.521583466583563 |
| CST3 | 14 | 0 |  | 0 | 0.51172742838675 |
| G0S2 | 15 | 0 |  | 0 | 1.31020242453646 |
| CCL20 | 15 | 0 |  | 0 | 1.1910385867773 |
| IL1B | 15 | 0 |  | 0 | 1.16904820672328 |
| IL1RN | 15 | 0 |  | 0 | 1.13695230685045 |
| CXCL2 | 15 | 0 |  | 0 | 1.1013659864457 |
| SOD2 | 15 | 0 |  | 0 | 1.0396801562291 |
| EREG | 15 | 0 |  | 0 | 1.01648810096171 |
| CXCL3 | 15 | 0 |  | 0 | 0.992309561561953 |
| TIMP1 | 15 | 0 |  | 0 | 0.917604496966471 |
| BCL2A1 | 15 | 0 |  | 0 | 0.912034646583678 |
| NFKBIA | 15 | 0 |  | 0 | 0.887715804402701 |
| SERPINB9 | 15 | 0 |  | 0 | 0.788023670942446 |
| CCL3 | 15 | 0 |  | 0 | 0.78468306037243 |
| PTGS2 | 15 | 0 |  | 0 | 0.744456169900574 |
| PLAUR | 15 | 0 |  | 0 | 0.715187688351475 |
| TNIP3 | 15 | 0 |  | 0 | 0.698811968865208 |
| CCL4 | 15 | 0 |  | 0 | 0.66394530188435 |
| SLC2A3 | 15 | 0 |  | 0 | 0.65390488387477 |
| WTAP | 15 | 0 |  | 0 | 0.641589010950646 |
| SOCS3 | 15 | 0 |  | 0 | 0.635238471843877 |
| C1QC | 16 | 0 |  | 0 | 0.780906819886668 |
| C1QB | 16 | 0 |  | 0 | 0.687707695359676 |
| APOE | 16 | 0 |  | 0 | 0.66154948358185 |
| FUCA1 | 16 | 0 |  | 0 | 0.65109007357447 |
| C1QA | 16 | 0 |  | 0 | 0.648464903093498 |
| DNASE1L3 | 16 | 0 |  | 0 | 0.619535529559957 |
| HLA-DPA1 | 16 | 0 |  | 0 | 0.503859340292952 |
| ACP5 | 16 | 0 |  | 0 | 0.496337039692993 |
| RNASE1 | 16 | 0 |  | 0 | 0.49230833358981 |
| HLA-DRB1 | 16 | 0 |  | 0 | 0.484172107349738 |
| CD74 | 16 | 0 |  | 0 | 0.464089656729891 |
| HLA-DQA1 | 16 | 0 |  | 0 | 0.462756981044624 |
| PLD3 | 16 | 0 |  | 0 | 0.438274913220692 |
| CD63 | 16 | 0 |  | 0 | 0.417817027655561 |
| CTSC | 16 | 0 |  | 0 | 0.412671331699033 |
| HLA-DRB5 | 16 | 0 |  | 0 | 0.409297550809637 |
| HLA-DRA | 16 | 0 |  | 0 | 0.381091750569485 |
| TMEM176B | 16 | 0 |  | 0 | 0.378117885915225 |
| LIPA | 16 | 0 |  | 0 | 0.378023623503133 |
| PRDX1 | 16 | 0 |  | 0 | 0.371969384471764 |
| FTL | 17 | 0 |  | 0 | 0.472488838736686 |
| TMSB4X | 17 | 0 |  | 0 | 0.388668045994297 |
| HBB | 17 | 3.09738472520539e-305 |  | 3.2634045464764e-301 | 1.03999980297162 |
| PTMS | 17 | 2.04133765146747e-276 |  | 2.15075334958613e-272 | 0.254037693723747 |
| SKP1 | 17 | 3.63429353284661e-259 |  | 3.82909166620719e-255 | 0.251291500500941 |
| VSIG4 | 17 | 1.06298159575704e-251 |  | 1.11995740928962e-247 | 0.310301772379258 |
| CD81 | 17 | 1.51083029637173e-228 |  | 1.59181080025726e-224 | 0.260366822321503 |
| ARPC5 | 17 | 9.74567995032368e-219 |  | 1.0268048395661e-214 | 0.262190422178419 |
| PSME2 | 17 | 3.47544666769334e-212 |  | 3.6617306090817e-208 | 0.285222007008656 |
| NDUFA1 | 17 | 9.75823575856474e-208 |  | 1.02812771952238e-203 | 0.296937334369709 |
| GSN | 17 | 2.76913891177841e-184 |  | 2.91756475744974e-180 | 0.323188618794287 |
| MSR1 | 17 | 2.33079306620987e-183 |  | 2.45572357455872e-179 | 0.282945653605732 |
| EIF2S2 | 17 | 1.20844176952367e-179 |  | 1.27321424837014e-175 | 0.259753302309624 |
| ST13 | 17 | 5.78041339044216e-179 |  | 6.09024354816986e-175 | 0.313096737649458 |
| UQCR10 | 17 | 1.57675687354327e-178 |  | 1.66127104196519e-174 | 0.257641607392454 |
| CAPZA2 | 17 | 2.59033772541635e-175 |  | 2.72917982749866e-171 | 0.296871645781919 |
| PSMA7 | 17 | 4.18978991947435e-174 |  | 4.41436265915818e-170 | 0.259195617039982 |
| ANXA5 | 17 | 1.82892192379662e-170 |  | 1.92695213891211e-166 | 0.304656440310628 |
| PPDPF | 17 | 6.35753311061583e-170 |  | 6.69829688534484e-166 | 0.280684191441098 |
| LIPA | 17 | 2.57323901188232e-168 |  | 2.71116462291921e-164 | 0.27773575350831 |
