# Supplementary material for: Monocyte-lineage tumor infiltration predicts immunoradiotherapy response in advanced pretreated soft-tissue sarcoma: phase 2 trial results
Source: Signal Transduct Target Ther. 2025 Mar 17;10:103. doi: 10.1038/s41392-025-02173-3 (PMC11914280; doi:10.1038/s41392-025-02173-3)
Supplement: Supplementary file 2 — Trial Protocol [file 41392_2025_2173_MOESM2_ESM.pdf]

Sponsor Protocol N°: 2335/2015

EudraCT N°: 2015-005464-42 - EUCT N°: 2024-515678-29-00

**A phase II study to assess the efficacy of the anti-PD-L1 antibody atezolizumab (MPDL3280A) administered with stereotactic ablative radiotherapy (SABR) in patients with metastatic tumours**

Abbreviated Title of Protocol: "SABR-PDL1"

Phase II trial of the anti-PD-L1 antibody atezolizumab (MPDL3280A) combined with SABR

Version N°6.1 dated December 18th, 2023

**COORDINATING INVESTIGATOR**	**Pr Eric Deutsch**<br>Radiation therapy and drug developpement department<br>Gustave Roussy Cancer Campus<br>Tel.: +33 1 42 11 65 73<br>Villejuif, France<br>eric.deutsch@gustaveroussy.fr	**Signature of the Coordinating Investigator:**<br><br>Date **: December 18th, 2023**
**SPONSOR**	**GUSTAVE ROUSSY**<br>**114 RUE EDOUARD VAILLANT**<br>**94 805 VILLEJUIF**<br>**FRANCE**	**Signature of the Head of Clinical Research Department:**<br><br>Date : 18/12/2023

FOLLOW-UP OF VERSIONS

Version	Date	Description of substantial modifications
V1.4	10/06/2016	Initial version authorized by ANSM and CPP
V2.0	31/08/2016	- Addition of a secondary efficacy objective – Assessment of the efficacy using modified RECIST criteria.<br>- Addition of precision concerning the following inclusions criteria:<br>• The wash out period, which must be 4 weeks before C1D1<br>• Prior anticancer therapies required for each cohort<br>• Contraception methods<br>- Addition of precision in exclusion criterion regarding cases in which the use of inhaled corticosteroids is allowed.<br>- Addition of details regarding the scheduling of some examinations<br>- Addition of biological exams<br>- Addition of precisions regarding sequential tumour biopsies and blood sample collection<br>- IB V9 of atezolizumab<br>- Addition of precisions on Data Safety Monitoring Board section
V3.0	02/12/2016	- Modification of the inclusion criteria concerning the number of measurable metastatic sites required in order to increase patient recruitment
V4.0	18/06/2019	- IB V10, V11 and V14 of atezolizumab
V4.1	04/12/2019	- Closing of Renal cohort<br>- Addition of a sarcoma cohort and increase number of patients<br>- Increase of the inclusion duration<br>- Addition of the possibility of a second SABR irradiation<br>- Addition of two secondary objectives following the possible second SABR irradiation<br>- Modification of efficacy analysis
V5.0	17/08/2020	- Reduction of vital sign monitoring during infusion<br>- IB V15 of atezolizumab
V6.1	06/11/2023	- Addition of a proposal for an end of treatment, for patients who have received two years of treatment, and who have no active disease detected by PETSCAN FDG.<br>- Increase of the total duration of the study<br>- IB V18, V19 and V20 of atezolizumab

SIGNATURE PAGE

A phase II study to assess the efficacy of the anti-PD-L1 antibody atezolizumab (MPDL3280A) administered with stereotactic ablative radiotherapy in patients with metastatic tumours

EudraCT N°: 2015-005464-42 – EUCT N°: 2024-515678-29-00

CSET N°: 2335/2015

Version N°6.1 dated December 18th, 2023

Study Site:

I have read and approve this protocol. My signature confirms the agreement that the clinical study will be conducted in accordance with the protocol and all applicable laws and regulations including, but not limited to, European regulations, Guideline for Good Clinical Practice (GCP), the ethical principles that have their origins in the Declaration of Helsinki, applicable privacy laws and applicable study specific procedures, and that all persons assisting with the clinical study are adequately informed about the protocol, all subsequent amendments and the investigational products.

Nothing in this document is intended to limit the authority of a physician to provide emergency medical care under applicable regulations.

Investigator Signature : **Date of Signature (DD MM YYYY)**

Investigator Name and Title :

STUDY CONTACTS

	Name and Address	Telephone / Fax number
**Principal Investigator**	**Eric Deutsch, MD, PhD**<br>Gustave Roussy<br>Department of Radiotherapy	Tel : 01 42 11 42 11<br>Eric.deutsch@gustaveroussy.fr
**Sponsor**	**Gustave Roussy**<br>114 Rue Edouard Vaillant<br>F-94805 Villejuif Cedex	
**Statistician**	**Matthieu Texier**<br>Gustave-Roussy<br>Biostatistics and Epidemiology Unit	Tel: 01 42 11 62 52<br>matthieu.texier@gustaveroussy.fr
**Data manager**	**Nadia Zaghdoud**<br>Gustave-Roussy<br>Biostatistics and Epidemiology Unit	Fax: 01 42 11 62 54<br>nadia.zaghdoud@gustaveroussy.fr
**Pharmacovigilance**	**Dr Salim Laghouati, MD**<br>Gustave Roussy<br>Direction de la Recherche Clinique<br>Unité Fonctionnelle de Pharmacovigilance	Tel.: +33 1 42 11 61 00<br>Fax : +33 1 42 11 61 50<br>CTpharmacovigilance@gustaveroussy.fr
**Radiotherapy France**	**Antonin Levy, MD, PhD**<br>Gustave Roussy<br>Department of Radiotherapy	Tel. : 01 42 11 47 57<br>antonin.levy@gustaveroussy.fr
**Translational Part**	**Aurelien Marabelle, MD, PhD**<br>Gustave Roussy<br>Head of the clinical immunology programm	+33 1 42 11 43 85<br>Aurelien.marabelle@gustaveroussy.fr
**Sub PI Spain**	**Pr Jordi Giralt, MD**<br>Head, Medical Oncology Department<br>Vall d'Hebron University Hospital<br>08035 Barcelona SPAIN	Tel.: +34 93 -274 6085<br>jgiralt@vhebron.net
**Clinical Project Manager**	**Flora Ngadjeua Tchouatieu, PhD**<br>Gustave Roussy<br>Direction de la Recherche Clinique<br>Service Promotion des Etudes Cliniques	Tel.: +33 (0)1 42 11 62 50<br>Fax : +33 (0) 1 42 11 62 90<br>Flora.NGADJEUA-TCHOUATIEU@gustaveroussy.fr
**Sponsor Clinical Study Coordinator**	**Souad Cossé**<br>Gustave Roussy<br>Direction de la Recherche Clinique<br>Service Promotion des Etudes Cliniques	Tel : 01 42 11 62 48<br>Fax: 01 42 11 62 90<br>souad.cosse@gustaveroussy.fr

SYNOPSIS – PROTOCOL N° (2335/2015)

EudraCT number<br><br>EUCT number	2015-005464-42<br><br>2024-515678-29-00	Version and Date	6.1	**December 18th, 2023**		
Title	A phase II study to assess the efficacy of the anti-PD-L1 antibody atezolizumab (MPDL3280A) administered with stereotactic ablative radiotherapy (SABR) in patients with metastatic tumours					
Abbreviated title	SABR-PDL1	Phase	II			
Sponsor	Gustave Roussy<br>114 rue Edouard Vaillant<br>94805 Villejuif Cedex France					
Coordonnating Investigator	Pr Eric Deutsch					
Number of centers	Total	6	France	4	International : 2	Spain: 2
Indication	Patients with metastatic tumours (colorectal cancer, non-small lung cancer, renal cell carcinoma or sarcoma)					
Primary objective	To determine the 1 year progression-free survival rate under combined SABR and anti-PDL1 atezolizumab therapy using RECIST v 1.1. :<br><br>• in patients with metastatic colorectal cancer *(closed to inclusions)*<br>• in patients with metastatic non-small lung cancer *(closed to inclusions)*;<br>• *in patients with metastatic renal cell carcinoma (closed to inclusions);*<br>• in patients with metastatic sarcoma *(closed to inclusions)*					
Secondary Objectives	- To determine, the Progression-Free Survival (PFS) rate under combined SABR and anti-PDL1 atezolizumab therapy using RECIST v 1.1 and modified RECIST (mRECIST).<br><br>- To further describe, the efficacy of combination both on the irradiated lesions and the non-irradiated lesions based on tumour response indicators and clinical endpoints using RECIST v 1.1 and mRECIST<br><br>- To determine the Treatment failure rate defined as the proportion of patients who cannot receive the planned irradiation dose or relative atezolizumab dose intensity below 75% of the initially targeted dose (i.e. below 900 mg).<br><br>- To evaluate the toxicity of atezolizumab in combination with SABR according to					

	the NCI-CTCAE scale (version 4.03).<br><br>- To investigate the safety profile using NCI CTC-AE 4.03 scale following the second SABR course in combination with atezolizumab of tumor lesions after progression in patients with lung cancer and sarcoma.<br><br>- To assess the time to progression ratio after a second SABR course, i.e. the ratio of the TTP2 measured from the time of first progression to the time of progression after the second SABR course, over the TTP1 measured from the time of inclusion in the trial to the time of first progression, in patients with lung cancer and sarcoma who received a second SABR course.<br><br>- To assess the objective response rate at 6 weeks after the second SABR course, using RECIST v1.1, in the case of an oligoprogression and continuation of Atezolizumab at the time of progression in patients with lung cancer and sarcoma.<br><br>- To compare the modification of the size of the lesion which received the second SABR session versus the lesion that is not (abscopal effect) in patients with lung cancer and sarcoma.
Exploratory Objectives	- To evaluate the functional imaging changes using FDG PET/CT and tumour growth rates<br>- To evaluate the systemic immunologic anti-tumour response based on the sequential tumour biopsies and immunomonitoring on peripheral blood samples.
Methodology	Open-label, non-comparative, phase II clinical trial conducted in four cohorts of patients:<br><br>- *Cohort 1: metastatic colorectal cancer (closed to inclusions)*<br><br>- *Cohort 2: metastatic non-small lung cancer (closed to inclusions)*<br><br>- *Cohort 3: metastatic renal cell carcinoma (closed to inclusions)*<br><br>- *Cohort 4: metastatic sarcoma (closed to inclusions)*<br><br>In addition, cohorts 2 and 4 will also serve to address the impact of a second SABR course on possible oligoprogressive lesion(s), defined as ≤ 3 extracranial sites of progressive disease, and continuation of atezolizumab.<br><br>Patients will receive:<br><br>- atezolizumab at a dose of 1200 mg every 3 weeks, until disease progression or unacceptable toxicity, and<br><br>- stereotactic ablative radiation therapy, with the second cycle of atezolizumab (week 4) using 6MV photons or other energies with standard field encompassing tumour. Hypofractionated SABR will be delivered at a dose of 45 Gy in 3 fractions of 15 Gy (equivalent biologic dose (BED) > 80 Gy). The recommended fractionation is 45 Gy (3 fractions of 15 Gy) but shall be adapted upon normal tissue tolerance constraints. Total duration of radiotherapy must not exceed eight days and a window of ± 3 days allowed to start the treatment.<br><br>- in patients with lung cancer and sarcoma cancer who progress after SABR +

	atezolizumab treatment, a second SABR treatment, while continuing atezolizumab, with the same conditions will be allowed.<br><br>After treatment discontinuation, patients will enter in follow-up period or have an end of study visit as described in section3.2.
Inclusion Criteria	1. Patients must be 18 years of age or older.<br><br>2. Histologically or cytologically proven metastatic solid tumours including:<br><br>- *colorectal (CRC, Microsatellite instability negative and positive) in treatment failure as per the current standard recommendation (cohort closed to inclusions).*<br><br>- *non-small cell lung cancer (NSCLC) pretreated by at least one line of treatment. Patients EGFR-mutant can be included only if they have been treated with, or developed toxicity with or refused to be treated with anti-EGFR therapy; Patients pretreated by anti−PD1, or anti−PDL1 therapeutic antibodies can be included only if they have received at least 4 months of treatment (cohort closed to inclusions).*<br><br>- *renal cell carcinoma (RCC) pretreated by at least one line therapy by a tyrosin kinase inhibitor (cohort closed to inclusions).*<br><br>- *metastatic sarcomas of any type (soft tissue, bone, GISTs) pretreated by at least one line of standard therapy ; at least three lines of standard TKi must be given in patients with GISTs. No enrolment restriction to certain sarcoma subtypes/groups was decided given the relative rarity of this disease type and that immunotherapy efficacy in certain histological subtypes is only preliminary (cohort closed to inclusions).*<br><br>3. Patients with at least :<br>- one measurable metastasis by RECIST 1.1 eligible for SABR in terms of dose constraints at organ at risk (refer to Appendix 1: Rules for SABR administration according to tumour location ; distinct criteria apply regarding lung and liver metastases) and ≤ 4 cm, **and**<br>- one not treated measurable metastasis by RECIST 1.1. If all tumour sites are accessible to SABR, one of them will not be treated.<br><br>Metastase located within the proximal bronchial tree as defined in RTOG 0236 (refer to Appendix 1: Rules for SABR administration according to tumour location) or within the brain are not eligible for SABR treatment in the present study. However, it can be considered as a not treated evaluable metastase.<br><br>4. WHO performance status of 0-1<br><br>5. Evaluation by a radiation oncologist within 45 days prior to study registration, including imaging workup to document metastases (cf. description in assessment section)<br><br>6. Patients must have adequate organ function defined by the following laboratory results obtained within 28 days prior to the first study treatment:<br><br>- Absolute neutrophil count of ≥ 1500/mm3;<br><br>- Lymphocyte count ≥ 500 mm3;<br><br>- Platelets ≥ 100,000/mm3;<br><br>- Hemoglobin > 9 gr/dL;<br><br>- Clearance Creatinine ≥ 50 mL/min;

	- Total bilirubin ≤ 1.5X ULN (unless Gilbert where 3X ULN is permitted);<br>- Serum ALT and AST ≤ 2.5X ULN (unless documented liver metastases where ≤ 5X ULN is permitted),<br>- ALK ≤ 2.5 ULN (unless documented bone or liver metastases where ≤ 5X ULN is permitted).<br>7. Life expectancy of more than 3 months<br>8. Patients must be aware of the investigational nature of the therapy and provide written informed consent.<br>9. Sexually active women of childbearing potential must agree to use a highly effective method of contraception supplemented with a barrier method, or to abstain from sexual activity during the study and for at least 5 months after the last dose of atezolizumab<br>Sexually active males patients must agree to use condom while on SABR treatment and for at least 90 days after SABR treatment. Taking into account the irradiated area, use of condom after SABR treatment can be shortened at investigator discretion. Also, their women of childbearing potential partner should use a highly effective method of contraception.<br>Women who are not postmenopausal (≥ 12 months of non-therapy-induced amenorrhea) or surgically sterile must have a negative serum β-HCG pregnancy test result within 7 days prior to initiation of study drug.<br>A list of highly effective birth control methods and the definition of a woman of childbearing potential are provided in the core protocol (section 4.1).<br>10. Patients must be free of significant comorbid conditions that would preclude safe administration or completion of protocol therapy.<br>11. The irradiated and unirradiated tumour sites must be accessible to tumour biopsy (additional written consent required).<br>12. Patients must be affiliated to a social security system
Exclusion Criteria	1. Known allergy to anti-PD-L1 including :<br>- History of severe allergic anaphylactic reactions to chimeric, human or humanized antibodies, or fusion proteins.<br>- Known hypersensitivity to CHO cell products or any component of the atezolizumab formulation.<br>2. Pregnant or breastfeeding women<br>3. Any malignancy other than the disease under study in the past 5 years excepting skin cancers such as BCC or SCC.<br>4. Uncontrolled tumour-related pain<br>Patients requiring pain medication must be on a stable regimen at study entry.<br>Asymptomatic metastatic lesions whose further growth would likely cause functional deficits or intractable pain (e.g., epidural metastasis that is not presently associated with spinal cord compression) should be considered for loco-regional therapy if appropriate prior to enrolment.<br>5. Uncontrolled pleural effusion, pericardial effusion, or ascites requiring recurrent drainage procedures (once monthly or more frequently).<br>Patients with indwelling catheters (e.g., PleurX) are allowed.

	6. Uncontrolled hypercalcemia (> 1.5 mmol/L ionized calcium or Ca > 12 mg/dL or corrected serum calcium > ULN) or symptomatic hypercalcemia requiring continued use of bisphosphonate therapy or denosumab.<br><br>Patients who are receiving bisphosphonate therapy or denosumab specifically to prevent skeletal events and who do not have a history of clinically significant hypercalcemia are eligible. However, patients who are receiving denosumab prior to enrollment must be eligible to receive bisphosphonate instead and willing to switch to bisphosphonate therapy while on the study.<br><br>7. Severe, active co-morbidity, defined as follows:<br>- Unstable angina and/or congestive heart failure requiring hospitalization within the last 6 months prior to registration;<br>- Transmural myocardial infarction within the last 6 months prior to registration;<br>- Acute bacterial or fungal infection requiring intravenous antibiotics at the time of registration;<br>- Uncontrolled Chronic Obstructive Pulmonary Disease or other respiratory illness requiring hospitalization or precluding study therapy within 30 days prior to registration<br>- History of idiopathic pulmonary fibrosis, organizing pneumonia (e.g., bronchiolitis obliterans), drug-induced pneumonitis, idiopathic pneumonitis, or evidence of active pneumonitis on screening chest CT scan<br>History of radiation pneumonitis in the radiation field (fibrosis) is permitted.<br>- Severe hepatic disease, defined as a diagnosis of Child-Pugh Class B or C hepatic disease.<br>- Known HIV positive status.<br>- End-stage renal disease (i.e., on dialysis or dialysis has been recommended).<br>- Patients with active hepatitis B (defined as having a positive hepatitis B surface antigen [HBsAg] test at screening) or hepatitis C.<br>Patients with past hepatitis B virus (HBV) infection or resolved HBV infection (defined as having a negative HBsAg test and a positive antibody to hepatitis B core antigen [anti-HBc] antibody test) are eligible<br>Patients positive for hepatitis C virus (HCV) antibody are eligible only if polymerase chain reaction (PCR) is negative for HCV RNA.<br><br>8. Active or History of autoimmune or inflammatory disease, including but not limited to myasthenia gravis, myositis, autoimmune hepatitis, systemic lupus erythematosus, rheumatoid arthritis, inflammatory bowel disease, vascular thrombosis associated with anti-phospholipid syndrome, Wegener's granulomatosis, Sjögren's syndrome, Guillain-Barré syndrome, multiple sclerosis, vasculitis, or glomerulonephritis (see Appendix 3 for a more comprehensive list of autoimmune diseases)<br><br>Patients with a history of autoimmune hypothyroidism on a stable dose of thyroid replacement hormone are eligible<br><br>Patients with controlled Type 1 diabetes mellitus on a stable insulin regimen

are eligible

Patients with vitiligo or psoriasis or grave's disease, not requiring systemic treatment within the last 2 years, are eligible

9. Metastases located to the brain and with clinical signs and/or leptomingeal carcinomatosis, or with indistinct borders making targeting not feasible

   Metastases located to the brain and without clinical signs can be included.

10. Irradiation required for cord compression and for superior veina cava syndrome.

11. Irradiation by SABR should not include metastases located within 3 cm of the previously irradiated structures:

    - Spinal cord previously irradiated to > 40 Gy
    - Brachial plexus previously irradiated to > 50 Gy
    - Small intestine, large intestine, or stomach previously irradiated to > 45 Gy
    - Brainstem previously irradiated to > 50 Gy
    - Lung previously irradiated with prior V20Gy > 30%

12. Metastasis localized to the central part of the chest and requiring irradiation (see "no fly zone" in Appendix 1: Rules for SABR administration according to tumour location).

13. Any approved anticancer therapy, including chemotherapy, hormonal therapy or radiotherapy, under the following guidelines:

    - investigational or cytotoxic treatments within 4 weeks prior to the study treatment initiation and while on study treatment
    - localized palliative radiotherapy within 2 weeks prior to the study treatment initiation and while on study treatment
    - any approved TKIs within 3 weeks prior to the study treatment initiation and while on study treatment

however Hormone-replacement therapy or oral contraceptives are allowed

14. Administration of a live, attenuated vaccine within 4 weeks prior to Cycle 1, Day 1 or anticipation that such a live attenuated vaccine will be required during the study.

15. Influenza vaccination should be given during influenza season only (example: approximately October to March in the Northern Hemisphere). Patients must not receive live, attenuated influenza vaccine (e.g., FluMist®) within 4 weeks prior to Cycle 1, Day 1 or at any time during the study

16. Treatment with systemic corticosteroids or other systemic immunosuppressive medications (including but not limited to prednisone, dexamethasone, cyclophosphamide, azathioprine, methotrexate, thalidomide, and anti−tumour necrosis factor [TNF] agents) within 2 weeks prior to Cycle 1, Day 1, or anticipated requirement for systemic immunosuppressive medications during the trial

    Patients who have received acute, low-dose, systemic immunosuppressant medications (e.g., a one-time dose of dexamethasone for nausea) may be enrolled in the study

    The use of inhaled corticosteroids for chronic obstructive pulmonary disease,

	mineralocorticoids (e.g., fludrocortisone) for patients with orthostatic hypotension, and low-dose supplemental corticosteroids for adrenocortical insufficiency are allowed.<br>17. Patient already enrolled in another therapeutic trial involving an investigational substance, and when such a substance has been taken during the previous 4 weeks.<br>18. Persons deprived of their freedom or under guardianship, or for whom it would be impossible to undergo the medical follow-up required by the trial, for geographic, social or psychological reasons<br>19. Prior treatment with CD137 agonists or immune checkpoint blockade therapies, anti−PD1, or anti−PDL1 therapeutic antibodies<br>Only patients with non-small cell lung cancer are allowed to have received anti−PD1, or anti−PDL1 therapeutic antibodies. Subjects who have received prior anti-PD-1/L1 therapies must have received at least 4 months of treatment.<br>Patients who have received prior treatment with anti−CTLA-4 may be enrolled, provided at least 5 half-lives (approximately 75 days) have elapsed from the last dose of anti-CTLA-4 to the first dose of atezolizumab and there was no history of severe immune-mediated adverse effects from anti−CTLA-4 (NCI CTCAE Grade 3 and 4)<br>20. Treatment with systemic immunostimulatory agents (including but not limited to interferon-alpha (IFN-α) and interleukin-2 (IL-2) within 4 weeks or five half-lives of the drug (whichever is shorter) prior to Cycle 1, Day 1
Study Treatment	**Drug:** The anti-PD-L1 antibody atezolizumab will be administered at a dose of 1200 mg every 3 weeks.<br>Atezolizumab treatment will be administered as long as:<br>- Patients are experiencing clinical benefit (as assessed by an investigator), and<br>- In the absence of unacceptable toxicity or symptomatic deterioration attributed to disease progression after an integrated assessment of radiographic data, biopsy results (if available) and clinical status.<br>In case of progressive disease according to RECIST v1.1 criteria, patients will be permitted to continue atezolizumab treatment if they meet all of the following criteria:<br>- Evidence of clinical benefit as assessed by the Investigator,<br>- Absence of symptoms and signs (including worsening of laboratory values, e.g., new or worsening hypercalcemia) indicating unequivocal progression of disease,<br>- No decline in ECOG performance status that can be attributed to disease progression, and<br>- Absence of tumour progression at critical anatomical sites (e.g., leptomeningeal disease) that cannot be managed by protocol-allowed medical interventions.<br>**SABR:** SABR will be delivered concurrently with the 2nd cycle of atezolizumab (at week 4) using 6MV photons or other energies with standard field encompassing tumour. If oligoprogression occurs, a second course of SABR is allowed while continuing atezolizumab (patient with lung cancer and sarcoma).<br>At least one tumour site will receive radiation and another tumour site(s) should be

	evaluable by RECIST v1.1.<br><br>Hypofractionated SABR will be delivered at a dose of 45 Gy in 3 fractions of 15 Gy (equivalent biologic dose (BED) > 80 Gy). The recommended fractionation is 45 Gy (3 fractions of 15 Gy) but shall be adapted upon normal tissue tolerance constraints. Total duration of radiotherapy must not exceed eight days. Tumour size will be ≤ 4 cm according to recent imaging (CT or PET).
Primary evaluation criterion	One year-Progression-free survival rate is defined as the rate of patients alive and free of progression at one year. Progression is defined using RECIST 1.1. criteria, or death, whatever the cause of death, whichever occurs first.
Secondary evaluation criteria	• **Efficacy:**<br><br>Progression-free survival (PFS) measured from the date of treatment initiation to date of progression or death whichever comes first. Patients alive and free of progression at the cut-off date will be censored at the last assessment date<br><br>Tumour response indicators and clinical endpoints, assessed according to RECIST v 1.1 and modified RECIST are:<br><br>- Disease Control Rate (DCR): complete responses (CR) + partial responses (PR) + stable disease (SD).<br>- Objective Response Rate (ORR): CR + PR<br>- Duration of response (DOR)<br>- Time to progression of non-irradiated lesions is computed as the delay from enrollment to the first occurrence of progression outside of the irradiated field<br>- Time to progression of irradiated lesions is computed as the delay from enrollment to the first occurrence of progression of irradiated lesions;<br>- Evaluation of the local control, distant control.<br><br>• **Toxicity** : NCI- CTCAE V4.03 scale Toxicity is adverse events graded with the NCI-CTC-AE v4.03 scale, at least possibly related to the treatment.<br><br>**For patients with lung cancer and sarcoma receiving a second SABR course for oligoprogression**:<br><br>- Progression-free survival 2 (PFS2) measured from the date of second SABR course to date of second progression or death whichever comes first. Patients alive and free of progression at the cut-off date will be censored at the last assessment date.<br>- Tumor response indicators and clinical endpoints, assessed according to RECIST v1.1 at 6 weeks after the second SABR course.<br><br>Time to progression ratio (TTPr) in patient receiving a second SABR course:<br><br>- To define the TTP ratio, let's denote:<br>○ TTP1 the time from treatment initiation to the time of progression after first irradiation<br>○ TTP2 the time from first progression to the time of progression after the second SABR course. Due to the very poor prognosis of those patients, death without documented progression will be counted as progressions. Patients alive and free of progression at the cut-off date will be censored at the last assessment date. Radiological progression is measured using RECIST v1.1 on the same tumor lesions over all the

	trial, whenever possible.<br>Treatment failure rate defined as the proportion of patients who cannot receive the planned irradiation dose or relative atezolizumab dose intensity below 75% of the initially targeted dose (i.e below 900 mg).	
Sample size determination	This is an open-label non-comparative phase II trial with four cohorts of patients.<br>One-year progression-free survival rate will be estimated in CRC (cohort 1), in NSCLC (cohort 2) and in RCC (cohort 3) and in sarcoma (cohort 4). In each cohort, a Fleming 1-stage design will be applied to demonstrate that the progression free survival rate at one year is not inferior to 15% but could reach 32%.<br>To ensure a global alpha risk (Type I error) will be fixed at 0.1, one-sided the Bonferroni correction will applied; Cohort 3 is stopped for lack of accrual and no test will be performed. Three tests will be carried out and the alpha risk for each test will be 0.033.<br>To test in each cohort, the hypothesis that the PFS rate is greater than p0 with alpha=0.033 and with a 90% power to detect activity greater than p1, we need to enroll 54 evaluable patients.<br>If 13 patients or more are alive and free of progression at one year in the cohort, the combined SABR + atezolizumab will be considered as a success for the corresponding histology.<br>To account for possibly 10% of non evaluable patients, 60 patients will be enrolled per cohort (RCC excluded). Considering that the RCC cohort will be closed after 7 patients, the overall expected sample size will then be 187 evaluable patients (60 patients with NSCLC, 7 with RCC, and 60 with CRC and 60 with sarcoma).<br>Patients with lung cancer and sarcoma progressing after their initial treatment with SABR + atezolizumab will be enrolled for a second SABR course while continuing atezolizumab. Assuming that up to 50% of patients may have oligoprogression (i.e. being eligible for a second SABR course) after the investigational immune checkpoint blockers, between 25 and 30 patients per cohort are expected to receive a second SABR course. These cohorts size will enable to detect a response rate at 6 weeks of p1=30% versus a response rate below p0=10% at the 10% type I error level (one-sided) and with a 90% power.	
Number of patients	Total: 138	France and Spain
Duration of the trial	Inclusion	3 years
	Treatment	Until disease progression or unacceptable toxicity<br>(estimation: 2 years)<br>In patients who have not progressed after 2 years of treatment, it is appropriate to consider and discuss treatment discontinuation, which will be proposed if active disease is not detected on PET scan.
	Follow-up	Until disease progression or up to maximum 1 year
	Duration of the study	8 years

Table 1: Investigation summary

Week	Enrolment*	1	2	3	4	5	6	7	8	9	10	11	12	13	16	19	Every 3 weeks	At first PD (cohorts 2&4)	W 6 post second SABR course only (cohorts 2&4)	Every 12 weeks until treatment discontinuation	FU period**	
																					End of Study visit	FU visit
Inclusion / exclusion criteria	X*																					
Informed consent form signed	X																					
Registration	X																					
Physical examination, assessment of safety and toxicities eg ECOG and vital signs	X	X			X	X	X	X	X	X	X			X	X	X	X				X	X
Electrocardiogram	X																					
	**Treatments**																					
Atezolizumab 1200 mg (Cycle #) + 3 days allowed		C1 X			C2 X			C3 X			C4 X			C5 X	C6 X	C7 X	Until disease progression or unacceptable toxicity					

Week	Enrolment*	1	2	3	4	5	6	7	8	9	10	11	12	13	16	19	Every 3 weeks	At first PD (cohorts 2&4)	W 6 post second SABR course only (cohorts 2&4)	Every 12 weeks until treatment discontinuation	FU period**	
																					End of Study visit	FU visit
SABR 45 Gy in 3 fractions[5] (on alternate days)					X													X[4]				
**Imaging**																						
FDG-PET **(optional part)**	X				X[1]			X[1]						X[1]						X[1]		
Chest and abdominal CT scan (± 7 days except at baseline)	X (within 2 weeks before registration at most)				X[1]			X[1]						X[1]					X[1]	X[1]		X
Brain CT scan / Brain MRI scan	X[2, 3]				X[1, 3]			X[1, 3]						X[1, 3]						X[1, 3]		X[3]
Liver MRI[3], Spinal MRI[3]	X[3]				X[1, 3]			X[1, 3]						X[1, 3]						X[1, 3]		X[3]
**Laboratory Evaluations**																						
Biological assessment<br><br>(refer to section 6.1 for baseline assessment,	X	X			X			X			X			X	X	X	X					

Week	Enrolment*	1	2	3	4	5	6	7	8	9	10	11	12	13	16	19	Every 3 weeks	At first PD (cohorts 2&4)	W 6 post second SABR course only (cohorts 2&4)	Every 12 weeks until treatment discontinuation	FU period**	
																					End of Study visit	FU visit
and then section 6.2)																						
Pregnancy test (serum) (for women of childbearing potential)	X (within 7 days prior to C1D1)				X			X			X			X	X	X	X					
	**Translational Research (optional part)**																					
Tumour biopsy***	X				X(-8)[1]			X(-8)[1]														
Serum sampling for banquing	X				X[1]			X[1]						X[1]						X[1]		
Blood sampling for immune-monitoring **(only for patients included at Gustave Roussy)**	X				X[1]			X[1]						X[1]						X[1]		

SABR : Stereotactic Ablative Radiotherapy

1 To be performed before atezolizumab administration

2 If clinical signs suggesting brain metastases (performed within 2 weeks before registration at most), or if known brain metastases

3 According to the investigator judgment

4 In case of first progression in patient with lung cancer and sarcoma. A chest and abdominal CT scan has to be performed 6 weeks after the second SABR course.

5 With a window of ± 3 days allowed for the beginning of the stereotaxy. The full treatment should not last more than 8 days long

* within 28 days before treatment no specific treatments will be allowed (washout period); the maximum duration for the screening period is 28 days.

** Patients who discontinue atezolizumab for disease progression will have an end of study visit 1 month after treatment discontinuation.

Patients who discontinue atezolizumab for other reason than disease progression will have a follow-up visit every 3 months up to 12 months or disease progression according to RECIST 1.1, whichever occurs first.

*** Archival tumour sample, biopsy at baseline (before Atezolizumab treatment), at week 4 or up to 8 days before C2D1 (i.e before start of SABR), at week 7 or up to 8 days before C3D1 (after atezolizumab and SABR combination).Only tissue from core needle, punch or excisional biopsy sample collection will be accepted. For core-needle biopsy specimens, three cores should be submitted for evaluation. Fine-needle aspiration, brushing, bone tissue, and lavage samples are not acceptable

ABREVIATIONS

AE	Adverse event
AESI	Adverse event of special interest
ALK	Alkaline phosphatase
ALT	Alanine transaminase
aPTT	Activated Partial Thromboplastin Time
AST	Aspartate aminotransferase
BCC	Basal Cell Carcinoma
BED	equivalent biologic dose
CBC	Complete Blood Count
CHO	Chinese hamster ovary
CL	Clearance
CR	Complete response
CRA	Clinical Research Assistant
CRC	Colorectal cancer
CRF	Case Report Form
CRP	C-Reactive Protein
CT	Computed tomography
DCR	Disease Control Rate
DOR	Duration of response
EC	Ethic committee
ECG	Electrocardiogram
ECOG	Eastern Cooperative Oncology Group
FDG	[18F]-fluorodeoxyglucose
GI	Gastrointestinal
GR	Gustave Roussy
HBV	Hepatitis B virus
HCG	Hormon chorionic gonadotropin
HCV	Hepatitis C virus
HIV	Human immunodeficiency virus
IB	Investigational Brochure
ICF	Informed consent form
ICH	International conference of harmonization

IFN-α	interferon-α
Ig	Immunoglobulin
IL-2	interleukin-2
IMP	Investigational Medicinal Product
INR	International Normalized Ratio
IR	Ionizing Irradiation
IrAE	Immune-related adverse event
IRR	Infusion Related Reaction
IUD	Intrauterine Device
IUS	intrauterine hormone-releasing system
IV	Intravenous
LFTs	Liver Function Tests
MDSC	myeloid-derived suppression cell
MedDRA	Medical dictionary for regulatory activities
mg	milligram
mM	milliMol
mRECIST	modified RECIST
MRI	Magnetic resonance imaging
NCI CTCAE	NCI Common terminology Criteria for Adverse Events
NSCLC	Non-small cell lung cancer
ORR	Objective response rate
PBMC	Peripheral Blood Mononuclear Cell
PCR	Polymerase Chain Reaction
PD	Progression Disease
PD-1	Programmed cell death 1
PD-L1	Programmed cell death ligand 1
PES	polyethersulfone
PET	Positron emission tomography
PFS	Progression-free survival
PK	Pharmacokinetic
PO	polyolefin
PR	Partial response
PTV	planned tumour volume

PVC	polyvinyl chloride
RBC	Red Blood Cell Count
RCC	renal cell carcinoma
RECIST	Response Evaluation Criteria In Solid Tumors
RNA	Ribonucleic acid
SABR	Stereotactic Ablative Radiotherap
SAE	Serious adverse event
SADR	Serious adverse drug reaction
SCC	Squamous cell carcinoma
SUSAR	Suspected Unexpected serious adverse reaction
T4	thyroxine
TNF	Tumor Necrosis Factor
TSH	thyroid stimulating hormone
ULN	Upper limit of normal
VEGFR	Vascular Endothelial Growth Factor Receptor
WB	Whole Blood
WBC	Whole Blood Count
WHO	World Health Organization

TABLE OF CONTENTS

1 INTRODUCTION AND RATIONAL OF THE STUDY 25

2 STUDY OBJECTIVES 29

2.1 Primary objective 29

2.2 Secondary objectives 29

3 METHODOLOGY 30

3.1 Treatment period 30

3.2 Follow-up period 31

3.3 Flowchart: 32

4 SELECTION OF PATIENTS 32

4.1 Inclusion criteria 32

4.2 Exclusion criteria 34

4.3 Withdrawal criteria from study treatment and/or from the study 37

5 TREATMENTS 38

5.1 Identification of the study treatments 38

5.1.1 Atezolizumab 38

5.1.2 Stereotactic Ablative Radiation Therapy (SABR) 40

5.2 Dosage and schedule of treatment 41

5.2.1 Atezolizumab 41

5.2.2 SABR 42

5.3 Adaptation of doses and interruption of treatment 43

5.3.1 Doses modifications for SABR 43

5.3.2 Management of atezolizumab-specific adverse events 43

5.4 Concomitant medications 74

5.4.1 Permitted Therapy 74

5.4.2 Prohibited Therapy 74

6 PLAN OF THE STUDY ... 75

6.1 Baseline assessments ... 75

6.2 During the treatment period ... 77

6.3 During the follow-up period ... 78

6.4 Exploratory objectives (optional part of the study): ... 78

6.4.1 Functional imaging changes using FDG PET/CT ... 78

6.4.2 Systemic immunologic anti-tumour response ... 79

7 EVALUATION CRITERIA ... 81

7.1 Primary criterion ... 81

7.2 Secondary criteria ... 81

7.2.1 Efficacy ... 81

7.2.2 Safety ... 82

8 DETERMINATION OF SAMPLE SIZE AND STATISTICAL ANALYSIS ... 82

8.1 Sample size ... 82

8.2 Stopping rule ... 83

8.3 Data Analysis ... 83

8.3.1 General Considerations ... 83

8.3.2 Efficacy Analysis ... 83

8.3.3 Safety Analysis ... 85

9 SERIOUS ADVERSE EVENTS ... 85

9.1 Definition ... 85

9.1.1 Adverse Event (AE) ... 85

9.1.2 Serious Adverse Event (SAE) ... 85

9.1.3 Expected Serious Adverse Event ... 86

9.1.4 Unexpected Serious Adverse Event ... 87

9.1.5 Intensity criteria ... 87

9.2 Reporting of Serious Adverse Events (SAE), Adverse Events of Special interest (AESI) and pregnancy 87

9.2.1 Reporting of Serious Adverse Events (SAE) 87

9.2.2 Reporting of (Non-Serious) Adverse Events of Special Interest (Study Specific AESI) 88

9.2.3 Reporting of exposure to study drug during pregnancy/lactation. 89

9.3 Responsibilities of the coordinating Sponsor 89

9.4 Data Safety Monitoring Board (DSMB) 90

10 STUDY DISCONTINUATION 91

11 ETHICAL AND REGULATORY ASPECTS 91

11.1 Rules and regulations 91

11.2 Committee for the Protection of Persons (CPP) – Competent Authority 92

11.3 Information and Consent of Participants 92

11.4 Principal Investigator Responsibilities 92

12 DATA MANAGEMENT METHODS 93

12.1 Registration of patients 93

12.2 Data collection 93

13 QUALITY ASSURANCE - MONITORING 94

13.1 Monitoring 94

14 DATA OWNERSHIP / PUBLICATION POLICY 94

15 DATA PROTECTION 95

15.1 Confidentiality 95

15.2 Investigator's personal data 95

16 REFERENCES 96

17 APPENDIX 1: RULES FOR SABR ADMINISTRATION ACCORDING TO TUMOUR LOCATION 99

18 APPENDIX 2 – NATIONAL CANCER INSTITUTE - COMMON TERMINOLOGY CRITERIA FOR ADVERSE EVENTS 104

19 APPENDIX 3: LIST OF AUTOIMMUNE DISEASES: 105

20 APPENDIX 4: RECIST 1.1 CRITERIA 109

21 APPENDIX 5: MRECIST CRITERIA MODIFIED RESPONSE EVALUATION CRITERIA IN SOLID TUMORS 113

1 INTRODUCTION AND RATIONAL OF THE STUDY

The development of human cancer is a multistep process characterized by the accumulation of genetic and epigenetic alterations that drive or reflect tumour progression. These changes distinguish cancer cells from their normal counterparts, allowing tumours to be recognized as foreign by the immune system. However, tumours are rarely rejected spontaneously, reflecting their ability to maintain an immunosuppressive microenvironment. Programmed death-ligand 1 (PD-L1; also called B7-H1 or CD274), which is expressed on many cancer and immune cells, plays an important part in blocking the 'cancer immunity cycle' by binding programmed death-1 (PD-1) and B7.1 (CD80), both of which are negative regulators of T-lymphocyte activation. Binding of PD-L1 to its receptors suppresses T-cell migration, proliferation and secretion of cytotoxic mediators, and restricts tumour cell killing. The PD-L1–PD-1 axis protects the host from overactive T-effector cells not only in cancer but also during microbial infections. Blocking PD-L1 therefore enhances anticancer immunity [Chen].

Atezolizumab treatment

Atezolizumab is a human monoclonal antibody based on a human IgG1 framework containing heavy chain V_HIII and light chain VκI subgroup sequences. The recombinant antibody consists of two heavy chains (448 amino acid residues each) and two light chains (214 amino acid residues each) with inter- and intra-chain disulfide bonds that are typical of IgG1 antibodies. Atezolizumab incorporates an amino acid substitution (asparagine to alanine) at position 298 in the CH2 domain of each heavy chain resulting in a non-glycosylated antibody that has minimal binding to Fcγ receptors and, consequently, prevents Fc-effector function and depletion of cells expressing PD-L1 at expected concentrations in humans. Therefore, atezolizumab lacks the N-linked oligosaccharides typically observed on other CHO-derived monoclonal antibodies.

<u>Clinical Pharmacokinetics</u>

On the basis of available preliminary PK data (0.03−20 mg/kg), atezolizumab appeared to show linear pharmacokinetics at doses ≥ 1 mg/kg. For the 1-mg/kg and 20-mg/kg dose groups, the mean apparent clearance (CL) and the mean volume of distribution at steady state (Vss) had a range of 3.20 to 4.44 mL/day/kg and 48.1 to 65.7 mL/kg, respectively, which is consistent with the expected PK profile of an IgG1 antibody in humans.

<u>Clinical efficacy and safety</u>

The high-affinity human monoclonal immunoglobulin-G1 (IgG1) antibody atezolizumab that specifically binds to PD-L1 and prevents its interaction with PD-1 and B7.1 was recently assessed in a dose-escalating phase I trial [Herbst]. A total of 277 patients with advanced incurable cancer received atezolizumab intravenously every 3 weeks. Mean single-dose atezolizumab pharmacokinetics were consistent with a typical IgG1 at doses ≥1 mg kg−1, with a mean terminal serum half-life of ~3 weeks. Overall, treatment was well tolerated up to the maximum administered dose of 20 mg kg−1 q3w. Most adverse events (AEs) did not require medical treatment. The most common treatment-related AE was fatigue, which often occurred with low-grade fever during the first treatment cycle. Pyrexia was reported in ~21% of patients; it most commonly occurred during cycle 1 and was uncommon during subsequent cycles. Treatment-related grade 3–4 AEs were observed in 35 patients (13%) and immune-related grade 3-4 AEs were observed in 3 patients (1%). According per Response Evaluation Criteria in Solid Tumours, version 1.1 (RECIST v1.1) on 175 evaluable patients; confirmed responses (complete and partial responses) were observed in 32 of 175 (18%), 11 of 53 (21%), 11 of 43 (26%), 7 of 56 (13%) and 3 of 23 (13%) of patients with all tumour types, non-small cell lung

cancer (NSCLC), melanoma, renal cell carcinoma and other tumours (including colorectal cancer, gastric cancer, and head and neck squamous cell carcinoma), respectively. Responses could also be rapid and durable. Also, the association of tumour PD-L1 expression correlated with clinical response to atezolizumab.

Further information on this study (named study PCD4989g in the IB) and from other studies conducted with atezolizumab in monotherapy or in combination are presented in the investigator brochure.

Therapy of sarcomas represents an area of significant unmet need in oncology. Sarcoma arises from cells of mesenchymal origin with many different subtypes. Immunotherapy response is dependent on complex interactions between tumor and immune cells within the tumor microenvironment. Several factors determine whether or not the immunotherapy response will be promoted or inhibited. These include the inherent antigenicity of the tumor. Mutations in proteins and/or aberrant proteins expressed by tumor cells and the “neoantigens” they generate are the primary targets for T-cell-mediated destruction. Tumor mutation burden has emerged as a quantitative marker which can help to predict responses to immune checkpoint inhibition. The immunotherapy response is also dependent on the infiltration of immune effector cells into the tumor. Specific patterns of tumor infiltrating lymphocytes (TILs) within the tumor microenvironment are associated with improved outcome in patients with many types of cancers, regardless of the type of therapy administered.

A naturally-occurring immune infiltrate has been reported for several sarcoma subtypes. Orui et al., provided a detailed characterization of the immune infiltrates in a variety of histologic subtypes with detection of cytotoxic CD8 T cells expressing granzyme B, indicative of their cytotoxic function, the presence of dendritic cells found to be CD1a negative but CD83 positive, indicative of a mature phenotype [Orui]. Tseng et al. reported evidence suggesting an adaptive immune response with the presence of intratumoral lymphoid structures [Tseng]. In liposarcoma, CD8 T cells appear to be scattered throughout the tumor, whereas CD4 T cells and CD20 B cells were localized to these lymphoid structures. The presence of such immune infiltrate suggests that immunotherapy may potentially be effective in this subtype.

The currently available human data to support the efficacy of immunotherapy in sarcomas remain limited. An intratumoral infiltration of PD-1 positive lymphocytes was observed in 65% of cases and PDL1 tumor expression in 58% across several soft tissue sarcoma subtypes (N=105, with leiomyosarcoma (n = 20), synovial sarcoma (n = 16), undifferentiated sarcoma (n = 11), and myxoid liposarcoma (n = 10) [Kim]. The phase II study SARC028 demonstrated a RR of 40 % and 20% for undifferentiated pleomorphic sarcoma and dedifferentiated liposarcoma, respectively; under pembrolizumab treatment. Two (5%) of 40 patients with bone sarcoma had an objective response, including one (5%) of 22 patients with osteosarcoma and one (20%) of five patients with chondrosarcoma. In the STS cohort, 20 % ORR was reported by RECIST 1.1 with additional 40 % of patients with best response of stable disease. Four-month progression-free rate was 44 % (C.I 22-66 %) which was considered as statistically significant improvement relative to historical PFR of 20 %. [Tawbi, Burgess]. Moreover, durable responses have been seen with atezolizumab phase 2 study in advanced alveolar soft part sarcoma. The objective response rate (ORR) was 42 % with confirmed PRs in 8 out of 19 patients [O’Sullivan].

Despite the small sample size and the clinical heterogeneity of sarcoma cohort analyzed, Sharma et al., [Sharma] reported that RT induced remarkable changes in various immune cells, immune-related transcripts and cancer-testis antigens, including: a higher infiltration of both CD4+ and CD8+ T cells after radiotherapy; an upregulated expression of MHC-I and of different CT-antigens, which makes tumors more visible to CD8+ T cells; a *de novo* expression of the highly immunogenic CT-antigens in some patients ; a higher expression of immune effector molecules, including perforin, granzyme B, NKG2D, IFN-γ, TNF-α, and IL-12. Of note,

recently, a complete clinical response was observed in patients with clear cell sarcoma treated with an anti-PD1 checkpoint inhibitor combined with standard fractionation radiotherapy [Marcrom].

Also, the mechanism of action of atezolizumab is associated with inflammation and/or immune-mediated adverse events including potential dermatologic, hepatic, endocrine, gastrointestinal and respiratory events. Such events should be closely monitored. Management guidelines are provided in this protocol (section 5.3.2).

Rational of the study

Although it is usually described as an immunosuppressive modality and not thought of as immunotherapy, there are new preclinical evidences suggesting that high-dose ionizing irradiation (IR) results in direct tumour cell death and augments tumour-specific immunity, which enhances tumour control both locally and distantly. Importantly, IR effects exceed the classical cytocidal properties by also causing phenotypic changes in the fraction of surviving cells, markedly enhancing their susceptibility to T cell-mediated elimination [Formenti]. However, not all IR-induced modifications of the tumour and its microenvironment favor immune rejection. The tumour microenvironment is populated by various types of inhibitory immune cells including Tregs, alternatively activated macrophages, and myeloid-derived suppression cells (MDSCs), which suppress T cell activation and promote tumour outgrowth. Chiang et al. showed the accumulation of pro-tumourigenic M2 macrophages in areas of hypoxia present in irradiated tumours [Chiang]. IR then may also induced responses that are inadequate to maintain antitumuor immunity.

Close interaction between IR, T cells, and the PD-L1/PD-1 axis exsit and provide a basis for the rational design of combination therapy with immune modulators and radiotherapy. Deng et al. demonstrate that PD-L1 was upregulated in the tumour microenvironment after IR. Moreover, administration of anti-PD-L1 enhanced the efficacy of IR through a cytotoxic T cell-dependent mechanism. Concomitant with IR-mediated tumour regression, IR and anti-PD-L1 synergistically reduced the local accumulation of tumour-infiltrating MDSCs, which suppress T cells and alter the tumour immune microenvironment. Finally, activation of cytotoxic T cells with combination therapy mediated the reduction of MDSCs in tumours through the cytotoxic actions of TNF [Sagiv-Barfi]. Sagiv-Barfi et al, also demonstrated in 5 patients receiving atezolizumab and radiation therapy, at least stabilization of systemic progression in all patients and a RECIST partial response at systemic sites in 1 patient. Transient, grade 1-2 inflammatory adverse events (fevers, flu-like symptoms) occurred with no serious immune-related toxicities [Sagiv-Barfi]. Abscopal out-field effects of irradiation has also been described in addition to a reduction in circulating MDSCs in a melanoma patient treated with the anti CTLA-4 ipilimumab and radiotherapy [Postow].

Lastly, recent evidence demonstrates that loco-regional curative treatment with stereotactic ablative radiotherapy (SABR) is a good alternative as compared with conventional 3D RT for patients with solid tumour, with durable remissions and a low toxicity profile. Many non-randomised studies have shown that SBRT for oligometastases is safe and effective, with local control rates of about 80%. Importantly, these studies also suggest that the natural history of the disease is changing, with 2–5 year progression-free survival of about 20% [Tree]. For colorectal, non-small cell, and renal cell cancers, 1-year metastasis control rates ranged from 67 to 91% [Salama]. Moreover, abscopal reponses in the setting of immune checkpoints inhibitors and radiotherapy combinations have been made in the setting of metastatic disease event in patients with extensive tumor burden [Postow], [Golden], [Reynders]. The goal of SABR is to deliver appropriate metastasis directed radiotherapy while minimizing exposure of surrounding normal tissues. Interestingly, the dose and fractionation employed modulate RT

ability to synergize with immunotherapy. Vanpouille-Box et al, showed that immune response genes were differentially expressed in irradiated tumours by 8Gyx3 but not 20Gyx1. This highlight the interest of hypofractionated SABR acting as a "*in situ* tumour vaccine" [Vanpouille-Box].

As hypofractionated SABR may, in addition to its good local control, increase the effectiveness of anti PD-L1, we aimed to investigate the efficacy and the tolerability of the combination of anti-PD-L1 antibody with SABR. Preliminary clinical data about the combination of Atezolizumab and radiotherapy in metastatic patients suggest that irradiation of one tumor site may induce disease stabilization which may last several Atezolizumab administration cycles (ie 3-6 months). These early data might reflect the fact that the systemic anti tumor response is transient. Thus, we plan to test whether focal re irradiation of a previously un irradiated tumor site might re induce systemic anti tumor response in the case of disease re evolution for patients progressing after initial stabilization of their disease. These data are in line with one of the first prospective clinical trial aimed at evaluating abscopal effect which performed radiation therapy to 2 distinct tumor sites separated from several weeks [Golden].

Shaverdian et al. showed that radiotherapy, given before PD-1/PD-L1 inhibition, could increase PFS and OS over PD-1/PD-L1 inhibition alone [Shaverdian]. This study included 98 patients from the Keynote-001 trial that tested PD-1 inhibition (pembrolizumab) in progressive stage III or stage IV NSCLC. Very interestingly, median PFS was increased by 2.3 months and median OS by 6.3 months. This survival advantage was confirmed in multivariate analysis. The tumoral expression increase of PD-L1 in these studies may partly have increased the tumour sensitivity to PD-1/PD-L1 inhibition.

2 STUDY OBJECTIVES

2.1 Primary objective

To determine the 1 year progression-free survival rate (based on RECIST v1.1.) under combined SABR and anti-PDL1 atezolizumab therapy:

- *in patients with metastatic colorectal cancer (cohort closed to inclusions);*
- *in patients with metastatic non-small lung cancer (cohort closed to inclusions);*
- *in patients with metastatic renal cell carcinoma (cohort closed to inclusions);*
- *in patients with metastatic sarcoma (cohort closed to inclusions).*

Each cohort will be analysed separately.

2.2 Secondary objectives

To determine, the progression-free survival (PFS) measured from the date of treatment initiation to the date of death or progression, whichever comes first under combined SABR and anti-PDL1 atezolizumab therapy using RECIST v 1.1 and modified RECIST (mRECIST).

To further describe, the efficacy of combination (SABR and atezolizumab) both on the irradiated lesions and the non-irradiated lesions based on tumour response indicators and clinical endpoints using RECIST 1.1 criteria and modified RECIST (mRECIST).

To determine the Treatment failure rate defined as the proportion of patients who cannot receive the planned irradiation dose or relative atezolizumab dose intensity below 75% of the initially targeted dose (i.e below 900 mg).

To evaluate the toxicity of atezolizumab in combination with SABR according to the NCI-CTCAE scale (version 4.03).

To assess tumor reponse indicators and clinical endpoints, assessed according to RECIST v1.1 at 6 weeks after the second SABR course.

In lung and sarcoma patiens who received a second SABR course after oligoprogression:

- To assess the objective response rate at 6 weeks after the second SABR course, using RECIST v1.1 and continuation of atezolizumab at the time of progression.
- To compare the modification of the size of the lesion which received the second SABR course versus the non-treated lesion (abscopal effect). Modification is assessed as the variation between the size at the time of the second SABR course and the size 6 weeks thereafter.
- To assess progression-free survival 2 (PFS2) measured from the date of the second SABR course to date of second progression or death whichever comes first. Patients alive and free of progression at the cut-off date will be censored at the last assessment date.
- Time to progression ratio (TTPr) in patient receiving a second SABR course:

To define the TTP ratio, let's denote:

- TTP1 the time from treatment initiation to the time of progression after first irradiation.
- TTP2 the time from first progression to the time of progression after the second SABR course. Patients receiving a second SABR course who die without a documented second progression will be counted as progressive as it is unlikely that patients die of a cause unrelated to the failure of the treatment and subsequent tumor progression. Patients alive and free of progression at the cut-off date will be censored at the last assessment date. Radiological progression is measured using RECIST v1.1 on the same tumor lesions over all the trial, whenever possible. TTP2 is defined as PFS2 as it is unlikely that patients die of cause unrelated to the treatment failure and subsequent tumor progression.

Exploratory objectives :

To evaluate the functional imaging changes using FDG PET/CT and tumour growth rates.

To evaluate the systemic immunologic anti-tumour response based on the sequential tumour biopsies and immunomonitoring on peripheral blood samples.

3 METHODOLOGY

This phase II study with direct individual benefit will evaluate, in patients with metastatic solid tumour, the efficacy and the tolerability of the atezolizumab anti-PD-L1 antibody in combination with SABR.

3.1 Treatment period

Drug: The anti-PD-L1 antibody atezolizumab will be administered at a dose of 1200 mg every 3 weeks.

Atezolizumab treatment will be administered as long as:

- Patients are experiencing clinical benefit, as assessed by an investigator, and
- In the absence of unacceptable toxicity or symptomatic deterioration attributed to disease progression after an integrated assessment of radiographic data, biopsy results (if available) and clinical status.

In case of progressive disease according to RECIST v1.1 criteria, patients will be permitted to continue atezolizumab treatment if they meet all of the following criteria:

- Evidence of clinical benefit as assessed by the Investigator,
- Absence of symptoms and signs (including worsening of laboratory values, e.g., new or worsening hypercalcemia) indicating unequivocal progression of disease,
- No decline in ECOG performance status that can be attributed to disease progression, and

- Absence of tumour progression at critical anatomical sites (e.g., leptomeningeal disease) that cannot be managed by protocol-allowed medical interventions.

Patients treated with atezolizumab in whom radiographic disease progression is confirmed at a subsequent tumour assessment may be considered for continuing study treatment at the discretion of the Investigator if they continue to meet the above criteria.

SABR: Stereotactic ablative Radiation therapy will be delivered concurrently with the 2rd cycle of atezolizumab (at week 4) using 6MV photons or other energies with standard field encompassing tumour.

At least one tumour site will receive radiation and another tumour site(s) should be not treated but evaluable by RECIST v1.1 in order to proper assess potential abscopal effect.

Hypofractionated SABR will be delivered with an equivalent biologic dose (BED) > 80 Gy. The recommended fractionation is 45 Gy (3 fractions of 15 Gy) but shall be adapted upon normal tissue tolerance constraint. Total duration of radiotherapy must not exceed eight days. Tumour size will be ≤ 4 cm according to recent imaging (CT or PET). The radiation dose will be prescribed to the 90% isodose line in order to deliver 95% of the planned dose to 95% of the planned tumour volume (PTV). The dose used to treat a given metastasis will be based on the location of the metastasis, as normal tissue toxicity is likely to arise from the organs at risk surrounding the metastasis.

In patients with lung cancer and sarcoma and in case of first progression defined using RECIST 1.1, a second SABR course can be delivered with the same conditions.

3.2 Follow-up period

- **Patients who discontinue atezolizumab for other reason than disease progression** will have **follow up visit** every 3 months up to 12 months or disease progression, whichever occurs first, for assessment of disease progression and collection of delayed toxicities.

- **Patients who discontinue atezolizumab for disease progression** will have an end of study visit 1 month after treatment discontinuation.

The end of the trial is defined as being the last protocol-specified visit of the last patient, *i.e* the last visit of the last patient during the follow-up period (up to 12 months after atezolizumab discontinuation) or the last attempt by the investigator to schedull this visit for the last patient.

During the follow-up period, patients are allowed to receive another anticancer therapy.

3.3 Flowchart:

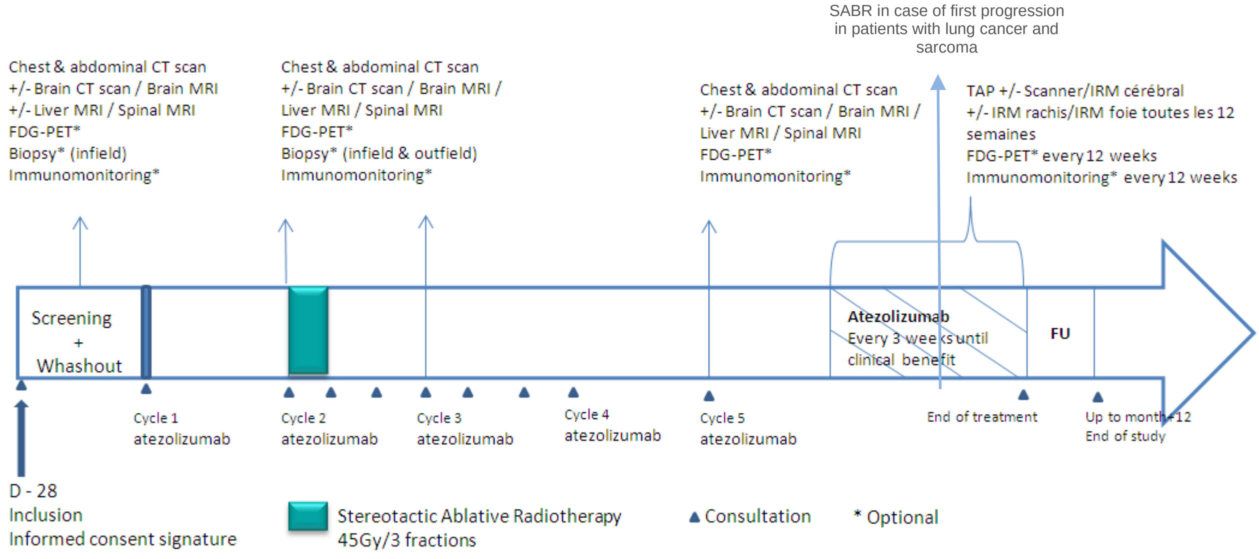

4 SELECTION OF PATIENTS

4.1 Inclusion criteria

1. Patients must be 18 years of age or older.

2. Histologically or cytologically proven metastatic solid tumours including:

   - *colorectal (CRC, Microsatellite instability negative and positive) in treatment failure as per the current standard recommendation (cohort closed to inclusions) ;*

   - *non-small cell lung cancer (NSCLC) pretreated by at least one line of treatment. Patients EGFR-mutant can be included only if they have been treated with, or developed toxicity with or refused to be treated with anti-EGFR therapy. Patients pre-treated by anti−PD1, or anti−PDL1 therapeutic antibodies can be included only if they have received at least 4 months of treatment (cohort closed to inclusions);*

   - *renal cell carcinoma (RCC) pretreated by at least one line therapy by a tyrosin kinase inhibitor (cohort closed to inclusions);*

   - *metastatic sarcomas of any type (soft tissue, bone, GISTs) pretreated by at least one line of standard therapy; at least three lines of standard TKi must be given in patients with GISTs. No enrolment restriction to certain sarcoma subtypes/groups was decided given the relative rarity of this disease type and that immunotherapy efficacy in certain histological subtypes is only preliminary (cohort closed to inclusions).*

3. Patients with at least:
- one measurable metastasis by RECIST 1.1 eligible for SABR in terms of dose constraints at organ at risk (refer to Appendix 1: Rules for SABR administration according to tumour location); distinct criteria apply regarding lung and liver metastases) and ≤ 4 cm, **and**
- one not treated measurable metastasis by RECIST 1.1. If all tumour sites are accessible to SABR, one of them will not be treated.

Metastase located within the proximal bronchial tree as defined in RTOG 0236 (refer to Appendix 1: Rules for SABR administration according to tumour location) or within the brain are not eligible for SABR treatment in the present study. However, it can be considered as a not treated evaluable metastase.

4. WHO performance status of 0-1

5. Evaluation by a radiation oncologist within 45 days prior to study registration, including imaging workup to document metastases (cf. description in assessment section)

6. Patients must have adequate organ function defined by the following laboratory results obtained within 28 days prior to the first study treatment:

   - Absolute neutrophil count of ≥ 1500/mm3;
   - Lymphocyte count ≥ 500 mm3;
   - Platelets ≥ 100,000/mm3;
   - Hemoglobin > 9 gr/dL;
   - Clearance Creatinine ≥ 50 mL/min;
   - Total bilirubin ≤ 1.5X ULN (unless Gilbert where 3X ULN is permitted);
   - Serum ALT and AST ≤ 2.5X ULN (unless documented liver metastases where ≤ 5X ULN is permitted),
   - ALK ≤ 2.5 ULN (unless documented bone or liver metastases where ≤5X ULN is permitted).

7. Life expectancy of more than 3 months

8. Patient must be aware of the investigational nature of the therapy and provide written informed consent.

9. Sexually active women of childbearing potential must agree to use a highly effective method of contraception supplemented with a barrier method, or to abstain from sexual activity during the study and **for at least 5 months after** the last dose of atezolizumab.

   Sexually active males patients must agree to use condom while on SABR treatment and for at least 90 days after SABR treatment. Taking into account the irradiated area, use of condom after SABR treatment can be shortened at investigator discretion. Also, their women of childbearing potential partner should use a highly effective method of contraception.

   Women who are not postmenopausal (≥ 12 months of non-therapy-induced amenorrhea) or surgically sterile must have a negative serum β-HCG pregnancy test result within 7 days prior to initiation of study drug.

A woman is considered of childbearing potential following menarche and until becoming post-menopausal (≥ 12 months of non-therapy-induced amenorrhea) unless permanently sterile. Permanent sterilization methods include hysterectomy, bilateral oophorectomy and bilateral salpingectomy.

A highly effective birth control method is a one which can achieve a failure rate of less than 1% per year when used consistently and correctly. Such methods include: combined (estrogen and progestogen containing) hormonal contraception; progestogen-only hormonal contraception; intrauterine device (IUD); intrauterine hormone-releasing system (IUS); bilateral tubal occlusion; vasectomized partner (on the understanding that this is the only one partner during the whole study duration), and sexual abstinence during the entire period of risk associated with atezolizumab. Following methods are considered as unacceptable methods (non-

exhaustive list): periodic abstinence (calendar, symptothermal, post-ovulation methods) and withdrawal (coitus interruptus).

10. Patients must be free of significant comorbid conditions that would preclude safe administration or completion of protocol therapy.

11. The irradiated and unirradiated tumour sites must be accessible to tumour biopsy (additional written consent required).

12. Patients must be affiliated to a social security system

4.2 Exclusion criteria

1. Known allergy to anti-PD-L1 including :
    - History of severe allergic anaphylactic reactions to chimeric, human or humanized antibodies, or fusion proteins.
    - Known hypersensitivity to CHO cell products or any component of the atezolizumab formulation.
2. Pregnant or breastfeeding women.
3. Any malignancy other than the disease under study in the past 5 years excepting skin cancers such as BCC or SCC.
4. Uncontrolled tumour-related pain.

    Patients requiring pain medication must be on a stable regimen at study entry.

    Asymptomatic metastatic lesions whose further growth would likely cause functional deficits or intractable pain (e.g., epidural metastasis that is not presently associated with spinal cord compression) should be considered for loco-regional therapy if appropriate prior to enrolment.
5. Uncontrolled pleural effusion, pericardial effusion, or ascites requiring recurrent drainage procedures (once monthly or more frequently).

    Patients with indwelling catheters (e.g., PleurX) are allowed.
6. Uncontrolled hypercalcemia (> 1.5 mmol/L ionized calcium or Ca > 12 mg/dL or corrected serum calcium > ULN) or symptomatic hypercalcemia requiring continued use of bisphosphonate therapy or denosumab.

    Patients who are receiving bisphosphonate therapy or denosumab specifically to prevent skeletal events and who do not have a history of clinically significant hypercalcemia are eligible. However, patients who are receiving denosumab prior to enrollment must be eligible to receive bisphosphonate instead and willing to switch to bisphosphonate therapy while on the study.
7. Severe, active co-morbidity, defined as follows:
    - Unstable angina and/or congestive heart failure requiring hospitalization within the last 6 months prior to registration;
    - Transmural myocardial infarction within the last 6 months prior to registration;
    - Acute bacterial or fungal infection requiring intravenous antibiotics at the time of registration;

- Uncontrolled Chronic Obstructive Pulmonary Disease or other respiratory illness requiring hospitalization or precluding study therapy within 30 days prior to registration
- History of idiopathic pulmonary fibrosis, organizing pneumonia (e.g., bronchiolitis obliterans), drug-induced pneumonitis, idiopathic pneumonitis, or evidence of active pneumonitis on screening chest CT scan
- History of radiation pneumonitis in the radiation field (fibrosis) is permitted.
- Severe hepatic disease, defined as a diagnosis of Child-Pugh Class B or C hepatic disease.
- Known HIV positive status.
- End-stage renal disease (i.e., on dialysis or dialysis has been recommended).
- Patients with active hepatitis B (defined as having a positive hepatitis B surface antigen [HBsAg] test at screening) or hepatitis C

  Patients with past hepatitis B virus (HBV) infection or resolved HBV infection (defined as having a negative HBsAg test and a positive antibody to hepatitis B core antigen [anti-HBc] antibody test) are eligible

  Patients positive for hepatitis C virus (HCV) antibody are eligible only if polymerase chain reaction (PCR) is negative for HCV RNA

8. Active or history of autoimmune or inflammatory disease, including but not limited to myasthenia gravis, myositis, autoimmune hepatitis, systemic lupus erythematosus, rheumatoid arthritis, inflammatory bowel disease, vascular thrombosis associated with anti-phospholipid syndrome, Wegener's granulomatosis, Sjögren's syndrome, Guillain-Barré syndrome, multiple sclerosis, vasculitis, or glomerulonephritis (see Appendix 3 for a more comprehensive list of autoimmune diseases)

   Patients with a history of autoimmune hypothyroidism on a stable dose of thyroid replacement hormone are eligible.

   Patients with controlled Type 1 diabetes mellitus on a stable insulin regimen are eligible.

   Patients with, vitiligo or psoriasis or grave's disease, not requiring systemic treatment within the last 2 years are eligible.

9. Metastases located to the brain and with clinical signs and/or leptomingeal carcinomatosis, or with indistinct borders making targeting not feasible.

   Metastases located to the brain and without clinical signs can be included.

10. Irradiation required for cord compression and for superior veina cava syndrome.

11. Irradiation by SABR should not include metastases located within 3 cm of the previously irradiated structures:

    - Spinal cord previously irradiated to > 40 Gy,
    - Brachial plexus previously irradiated to > 50 Gy,
    - Small intestine, large intestine, or stomach previously irradiated to > 45 Gy,
    - Brainstem previously irradiated to > 50 Gy,
    - Lung previously irradiated with prior V20Gy > 30%,

12. Metastasis localized to the central part of the chest and requiring irradiation (see "no fly zone" in Appendix 1: Rules for SABR administration according to tumour location).

13. Any approved anticancer therapy, including chemotherapy, hormonal therapy or radiotherapy under the following guidelines:

   - investigational or cytotoxic treatments within 4 weeks prior to the study treatment initiation and while on study treatment.
   - localized palliative radiotherapy within 2 weeks prior to the study treatment initiation and while on study treatment.
   - any approved TKIs within 3 weeks prior to the study treatment initiation and while on study treatment.

   however Hormone-replacement therapy or oral contraceptives are allowed.

14. Administration of a live, attenuated vaccine within 4 weeks prior to Cycle 1, Day 1 or anticipation that such a live attenuated vaccine will be required during the study.
15. Influenza vaccination should be given during influenza season only (example: approximately October to March in the Northern Hemisphere). Patients must not receive live, attenuated influenza vaccine (e.g., FluMist®) within 4 weeks prior to Cycle 1, Day 1 or at any time during the study
16. Treatment with systemic corticosteroids or other systemic immunosuppressive medications (including but not limited to prednisone, dexamethasone, cyclophosphamide, azathioprine, methotrexate, thalidomide, and anti−tumour necrosis factor [TNF] agents) within 2 weeks prior to Cycle 1, Day 1, or anticipated requirement for systemic immunosuppressive medications during the trial.

    Patients who have received acute, low-dose, systemic immunosuppressant medications (e.g., a one-time dose of dexamethasone for nausea) may be enrolled in the study.

    The use of inhaled corticosteroids for chronic obstructive pulmonary disease, mineralocorticoids (e.g., fludrocortisone) for patients with orthostatic hypotension, and low-dose supplemental corticosteroids for adrenocortical insufficiency are allowed.

17. Patient already enrolled in another therapeutic trial involving an investigational substance, and when such a substance has been taken during the previous 4 weeks.
18. Persons deprived of their freedom or under guardianship, or for whom it would be impossible to undergo the medical follow-up required by the trial, for geographic, social or psychological reasons
19. Prior treatment with CD137 agonists or immune checkpoint blockade therapies, anti−PD1, or anti−PDL1 therapeutic antibodies.

    Only patients with non-small cell lung cancer are allowed to have received anti−PD1, or anti−PDL1 therapeutic antibodies are eligible. Subjects who have received prior anti-PD-1/L1 therapies must have received at least 4 months of treatment (cohort closed to inclusions).

    Patients who have received prior treatment with anti−CTLA-4 may be enrolled, provided at least 5 half-lives (approximately 75 days) have elapsed from the last dose of anti-CTLA-4 to the first dose of atezolizumab and there was no history of severe immune-mediated adverse effects from anti−CTLA-4 (NCI CTCAE Grade 3 and 4)

20. Treatment with systemic immunostimulatory agents (including but not limited to interferon-alpha (IFN-α) and interleukin-2 (IL-2) within 4 weeks or five half-lives of the drug (whichever is shorter) prior to Cycle 1, Day 1.

4.3 Withdrawal criteria from study treatment and/or from the study

Premature discontinuation of study treatments does not mean that the patient prematurely stops the participation in the study.

The reasons for premature discontinuation of study treatements are:

- No clinical benefit, in the opinion of the investigator, for continuing with the study drug administration
- Progressive disease according to RECIST v1.1 criteria*****
- Unacceptable toxicity or symptomatic deterioration attributed to disease progression after an integrated assessment of radiographic data, biopsy results (if available) and clinical status
- Adverse events which preclude further study drug administration as described in section 5.3.1 and 5.3.2
- Pregnancy or intent to become pregnant
- Major deviation to protocol if it interferes with the study evaluations and/or if it jeopardises patient's safety, e.g. any medical event requiring administration of an unauthorised concomitant treatment
- Patient's removal
- Stopping rule, as defined in section 8.2, are fulfilled
- Study terminated by the sponsor

* However, if following conditions are met, patients will be permitted to continue atezolizumab treatment:

- Evidence of clinical benefit as assessed by the Investigator,
- Absence of symptoms and signs (including worsening of laboratory values, e.g., new or worsening hypercalcemia) indicating unequivocal progression of disease,
- No decline in ECOG performance status that can be attributed to disease progression, and
- Absence of tumour progression at critical anatomical sites (e.g., leptomeningeal disease) that cannot be managed by protocol-allowed medical interventions.

The reasons for premature discontinuation of the study drug and entire study are:

- Consent withdrawal
- Death
- Completed study follow-up

Patients who discontinue the study drug will have follow-up procedures performed as shown in Table 1 and in section 6.3.

If a patient does not return for a scheduled visit, every effort should be made to contact him (her). In any circumstance, every effort should be made to document the patient outcome.
The investigator should inquire about the reason for withdrawal, requests the patient to return for a final visit, and follow-up with the patient regarding any unresolved adverse events. The early termination final visit should include all assessments listed for the follow-up visit.

In case of consent withdrawal, the subject will not receive any further treatment or further study evaluation and no further data will be collected.

5 TREATMENTS

5.1 Identification of the study treatments

5.1.1 Atezolizumab

Formulation, Packaging, and Handling

Atezolizumab drug product is provided in a single-use, 20-mL USP/Ph. Eur. Type 1 glass vial as a colorless to slightly yellow, sterile, preservative-free clear liquid solution intended for IV administration. The vial is designed to deliver 20 mL (1200 mg) of atezolizumab solution but may contain more than the stated volume to enable delivery of the entire 20 mL volume. The atezolizumab drug product is formulated as 60 mg/mL atezolizumab in 20 mM histidine acetate, 120 mM sucrose, 0.04% polysorbate 20, pH 5.8.

Atezolizumab must be refrigerated at 2°C–8°C upon receipt until use.

Atezolizumab vials should not be used beyond the expiration date provided by the manufacturer. No preservative is used in the atezolizumab drug product; therefore, each vial is intended for single use only. Vial contents should not be frozen or shaken and should be protected from direct sunlight.

For further details, see the atezolizumab Investigator's Brochure.

Dose Solution Preparation and Storage:

Dose preparation will be done on the day when atezolizumab is administered.

Atezolizumab is administered only using 250 mL 0.9% sodium chloride saline IV bags and infusion lines equipped with 0.2 µm in-line filters. Bags may be constructed of polyvinyl chloride (PVC) or polyolefin (PO); the IV infusion line may be constructed in PVC or polyethylene; and the 0.2 µm in-line filters may be constructed in polyethersulfone (PES).

The required volume of atezolizumab for a patient is 20 mL. No dilution of the vial contents is required.

Atezolizumab Drug Product should be held at room temperature for 30 minutes prior to removal from the vials.

Dose solutions are prepared by removing 20 mL of saline solution from the IV bag prior to the addition of 20 mL of atezolizumab Drug Product. The IV bags containing atezolizumab should be mixed and handled gently. Once the atezolizumab dose solution is prepared, it may be stored at 2°C–8°C (36°F–46°F) and/or at room temperature for up to 8-hours prior to the start of infusion. However, if the infusion is interrupted and the combined storage and dose hold time of the diluted IV bags exceeds the 8-hour limit, prepare a new dose solution to resume the infusion.
If the dose solution is stored at 2°C–8°C (36°F–46°F), it should be removed from refrigeration and allowed to reach room temperature prior to administration. Protect dose solutions from intense light and heat.

Labelling

The product will be labelled according to recommendations set in Appendix 13 of the European Guide to Good Manufacturing Practice.

Compliance and study drug accountablity:

A qualified individual responsible for dispensing the study drug will prepare the correct dose according to the protocol. This individual will write the date dispensed and study subject number and initials on the study drug vial label and on the Drug Accountability Record. This individual will also record the study drug batch or lot number received by each study subject during the study.

Atezolizumab will be provided by the Sponsor. The investigational site will acknowledge receipt of IMP, to confirm the shipment condition and content. Any damaged shipments will be replaced.

The investigator is responsible for the control of drugs under investigation. Adequate records of the receipt (e.g., Drug Receipt Record) and disposition (e.g., Drug Dispensing Log) of the study drug must be maintained. The Drug Dispensing Log must be kept current and should contain the following information:

- the identification of the Study Subject to whom the study drug was dispensed (for example Study Subject initials and date of birth);
- the date(s) of the study drug dispensed to the Study;
- All records and drug supplies must be available for inspection by the Gustave Roussy Monitor [at every monitoring visit].

IMPs will either be disposed of at the study site according to the study site's institutional standard operating procedure or returned to the Sponsor with the appropriate documentation. The site's method of IMP destruction must be agreed upon by the Sponsor. Local or institutional regulations may require immediate destruction of used investigational medicinal product for safety reasons. In these cases, it may be acceptable for investigational study site staff to destroy dispensed investigational product before a monitoring inspection provided that source document verification is performed on the remaining inventory and reconciled against the documentation of quantity shipped, dispensed, returned, destroyed and provided that adequate storage and integrity of drug has been confirmed.

The site must obtain written authorization from the Sponsor before any IMP is destroyed, and IMP destruction must be documented on the appropriate form.

Written documentation of destruction must contain the following:

- Identity [batch numbers or Study Subject numbers] of investigational product[s] destroyed
- Quantity of investigational product[s] destroyed
- Method of destruction
- Name and signature of responsible person [or company] who destroyed investigational product(s)

Accurate records of all IMPs received at, dispensed from, returned to, and disposed of by the study site should be recorded on the Drug Inventory Log.

5.1.2 *Stereotactic Ablative Radiation Therapy (SABR)*

SABR will be administered concurrently during the second cycle of the anti-PD-L1 Atezolizumab (week 4) and in case of first progression in patients with lung cancer and sarcoma.

SABR will be delivered using 6MV photons or other energies with standard field encompassing tumour. Hypofractionated SABR will be delivered within 3 fractions in total to a total of 45 Gy (3 fractions of 15 Gy). In order to comply with the QUANTEC normal tissue tolerance guidelines, the dose may be adapted according to dosimetric constraints but must never be inferior to 33 Gy in fractions of 11 Gy.

Tumour size will be ≤ 4 cm according to recent imaging (CT, MR or PET).

The radiation dose will be prescribed to the 90% isodose line in order to deliver 95% of the planned dose to 95% of the planned tumour volume (PTV).

Patients will be eligible if dose constraints to organ at risk are respected.

SABR software tools and accurate dose calculations allow for better targeting of tumours, while providing motion management for precise dose delivery. Generally, this technique includes:

- 4D CT data integration for moving target such as lung tumours
- Patient's immobilization with a vacuum cushion.
- Precise type B dose calculation algorithm (Monte Carlo, or other type B dose calculation algorithms)
- Monitoring of respiratory motion.
- Real-time detection and compensation of tumour motion (on-board CT)

The definition of volumes will be in accordance with ICRU Reports #50, #62 and #83.

SABR will be given on alternate days and only type B dose calculation algorithms will be accepted.

Margins from GTV (or ITV) to PTV are of 5 - 10 mm to account for set-up error and organ motion.

Any organ that is traversed by part or all of a beam should be contoured so that the dose it receives can be assessed. Organs should be outlined by the treating radiotherapist (or dosimetrist and checked by treating radiotherapist). Dose constraints are adapted to the standard recommendations (cf Appendix 1: Rules for SABR administration according to tumour location).

5.2 Dosage and schedule of treatment

5.2.1 Atezolizumab

The dose of atezolizumab will be 1200 mg administered by intravenous infusion every 3 weeks (21 [+ 3] days).

Administration of atezolizumab will be performed in a setting with emergency medical facilities and staff who are trained to monitor for and respond to medical emergencies.

The initial dose of atezolizumab will be delivered over 60 (± 15) minutes. If the first infusion is tolerated without infusion-associated adverse events, the second infusion may be delivered over 30 (± 10) minutes. If the 30-minute infusion is well tolerated, all subsequent infusions may be delivered over 30 (± 10) minutes.

For the infusion, the patient’s vital signs (heart rate, respiratory rate, blood pressure, and temperature) should be determined within 60 minutes before, and 30 (± 10) minutes and after the infusion. For subsequent infusions, vital signs will be collected within 60 minutes before infusion and at the end of the infusion, if clinically indicated.

Patients will be informed about the possibility of delayed post-infusion symptoms and instructed to contact their study physician if they develop such symptoms.

No premedication will be allowed for the first dose of atezolizumab. Premedication may be administered for Cycles ≥ 2 at the discretion of the treating physician. The management of infusion-related reactions will be performed according to severity as described in section 5.3.2.6

In the event of a suspected anaphylactic reaction during study drug infusion, the following procedures should be performed:

- Stop the study drug infusion
- Apply a tourniquet proximal to the injection site to slow systemic absorption of study drug. Do not obstruct arterial flow to the limb.
- Maintain an adequate airway
- Administer antihistamines, epinephrine or other medications as required by patient status and directed by the physician in charge
- Continue to observe the patient and document observations

Guidelines for dosage modification, treatment interruption or discontinuation, and the management of specific adverse events are provided 5.3.2.

Any overdose or incorrect administration of study drug should be noted on the Study Drug Administration Case Report Form (CRF). AEs associated with an overdose or incorrect administration of study drug should be recorded on the Adverse Events CRF pages.

Cessation of immunotherapy for long-term responders

The optimal duration of treatment for immunotherapy remains debated. Phase 3 clinical trials in NSCLC and melanoma were designed with treatment until progression or unacceptable

toxicity. Responses however can be durable after discontinuation in patients who received receiving 2 years of treatment.

In melanoma patients, the KEYNOTE 006 trial compared pembrolizumab (10 mg/kg2 weekly or 3 weekly) to ipilimumab (3 mg/kg 3 weekly for 4 doses) and trial design stipulated planned discontinuation of treatment at 24 months or earlier if confirmed complete response (CR). If discontinued at 24 months, patients were allowed to recommence 12 months of pembrolizumab upon progressive disease. Of the 834 patients enrolled, 103 of 566 (19%) patients receiving one of the two pembrolizumab arms received the full 24 months of treatment and discontinued. At a median follow up of 20 months post-discontinuation, 86% of these patients had ongoing responses [Robert].

In previously treated non-small cell lung cancer (NSCLC) patients with PD-L1 positive tumors (TPS >1%), the KEYNOTE 010 trial randomized to one of three arms; pembrolizumab 10 mg/kg3 weekly, pembrolizumab 2 mg/kg3 weekly or docetaxel for 35 cycles (2 years). At a median follow up of 42.6 months, 79 of 690 (11%) enrolled patients had completed the 35 cycles being 2 years of treatment and of these, 95% had achieved CR or partial response (PR) as best overall response [Herbst].

This data supports the evidence of durability of response upon discontinuation. Durability post-discontinuation due to toxicity is also a feature of immunotherapy. In patients who have not progressed after 2 years of anti-PD(L)1 treatment, it is then appropriate to consider and discuss treatment discontinuation.

Therefore, if active disease is not detected on CT or PET/CT scans discontinuing anti-PD-L1 therapy after 24 months will be proposed.

5.2.2 SABR

It will be administered concurrently during the second cycle of the anti-PD-L1 (week 4) and in case of a first radiological progression as per RECIST v1. in patients with lung cancer and sarcoma.

SABR will be delivered using 6MV photons or other enegies with standard field encompassing tumour. Hypofractionated SABR will be delivered with an equivalent biologic dose > 80 Gy (45 Gy in 3 fractions of 15 Gy). The recommended fractionation is 45 Gy (3 fractions of 15 Gy) but shall be adapted upon normal tissue tolerance constraints (Annex 1). Total duration of radiotherapy must not exceed eight days and a window of ± 3 days allowed to start the treatment.

Tumour size will be ≤ 4 cm according to recent imaging (CT or PET).

The radiation dose will be prescribed to the 90% isodose line in order to deliver 95% of the planned dose to 95% of the planned tumour volume (PTV). The dose used to treat a given metastasis will be based on the location of the metastasis, as normal tissue toxicity is likely to arise from the organs at risk surrounding the metastasis.

5.3 Adaptation of doses and interruption of treatment

5.3.1 Doses modifications for SABR

Management of treatments during protocol treatment toxicity according to the NCI-CTCAE scale (CTCAE v 5.0)

Cutaneous toxicity (radiotherapy-associated dermatitis)

- **Grade 4**, SABR must be discontinued

Pulmonary toxicity (dyspnea, pneumopathy)

- **Grade 3/4,** SABR and atezolizumab must be interrupted. Patients must then be reassessed every week

SABR must only be discontinued in the event of a grade or 3 or 4 pharyngo-oesophageal, cutaneous or pulmonary reaction in the irradiated field. SABR and/or atezolizumab must only be reintroduced after the toxicity has recovered to grade 2 or less.

Digestive toxicity within the field:

Medical management includes treating diarrhea, dehydration, malabsorption, and abdominal or rectal discomfort. Symptoms usually resolve with medications, dietary changes, and rest. If symptoms become severe despite these measures, a treatment break may be warranted.

In addition to these medications, opioids may offer relief from abdominal pain. If proctitis or oesophgitis is present, steroid foam given rectally may offer relief from symptoms.

If esophagitis occurs, making oral feeding impossible, placement of a nasogastric tube must be considered.

In the event of grade 3 esophagitis that is poorly tolerated in spite of appropriate treatment, SABR can either be continued with enteral and/or parenteral feeding plus appropriate analgesic treatment, or it may be discontinued for less than 5 days until improvement (≤ grade 2 or grade 3 that is well tolerated with appropriate treatment).

- Grade 4 (oesophagitis, diarrhea, abdominal pain), SABR and atezolizumab must be interrupted. Patients must then be reassessed every week

- Grade 3 (diarrhea, abdominal pain), Atezolizumab must be discontinued only if symptoms do not improve despite discontinuation of SABR for 8 days.

5.3.2 Management of atezolizumab-specific adverse events

Toxicities associated or possibly associated with atezolizumab treatment should be managed according to standard medical practice. Additional tests, such as autoimmune serology or biopsies, should be used to determine a possible immunogenic etiology.

Although most immune-related adverse events observed with immune-modulatory agents have been mild and self-limiting, such events should be recognized early and treated promptly to avoid potential major complications. Discontinuation of atezolizumab may not have an immediate therapeutic effect and in severe cases, immune-related toxicities may require acute

management with topical corticosteroids, systemic corticosteroids or other immunosuppressive agents.

Patients and family caregivers should receive timely and up-to-date information about immunotherapies, their mechanism of action, and the clinical profile of possible immune-related -adverse events prior to initiating therapy and throughout treatment and survival follow-up. There should be a high level of suspicion that new symptoms are treatment related.

The following are general recommendations for management of any other adverse events that may occur and are not specifically listed in subsequent subsections:

- In general, atezolizumab therapy should be continued with close monitoring for Grade 1 toxicities, with the exception of some neurologic toxicities.
- Consider holdingwithholding atezolizumab for most Grade 2 toxicities and resume when symptoms and/or laboratory values resolve to Grade 1 or better. Corticosteroids (initial dose of 0.5-1 mg/kg/day of prednisone or equivalent) may be administered.
- For Grade 2 recurrent or persistent (lasting for more than 5 days) events, treat as a Grade 3 event.
- HoldWithhold atezolizumab for Grade 3 toxicities and initiate treatment with high--dose corticosteroids (162 mg/kg/day oral prednisone or equivalent). Corticosteroids should be tapered over 1 month to 10 mg/day oral prednisone or equivalent, before atezolizumab can be resumed. If symptoms do not improve within 48 to 72 hours of high-dose corticosteroid use, other immunosuppressants may be offered for some toxicities.
- In general, Grade 4 toxicities warrant permanent discontinuation of atezolizumab treatment, with the exception of endocrinopathies that are controlled by hormone-replacement therapy.
- The investigator should consider the benefit□risk balance for a given patient prior to further administration of atezolizumab. Atezolizumab should be permanently discontinued in patients with life-threatening immune-related adverse events. Resumption of atezolizumab may be considered in patients who are deriving benefit and have fully recovered from the immune-mediated event. The decision to rechallenge patients with atezolizumab should be based on the investigator's benefit-risk assessment of the benefits and risks and documented by the investigator. The Medical Monitor is available to advise as needed..

Guidelines for managing patients who experience selected adverse events are provided in the following sections. Management guidelines are presented by adverse event severity based on the National Cancer Institute Common Terminology Criteria for Adverse Events (NCI CTCAE).

5.3.2.1 Pulmonary Events

Pulmonary events may present as new or worsening cough, chest pain, fever, dyspnea, fatigue, hypoxia, pneumonitis, and pulmonary infiltrates. Patients will be assessed for pulmonary signs and symptoms throughout the study and will also have CT scans of the chest performed at every tumor assessment.

All pulmonary events should be thoroughly evaluated for other commonly reported etiologies such as pneumonia or other infection, lymphangitic carcinomatosis, pulmonary embolism, heart failure, chronic obstructive pulmonary disease, or pulmonary hypertension. COVID-19 evaluation should be performed per institutional guidelines where relevant.

Table 2: Management guidelines for pulmonary events including pneumonitis

**Severity**	**Management**
Grade 1	• Continue atezolizumab with close monitoring<br>• Re-evaluate on serial imaging<br>• Consider pulmonary consultation<br>• For grade 1 pneumonitis, consider withholding Atezolizumab<br>• Consider resumingon radiographic evidenceof improvement
Grade 2	• Withhold atezolizumab for up to 12 weeks after event onset. [a]<br>• Refer patient to Pulmonary and infectious disease specialists and consider bronchoscopy or BAL with or without transbronchial biopsy.<br>• Initiate treatment with 1−2 mg/kg/day oral prednisone or equivalent.<br>• Resume atezolizumab if event resolves to Grade 1 or better within 12 weeks. [a, b]<br>• Permanently discontinue atezolizumab and contact the principal investigator if event does not resolve to Grade 1 or better within 12 weeks.[a, b, c, d]<br>• For recurrent events, or events with no improvement after 48-72 hours of corticosteroids, treat as a Grade 3 or 4 event.
Grade 3 - 4	• Permanently discontinue atezolizumab and contact the principal investigator.[c,d]<br>• Oral or IV broad-spectrum antibiotics should be administered in parallel to the immunosuppressive treatment<br>• Bronchoscopy or BAL with or without transbronchial biopsy is recommended<br>• Initiate treatment with 1−2 mg/kg/day oral prednisone or equivalent.<br>• If event does not improve within 48 hours after initiating corticosteroids, consider adding an immunosuppressive agent.<br>• If event resolves to Grade 1 or better, taper corticosteroids over ≥ 1 month.

BAL = bronchoscopic alveolar lavage; ID = infectious disease, IVIG = intravenous immunoglobulin

a If corticosteroids have been initiated, they must be tapered over ≥ 1 month to ≤ 10 mg/day oral prednisone or equivalent before atezolizumab can be resumed.
b Atezolizumab may be withheld for a period of time beyond 12 weeks to allow for corticosteroids to be reduced to ≤ 10 mg/day oral prednisone or equivalent. The acceptable length of the extended period of time must be agreed upon by the investigator and the Medical Monitor.
c Resumption of atezolizumab may be considered in patients who are deriving benefit and have fully recovered from the immune-related event. Patients can be rechallenged with atezolizumab only after approval has been documented by both the investigator (or an appropriate delegate) and the sponsor.
d In case of pneumonitis, atezolizumab should not be resumed after permanent discontinuation.

5.3.2.2 Hepatic Events

Eligible patients must have adequate liver function, as manifested by measurements of total bilirubin and hepatic transaminases, and liver function will be monitored throughout study treatment.

Patients with right upper-quadrant abdominal pain and/or unexplained nausea or vomiting should have liver function tests (LFTs) performed immediately and reviewed before administration of the next dose of study drug.

For patients with LFTs increase, concurrent medications, viral hepatitis, and toxic or neoplastic etiologies should be considered and addressed, as appropriate.

Table 3: Management guidelines for hepatic events

Severity	Management
Grade 1	• Continue atezolizumab<br>• Monitor LFTs until values resolve to within normal limits or baseline values
Grade 2	• All events:<br>o Monitor LFTs more frequently until return to baseline values.<br>• Events of > 5 days' duration:<br>o Withhold atezolizumab for up to 12 weeks after event onset. [a]<br>o Initiate treatment with 1−2 mg/kg/day oral prednisone or equivalent.<br>o Resume atezolizumab if event resolves to Grade 1 or better within 12 weeks. [a, b]<br>o Permanently discontinue atezolizumab and contact the principal investigator if event does not resolve to Grade 1 or better within 12 weeks. [a, b, c]
Grades 3–4	• Permanently Discontinue therapy and contact the principal investigator [c].<br>• Consider GI consult and liver biopsy to establish etiology of hepatic injury<br>• Initiate treatment with 1−2 mg/kg/day oral prednisone or equivalent<br>• If event does not improve within 48 hours after initiating corticosteroids, consider adding an immunosuppressive agent.<br>• Taper corticosteroids over ≥1 month, when symptoms improve to Grade 0 or Grade 1.

GI = gastrointestinal; LFT = liver function test; TNF-α = tumor necrosis factor alpha.
a Atezolizumab may be withheld for a longer period of time (i.e., >12 weeks after event onset) to allow for corticosteroids (if initiated) to be reduced to ≤ 10 mg/day oral prednisone or equivalent. Protocol defined windows for treatment discontinuation may differ and should be followed accordingly.

b If corticosteroids have been initiated, they must be tapered over ≥ 1 month to ≤ 10 mg/day oral prednisone or equivalent before atezolizumab can be resumed.

c Resumption of atezolizumab may be considered in patients who are deriving benefit and have fully recovered from the immune-related event. Patients can be rechallenged with atezolizumab only after approval has been documented by both the investigator (or an appropriate delegate) and the Medical Monitor.

5.3.2.3 Gastrointestinal Events

All events of diarrhea or colitis should be thoroughly evaluated for other more common etiologies. For events of significant duration or magnitude or associated with signs of systemic inflammation or acute phase reactants (e.g., increased c-reactive protein, platelet count, or bandemia), it is recommended to do the following:

- Perform sigmoidoscopy (or colonoscopy, if appropriate) with colonic biopsy with three to five specimens for standard paraffin block to check for inflammation and lymphocytic infiltrates for confirmation of the diagnosis of colitis.

Table 4: Management guidelines for gastrointestinal events (diarrhea/colitis)

Severity	Management
Grade 1 diarrhea or colitis	• Continue atezolizumab<br>• Initiate Symptomatic treatment<br>• Endoscopy is recommended if symptoms persist for > 7 days.<br>• Close monitoring
Grade 2 diarrhea or Grade 2 colitis	• Withhold atezolizumab for up to 12 weeks after event onset.[a]<br>• Initiate Symptomatic therapy<br>• If strong clinical suspicion for immune-mediated colitis, initiate empiric IV corticosteroids while waiting for definitive diagnosis.<br>• GI consultation is recommended<br>• If persists > 5 days or recurs: initiate treatment with 1-2 mg/kg/day oral prednisone or equivalent. If the event does not improve within 48 hours after initiating corticosteroids, consider adding an immunosuppressive agent.<br>• Resume atezolizumab if event resolves to Grade 1 or better within 12 weeks.[a, b]<br>• Permanently discontinue atezolizumab and contact principal investigator if event does not resolve to Grade 1 or better within 12 weeks [c]
Grade 3 diarrhea or colitis	• Withhold atezolizumab. for up to 12 weeks after event onset.[a]<br>• GI referral and confirmation biopsy<br>• Treat with IV steroids (1–2 mg/kg/day methylprednisolone or equivalent) and convert to oral steroids (prednisone 1-2 mg/kg/day or equivalent) after improvement.<br>• If event does not improve within 48 hours after initiating corticosteroids, consider adding an immunosuppressive agent.<br>• Resume atezolizumab if event resolves to Grade 1 or better within 12 weeks. [a, b]<br>• Permanently discontinue atezolizumab and contact the principal

Severity	Management
	investigator if event does not resolve to Grade 1 or better within 12 weeks [c]
Grade 4 diarrhea or colitis	• Permanently Discontinue atezolizumab and contact the principal investigator. [C]<br>• GI referral and confirmation biopsy<br>• Treat with IV steroids (1–2 mg/kg/day methylprednisolone or equivalent) and convert to oral steroids (prednisone 1-2 mg/kg/day or equivalent) after improvement.<br>• If event does not improve within 48 hours after initiating corticosteroids, consider adding an immunosuppressive agent.<br>• If event resolves to Grade 1 or better, taper corticosteroids over ≥ 1 month

GI = gastrointestinal; IV = intravenous; PO = orally; TNF-α = tumor necrosis factor alpha.

a. Atezolizumab may be withheld for a longer period of time (i.e., > 12 weeks after event onset) to allow for corticosteroids (if initiated) to be reduced to ≤ 10 mg/day oral prednisone or equivalent. Protocol defined windows for treatment discontinuation may differ and should be followed accordingly.

b. If corticosteroids have been initiated, they must be tapered over ≥ 1 month to ≤ 10 mg/day oral prednisone or equivalent before atezolizumab can be resumed.

c. Resumption of atezolizumab may be considered in patients who are deriving benefit and have fully recovered from the immune-related event. Patients can be rechallenged with atezolizumab only after approval has been documented by both the investigator (or an appropriate delegate) and the sponsor.

5.3.2.4 Endocrine events

Thyroid disorders ~~or~~ adrenal insufficiency diabetes mellitus and pituitary disorders have been associated with the administration of atezolizumab.

Patients with unexplained symptoms such as headache, fatigue, myalgias, impotence, mental status changes, or constipation should be investigated for the presence of thyroid, pituitary, or adrenal endocrinopathies.

An endocrinologist should be consulted if an endocrinopathy is suspected. TSH and free T3 and T4 levels should be obtained to determine whether thyroid abnormalities are present. Pituitary hormone levels and function tests (e.g. TSH, growth hormone, luteinizing hormone, follicle-stimulating hormone, testosterone, prolactin, adrenocorticotropic hormone levels and ACTH stimulation test) and magnetic resonance imaging (MRI) of the brain (with detailed pituitary sections) may help to differentiate primary pituitary insufficiency from primary adrenal insufficiency.

Table 5: Management guidelines for endocrine events

Severity	Management
Grade 1 hypothyroidism	• Continue atezolizumab.<br>• Start thyroid replacement hormone.<br>• Monitor TSH closely.
Grade 2	

Severity	Management
hypothyroidism	• Consider withholding atezolizumab.<br>• Initiate treatment with thyroid replacement hormone.<br>• Monitor TSH closely.<br>• Consider patient referral to endocrinologist.<br>• Resume atezolizumab when symptoms are controlled and thyroid function is improving.
Grade 3 or 4 hypothyroidism	• Withhold atezolizumab.<br>• Start thyroid replacement hormone.<br>• Monitor TSH closely.<br>• Refer to an endocrinologist.<br>• Admit patient to hospital for developing myxedema (bradycardia, hypothermia and altered mental status).<br>• Restart atezolizumab when symptoms are controlled and thyroid function is improving.<br>• Permanently discontinue atezolizumab and contac the principal investigator for life-threatening immune-mediated hypothyroidism. [c]
Grade 1 hyperthyroidism	**TSH ≥0.1 mU/L and <0.5 mU/L:**<br>• Continue atezolizumab.<br>• Monitor TSH every 4 weeks.<br>• Consider referral to an endocrinologist.<br>**TSH <0.1 mU/L:**<br>• Follow guidelines for grade 2 hyperthyroidism.<br>• Consider referral to an endocrinologist.
Grade 2 hyperthyroidism	• Consider withholding atezolizumab.<br>• Start treatment with anti-thyroid drug such as methimazole or carbimazole as needed.<br>• Consider referral to an endocrinologist.<br>• Restart atezolizumab when symptoms are controlled and thyroid function is improving
Grade 3 or 4 hyperthyroidism	• Withhold atezolizumab.<br>• Initiate treatment with anti-thyroid drugs such as methimazole or carbimazole as needed.<br>• Refer to endocrinologist<br>• Resume atezolizumab when symptoms are controlled and thyroid function is improving.<br>• Permanently discontinue atezolizumab and contact the Medical Monitor for life-threatening immune-mediated hyperthyroidism.c

Severity	Management
Symptomatic adrenal insufficiency, Grade 2−4	• Withhold atezolizumab for up to 12 weeks.[a] • Refer patient to endocrinologist. • Perform appropriate imaging. • Initiate treatment with 1–2 mg/kg/day intravenous methylprednisolone or equivalent and convert to 1-2 mg/kg/day oral prednisone or equivalent upon improvement. • Resume atezolizumab if event resolves to Grade 1 or better and patient is stable on replacement therapy (if required) within 12 weeks.[a,b] • If event does not resolve to Grade 1 or better or patient is not stable on replacement therapy while withholding atezolizumab, permanently discontinue atezolizumab and contact the principal investigator
Hyperglycemia, Grade 1 or 2	• Continue atezolizumab. • Investigate for diabetes. If patient has Type 1 diabetes, treat as a Grade 3 event. If patient does not have Type 1 diabetes, treat as per institutional guidelines. • Initiate treatment with insulin if needed. • Monitor for glucose control.
Hyperglycemia, Grade 3 or 4	• Withhold atezolizumab. • Initiate treatment with insulin. • Evaluate for diabetic ketoacidosis and manage as per institutional guidelines • Monitor for glucose control. • Resume atezolizumab when symptoms resolve and glucose levels are stable.
Hypophysitis Grade 2-3	• Withhold atezolizumab for up to 12 weeks after event onset. [a] • Refer patient to endocrinologist. • Perform brain MRI (pituitary protocol). • Initiate treatment with 1-2 mg/kg/day IV methylprednisolone or equivalent and convert to 1-2 mg/kg/day oral prednisone or equivalent upon improvement.[a] • Initiate hormone replacement therapy if clinically indicated. • If event resolves to Grade 1 or better, resume atezolizumab. [b] • If event does not resolve to Grade 1 or better while withholding atezolizumab, permanently discontinue atezolizumab and contact the principal investigator . [c] • For recurrent hypophysitis, treat as a Grade 4 event.

Severity	Management
Hypophysitis Grade 4	• Permanently discontinue atezolizumab and contact the principal investigator.[c]<br>• Refer patient to endocrinologist.<br>• Perform brain MRI (pituitary protocol).<br>• Initiate treatment with 1-2 mg/kg/day IV methylprednisolone or equivalent and convert to 1-2 mg/kg/day oral prednisone or equivalent upon improvement.[a]<br>• Initiate hormone replacement therapy if clinically indicated.

IV = intravenous; T4 = thyroxine; TSH = thyroid stimulating hormone.

a. Atezolizumab may be withheld for a longer period of time (i.e., >12 weeks after event onset) to allow for corticosteroids (if initiated) to be reduced to ≤ 10 mg/day oral prednisone or equivalent. Protocol defined windows for treatment discontinuation may differ and should be followed accordingly.

b. If corticosteroids have been initiated, they must be tapered over ≥ 1 month to ≤ 10 mg/day oral prednisone or equivalent before atezolizumab can be resumed

c. Resumption of atezolizumab may be considered in patients who are deriving benefit and have fully recovered from the immune-related event. Patients can be rechallenged with atezolizumab only after approval has been documented by both the investigator (or an appropriate delegate) and the Medical Monitor.

5.3.2.5 Ocular events

An ophthalmologist should evaluate visual complaints (e.g., uveitis, retinal events).

Table 6: Management guidelines for Ocular events

Severity	Management
Grade 1	• Continue atezolizumab.<br>• Patient referral to ophthalmologist is strongly recommended.<br>• Initiate treatment with topical corticosteroid eye drops and topical immunosuppressive therapy.<br>• If symptoms persist, treat as a Grade 2 event.
Grades 2	• Withhold atezolizumab.<br>• Evaluation by an ophthalmologist is strongly recommended.<br>• Treat with topical corticosteroid eye drops and topical immunosuppressive therapy.<br>• Resume atezolizumab if event resolves to Grade 1 or better within 12 weeks.[a, b]<br>• Permanently discontinue atezolizumab and contact the principal investigator if event does not resolve to Grade 1 or better within 12 weeks.[a, b, c]
Grades 3–4	• Permanently discontinue treatment and contact the principal investigator.[c]<br>• Refer patient to ophtalmologist<br>• Start 1-2 mg/kg/day prednisone or equivalent<br>• Taper steroids over ≥ 1 month after symptoms improve to Grade 0 or Grade 1.

[a] If corticosteroids have been initiated, they must be tapered over ≥ 1 month to ≤ 10 mg/day oral prednisone or equivalent before atezolizumab can be resumed.
[b] Atezolizumab may be withheld for a period of time beyond 12 weeks to allow for corticosteroids to be reduced to ≤ 10 mg/day oral prednisone or equivalent. The acceptable length of the extended period of time must be agreed upon by the investigator and the sponsor.

[c] Resumption of atezolizumab may be considered in patients who are deriving benefit and have fully recovered from the immune-related event. Patients can be rechallenged with atezolizumab only after approval has been documented by both the investigator (or an appropriate delegate) and the sponsor

5.3.2.6 Immune-mediated Cardiac events

In high-risk patients (including those with abnormal baseline cardiac troponin levels, when available), transthoracic echocardiogram (TTE) monitoring should be considered, as clinically indicated, and based on local clinical practice. Management guidelines for cardiac events are provided in Table 7.

Immune-Mediated Myocarditis

Patients will be assessed for cardiac signs and symptoms throughout the study.
Immune-mediated myocarditis should be suspected in any patient presenting with signs or symptoms suggestive of myocarditis, including, but not limited to, laboratory (e.g., troponin, B-type natriuretic peptide) or cardiac imaging abnormalities, dyspnea, chest pain, palpitations, fatigue, decreased exercise tolerance, or syncope. Myocarditis may also be a clinical manifestation of myositis or associated with pericarditis and should be managed accordingly. Immune-mediated myocarditis needs to be distinguished from myocarditis resulting from

infection (commonly viral, e.g., in a patient who reports a recent history of gastrointestinal illness), ischemic events, underlying arrhythmias, exacerbation of preexisting cardiac conditions, or progression of malignancy.

All patients with possible myocarditis should be urgently evaluated by performing cardiac enzyme assessment, an ECG, a chest X-ray, an echocardiograma (TTE) for evaluation of left ventricular injection fraction and global longitudinal strain, and a cardiac MRI as appropriate per institutional guidelines. A cardiologist should be consulted. An endomyocardial biopsy may be considered to enable a definitive diagnosis and appropriate treatment, if clinically indicated.

Patients with signs and symptoms of myocarditis, in the absence of an identified alternate etiology, should be treated according to the guidelines in Table 7.

Atezolizumab should be permanently discontinued for all grades of immune-related myocarditis. Corticosteroids and/or additional immunosuppressive agents should be administered as clinically indicated. (see also section 5.3.2.16)

Immune-Mediated Pericardial disorders

Pericardial disorders encompass a range of diseases of the pericardium including pericarditis, pericardial effusion and cardiac tamponade.

Pericardial disorders are also known to be associated with drugs including immune-checkpoint inhibitors.

Pericarditis may be associated with pericardial effusion, which if significant in volume may result in hemodynamic instability and progress to cardiac tamponade. Cardiac tamponade is a life-threatening condition and should be treated as a medical emergency.

Immune-mediated pericarditis should be suspected in any patient presenting with chest pain and may be associated with immune-mediated myocarditis.

Immune-mediated pericardial effusion and cardiac tamponade should be suspected in any patient presenting with chest pain associated with dyspnea or hemodynamic instability.

Patients should be evaluated for other causes of pericardial disorders such as infection (commonly viral), cancer (e.g . metastatic disease), cancer treatment (e.g. chest radiotherapy) cardiac injury (e.g. cardiac injury due to myocardial infarction or iatrogenesis), and autoimmune disorders, and should be managed accordingly.

All patients with suspected pericardial disorders should be urgently evaluated by performing an ECG, chest X-ray, transthoracic echocardiogram, and cardiac MRI as appropriate per institutional guidelines. A cardiologist should be consulted. Pericardiocentesis should be considered for diagnostic or therapeutic purposes, if clinically indicated.

Patients with signs and symptoms of pericarditis, pericardial effusion, or cardiac tamponade, in the absence of an identified alternate etiology, should be treated according to the guidelines in Table 7.. Withhold treatment with atezolizumab for Grade 1 pericarditis and conduct a detailed cardiac evaluation to determine the etiology and manage accordingly.

Cardiac tamponade should be treated as a medical emergency and consultation with a cardiologist should be sought for further management.

Caution should be used when considering the use of atezolizumab in a patient who has previously experienced a pericardial disorder on prior treatment with other immune-stimulatory anticancer agents.

Table 7: Management Guidelines for Immune-Mediated cardiac Events

Event	Management
Immune-mediated myocarditis, Grade 2-4<br>Or<br>Immune-mediated pericardial disorders, Grade 2-4	• Permanently discontinue atezolizumab and contact Medical Monitor.<br>• Refer patient to cardiologist.<br>• Initiate treatment as per institutional guidelines and consider anti-arrhythmic drugs, temporary pacemaker, ECMO, VAD or pericardiocentesis as appropriate.<br>• Initiate treatment with corticosteroids equivalent to 1g/day IV methylprednisolone for 3-5 days and convert to 1–2 mg/kg/day oral prednisone or equivalent upon improvement.<br>• If event does not improve within 24 hours after initiating corticosteroids, consider adding an immunosuppressive agent.<br>• If event resolves to Grade 1 or better, taper corticosteroids over ≥ 1 month.

ECMO = extracorporeal membrane oxygenation; VAD = ventricular assist device

[a] .

5.3.2.7 Infusion related reaction (IRR) and Cytokine-Release syndrome (CRS)

No premedication is indicated for administration of atezolizumab in cycle 1.

Patients who experience an IRR or CRS with atezolizumab may receive premedication with antihistamines or antipyretics/analgesics (e.g. paracetamol) for subsequent infusions.

Metamizole (dipyrone) is prohibited in treating atezolizumab associated infusion-related reactions, due to its potential for causing agranulocytosis.

IRRs are known to occur with the administration of monoclonal antibodies and have been reported with atezolizumab. These reactions, which are thought to be due to release of cytokines and/or other chemical mediators, occur within 24 hours of atezolizumab administration and are generally mild to moderate in severity.

CRS is defined as a supraphysiologic response following administration of any immune therapy that results in activation or engagement of endogenous or infused T cells and/or other immune effector cells. Symptoms can be progressive, always include fever at the onset, and may include hypotension, capillary leak (hypoxia), and end-organ dysfunction (Lee et al. 2019). CRS has been well documented with chimeric antigen receptor T-cell therapies and bispecific T cell-engager antibody therapies but has also been reported with immunotherapies that target PD-L1 or PD-1 (Rotz et al. 2017; Adashek and Feldman 2019), including atezolizumab.

There may be significant overlap in signs and symptoms of IRRs and CRS, and in recognition of the challenges in clinically distinguishing between the two, consolidated guidelines for the medical management of IRRs and CRS are provided in Table 8.

Severe SARS-COV-2 infection appears to be associated with a CRS involving the inflammatory cytokines interleukin (IL)-6, IL-10, IL-2, and interferon-□ (Merad and Martin

2020). If a patient develops suspected CRS during the study, a differential diagnosis should include COVID-19, which should be confirmed or refuted through assessment of exposure history, appropriate laboratory testing, and clinical or radiologic evaluations per the investigator's judgment. If a diagnosis of COVID-19 is confirmed, the disease should be managed as per local or institutional guidelines.

Table 8: Management Guidelines for Infusion-Related Reactions and Cytokine-Release Syndrome

Event	Management
Grade 1 [a]<br>Fever [b] with or without constitutional symptoms	• Immediately interrupt infusion.<br>• Upon symptom resolution, wait for 30 minutes and then restart infusion at half the rate being given at the time of event onset.<br>• If the infusion is tolerated at the reduced rate for 30 minutes, the infusion rate may be increased to the original rate.<br>• If symptoms recur, discontinue infusion of this dose.<br>• Administer symptomatic treatment,[c] including maintenance of IV fluids for hydration.<br>• In case of rapid decline or prolonged CRS (> 2 days) or in patients with significant symptoms and/or comorbidities, consider managing as per Grade 2.<br>• For subsequent infusions, consider administration of oral premedication with antihistamines, anti-pyretics, and/or analgesics, and monitor closely for IRRs and/or CRS.
Grade 2 [a]<br>Fever [b] with at least one of the following:<br>• Hypotension not requiring vasopressors<br>• Hypoxia requiring low-flow oxygen [d] by nasal cannula or blow-by	• Immediately interrupt infusion.<br>• Upon symptom resolution, wait for 30 minutes and then restart infusion at half the rate being given at the time of event onset.<br>• If symptoms recur, discontinue infusion of this dose.<br>• Administer symptomatic treatment. [c]<br>• For hypotension, administer IV fluid bolus as needed.<br>• Monitor cardiopulmonary and other organ function closely (in the ICU, if appropriate). Administer IV fluids as clinically indicated and manage constitutional symptoms and organ toxicities as per institutional practice.<br>• Rule out other inflammatory conditions that can mimic CRS (e.g., sepsis). If no improvement within 24 hours, initiate workup and assess for signs and symptoms of HLH or MAS as described in this appendix.<br>• Consider IV corticosteroids (e.g., methylprednisolone 2 mg/kg/day or dexamethasone 10 mg every 6 hours).<br>• Consider anti-cytokine therapy.<br>• Consider hospitalization until complete resolution of symptoms. If no improvement within 24 hours, manage as per Grade 3, that is, hospitalize patient (monitoring in the ICU is recommended), permanently discontinue atezolizumab, and contact Medical Monitor.<br>• If symptoms resolve to Grade 1 or better for 3 consecutive days, the next dose of atezolizumab may be administered. For subsequent infusions, consider administration of oral premedication with antihistamines, anti-pyretics, and/or analgesics and monitor closely for IRRs and CRS.<br>• If symptoms do not resolve to Grade 1 or better for 3 consecutive days, contact Medical Monitor.

Event	Management
Grade 3 [a]<br><br>Fever [b] with at least one of the following:<br>• Hypotension requiring a vasopressor (with or without vasopressin)<br>• Hypoxia requiring high-flow oxygen [d] by nasal cannula, face mask, non-rebreather mask, or Venturi mask	• Permanently discontinue atezolizumab and contact Medical Monitor. [e]<br>• Administer symptomatic treatment. [c]<br>• For hypotension, administer IV fluid bolus and vasopressor as needed.<br>• Monitor cardiopulmonary and other organ function closely; monitoring in the ICU is recommended. Administer IV fluids as clinically indicated and manage constitutional symptoms and organ toxicities as per institutional practice.<br>• Rule out other inflammatory conditions that can mimic CRS (e.g., sepsis). If no improvement within 24 hours, initiate workup and assess for signs and symptoms of HLH or MAS..<br>• Administer IV corticosteroids (e.g., methylprednisolone 2 mg/kg/day or dexamethasone 10 mg every 6 hours).<br>• Consider anti-cytokine therapy.<br>• Hospitalize patient until complete resolution of symptoms. If no improvement within 24 hours, manage as per Grade 4, that is, admit patient to ICU and initiate hemodynamic monitoring, mechanical ventilation, and/or IV fluids and vasopressors as needed; for patients who are refractory to anti-cytokine therapy, experimental treatments may be considered at the discretion of the investigator and in consultation with the principal investigator
Grade 4 [a]<br><br>Fever [b] with at least one of the following:<br>• Hypotension requiring multiple vasopressors (excluding vasopressin)<br>• Hypoxia requiring oxygen by positive pressure (e.g., CPAP, BiPAP, intubation and mechanical ventilation)	• Permanently discontinue atezolizumab and contact principal investigator. [e]<br>• Administer symptomatic treatment. [c]<br>• Admit patient to ICU and initiate hemodynamic monitoring, mechanical ventilation, and/or IV fluids and vasopressors as needed. Monitor other organ function closely. Manage constitutional symptoms and organ toxicities as per institutional practice.<br>• Rule out other inflammatory conditions that can mimic CRS (e.g., sepsis). If no improvement within 24 hours, initiate workup and assess for signs and symptoms of HLH or MAS as described in this appendix.<br>• Administer IV corticosteroids (e.g., methylprednisolone 2 mg/kg/day or dexamethasone 10 mg every 6 hours).<br>• Consider anti-cytokine therapy. For patients who are refractory to anti-cytokine therapy, experimental treatments [f] may be considered at the discretion of the investigator and in consultation with the Medical Monitor.<br>• Hospitalize patient until complete resolution of symptoms.

ASTCT = American Society for Transplantation and Cellular Therapy; BiPAP = bi-level positive airway pressure; CAR = chimeric antigen receptor; CPAP = continuous positive airway pressure; CRS = cytokine-release syndrome; CTCAE = Common Terminology Criteria for Adverse Events; eCRF = electronic Case Report Form; HLH = hemophagocytic lymphohistiocytosis; ICU = intensive care unit; MAS = macrophage activation syndrome; NCCN = National Cancer Comprehensive Network; NCI = National Cancer Institute.

The management guidelines have been adapted from NCCN guidelines for management of CAR T-cell–related toxicities (Version 2.2019).

[a] Grading system for management guidelines is based on the ASTCT CRS consensus grading scale. NCI CTCAE V5 and the ASTCT CRS consensus grading scale should be used when reporting severity of CRS on the Adverse Event eCRF. NCI CTCAE V5 should be used when reporting severity of organ toxicities associated with CRS on the dedicated Cytokine Release Syndrome eCRF. Organ toxicities associated with CRS should not influence overall CRS grading.

[b] Fever is defined as temperature ≥38°C not attributable to any other cause. In patients who develop CRS and then receive anti-pyretic, anti-cytokine, or corticosteroid therapy, fever is no longer required when subsequently determining event severity (grade). In this case, the grade is driven by the presence of hypotension and/or hypoxia.

[c] Symptomatic treatment may include oral or IV antihistamines, anti-pyretics, analgesics, bronchodilators, and/or oxygen. For bronchospasm, urticaria, or dyspnea, additional treatment may be administered as per institutional practice.

[d] Low flow is defined as oxygen delivered at ≤ 6 L/min, and high flow is defined as oxygen delivered at > 6 L/min.

[e] Resumption of atezolizumab may be considered in patients who are deriving benefit and have fully recovered from the event. The decision to re-challenge patients with atezolizumab should be based on investigator's assessment of benefit–risk and documented by the investigator (or an appropriate delegate). For subsequent infusions, administer oral premedication with antihistamines, anti-pyretics, and/or analgesics, and monitor closely for CRS. Premedication with corticosteroids and extending the infusion time may also be considered after assessing the benefit–risk ratio.

[f] Refer to Riegler et al. (2019)

5.3.2.8 Pancreatic events

Symptoms of abdominal pain associated with elevations of amylase and lipase, suggestive of pancreatitis, have been associated with the administration of atezolizumab. The differential diagnosis of acute abdominal pain should include pancreatitis. Appropriate work-up should include an evaluation for ductal obstruction, as well as serum amylase and lipase tests.

Table 7: Management guidelines for Pancreatic events including pancreatitis

Severity	Management
	•
Grade 2 amylase/lipase elevation	Amylase and/or lipase >1.5-2.0xULN:<br>• Continue atezolizumab.<br>• Monitor amylase and lipase weekly.<br>• For prolonged elevation (e.g., >3 weeks), consider treatment with 10 mg/day oral prednisone or equivalent.<br><br>Asymptomatic with amylase and/or lipase >2.0-5.0xULN:<br>Treat as grade 3<br>•
Grades 3 and 4 amylase/lipase elevation	• Withhold atezolizumab for up to 12 weeks. [a]<br>• Refer patient to gastrointestinal specialist.<br>• Monitor amylase/lipase every other day, and consider oral prednisone 1-2 mg/kg/day or equivalent if no improvement.<br>• Atezolizumab may be resumed if the event resolves to Grade 0 or Grade 1

	within 12 weeks [b]<br>• Permanently discontinue atezolizumab and contact the principal investigator if event does not resolve to Grade 1 or better within 12 weeks. [c]<br>• Permanently discontinue atezolizumab for recurrent Grade 3 or 4 amylase/lipase elevations and contact the principal investigator [c]
Immune-related pancreatitis, Grade 2 or 3	• Withhold atezolizumab for up to 12 weeks after event onset.[a]<br>• Refer patient to gastrointestinal specialist.<br>• Initiate treatment with 1−2 mg/kg/day intravenous methylprednisolone or equivalent and convert to 1−2 mg/kg/day oral prednisone or equivalent upon improvement.<br>• Resume atezolizumab if event resolves to Grade 1 or better within 12 weeks. [b]<br>• Permanently discontinue atezolizumab and contact the Medical Monitor if event does not resolve to Grade 1 or better within 12 weeks. [c]<br>• For recurrent events, permanently discontinue atezolizumab and contact the Medical Monitor. [c]
Immune-related pancreatitis, Grade 4	• Permanently discontinue atezolizumab and contact the principal investigator [c]<br>• Refer patient to gastrointestinal specialist.<br>• Initiate treatment with 1−2 mg/kg/day intravenous methylprednisolone or equivalent and convert to 1−2 mg/kg/day oral prednisone or equivalent upon improvement.<br>• If event does not improve within 48 hours after initiating corticosteroids, consider adding an immunosuppressive agent.<br>• If event resolves to Grade 1 or better, taper corticosteroids over ≥ 1 month.

IV = intravenous; PO = orally.

a Atezolizumab may be withheld for a period of time beyond 12 weeks to allow for corticosteroids to be reduced to ≤ 10 mg/day oral prednisone or equivalent. The acceptable length of the extended period of time must be based on the investigator benefit-risk assessment and in alignment with the protocol requirement fir duration of treatment and documented by the investigator. The Medical Monitor is available to advise as needed

b If corticosteroids have been initiated, they must be tapered over ≥ 1 month to ≤ 10 mg/day oral prednisone or equivalent before atezolizumab can be resumed.

.

c Resumption of atezolizumab may be considered in patients who are deriving benefit and have fully recovered from the immune-related event. Patients can be rechallenged with atezolizumab only after approval has been documented by both the investigator (or an appropriate delegate) and the sponsor.

5.3.2.9 Dermatologic Events

The majority of cases of rash reported with the use of atezolizumab were mild in severity and self-limited, with or without pruritus. Although uncommon, cases of severe cutaneous adverse reactions such as Stevens-Johnson syndrome and toxic epidermal necrolysis have been reported with atezolizumab. A dermatologist should evaluate persistent and/or severe rash or pruritus. A biopsy should be considered unless contraindicated.

Table 8: Management Guidelines for Dermatologic Events

Severity	Management
**Grade 1**	• Continue atezolizumab.<br>• Consider topical steroids and/or other symptomatic therapy (e.g., antihistamines).
**Grade 2**	• Continue atezolizumab.<br>• Consider dermatologist referral.<br>• Administer topical corticosteroids.<br>• Consider higher potency topical corticosteroids if event does not improve.<br>• If unresponsive to topical corticosteroids, consider oral prednisone 0.5 mg/kg/day.
**Grade 3**	• Withhold atezolizumab.<br>• Refer patient to dermatologist.<br>• Initiate treatment with 10 mg/day oral prednisone or equivalent, increasing dose to 1−2 mg/kg/day if event does not improve within 48−72 hours.<br>• Restart atezolizumab if event resolved to Grade 1 or better within 12 weeks. [a, b, c]<br>• Permanently discontinue atezolizumab and contact the principal investigator if event does not resolve to Grade 1 or better within 12 weeks.[a, b, c]
**Grade 4**	• Permanently discontinue atezolizumab.and Contact the Medical Monitor [c]
Stevens-Johnson syndrome or toxic epidermal necrolysis (any grade)	**Additional guidance for Stevens-Johnson syndrome or toxic epidermal necrolysis:**<br>• Withhold atezolizumab or atezolizumab-tiragolumab for suspected Stevens-Johnson syndrome or toxic epidermal necrolysis.<br>• Confirm diagnosis by referring patient to a specialist (dermatologist, ophthalmologist, or urologist as relevant) for evaluation and, if indicated, biopsy.<br>• Follow the applicable treatment and management guidelines above.<br>• If Stevens-Johnson syndrome or toxic epidermal necrolysis is confirmed, permanently discontinue atezolizumab or atezolizumab-tiragolumab.<br>•

[a] If corticosteroids have been initiated, they must be tapered over ≥1 month to ≤10 mg/day oral prednisone or equivalent before atezolizumab can be resumed.

[b] Atezolizumab may be withheld for a period of time beyond 12 weeks to allow for corticosteroids to be reduced to ≤10 mg/day oral prednisone or equivalent. The acceptable length of the extended period of time must be agreed upon by the investigator and the Medical Monitor.

[c] Resumption of atezolizumab may be considered in patients who are deriving benefit and have fully recovered from the immune-related event. Patients can be rechallenged with atezolizumab only after approval has been documented by both the investigator (or an appropriate delegate) and the principal investigator.

Severe Cutaneous Adverse Reactions (SCARs), including SJS, TEN, acute generalized exanthematous pustulosis and DRESS/DIHS
General instructions:

1. Discontinue treatment and monitor closely for improvement, regardless of grade.

2. Rule out any other etiology of the skin problem, such as an infection, an effect of another drug, or a skin condition linked to another systemic disease.

3. Biologic checkup, including a CBC with differential test, and liver and kidney function tests, including urinalysis, in addition to the blood work; if the patient is febrile, blood cultures should be considered as well.

4. Skin biopsies to assess for full-thickness epidermal necrosis, as is seen in SJS/TEN, as well as other possible etiologies like paraneoplastic pemphigus or other autoimmune blistering dermatoses or other drug reactions, such as acute generalized exanthematous pusulosis.

5. Consider following patients closely using serial clinical photography.

6. If mucous membrane involvement or blistering is observed on the skin, consider early admission to a burn center for further monitoring and management.

7. Review of systems: Skin pain (like a sunburn), fevers, malaise, myalgias, arthralgias, abdominal pain, ocular discomfort or photophobia, sores or discomfort in the nares, sores or discomfort in the oropharynx, odynophagia, hoarseness, dysuria, sores or discomfort in the vaginal area for women or involving the meatus of the penis for men, sores in the perianal area, or pain with bowel movements.

8. Physical examination: Include vital signs and a full skin examination specifically evaluating all skin surfaces and mucous membranes. Assess for lymphadenopathy, facial or distal extremity swelling (may be signs of DIHS/DRESS). Assess for pustules, blisters or erosions in addition to areas of “dusky erythema,” which may feel painful to palpation. To assess for a positive Nikolsky sign, place a gloved finger tangentially over erythematous skin and apply friction parallel to the skin surface. Nikolsky sign is positive if this results in detached or sloughing epidermis demonstrating poor attachment of the epidermis to the dermis, which is the case in some autoimmune disorders (eg, pemphigus) and SJS/TEN.

9. Additional considerations: The usual prohibition of corticosteroids for SJS is not relevant here, as the underlying mechanism is a T-cell immunodirected toxicity. Adequate suppression is necessary with corticosteroids or other agents and may be prolonged in cases of DRESS/DIHS.

**Grade 1**		Not applicable. For SCARs, there is no G1 category; if lower BSA is involved with bullae or erosions, there should remain a high concern that this reaction will progress to G3 or G4.	
**Other grades**	**Action taken on suspected IMP**	**Management with corticosteroids and/or other therapies**	**Monitor and follow up**
**Grade 2:** Morbilliform “maculopapular”) exanthem covering 10%-30% BSA with systemic symptoms, lymphadenopathy, or facial swelling	Withhold atezolizumab	• Initiate therapy with topical emollients, oral antihistamines, and medium- to highstrength topical corticosteroids<br>• Consider initiation of prednisone (or equivalent) 0.5-1 mg/kg tapered over at least 4 weeks	• Consider to refer patient to dermatologist<br>• Monitor patients closely every 3 days for progression to involvement of greater BSA and/or mucous membrane involvement.<br>• Consider following patients closely using serial photography<br>• Investigators are encouraged to contact experts in the management of immune toxicities of the ImmunoTOX network using the contacts details below.

			• Patient can be rechallenged only after the sponsor and coordinating investigator approval
**Grade 3:**<br>Skin sloughing covering , 10% BSA with mucosal involvement associated signs (eg, erythema, purpura, epidermal detachment, mucous membrane detachment)	Withhold atezolizumab *	• Treat skin with topical emollients and other petrolatum emollients, oral antihistamines, and high-strength topical corticosteroids; dimethicone may also be offered as an alternative to petrolatum<br>• Administer IV prednisolone (or equivalent) 0.5-1 mg/kg and convert to oral corticosteroids on response, wean over at least 4 weeks.<br>• Given the immune mechanism of action of Atezolizumab, use of immune suppression is warranted.<br>• Admit to burn and/or consult wound services with attention to supportive care, including fluid and electrolyte balance, minimizing insensible water losses, and preventing infection	• Refer patient to dermatologist<br>• For mucous membrane involvement of SJS or TEN, appropriate consulting services should be offered to guide management in preventing sequelae from scarring (eg, ophthalmology; ear, nose, and throat; urology; gynecology; etc, as appropriate).<br>• Seek infectious disease consultation if patient might have secondary cellulitis or if patient has other infection risk factors, such as neutropenia<br>• Investigators are encouraged to contact experts in the management of immune toxicities of the ImmunoTOX network using the following contacts :<br><br>**Urgent advice**: Contact directly the referent specialist of the ImmunoTOX network, Pr C.Robert caroline.robert@gustaveroussy.fr<br><br>**Half-urgent:**<br>Send email to the immunoTOX recommendation panel: rcp.itox@gustaveroussy.fr and/or contact Mme Janine NDA<br>Tel: +33 (0)1 42 11 43 31<br>• Patient can be rechallenged only after the sponsor and coordinating investigator approval

**Grade 4:**<br>Skin erythema and blistering/sloughing covering ≥ 10% BSA with associated signs (eg, erythema, purpura, epidermal detachment, mucous membrane detachment) and/or systemic symptoms and concerning associated blood work abnormalities (eg, liver function test elevations in the setting of DRESS/DIHS)	Permanently discontinue atezoluzumab	• Initiate IV (methyl)prednisolone (or equivalent) 1-2 mg/kg, tapering when toxicity resolves to normal<br>• IVIG or cyclosporine may also be considered in severe or corticosteroid unresponsive cases.	• Admit patient immediately to a burn unit or ICU with consulted dermatology and wound care services.<br>• Refer patient to dermatologist<br>• Consider further consultations based on management of mucosal surfaces (eg, ophthalmology; urology; gynecology; ear, nose, and throat surgery; etc).<br>• Consider pain/palliative consultation and/or admission in patients presenting with DRESS manifestations.<br>• Seek infectious disease consultation if patient might have secondary cellulitis or if patient has other infection risk factors, such as neutropenia<br>• Investigators are encouraged to contact experts in the management of immune toxicities of the ImmunoTOX network using the contacts details above

BSA = body surface area; CTCAE = Common Terminology Criteria for Adverse Events; DIHS = drug-induced hypersensitivity syndrome; DRESS = drug reaction with eosinophilia and systemic symptoms; G = grade; ICU = intensive care unit; irAE = immune-related adverse event; IV = intravenous; IVIG = intravenous immunoglobulin; NA = not applicable; SCAR = severe cutaneous adverse reactions; SJS = Stevens-Johnson syndrome; TENS = toxic epidermal necrolysis.

* **For confirmed Stevens Johnson syndrome or toxic epidermal necrolysis**, permanently discontinue atezolizumab. For other cases, resumption of Atezolizumab may be considered in patients who are deriving benefit and have fully recovered from the SCARs. **Patients can be rechallenged only after approval has been documented by both the coordinating investigator and the referent specialist of the Immunotox network**.

5.3.2.10 Neurologic Disorders

Myasthenia gravis and Guillain-Barre syndrome have been observed with single agent atezolizumab.

Patients may present with signs and symptoms of sensory and/or motor neuropathy. Diagnostic work-up is essential for an accurate characterization todifferentiate between alternate etiologies. Myasthenia may be associated with myositis and patients should be managed accordingly. Management guidelines for neurologic disorders are provided in Table 11., with specific guidelines for myelitis provided in Table 12.

Table 9: Management Guidelines for Neurologic Disorder

Neurologic Disorder	Management
Grade 1 Immune-related Neuropathy,	• Continue atezolizumab.<br>• Evaluate for alternate causes.<br>• Any cranial nerve disorder (including facial paresis) should be managed as per Grade 2 management guidelines below
Grade 2 Immune-related Neuropathy, including facial paresis	• Withhold atezolizumab for up to 12 weeks after event onset.<br>• Investigate etiology and refer patient to a neurologist.<br>• Initiate treatment as per institutional guidelines.<br>• For general immune-mediated neuropathy<br>○ If event resolves to Grade 1 or better, resume atezolizumab. b<br>○ If event does not resolve to Grade 1 or better while withholding atezolizumab, permanently discontinue atezolizumab and contact the Medical Monitor. c<br>• For facial paresis:<br>○ Initial observation OR initiate prednisone 1-2 mg/kg/day (if progressing from mild). Initiate treatment with gabapentin, pregabalin, or duloxetine for pain.<br>○ If event resolves fully, resume atezolizumab. b<br>○ If event does not resolve fully while withholding atezolizumab, permanently discontinue atezolizumab and contact the Medical Monitor. c<br>•
Grade 3 or 4 Immune-related Neuropathy, including facial paresis	• Permanently discontinue atezolizumab and the principal investigator [c]<br>• Refer patient to a neurologist<br>• Treatment should be as per institutional guidelines and proceed as per Guillain-Barré syndrome management.

Myasthenia Gravis and Guillain-Barré syndrome (any grade)	• Permanently discontinue atezolizumab.and the principal investigator [c]<br>• Refer patient to neurologist<br>• Initiate treatment as per institutional guidelines.<br>• Consider initiation of corticosteroids 1-2 mg/kg/day oral or intravenous prednisone<br>• Consider IVIG or plasmapheresis in patients with rapid progression with development of bulbar and/or respiratory symptoms.<br>• In life-threatening cases, consider IV methylprednisone 1 g/day for 3-5 days and consider other immunosuppressive agent. a

a If corticosteroids have been initiated, they must be tapered over ≥ 1 month to ≤ 10 mg/day oral prednisone or equivalent before atezolizumab can be resumed.
b Atezolizumab may be withheld for a period of time beyond 12 weeks to allow for corticosteroids to be reduced to ≤ 10 mg/day oral prednisone or equivalent. The acceptable length of the extended period of time must be agreed upon by the investigator and the Medical Monitor.

c Resumption of atezolizumab may be considered in patients who are deriving benefit and have fully recovered from the immune-related event. Patients can be rechallenged with atezolizumab only after approval has been documented by both the investigator (or an appropriate delegate) and the principal investigator

Table 12 Management Guidelines for Immune-Mediated Myelitis

Event	Management
Immune-mediated myelitis, Grade 1	• Continue atezolizumab unless symptoms worsen or do not improve.<br>• Investigate etiology and refer patient to neurologist.
Immune-mediated myelitis, Grade 2	• Permanently discontinue atezolizumab and contact the Medical Monitor.<br>• Investigate etiology and refer patient to neurologist.<br>• Rule out infection.<br>• Initiate treatment with corticosteroids equivalent to 1□2 mg/kg/day oral prednisone.
Immune-mediated myelitis, Grade 3 and 4	• Permanently discontinue atezolizumab and contact the Medical Monitor.<br>• Initiate non-opioid treatment (e.g., pregabalin, gabapentin, duloxetine) for pain.<br>• Hospitalize patient.<br>o Initiate treatment with corticosteroids equivalent to 1 g/day IV methylprednisolone.<br>o if event does not improve or there is worsening of symptoms within 3 days, consider IVIG or plasmapheresis and manage as per institutional guidelines.<br>• Refer patient to a neurologist.

IVIG : intravenous immunoglobulin.

5.3.2.11 Immune-Mediated Meningoencephalitis

Immune-related meningoencephalitis is an identified risk associated with the administration of atezolizumab.

Immune-related meningoencephalitis should be suspected in any patient presenting with signs or symptoms suggestive of meningitis or encephalitis, including, but not limited to, headache, neck pain, confusion, seizure, motor or sensory dysfunction, and altered or depressed level of consciousness. Encephalopathy from metabolic or electrolyte imbalances needs to be

distinguished from potential meningoencephalitis resulting from infection (bacterial, viral, or fungal) or progression of malignancy, or secondary to a paraneoplastic process.

All patients being considered for meningoencephalitis should be urgently evaluated with a CT scan and/or MRI scan of the brain to evaluate for metastasis, inflammation, or edema. If deemed safe by the treating physician, a lumbar puncture should be performed and a neurologist should be consulted.

Patients with signs and symptoms of meningoencephalitis, in the absence of an identified alternate etiology, should be treated as defined below.

Table13: Management Guidelines for Immune-Related Meningoencephalitis

Event	Management
Immune-related meningoencephalitis, all grades	• Permanently discontinue atezolizumab and contact the principal investigator.<br>• Refer patient to neurologist.<br>• Initiate treatment with 1−2 mg/kg/day IV methylprednisolone or equivalent and convert to 1−2 mg/kg/day oral prednisone or equivalent upon improvement.<br>• If event does not improve within 48 hours after initiating corticosteroids, consider adding an immunosuppressive agent.<br>• If event resolves to Grade 1 or better, taper corticosteroids over ≥ 1 month.

IV = intravenous.

5.3.2.12 Renal Events

Immune-related nephritis has been associated with the administration of atezolizumab.

Eligible patients must have adequate renal function, and renal function, including serum creatinine, should be monitored throughout study treatment. Patients with abnormal renal function should be evaluated and treated for other more common etiologies (including prerenal and postrenal causes, and concomitant medications such as nonsteroidal anti-inflammatory drugs). Refer the patient to a renal specialist if clinically indicated. A renal biopsy may be required to enable a definitive diagnosis and appropriate treatment. If no alternative cause of acute kidney injury is identified, patients with signs and symptoms of acute kidney injury, in the absence of an identified alternate etiology, should be treated according to the management guidelines for immune-related renal events in the table below.

Patients with signs and symptoms of nephritis, in the absence of an identified alternate etiology, should be treated according to the guidelines in table 14.

Table 14: Management guideline for renal events

**Severity**	**Management**
**Grade 1**	• Continue atezolizumab.<br>• Monitor kidney function, including creatinine, closely until values resolve to normal limits or to baseline values.
**Grade 2**	• Withhold atezolizumab for up to 12 weeks after event onset. [a]

	• Refer patient to renal specialist.<br>• Initiate treatment with corticosteroids equivalent to 1-2 mg/kg/day oral prednisone.<br>• If event resolves to Grade 1 or better, resume atezolizumab. [b]<br>• If event does not resolve to Grade 1 or better while withholding atezolizumab, permanently discontinue atezolizumab and contact the principal investigator. [c]
**Grade 3-4**	• Permanently discontinue atezolizumab and contact the principal investigator.<br>• Refer patient to renal specialist and consider renal biopsy.<br>• Initiate treatment with corticosteroids equivalent to 1-2 mg/kg/day oral prednisone.<br>• If event does not improve within 48 hours after initiating corticosteroids, consider adding an immunosuppressive agent.<br>• If event resolves to Grade 1 or better, taper corticosteroids over >1 month

a Atezolizumab may be withheld for a longer period of time (i.e., > 12 weeks after event onset) to allow for corticosteroids (if initiated) to be reduced to the equivalent of ≤ 10 mg/day oral prednisone. The acceptable length of the extended period of time must be agreed upon by the investigator and the Medical Monitor.
b If corticosteroids have been initiated, they must be tapered over ≥ 1 month to the equivalent of ≤ 10 mg/day oral prednisone before atezolizumab can be resumed.
c Resumption of atezolizumab may be considered in patients who are deriving benefit and have fully recovered from the immune-related event. Patients can be re-challenged with atezolizumab only after approval has been documented by both the investigator (or an appropriate delegate) and the sponsor.

5.3.2.13 Immune-Mediated Myositis

Immune-mediated myositis has been associated with the administration of atezolizumab. Myositis or inflammatory myopathies are a group of disorders sharing the common feature of inflammatory muscle injury; dermatomyositis and polymyositis are among the most common disorders. Initial diagnosis is based on clinical (muscle weakness, muscle pain, skin rash in dermatomyositis), biochemical (serum creatine kinase/ creatine phosphokinase increase), and imaging (electromyography/MRI) features, and is confirmed with a muscle biopsy. ¨Patients mayinitially present with low grade nondescript symptoms including mild pain and weakness; thus,there should be a low threshold for suspicion of myositis.

Patients with possible myositis should be referred to a rheumatologist or neurologist. Patients with possible myositis should be monitored for signs of myocarditis and myasthenia gravis (bulbar symptoms such as dysphagia, dysphonia and dyspnea).

Patients with signs and symptoms of myositis, in the absence of an identified alternate etiology, should be treated according to the guidelines in Table 14.

Table 4 Management Guidelines for Immune-Mediated Myositis

Event	Management
Immune-mediated myositis, Grade 1	• Continue atezolizumab.<br>• Refer patient to rheumatologist or neurologist.<br>• Initiate treatment as per institutional guidelines.

Immune-mediated myositis, Grade 2	• Withhold atezolizumab for up to 12 weeks after event onset [a] and contact the principal investigator.<br>• Refer patient to rheumatologist or neurologist.<br>• Initiate treatment as per institutional guidelines.<br>• Consider treatment with corticosteroids equivalent to 1–2 mg/kg/day IV methylprednisolone and convert to 1–2 mg/kg/day oral prednisone or equivalent upon improvement.<br>• If corticosteroids are initiated and event does not improve within 48 hours after initiating corticosteroids, consider adding an immunosuppressive agent.<br>• If event resolves to Grade 1 or better, resume atezolizumab. [b]<br>• If event does not resolve to Grade 1 or better while withholding atezolizumab, permanently discontinue atezolizumab and contact the principal investigator. [c]
Immune-mediated myositis, Grade 3	• Withhold atezolizumab for up to 12 weeks after event onset [a] and contact the principal investigator.<br>• Refer patient to rheumatologist or neurologist.<br>• Initiate treatment as per institutional guidelines. Respiratory support may be required in more severe cases.<br>• Initiate treatment with corticosteroids equivalent to 1–2 mg/kg/day IV methylprednisolone, or higher-dose bolus if patient is severely compromised (e.g., cardiac or respiratory symptoms, dysphagia, or weakness that severely limits mobility); convert to 1–2 mg/kg/day oral prednisone or equivalent upon improvement.<br>• Consider IVIG or plasmapheresis<br>• If event does not improve within 24 hours after initiating corticosteroids, consider adding an immunosuppressive agent.<br>• If event resolves to Grade 1 or better, resume atezolizumab. [b]<br>• If event does not resolve to Grade 1 or better while withholding atezolizumab, permanently discontinue atezolizumab and contact Medical Monitor. [c]<br>• For recurrent events, treat as a Grade 4 event.
Immune-mediated myositis, Grade 4	• Permanently discontinue atezolizumab and contact the principal investigator. [c]<br>• Refer patient to rheumatologist or neurologist.<br>• Initiate treatment as per institutional guidelines. Respiratory support may be required in more severe cases.<br>• Initiate treatment with corticosteroids equivalent to 1–2 mg/kg/day IV methylprednisolone, or higher-dose bolus if patient is severely compromised (e.g., cardiac or respiratory symptoms, dysphagia, or weakness that severely limits mobility); convert to 1–2 mg/kg/day oral prednisone or equivalent upon improvement.<br>• Consider IVIG or plasmapheresis<br>• If event does not improve within 24 hours after initiating corticosteroids, consider adding an immunosuppressive agent.

	• If event resolves to Grade 1 or better, taper corticosteroids over ≥ 1 month.

IVIG : intravenous immunoglobulin.

[a] Atezolizumab may be withheld for a longer period of time (i.e., > 12 weeks after event onset) to allow for corticosteroids (if initiated) to be reduced to the equivalent of ≤ 10 mg/day oral prednisone. The acceptable length of the extended period of time must be agreed upon by the investigator and the sponsor.

[b] If corticosteroids have been initiated, they must be tapered over ≥ 1 month to the equivalent of ≤ 10 mg/day oral prednisone before atezolizumab can be resumed.

[c] Resumption of atezolizumab may be considered in patients who are deriving benefit and have fully recovered from the immune-related event. Patients can be re-challenged with atezolizumab only after approval has been documented by both the investigator (or an appropriate delegate) and the sponsor.

5.3.2.14 Hemophagocytic Lymphohistiocytosis

Immune-mediated reactions may involve any organ system and may lead to hemophagocytic lymphohistiocytosis (HLH).

Clinical and laboratory features of severe CRS overlap with HLH, and HLH should be considered when CRS presentation is atypical or prolonged.

Patients with suspected HLH should be diagnosed according to published criteria by McClain and Eckstein (2014). A patient should be classified as having HLH if five of the following eight criteria are met:

- Fever ≥38.5°C
- Splenomegaly
- Peripheral blood cytopenia consisting of at least two of the following: Hemoglobin < 90 g/L (9 g/dL) (< 100 g/L [10 g/dL] for infants < 4 weeks old) Platelet count < 100×10^9/L (100,000/μL)
ANC <1.0×10^9/L(1000/μL)
- Fasting triglycerides > 2.992 mmol/L (265 mg/dL) and/or fibrinogen < 1.5 g/L (150 mg/dL)
- Hemophagocytosis in bone marrow, spleen, lymph node, or liver
- Low or absent natural killer cell activity
- Ferritin > 500 mg/L (500 ng/mL)
- Soluble interleukin 2 (IL-2) receptor (soluble CD25) elevated ≥ 2 standard deviations above age-adjusted laboratory-specific norms

Patients with suspected HLH should be treated according to the guidelines in Table 16.

Table15: Management Guidelines for Suspected Hemophagocytic Lymphohistiocytosis

Event	Management
Suspected HLH	• Permanently discontinue atezolizumab and contact the Medical Monitor.<br>• Consider patient referral to hematologist.<br>• Initiate supportive care, including intensive care monitoring if indicated per institutional guidelines.<br>• Consider initiation of IV corticosteroids, an immunosuppressive agent, and/or anti-cytokine therapy.<br>• If event does not respond to treatment within 24 hours, contact the Medical Monitor and initiate treatment as appropriate according to published guidelines (La Rosée 2015; Schram and Berliner 2015; La Rosée et al. 2019).

	- If event resolves to Grade 1 or better, taper corticosteroids over 1 month.

5.3.2.15 Systemic Immune Activation

Systemic immune activation is a rare condition characterized by an excessive immune response. Given the mechanism of action of atezolizumab, systemic immune activation is considered a potential risk for atezolizumab.

Recommendations regarding early identification and management of systemic immune activation are provided below. In the event of suspected systemic immune activation, atezolizumab should be withheld and clinical specialists (e.g., rheumatology, clinical immunology, or solid organ or hematopoietic stem cell transplant specialists) and the Medical Monitor should be consulted for additional guidance.

Early disease recognition is critical, and systemic immune activation should be suspected if, in the absence of an alternative etiology, the patient meets two or more of the following criteria:

- Hypotension that is refractory to aggressive IV fluid challenge

  Vasopressor support may be required.
- Respiratory distress that requires aggressive supportive care

  Supplemental oxygen and intubation may be required.
- Fever $>$38.5°C
- Acute renal or hepatic failure
- Bleeding from coagulopathy
- Any of the following unexplained laboratory abnormalities (change from baseline): cytopenias (in two or more lineages), significant transaminitis, or coagulopathy

For patients with suspected systemic immune activation, an initial evaluation should include the following:

- CBC with peripheral smear
- PT, PTT, fibrinogen, and D-dimer
- Ferritin
- Soluble interleukin 2 (IL-2) receptor (soluble CD25)
- Triglycerides
- AST, ALT, and direct bilirubin
- LDH
- Complete neurologic and abdominal examination (assess for hepatosplenomegaly)

Laboratory tests with normal results should be repeated frequently in patients for whom a high clinical suspicion of systemic immune activation exists. If neurologic abnormalities are present, consider cerebrospinal fluid analysis and/or an MRI of the brain. If cytopenias are present (Grade ≥ 2 in two or more lineages) or ferritin is ≥ 3000 ng/mL, the following evaluations should also be performed:

- Bone marrow biopsy and aspirate (assess for evidence of hemophagocytosis)
- Adenovirus, cytomegalovirus, Epstein-Barr virus, herpes-simplex virus, and human herpesvirus 6, 7, and 8 evaluation (for reactivated or active disease)

Diagnostic criteria and recommended management for systemic immune activation are provided in Table 14. The diagnostic criteria apply only when alternative etiologies have been excluded. An adverse event of systemic immune activation should be reported on the Adverse Event eCRF if it meets the criteria for "consistent with systemic immune activation" or "probable systemic immune activation" as outlined in Table 14.

Table 104 Diagnostic Criteria and Recommended Management for Systemic Immune Activation

Systemic Immune Activation Diagnostic Criteria (applicable only when alternative etiologies have been excluded)	
Major Criteria	Minor Criteria
• Fever ≥ 38.5°C on more than one occasion<br>• Ferritin ≥ 3000 ng/mL<br>• Cytopenias (Grade ≥ 2 in two or more lineages)<br>• Age-adjusted soluble interleukin-2 receptor elevated by ≥ 2 standard deviations<br>• Severe (Grade ≥ 3) or progressive dysfunction in two or more organs<br>• Decreased fibrinogen	• Splenomegaly<br>• Hemophagocytosis in bone marrow, spleen, or lymph nodes<br>• Elevated γ-glutamyl transpeptidase (GGT) or liver function tests (AST, ALT, or direct bilirubin)<br>• Elevated triglycerides<br>• Elevated LDH<br>• Decreased natural killer cell activity

Diagnosis and Management of Systemic Immune Activation		
Number of Criteria	Diagnosis	Action to Be Taken
≥ 4 major criteria	Consistent with systemic immune activation	• Permanently discontinue atezolizumab.<br>• Consider treatment with an immunosuppressive agent (i.e., cytokine inhibitors) and IV corticosteroids (i.e., methylprednisolone 1 g once daily or equivalent, or dexamethasone ≥ 10 mg/m^2 once daily if neurologic abnormalities are present).<br>• Contact the principal investigator for additional recommendations.<br>• Consider HLH-2004 protocol [Henter] if there is no clinical improvement.
3 major criteria<br>OR<br>2 major plus ≥ 3 minor criteria	Probable systemic immune activation	• Depending on clinical severity, follow guidelines for “Consistent with systemic immune activation” or “Possible systemic immune activation” diagnosis.<br>• Clinical specialists may be contacted for recommendations.
2 major plus ≤ 2 minor criteria<br>OR<br>1 major plus ≥ 4 minor criteria	Possible systemic immune activation	• Withhold atezolizumab.<br>• Consider treatment with IV corticosteroids.<br>• Clinical specialists may be contacted for additional recommendations.<br>• Follow guidelines for “Consistent with systemic immune activation” diagnosis if there is no clinical improvement or if clinical worsening occurs.<br>• If clinical improvement occurs, atezolizumab may be resumed following a benefit-risk assessment by the principal investigator.

Notes: Criteria are adapted from a Delphi Survey of 26 experts who provided helpful criteria in the positive diagnosis of hemophagocytic syndrome in adult patients [Hejblum].

Grades are based on National Cancer Institute Common Terminology Criteria for Adverse Events.

These recommendations do not replace clinical judgment and are intended as suggested guidance.

5.4 Concomitant medications

Patients must be instructed not to take any medications, including all over-the-counter products such as vitamins, minerals, and other dietary supplements, without first consulting with the investigator. All concomitant medication must be recorded on the Case Report Form (CRF). Additionally, any diagnostic, therapeutic or surgical procedure performed during the study period, should be recorded including the date, indication, description of the procedures(s) and any clinical findings. If medically feasible, patients taking regular medication, with the exception of the prohibited medication listed below, should be maintained on it throughout the study period.

Concomitant therapy includes any prescription medications or over-the-counter preparations used by a patient between the 7 days preceding the screening evaluation and the treatment discontinuation visit.

5.4.1 Permitted Therapy

Patients who experience infusion-associated symptoms may be treated symptomatically with paracetamol, ibuprofen, diphenhydramine and/or famotidine or another H2 receptor antagonist, as per standard practice. Serious infusion-associated events manifested by dyspnea, hypotension, wheezing, bronchospasm, tachycardia, reduced oxygen saturation or respiratory distress should be managed with supportive therapies as clinically indicated (e.g., supplemental oxygen and β2-adrenergic agonists).

Systemic corticosteroids and tumour necrosis factor−α inhibitors may attenuate potential beneficial immunologic effects of treatment with atezolizumab but may be administered at the discretion of the treating physician. If feasible, alternatives to corticosteroids should be considered. Premedication may be administered for Cycles ≥ 2 at the discretion of the treating physician. The use of inhaled corticosteroids for COPD and mineralocorticoids (e.g., fludrocortisone) and low-dose corticosteroids for patients with orthostatic hypotension or adrenocortical insufficiency is allowed. Megestrol administered as an appetite stimulant is acceptable while the patient is enrolled in the study.

Colony-stimulating factors, such as granulocyte colony-stimulating factor and erythropoietin, should only be used according to the ASCO and ASCO/ASH guidelines, respectively (Smith TJet al. 2006; Rizzo et al. 2010). Influenza vaccination should be given during influenza season only (approximately October to March).

Patients who use hormonal therapy with gonadotropin-releasing hormone agonists or antagonists for prostate cancer, oral contraceptives, hormone-replacement therapy, prophylactic or therapeutic anticoagulation therapy (such as low molecular weight heparin or warfarin at a stable dose level), or other allowed maintenance therapy should continue their use.

Sexually actives females of reproductive potential should use a highly effective means of contraception during the study and for at least 5 months after last dose of study drug. Male should use condom while on SABR treatment and for at least 90 days after SABR treatment. But, taking into account the irradiated area, use of condom after SABR treatment can be shortened at investigator discretion

5.4.2 Prohibited Therapy

Any concomitant therapy intended for the treatment of cancer, whether health authority-approved or experimental, is prohibited. This includes but is not limited to the following:

Chemotherapy, hormonal therapy, immunotherapy, investigational agents or herbal therapy

• Traditional herbal medicines should not be administered because the ingredients of many herbal medicines are not fully studied, and their use may result in unanticipated drug-drug interactions that may cause or confound assessment of toxicity

• Patients who are receiving a receptor activator of nuclear factor kappa B ligand inhibitor (denosumab) prior to enrollment must be willing and eligible to receive a bisphosphonate instead while on study; denosumab could potentially alter the activity and the safety of Atezolizumab

Patients must not receive live, attenuated influenza vaccine (e.g., FluMist®) within 4 weeks prior to Cycle 1, Day 1 or at any time during the study but may receive inactivated vaccine.

Patients should not receive any live, attenuated vaccine (e.g., FluMist®) at any time during the study while the patient is receiving atezolizumab and for a period of 5 months after the discontinuation of atezolizumab.

Patients are not allowed to receive immunostimulatory agents, including but not limited to interferon (IFN)-α, IFN-γ, or IL-2, during the entire study. These agents, in combination with atezolizumab, could potentially increase the risk for autoimmune conditions.

Patients should also not receive immunosuppressive medications, including but not limited to cyclophosphamide, azathioprine, methotrexate, and thalidomide. These agents could potentially alter the activity and the safety of atezolizumab.

Systemic corticosteroids and anti−TNF-α agents may attenuate potential beneficial immunologic effects of treatment with atezolizumab, but may be administered at the discretion of the treating physician. If feasible, alternatives to these agents should be considered.

In addition, all patients should not receive other immunostimulatory agents for 10 weeks after the last dose of atezolizumab.

The above list of medications is not necessarily comprehensive. The investigator should consult the prescribing information for any concomitant medication.

6 PLAN OF THE STUDY

The patient visits from the date of enrolment and the evaluation schedule are given in the summary Table 1.

6.1 Baseline assessments

Baseline assessment can be performed within 28 days before the first treatment administration unless stated otherwise.

- A complete physical examination including an evaluation of the head, eyes, ears, nose, and throat and the cardiovascular, dermatological, musculoskeletal, respiratory, gastrointestinal, and neurological systems

- Documentation of concomitant treatment and collection of AE

- Electrocardiogram
- ECOG performance status, arterial blood pressure, O2 saturation, cardiac rhythm, temperature, weight, lung and heart auscultation, lymph node examination for glandular enlargement

▪ **Imaging**:

- FDG-PET/CT (optional)
- Chest and abdominal perfusion CT scan (with IV contrast agent injection) (performed within 2 weeks before registration at most).
- Brain CT scan and/or brain MRI scan (with IV contrast agent injection) if clinical signs suggesting brain metastases (performed within 2 weeks before registration at most), or if known brain metastases.
- According to the investigator judgment: Liver MRI, Spinal MRI, brain MRI.

▪ **Laboratory evaluations :**

- Hematology : CBC, including RBC count, hemoglobin, hematocrit, WBC count with differential [neutrophils, eosinophils, lymphocytes, monocytes, basophils, and other cells], and platelet count
- Serum chemistries : BUN, sodium, potassium, magnesium, chloride, bicarbonate, calcium, phosphorus, glucose, creatinine/creatinine clearance, total bilirubin with fractionation into direct and indirect (if total bilirubin elevated during the study), ALT, AST, alkaline phosphatase, lactate dehydrogenase, total protein and albumin
- Coagulation (aPTT and INR)
- Serum pregnancy test for women of childbearing potential, including women who have had a tubal ligation
- Urinalysis : specific gravity, pH, glucose, protein, ketones, and blood; (dipstick permitted)
- Thyroid function testing : thyroid-stimulating hormone [TSH], free T3, free T4
- HBV serology (HBsAg, antibodies against HBsAg, hepatitis B core antigen); HBV DNA should be obtained prior to Cycle 1, Day 1 if patient has positive serology for anti-HBc Ab
- HCV serology (anti-HCV)
- C-reactive protein (CRP)
- Urine ketones and urine glucose in case of hyperglycemia > 20 mmol / L,

▪ **Translational research (optional)**

- Tumour biopsy under ultrasonography guidance (for paraffin inclusion and snap frozen tissue)

- Immunomonitoring: Blood sampling (50 ml) (only for patients included at Gustave Roussy, France)
- Serum banquing (5 ml)

6.2 During the treatment period

- Physical examination (every three weeks from W1 to W4, every week during SABR and up to W10 and then prior each atezolizumab injection)
- Safety and toxicity assessment with collection of AE
- Documentation of concomitant treatment

▪ **Imaging** (at week 4, 7, 13, and then every 12 weeks – prior atezolizumab administration):

- FDG-PET/CT (optional)
- Chest and abdominopelvic CT (± 7 days allowed)
- According to the investigator judgment : Liver MRI, Spinal MRI, Brain CT scan (with IV contrast agent injection), Brain MRI

▪ **Laboratory evaluations** (prior to each atezolizumab administration):

- Hematology: CBC, including RBC count, hemoglobin, hematocrit, WBC count with differential [neutrophils, eosinophils, lymphocytes, monocytes, basophils, and other cells], and platelet count)
- Serum chemistries: BUN, creatinine/creatinine clearance, sodium, potassium, magnesium, chloride, bicarbonate, calcium, phosphorus, glucose, total bilirubin with fractionation into direct and indirect (if total bilirubin elevated during the study), ALT, AST, alkaline phosphatase, lactate dehydrogenase, total protein and albumin)
- Coagulation (aPTT and INR)
- Serum pregnancy test for women of childbearing potential, including women who have had a tubal ligation
- Urinalysis: specific gravity, pH, glucose, protein, ketones, and blood; dipstick permitted
- Thyroid function testing : thyroid-stimulating hormone [TSH], free T3, free T4
- C-reactive protein (CRP)
- Urine ketones and urine glucose in case of hyperglycemia > 20 mmol / L

▪ **Translational research (optional)**

- Immunomonitoring (at week 4, 7 and 13, and then every 12 weeks up to treatment discontinuation – to be sampled prior atezolizumab administration): Blood sampling (50 ml) (only for patients included at Gustave Roussy, France)

- Serum banquing (5 ml): sampling performed at the same timepoints than immunomonotoring

- Tumour biopsie (at week 4 or up to 8 days before C2D1 and week 7 or up to 8 days after C3D1)

6.3 During the follow-up period

❖ Patients who **discontinue atezolizumab for disease progression** will have an end of study visit 1 month after treatment discontinuation which will include following examinations:

  - Physical examination

  - Safety and toxicity assessment with collection of AE

In patients with lung cancer and sarcoma, in case of first radiological progression as per RECIST v1.1, a second SABR course can be delivered. Patients will discontinue all treatment after second progression.

❖ Patients who **discontinued atezolizumab for other reason than disease progression** will have follow-up visit every 3 months up to 12 months or disease progression according to RECIST 1.1, whichever occurs first, and will include following examinations:

  - Physical examination

  - Safety and toxicity assessment with collection of AE

- **Imaging**
  - Chest and abdominopelvic CT

  - According to the investigator judgment : Liver MRI, Spinal MRI, Brain CT scan (with IV contrast agent injection), Brain MRI

6.4 Exploratory objectives (optional part of the study):

6.4.1 Functional imaging changes using FDG PET/CT

The objective is to study the relationship between the administration of atezolizumab and SABR and early changes in the FDG uptake of the tumour on PET/CT imaging. FDG-PET/CT will be performed:

- At baseline (within 28 days before patient registration),

- At week 4

- At week 7

- At week 13

- And then, every 12 weeks up to treatment discontinuation

6.4.2 *Systemic immunologic anti-tumour response*

6.4.2.1 Biomarker research on tumour samples

When available, archival tumour material should be made available.

Additionally, in patients with irradiated and unirradiated tumours sites accessible to tumour biopsy and having signed the additional written consent, sequential tumour biopsies will be performed :

- At baseline, *i.e. before atezolizumab treatment* (on irradiated site or, if not possible and as second option from evaluable lesion (unirradiated site) or, if not possible and as third option from other unirradiated lesion (metastasis site))
- At week 4 (before start of SABR, both on sites to be irradiated and unirirradiated and including the same lesion than the one performed at baseline),
- At week 7 (after atezolizumab and SABR combination, both on irradiated and unirirradiated sites, and from the same lesions than the previous ones).

Only tissue from core needle, punch or excisional biopsy sample collection will be accepted.

A maximum of 3 cores will be collected at each biopsy. The choice of the site shall be left to the discretion of the investigator team, who shall determine what is feasible and safest on the basis of accessibility and the volume of the metastasis.

Tumour irradiation might serve as an adjuvant of immunotherapy as it can trigger e.g. additional tumour-antigen release, which supports stimulation of anti-tumour immune-responses (see introduction). A sequential tumour biopsy approach will help to further characterise changes in the tumour immune landscape and if/how such changes generate a favourable tumour microenvironment for combination therapy with atezolizumab. All available longitudinal tumour samples from each patient will be analysed and compared: archival tumour samples, biopsies at baseline, during week 4 and week 7. The exploratory biomarker research, which aims to characterise the above mentioned changes, might utilize the below (but not limited to) assessments:

- Investigation of expression changes of immune-checkpoint molecules (e.g., but not limited to PD-L1 staining via IHC)
- Comparison of dynamic changes of immune cell subpopulations like T cells, B cells, Dendritic cells and Macrophages (e.g. but not limited to CD8, CD3/Perforin+ T-cells, Tregs, CD68/CD163, macrophages, MHC class I & II staining via IHC)
- Comparison of RNA expression profiles by e.g. RNAseq
- Comparison of (targeted) DNA mutation profiles by e.g. Next Generation Sequencing methods

- Assessment of T-cell receptor diversity by e.g. sequencing

6.4.2.2 Biomarker research on blood samples (flow cytometry)

50 ml of peripheral blood for immunomonitoring (only for patients included at Gustave Roussy, France) and 5 ml for serum banquing (for all patients) will be collected distantly from atezolizumab administration (immediately before the next infusion *e.g* immediately before C2, C3, C5, etc).

- At baseline (within 28 days before patient registration),
- At week 4
- At week 7
- At week 13,
- And then every 12 weeks up to treatment discontinuation.

We have validated 7 color (10C) panels for **fresh** whole blood (100 µL heparinized WB per 10C panel) for the blood immunophenotyping and PBMC banking.

The analysis might include but is not limited to the following mentioned parameters:

• Numeration panel with beads allowing absolute counts: Lymphocytes T4, Lymphocytes T8, Lymphocytes NK, Lymphocytes B, Eosinophils, neutrophils: this is mandatory to have absolute cells.

• T differentiation panel: for T4 and T8 as well as Treg (CD127low CD25high) , ICOS and HLA-DR with TN, TEM, TCM, TEMRA and TSCM

• T cell activation panel: T4, T8, PD1, OX40, 4.1BB, CD57

• Treg "function": (CD4 staining as well as CD8 and Treg (CD127low CD25high)) LAP, GITR, LAG-3, OX40

• Treg proliferation intracellular: (CD4 staining as well as CD8 and Treg (Foxp3/Helios/CD127/CD25)) with Foxp3, Helios, Ki67, CTLA4

• T polarization: Lymphocytes Th1, Th2, Th9, Th17, Th1/Th17, Th22 and TFh

• MDSC panel (including monocyte MDSC, GranMDSC and inflammatory monocytes)

We will perform 7 panels 10 colors for the first 10 patients and then focus on the panels of interest for the rest of the cohort.

7 EVALUATION CRITERIA

7.1 Primary criterion

In each cohort, progression-free survival rate at one year is defined as the rate of patients alive and free of progression at one year from treatment initiation. Progression is defined using RECIST 1.1. criteria, or death, whatever the cause of death, whichever occurs first.

7.2 Secondary criteria

7.2.1 Efficacy

- Progression-free survival (PFS) measured from the date of treatment initiation to the date of progression or death, whichever comes first. Patients alive and free of progression at the cut-off date will be censored at the last assessment date.
- Modification of the size of the irradiated and non irradiated lesion(s) as measured by its longest diameter according to RECIST 1.1 will be compared.

Assessment of efficacy endpoint listed below will be performed according to RECIST v 1.1 criteria (refer to Appendix 4: RECIST 1.1 CRITERIA) and modified RECIST (mRECIST) (refer to Appendix 5: mRECIST CRITERIA MODIFIED RESPONSE EVALUATION CRITERIA IN SOLID TUMORS).

Efficacy endpoints are:

- Disease Control Rate (DCR): complete responses (CR) + partial responses (PR) + stable disease (SD).
- Objective Response Rate (ORR): CR + PR
- Duration of response (DOR)
- Time to progression (TTP) of <u>non irradiated</u> lesions is computed as the delay from enrollment to the first occurrence of progression outside of the irradiated field
- Time to progression (TTP) of <u>irradiated</u> lesions is computed as the delay from enrollment to the first occurrence of progression of irradiated lesions;
- Evaluation of the local control, distant control.

In lung cancer and sarcoma patients receiving a second SABR course:

For patients in lung and sarcoma cohorts who can receive a second SABR course if oligoprogression, the objective response rate at 6 weeks is defined as any partial or complete response according to RECIST v1.1 observed at the first radiological evaluation (6 weeks after the second SABR course) over the number of patients that received a second SABR course.

- Size of the irradiated and non irradiated lesion(s) is measured in its longest diameter according to RECIST 1.1. Modification observed between the time of progression 1 and the value at 6 weeks will be compared between irradiated and non irradiated lesions.

- Progression-free survival 2 (PFS2) measured from the date of the second SABR course to the date of second progression or death. Patients alive and free of progression at the cut-off date will be censored at the last assessment date.
- Time to progression ratio (TTPr) in patient that received a second SABR course. To define the TTP ratio, let's denote:
  - TTP1 the time from treatment initiation to the time of progression after first irradiation
  - TTP2 the time from first progression to the time of progression after the second SABR course. Patients alive and free of progression at the cut-off date will be censored at the last assessment date. Radiological progression is measured using recist v1.1 on the same tumor lesions over all the trial whenever possible. TTP2 is defined as PFS2 as it is unlikely that patients die of cause unrelated to the treatment failure and subsequent tumor progression.

7.2.2 Safety

Toxicity is assessed according to the NCI- CTCAE V4.03 scale during physical examination Identified risks as well as potential risks associated with atezolizumab are extensively described in the Investigator Brochure.

Toxicity is the adverse events graded with the NCI-CTC-AE v4.03 scale, at least possibly related to the treatment.

8 DETERMINATION OF SAMPLE SIZE AND STATISTICAL ANALYSIS

8.1 Sample size

One-year progression-free survival will be estimated in CRC (cohort 1, *closed to inclusions*), in NSCLC (cohort 2, *closed to inclusions*), in RCC (cohort 3, *closed to inclusions*) and in sarcoma (cohort 4, *closed to inclusions*). Within each cohort, a Fleming 1-stage design will be applied to demonstrate that the progression free survival rate at one year is not inferior to 15% but could reach 32%.

To ensure a global alpha risk (Type I error) will be fixed at 0.1 (unilateral), the Bonferroni correction will applied: Out of the 4 cohort, one (RCC cohort) has been closed for lack of accrual and no test will be carried out in this cohort. Therefore, the alpha risk for each test will be 0.033.

To test in each cohort, the hypothesis that the PFS rate is greater than p0 with alpha=0.033 and with a 90% power to detect activity greater than p1, we need to enroll 54 evaluable patients.

If 13 patients or more are alive and free of progression at one year in the cohort, the combined SABR+ atezolizumab will be considered as a success for the corresponding histology.

To take into account a possible rate of 10% of non evaluable patients, and considering that the renal cohort will close after the enrolment of 7 patients, the total number of patients will be 187 = 3 * 60 + 7 to achieve 162 evaluable patients total in the 3 opened cohorts.

In lung cancer and sarcoma patients receiving a second SABR course:

A group of NSCLC and sarcoma patients progressing after their initial treatment with SABR+atezolizumab will be enrolled for a second SABR course. Assuming that up to 50% of patients may display a oligoprogression (i.e. being eligible for a second SABR course), after immune checkpoint blockers, between 25 and 30 patients per cohort are expected to receive a second SABR course. These cohorts size will enable to detect a response rate at 6 weeks of p1=30% versus a response rate below p0=10% at the 10% type I error level (one-sided) and with a 90% power. In each cohort, if 5 or more responses are observed, the second SABR course will be considered as promising. This threshold will be recomputed based on the actual number of patients re-irradiated in each cohort.

8.2 Stopping rule

Safety and feasibility will be carefully monitored and strict statistical stopping rules will be implemented. The following limiting events will be considered for the first 20 patients (all cohorts combined together:

- Any toxicity of grade 4 and above
- Any toxicity leading to treatment interruption more than 2 cycles.

The approach developed by Kramar and Molevi (2009) will be used to define stopping boundaries. The maximum acceptable rate of limiting event is defined as 20%. This gives the following stopping rules for the first patients (across all cohorts):

Stop if

Number of events	2	3	4	5	6	7
in less than (n_k assessable pts)	2	4	8	11	15	18

8.3 Data Analysis

8.3.1 General Considerations

- Statistical analysis for this study will be the responsibility of the Sponsor. For continuous variables, summary statistics will include number of patients, mean, standard deviation, median, interquartile range, minimum, and maximum. Categorical endpoints will be summarized using number of patients and frequency in %. Missing data will not be imputed.
- The interpretation of the study results will be the responsibility of the investigators, pharmacokineticist and statistician.
- Exploratory analyses of the data not described below will be conducted as deemed appropriate.

8.3.2 Efficacy Analysis

Progression free survival (PFS) is defined as the time from treatment initiation to first documentation of tumour progression or to death, whatever the cause of death, whichever occurs first. Any patient that discontinues due to a reason other than progressive disease (for example, AE, patient/investigator decision) will have their time-to-event data either censored at

the follow-up visit if no progressive disease is found during this visit, or censored at the date of the last objective progression-free disease assessment if progressive disease is found at the follow-up visit. The progression will be assessed using RECIST v 1.1 and mRECIST.

Time to progression of non irradiated lesions is computed as the delay from enrollment to the first occurrence of progression outside of the irradiated field. Time to progression of irradiated lesions is computed as the delay from enrollment to the first occurrence of progression of irradiated lesions.

The disease Control Rate (DCR) will be defined as the sum of complete responses (CR), partial responses (PR) and stable disease (SD). The objective Response Rate (ORR) will be defined as the sum of complete responses (CR) and partial responses (PR).

The PFS, DCR, ORR and DOR will be computed by patient. In each cohort, response rates with exact 95% confidence intervals will be provided, and PFS and duration of response will be estimated using Kaplan-Meier survival method and presented with Rothman's 95% Confidence Intervals. Median follow-up will be estimated using inverse Kaplan-Meier method (Collet D., 1994). Secondary analysis will investigate the impact of the number of metastatic sites in 2 modalities (<5 and ≥5). A semi-parametric Cox model stratified on the cohort and adjusted for the number of metastasis will be developed.

The TTP ratio (TTPr) will be computed in all patients receiving a second SABR course, as the ratio of TTP2 over TTP1. Patients receiving a second SABR course without a documented progression 1 will not be included. Patients dying without documented second progressions will be counted as progressions at the date of death considering the very poor prognosis of those patients. Censored TTP2 will make the TTPr censored too. In this case, the Kaplan-Meier approach will be used to derive values of TTPr. For instance, rate of patients with TTPr greater than 1.5 will be computed, among other thresholds (1.33, 1.5, 2).

8.3.3 Safety Analysis

All included subjects who take at least 1 dose of the atezolizumab will be included in the safety analyses. Adverse events will be summarized by worst severity grade. AEs, as well as treatment-emergent AEs, will be summarized by system organ class, and preferred term. Atezolizumab-related adverse events, adverse events leading to death or to discontinuation from treatment, events classified as NCI-CTCAE v4.0 Grade 3 or Grade 4 (or moderate/severe if other rating scale is used), atezolizumab-related events, and serious adverse events will be summarized separately.

Cross tabulations will be provided to summarize frequencies of abnormalities.

By-subject listings will be provided for all relevant safety data.

Graphical displays and figures will be provided where useful to assist in the interpretation of results especially blood pressure handling.

Descriptive analysis will be described for:

- Deaths,
- SAEs,
- AE drop-outs,
- AEs.

9 SERIOUS ADVERSE EVENTS

9.1 Definition

9.1.1 Adverse Event (AE)

An Adverse Event (AE) is any new untoward medical occurrence or worsening of a preexisting medical condition in a patient or clinical investigation subject administered an investigational (medicinal) product. An AE can therefore be any unfavourable and unintended sign (including abnormal laboratory findings for example), symptom, or disease temporally associated with the use of a medical product, whether or not a causal relationship (i.e. related/not related) with the treatment is suspected.

Any unfavorable and unintended sign (including an abnormal laboratory finding), symptom, or disease temporally associated with the treatment, whether or not considered related to study treatment.

Any deterioration in a laboratory value or other clinical test (e.g., ECG, X-ray) that is associated with symptoms or leads to a change in study treatment or concomitant treatment or discontinuation from study treatment.

9.1.2 Serious Adverse Event (SAE)

A Serious Adverse Event (SAE) is any untoward medical occurrence that at any dose:

- Is fatal (results in death)
- Is life-threatening
- Requires or prolongs in-patient hospitalization

- Results in persistent or significant disability / incapacity
- Is a congenital anomaly / birth defect
- Is medically significant (defined as any clinical event or laboratory result that may not be immediately life-threatening or result in death or hospitalization but, based upon appropriate medical and scientific judgment, may jeopardize the subject or may require intervention (eg,medical, surgical) to prevent one of the other serious outcomes listed in the definition above. Examples of such events include but are not limited to, allergic bronchospasm requiring intensive treatment in an emergency room or at home, blood dyscrasia or convulsions that do not result in inpatient hospitalization, development of drug dependency or drug abuse, transmission of an infectious agent.

Although overdose and new cancer are not always serious by regulatory definitions, these events should be reported on a SAE report form and sent to the sponsor in an expedited

A SAE judged as potentially related to a study drug qualifys as Serious Adverse Drug Reaction (SADR).

The following are not considered to be serious adverse events (SAE):

- Events exclusively related to tumour relapse / progression or treatment of tumour relapse / progressions are not considered as SAE,
- A visit to the emergency room or other hospital department for less than 24 hours that does not result in admission (unless considered an “important medical event” or a life-threatening event),
- Outpatient or same-day or ambulatory procedures,
- Observation or short-stay units,
- Hospitalization due to diagnostic procedures or standard supportive care (e.g. implant of central venous catheter),
- A pre-planned hospitalization for a condition which existed at the start of study drug and which did not worsen during the course of study drug treatment,
- Social admission (e.g., subject has no place to sleep; hospice facilities),
- Administrative admission (e.g., for yearly physical examinations),
- Protocol-specified admission during a clinical trial (e.g., for a procedure required by the study protocol or for clinical research),
- Optional admission not associated with a precipitating clinical AE (e.g., for elective cosmetic surgery).

9.1.3 Expected Serious Adverse Event

An expected SAE is an event already mentioned in the most recent version of the investigator brochure.

9.1.4 Unexpected Serious Adverse Event

An unexpected SAE is an event not mentioned or different by its nature, intensity and/or, evolution with respect to the investigator brochure.

9.1.5 Intensity criteria

Intensity criteria must not be confused with criteria for seriousness, which serve as guidelines for definition of reporting obligations.

Intensity of events will be estimated according to the NCI-CTCAE classification, version 4.0 (toxicity score grade 1 to 5). Intensity of adverse events not listed in this classification will be evaluated according to the following terms:

- Mild (grade 1): does not affect the patient's usual daily activity
- Moderate (grade 2): perturbs the patient's usual daily activity
- Severe (grade 3): prevents the patient carrying out his usual daily activities
- Very Severe (grade 4): necessitates intensive care or is life-threatening
- Death (grade 5)

9.2 Reporting of Serious Adverse Events (SAE), Adverse Events of Special interest (AESI) and pregnancy

9.2.1 Reporting of Serious Adverse Events (SAE)

Any SAE/AESI which occurs or comes to the attention of the investigator at any time during the study since consent is given and within 90 days after the last administration of study drug/treatment, independent of the circumstances or suspected cause, must be reported immediately, via my eclinical, a web portal that allow electronic transmission of SAEs /AESIs, https://myeclinical.evedrug.eu/form/IGR/login.php or if it's not possible by fax using a SAE report form at +33 (0) 1 42 11 61 50 :

**Pharmacovigilance unit**
Phone : +33 (0)1 42 11 61 00
(9 a.m. - 6 p.m. from Monday to Friday, except on bank holidays)
E-mail: phv@gustaveroussy.fr/CTpharmacovigilance@gustaveroussy.fr

All late Serious Adverse Events/AESIs (occurring after this period of 90 days) considered to be reasonably related to the study treatment(s) or the research must be reported (no time limit).

Information collected in the SAE form is crucial to assess the case. For this reason, diligence in collecting as much verifiable and reliable information is needed: both, quality and timeliness are key factors. If known, the diagnosis of the underlying illness or disorder should be recorded, rather than its individual symptoms. The following information should be captured for all SAEs/AESIs: onset, duration, intensity, and seriousness, relationship to study drug/treatment, action taken and treatment required.

The investigator must also attach the following to the serious adverse event report form, wherever possible:

- A copy of the summary of hospitalization or prolongation of hospitalization
- A copy of the post-mortem report (if applicable)
- A copy of all relevant laboratory examinations and the dates on which these examinations were carried out, including relevant negative results, as well as normal laboratory ranges.
- All other document that he judges useful and relevant.

All these documents will remain anonymous.

Further information can be requested (by e-mail, fax, telephone or when visiting) by the monitor and/or the safety manager.

Follow-up information

The investigator is responsible for the appropriate medical follow-up of patients until resolution or stabilization of the adverse event or until the patient's death. **This may mean that follow-up should continue once the patient has left the trial.**

Follow up information about a previously reported serious adverse event must be reported by the investigator to the Pharmacovigilance Unit immediately after becoming aware of it. The investigator also transmits the final report at the time of resolution or stabilization of the SAE. /AESI.

9.2.2 Reporting of (Non-Serious) Adverse Events of Special Interest (Study Specific AESI)

There are certain types of AE that are considered of study specific importance (AESI). These AEs are summarized below and must be reported in an expedited manner (immediately after learning of the event) by using my eclinical report or if not possible the SAE form, even if they do not meet the criteria for seriousness (check the box 'medically significant' and specify 'Non-Serious Adverse Event of Special Interest').

Adverse events of special interest for this study include the following:

- Pneumonitis
- Hypoxia or dyspnea Grade ≥ 3
- Myocarditis (any grade) and other cardiac disorders grade ≥ 2
- Autoimmune mediated pericardial disorders including pericarditis, pericardial effusion and cardiac tamponade
- Colitis
- Endocrinopathies: diabetes mellitus, pancreatitis, adrenal insufficiency, thyroiditis (hypo or hyperthyroidism), hypophysitis
- Hepatitis
- Pancreatitis

- Transaminitis: Grade ≥ 2 (AST or ALT > 3 × ULN AND total bilirubin > 2 × ULN) or AST/ALT > 10 × ULN
- Systemic lupus erythematosus
- Neuropathies: Guillain-Barré syndrome, Myasthenic syndrome, Myasthenia Gravis, Meningoencephalitis
- Immune-related Nephritis

- Events suggestive of hypersensitivity, cytokine release syndrome, influenza-like illness, systemic inflammatory response syndrome, systemic inflammatory activation or infusion-reaction syndromes, Hemophagocytic lymphohistiocytosis or macrophage activation syndrome

- Cases of potential drug-induced liver injury that include an elevated ALT or AST in combination with either an elevated bilirubin or clinical jaundice, as defined by Hy's law
- Suspected transmission of an infectious agent by the study drug, as defined below:
  - Any organism, virus, or infectious particle (e.g., prion protein transmitting transmissible spongiform encephalopathy), pathogenic or non-pathogenic, is considered an infectious agent. A transmission of an infectious agent may be suspected from clinical symptoms or laboratory findings that indicate an infection in a patient exposed to a medicinal product. This term applies only when a contamination of the study drug is suspected.
- Immune-related myopathies includingrhabdomyolysis
- Vasculitis
- Myositis
- Autoimmune haemolytic anaemia
- Severe cutaneous reactions (e.g., Stevens-Johnson syndrome, dermatitis bullous, toxic epidermal necrolysis)
- Ocular toxicities (e.g. uveitis, retinitis, optic neuritis)
- Myelitis
- Facial paresis

9.2.3 Reporting of exposure to study drug during pregnancy/lactation.

In the event of a pregnancy occurring during the course of the study, the subject must be withdrawn from the clinical trial immediately. The Pharmacovigilance Unit of GUSTAVE ROUSSY must be notified within 24h (via the pregnancy report form) and the subject followed by a multidisciplinary team during the entire course of the pregnancy and postpartum period. Parental and neonatal outcomes must be recorded even if they are completely normal and without AEs.

Women who become pregnant should also be advised of the possibility of harm to the foetus.

9.3 Responsibilities of the coordinating Sponsor

The Pharmacovigilance Unit at GR will assess the SAE in terms of seriousness, severity (NCI-CTCAE v4.0), relationship to the study drug/treatment and expectedness). All SAEs will be coded using MedDRA.

Suspected Unexpected Serious Adverse Reactions (SUSARs)

To comply with regulatory requirements, the coordinating sponsor will notify all national sponsor of all SAEs that are related to the investigational medicinal product and unexpected (ie, not previously described in the investigator brochure). In the European Union, an event meeting these criteria is termed as suspected Unexpected Serious Adverse Reaction (SUSAR).

In case of a SUSAR, a CIOMS-1 form will be sent by the Pharmacovigilance Unit of Gustave Roussy to each national sponsor within 72 hours.

Each national sponsor is responsible for the reporting to the National Ethic committee (if required according to local regulation) and to the national competent authority.

All SUSAR reports and all reports involving expected SADR that are fatal will additionally be forwarded to all study investigators and to the Independent Data Monitoring Committee. The involved study offices are responsible for information of the Ethical Review Board(s) concerned as well as of the respective investigators. The reporting procedure has to comply with the national legislation.

Development Safety Update Report

The pharmacovigilance unit at Gustave Roussy will issue once a year throughout the clinical trial, or on request, the Development Safety Update Report (DSUR) of the study, in accordance with the ICH E2F detailed guidance

The pharmacovigilance unit will send a copy of the DSUR to national sponsors.

Each national sponsor should submit the DSUR within 60 days of the data lock point (date of the first authorisation of the concerned clinical trial by a competent authority in a member state) to the national competent authority and the national Ethic Committee of the concerned Member State, according to national legislation.

9.4 Data Safety Monitoring Board (DSMB)

A DSMB will be organised under the supervision of the coordinating investigator and composed of people well trained in the field of clinical research in oncology not directly involved into the current trial.

The DSMB will meet after the first 15 patients are enrolled and then when one third, half and then two third of the inclusions are met.

The role of the DSMB is to advise on the safety of the trial and to give advice regarding the continuation of the study.

In the meantime, up to the first 15 patients are enrolled, meeting or teleconferences will be organised after the first 5 patients and then after the first 10 patients are enrolled between the coordinating investigator and investigators from sites where patients were enrolled to review safety data. End of meeting reports will be sent to DSMB members within 2 working days after meeting or teleconferences. If deemed necessary by a DSMB member or the coordinating investigator or the sponsor, a DSMB will be organized before the first 15 patients are enrolled.

In case of disagreement between DSMB and investigators, Competent Autorities will be informed and will receive meeting reports.

10 STUDY DISCONTINUATION

The study could be interrupted or terminated by the sponsor in agreement with the coordinator and with the competent authority for the following reason:

- frequency and/or unexpected severity of the toxicity,
- recruitment of patients too low,
- poor quality of the data collected,
- request of the DSMB.

11 ETHICAL AND REGULATORY ASPECTS

11.1 Rules and regulations

The clinical trial is conducted in conformity with:

- Ethical principles stated in the Declaration of Helsinki 1964, as revised in Fortaleza, 2013
- Regulation (EU) 2016/679 of the europan parliament and of the council of 27 April 2016 on the protection of natural persons with regard to the processing of personal data and on the free movement of such data, and repealing Directive 95/46/EC (General Data Protection Regulation)
- Regulation (EU) 536/2014 of the european parliament and of the council of 16 april 2014 on clinical trials on medicinal products for human use, and repealing directive 2001/20/EC
- The European Directive (2001/20/EC and 2005/28/EC)
- Appendix 13 of the E. U. Guide to Good Manufacturing Practices (revised and adopted in February 2010 by the European Commission),
- The Good Clinical Practices guidelines (International Conference on Harmonization ICH E6 and Statistical Principles for Clinical Trials (ICH E9),
- The Clinical Safety Data Management guidance (ICH E2A),
- And any local Regulations including:
    - French Public Healthcare Law (n° 2004-806) of August 9, 2004, a partial adaptation of the European Directive (2001/20/EC) on the conduct of clinical trials,
    - French Public Healthcare Law (n° 2016-41) of January 26, 2016, about modernisation of the health system,
    - Ordinance n°2016-800 of June 16, 2016 on medical research involving human subjects,
    - French Law n° 2002-303 of March 4, 2002 relative to patients’ rights and to the quality of the healthcare system,
    - French Informatics and Liberties Law (n° 78-17) of January 6, 1978 modified by Law n° 2018-493 of June 20, 2018,
    - French decree N° 2018-687 of 1 August 2018 adopted for the application of Law No. 78-17 of 6 January 1978

11.2 Committee for the Protection of Persons (CPP) – Competent Authority

This protocol was submitted to the Ethic Committee/IRB/CPP which gave its approval on the 25/04/2016. This protocol has also been approved by the Competent Authority on the 16/06/2016.

Gustave Roussy has taken out a legal liability insurance policy (N°124.895).

A final report on the trial will be written at the latest, 1 year after the end of the trial and sent to the competent authority and to the Ethic Committee/IRB/CPP.

Gustave Roussy will maintain records of essential trial documentation in the Sponsor file for a minimum duration of 25 years after the end of the trial.

11.3 Information and Consent of Participants

Prior to the conduct of any procedure linked to biomedical research, any person wishing to participate in a research study gives his/her free, informed and written consent. This consent is obtained once the participant has been informed by the investigator during a consultation and after the person had been given sufficient time to think it over.

Having read the information notice, the patient must date and sign the **consent form** if he/she accepts to participate**.** This consent form must also be signed by the investigator. The original consent form must be kept in the study file by the investigator and the study participant should receive a copy.

11.4 Principal Investigator Responsibilities

The principal investigator of each establishment concerned promises to conduct the clinical trial in conformity with the protocol which has been approved by the CPP and the competent authority.

The principal investigator should not modify any aspect of the protocol without prior written permission from the Sponsor nor without the approval of the proposed modifications by the Ethic Committee/IRB/CPP and the competent authority.

The Principal Investigator is responsible for:

- providing the Sponsor with his/her CV as well as that of co-investigators,

-identifying members of his/her team participating in the trial and defining their responsibilities,

- recruiting patients after receiving the Sponsor’s approval,

Each investigator is responsible for:

- personally obtaining the informed consent form which has been dated and signed by the participant in the research prior to any specific trial selection procedure,

- regularly completing the case report form (CRF) for each patient included in the trial and ensuring that the Clinical Research Assistant (CRA) mandated by the Sponsor has direct access to source documents in order to validate information on the CRF,

- dating, correcting and signing the corrections on the CRF for each patient included in the trial,

- accepting regular visits from a CRA and possibly visits from auditors mandated by the Sponsor or inspectors from the regulatory authorities.

All documentation concerning the trial (protocol, consent form, case report form, investigator file, etc…), as well as the original documents (laboratory results, imaging studies, medical consultation reports, clinical examination reports, etc.) is considered confidential and should be kept in a safe place. The Principal Investigator should keep data as well as a list of patient-identifying data for at least 25 years after the end of the study.

12 DATA MANAGEMENT METHODS

12.1 Registration of patients

Patients will be prospectively registered in the study after checking of eligibility criteria and signature of the informed consent form. Registration will be performed on-line, using a connection process provided by the data manager. The confirmation of registration will be sent back to the investigator.

12.2 Data collection

The Biostatistics and Epidemiology Unit in Gustave Roussy will implement an electronic CRF (eCRF) using MACRO, software developed by InferMed, thus allowing secure online direct data collection. Each user will have personal identifiers (user ID / password) and data access will be strictly limited according to profiles:

- The hospital CRA profile allows data entry on the eCRF online,

- The Data Manager profile allows performing a first data monitoring, thanks to consistency checks, and edit requests for clarification addressed to the investigator or hospital CRA,

- The “Promoting” CRA profile allows checking of data sources,

- The investigator profile enables to sign electronically the data.

13 QUALITY ASSURANCE - MONITORING

In order to guarantee the authenticity and the credibility of the data in conformity with good clinical practices, the Sponsor has installed a quality assurance system which includes:

- trial management in accordance with the procedures at Gustave Roussy,

- quality control of data at the investigating site by the Clinical Research Assistant (CRA),

- possible auditing of investigating centres,

- the first 3 patients clinical and technical radiotherapy files of each center will be centrally reviewed by the sponsor.

13.1 Monitoring

Quality control on the site will be ensured by the CRA.

The CRA must check that the investigator's file exists and that it is updated.

The CRA must verify the consent forms, that subjects fulfil eligibility criteria, the validity of evaluation criteria and treatment toxicity with the help of source documents (and others to be specified and adapted according to the study).

The CRA will check drug accountability and ensure that the drug accountability forms are validated and signed by the in-house pharmacist before any request for destruction.

The data will be recorded through an electronic CRF (eCRF), the monitoring plan will be adapted in order to provide timely available data so to inform in due time the DSMB before every meeting and the investigators.

14 DATA OWNERSHIP / PUBLICATION POLICY

The investigator promises, on his/her behalf as well as that of all the persons involved in the conduct of the trial, to guarantee the confidentiality of all the information provided by Gustave Roussy until the publication of the results of the trial.

All publications, abstracts or presentations including the results of the trial require prior approval of the Sponsor (Gustave Roussy) and in parallel, the draft should be submitted for review by Roche.

All oral presentations, manuscripts must include a rubric mentioning the Sponsor, the investigators / institutions that participated in the trial, the cooperative groups, learned societies which contributed to the conduct of the trial and the bodies which funded the research.

The Study Investigator-Coordinator will write an article reporting on the results as soon as possible after the final analysis and will be the first author of the publication.

Specify the other authors (other investigators, statistician…) in conformity with « Uniform requirements for manuscripts submitted to biomedical journal » (http://www.icmje.org/). Indicate how you determine the order in which authors appear.

Indicate publications of additional results (biological study…).

15 DATA PROTECTION

15.1 Confidentiality

Investigator agrees that the collection, processing and disclosure of personal data and medical information related to the Subject, and personal data related to Investigator and any investigational staff is subject to compliance with applicable personal data protection and security laws and regulations.

Investigator agrees to adhere to the principles of medical confidentiality in relation to Clinical Trial Subjects.

Investigator shall not disclose the identity of Clinical Trial Subjects to third parties without prior written consent of the Sponsor.

15.2 Investigator's personal data

Investigator hereby expressly consents to the processing of Investigator's personal data collected by Sponsor. Such consent shall authorize the transfer of personal data to countries other than the Institution's own country, for the following purposes:

a) the conduct and interpretation of the Clinical Trial;
b) review by governmental or regulatory agencies, Sponsor, and its agents, affiliates and collaborators;
c) satisfying legal or regulatory requirements;
d) publication on national and international public websites and other websites and databases that serve a comparable purpose;
e) upon request of individual patients and doctors provision to individual patients and doctors who may be interested in participating in a clinical trial at Institution;
f) storage in Sponsor's databases for use in selecting sites in future clinical trials.

16 REFERENCES

1. Chen DS, Mellman I. Oncology meets immunology: the cancer-immunity cycle. Immunity 2013; 39, 1–10
2. Herbst RS, Soria JC, Kowanetz M, et al. Predictive correlates of response to the anti-PD-L1 antibody Atezolizumab in cancer patients. Nature. 2014 27;515:563-7.
3. Orui H., Ishikawa A., Okada K., Nishida J., Mitsui H., Kashiwa H., Tsuchiya T., Ogino T., Yamakawa M.. Dendritic cell and effector cell infiltration in soft tissue sarcomas with reactive lymphoid hyperplasia. J Orthop Sci.; 2003; 8:669-677.
4. Tseng WW, Demicco EG., Lazar AJ., Lev DC, Pollock RE. Lymphocyte composition and distribution in inflammatory, well-differentiated retroperitoneal liposarcoma: clues to a potential adaptive immune response and therapeutic implications. Am J Surg Pathol.; 2012; 36:941-944.
5. Kim JR., Moon YJ., Kwon KS., Bae JS., Wagle S., Kim KM., Park HS., Lee H., Moon WS., Chung MJ, et al. Tumor infiltrating PD1-positive lymphocytes and the expression of PD-L1 predict poor prognosis of soft tissue sarcomas. PLoS One; 2013; 8:e82870.
6. Tawbi HA, Burgess M et al. Pembrolizumab in advanced soft-tissue sarcoma and bone sarcoma (SARC028): a multicentre, two-cohort, single-arm, open-label, phase 2 trial. Lancet Oncol 2017; 18:1493-1501.
7. Burgess MA, Bolejack V, Van Tine BA, et al. Multicenter phase II study of pembrolizumab (P) in advanced soft tissue (STS) and bone sarcomas (BS): Final results of SARC028 and biomarker analyses. 2017 ASCO Annual Meeting. J Clin Oncol. 2017 35 :(suppl; abstr 11008).
8. O'Sullivan Coyne G, Sharon E et al. Phase 2 study of atezolizumab in patients with alveolar soft part sarcoma. CTOS 2018 Annual Meeting; Rome, Italy.
9. Sharma A, Bode B, Studer G, et al. Radiotherapy of human sarcoma promotes an intratumoral immune effector signature. Clin Cancer Res.; 2013;19(17):4843–4853.
10. Marcrom S, De Los Santos JF1, Conry RM. Complete response of mediastinal clear cell sarcoma to pembrolizumab with radiotherapy. Clin Sarcoma Res. 2017 Jul 14; 7:14.
11. Shaverdian N, et al. Previous radiotherapy and the clinical activity and toxicity of pembrolizumab in the treatment of non-small-cell lung cancer: a secondary analysis of the KEYNOTE-001 phase 1 trial. Lancet Oncology Jul;18(7):895-903.
12. Formenti SC, Demaria S. Combining radiotherapy and cancer immunotherapy: a paradigm shift. J Natl Cancer Inst. 2013 Feb 20;105(4):256-65
13. Tree AC, Khoo VS, Eeles RA, et al. Stereotactic body radiotherapy for oligometastases. Lancet Oncol. 2013;14:e28-37.
14. Salama JK, Milano MT. Radical irradiation of extracranial oligometastases. J Clin Oncol. 2014;32:2902-12. Chiang CS, Fu SY, Wang SC, et al. (2012). Irradiation promotes an M2 macrophage phenotype in tumour hypoxia. Front. Oncol. 2:89.
15. Chiang CS, Fu SY, Wang SC, Yu CF, Chen FH, Lin CM, Hong JH. Irradiation promotes an m2 macrophage phenotype in tumor hypoxia. Front Oncol. 2012 Aug
16. Deng L, Liang H, Burnette B, et al. Irradiation and anti-PD-L1 treatment synergistically promote antitumour immunity in mice. J Clin Invest. 2014 Feb;124(2):687-95.

17. Sagiv-Barfi I, Rajapaksa A, Czerwinski D, Chang S, et al. Local tumour irradiation combined with α-PDL-1 immune checkpoint inhibition results in local and systemic anti-tumour responses: successful translation of a mouse model to a human case series. Proceedings of the 105th Annual Meeting of the American Association for Cancer Research; 2014 Apr 5–9; San Diego, CA. Philadelphia (PA): AACR; 2014. Abstract nr 2941.

18. Postow MA, Callahan MK, Barker CA, et al. Immunologic correlates of the abscopal effect in a patient with melanoma. N Engl J Med. 2012;366:925-31.

19. C. Vanpouille-Box, M. Aryankalayil, K. Pilones, et al. Journal for ImmunoTherapy of Cancer 2014, S2:164. Marks LB, Yorke ED, Jackson A, Ten Haken RK, Constine LS, Eisbruch A, Bentzen SM, Nam J, Deasy JO. Use of normal tissue complication probability models in the clinic. Int J Radiat Oncol Biol Phys. 2010;76:S10-9.

20. Smith TJ, et al. 2006 update of recommendations for the use of white blood cell growth factors: an evidence-based clinical practice guideline J Clin Oncol. 2006 Jul 1;24(19):3187-205

21. Rizzo JD, et al. American Society of Clinical Oncology/American Society of Hematology clinical practice guideline update on the use of epoetin and darbepoetin in adult patients with cancer, J Clin Oncol. 2010 Nov 20;28(33):4996-5010. doi: 10.1200/JCO.2010.29.220

22. Collet D. (1994): Modelling survival data in medical research. Chapman and Hall, London

23. Eisenhauer E, et al. New response evaluation criteria in solid tumours: Revised RECIST guideline (version 1.1) Eur J Cancer 45 (2009) 228-247

24. Lencioni R, et al. New Data Supporting Modified RECIST (mRECIST) for Hepatocellular Carcinoma Clin Cancer Res; 2013, 19(6); 1312–4.

25. Jedd D. Wolchok, et al. Guidelines for the Evaluation of Immune Therapy Activity in Solid Tumors: Immune-Related Response Criteria Clin Cancer Res 2009;15(23) December 1, 2009

26. Postow MA, Callahan MK, Barker CA, et al. Immunologic correlates of the abscopal effect in a patient with melanoma. N Engl J Med 2012; 366:925-931

27. Golden EB, Demaria S, Schiff PB, et al. An abscopal response to radiation and ipilimumab in a patient with metastatic non-small cell lung cancer. Cancer Immunol Res 2013;1:365-372.

28. Reynders K, Illidge T, Siva S, et al. The abscopal effect of local radiotherapy: Using immunotherapy to make a rare event clinically relevant. Cancer Treat Rev 2015;41:503-510.

29. Golden EB et al. Local radiotherapy and granulocyte-macrophage colonystimulating factor to generate abscopal responses in patients with metastatic solid tumours: a proof-of-principle trial. Lancet Oncol 2015 Jul; 16(7):795-8032015.

30. Robert C, Schachter J, Long GV, Arance A, Grob JJ, Mortier L *et al*. Pembrolizumab versus ipilimumab in advanced melanoma. *N Engl J Med* 2015; 372: 2521– 32.

31. Herbst RS, Garon EB, Kim DW, Chul Cho B, Pérez Gracia JL, Han JY *et al*. LBA4 Long-term follow-up in the KEYNOTE-010 study of pembrolizumab (pembro) for advanced NSCLC, including in patients (pts) who completed 2 years of pembro and pts who received a second course of pembro. *Ann Oncol* 2018; 29: mdy511-003.

32. Hejblum G, Lambotte O, Galicier L, et al. A web-based Delphi study for eliciting helpful criteria in the positive diagnosis of hemophagocytic syndrome in adult patients. PLoS ONE 2014;9(4):e94024. doi: 10.1371/journal.pone.0094024.

33. Henter JI, Horne A, Aricó M, et al. HLH-2004: diagnostic and therapeutic guidelines for hemophagocytic lymphohistiocytosis. Pediatr Blood Cancer 2007; 48:124□31.

17 APPENDIX 1: RULES FOR SABR ADMINISTRATION ACCORDING TO TUMOUR LOCATION

All metastases are allowed for irradiation and a second SABR course, except brain lesions.

General requirement for SABR will be used, including:

- *Patients positioning*: patients should be immobilized with a vacuum cushion. The treatment

setup was reproduced should use modern image guidance, most commonly with kilovoltage cone beam modern imaging guidance such as on board computed tomography or ExacTrac patient positioning platform. Patients will have CT-based treatment planning in custom-made immobilization. CT images for radiation planning typically included a free breathing CT scan, an end expiratory respiratory gated scan, as well as 4-dimensional CT aided by contrast as needed.

- *Target volumes delineation*: in general, according to previous reports, GTV (Gross Tumour Volume) was equal to the CTV (Clinical Target Volume). They were defined as visible tumour using all available clinical and metabolic information with no additional margin to account for potential microscopic extension. The PTV (Planning Target Volume) was an expansion from CTV by 5-10 mm to account for set-up error and organ motion. In the Rochester experience, the PTV was generated with a 10mm expansion in the cranio-caudal direction and a 7mm expansion in other directions.

- *Dose prescription*: The radiation dose will be prescribed to the 90% isodose line in order to deliver 95% of the planned dose to 95% of the PTV. Tissue heterogeneity corrections should be always used. When needed, respiratory motion management was used.

Dosimetric requirement are available for each location ans dose constraints to organs at risk (OARs) have been published.

Table 15: Example of dose constraints limits to OARs:

(source Stereotactic Ablative Body Radiation Therapy (SABR UK Consortium) : A Resource Version 4.1, April 2014 Endorsed by The Faculty of Clinical Oncology of The Royal College of Radiologists)

Table 6-6 **OAR Dose Limits for 3 fraction SBRT**

Serial Organ	Volume	Volume Dose (Gy)	Avoidance Endpoint (Reference)
Spinal Cord	<0.03 cc	22.5	Myelitis (Timmerman)
	<1.2 cc	13	Myelitis (Timmerman)
Ipsilateral Brachial Plexus	< 0.03 cc	26	Brachial Plexopathy (Timmerman)
	<3 cc	22	Brachial Plexopathy (Timmerman)
Cauda Equina	<0.03 cc	25.5	Neuritis (Timmerman)
	<5 cc	21.9	Neuritis (AAPM TG-101)
Sacral Plexus	<0.03 cc	24	Neuropathy (AAPM TG-101)
	<5 cc	22.5	Neuropathy (AAPM TG-101)
Trachea and Ipsilateral Bronchus*	<0.03 cc	30	Stenosis/Fistula (Z4099)
	<5cc	25.8	Stenosis/Fistula (Timmerman)
Esophagus*	<0.03 cc	27	Stenosis/Fistula (Timmerman 2006 /RTOG 0618)
	<5cc	17.7	Stenosis/Fistula (Z4099)
Heart/Pericardium	<0.03cc	30	Pericarditis (Z4099)
	<15 cc	24	Pericarditis (Z4099)
Great vessels*	<0.03cc	45	Aneurysm (Z4099)
	<10 cc	39	Aneurysm (Z4099)
Skin	<0.03cc	33	Ulceration (Z4099)
	<10cc	31	Ulceration (Timmerman)
Stomach	<0.03cc	30	Ulceration/Fistula (Timmerman)
	<10cc	22.5	Ulceration/Fistula (Timmerman)

Duodenum*	<0.03cc	24	Ulceration (Timmerman 2006)
	<10cc	15	Ulceration (Timmerman 2006)
Bowel*	<0.03 cc	34.5	Ulceration (Timmerman)
	<20cc	24	Colitis/Fistula (Z4099)
Rectum*	<0.03 cc	49.5	Ulceration (Timmerman)
	<3.5 cc	45	Proctitis/Fistula (Timmerman)

	< 20 cc	27.5	Proctitis/Fistula (Timmerman)
Bladder	0.03cc	33	Cystitis/Fistula (Timmerman)
	<15 cc	16.8	Cystitis/Fistula (AAPM TG-101)
Ureter	<0.03 cc	40	Stenosis (Timmerman)
Penile bulb	< 3cc	25	Impotence (Timmerman)
Femoral heads	<10 cc	24	Necrosis (Timmerman)
Bile duct	< 0.03 cc	36	Stenosis (Timmerman)
Renal hilum/vascular trunk	<15 cc	19.5	Malignant Hypertension (Timmerman)
Rib	< 0.03 cc	50	Pain or Fracture (Timmerman
	<5 cc	40	Pain or Fracture (Timmerman

**Parallel Organ**	**Volume**	**Volume Dose (Gy)**	**Avoidance Endpoint (Reference)**
Lung (total)	<15% lung volume	20	Pneumonitis/Lung Function (RTOG 0618)
	< 37% lung volume	11	Pneumonitis (Timmerman)
	1500 cc	10.5	Basic Lung Function (Z4099)
	1000 cc	11.4	Pneumonitis (Z4099)
Ipsilateral kidney	<130 cc	12.3	Nephritis (Timmerman 2006)
Total Kidney	<200cc	15	Basic Renal Function (Timmerman)
Liver	<700 cc	17.1	Liver function (Timmerman 2006/Z4099)

1. Lung metastasis

SABR will be used for peripheral tumour only.

Tumour located within the proximal bronchial tree as defined in RTOG 0236 protocol should not be treated:

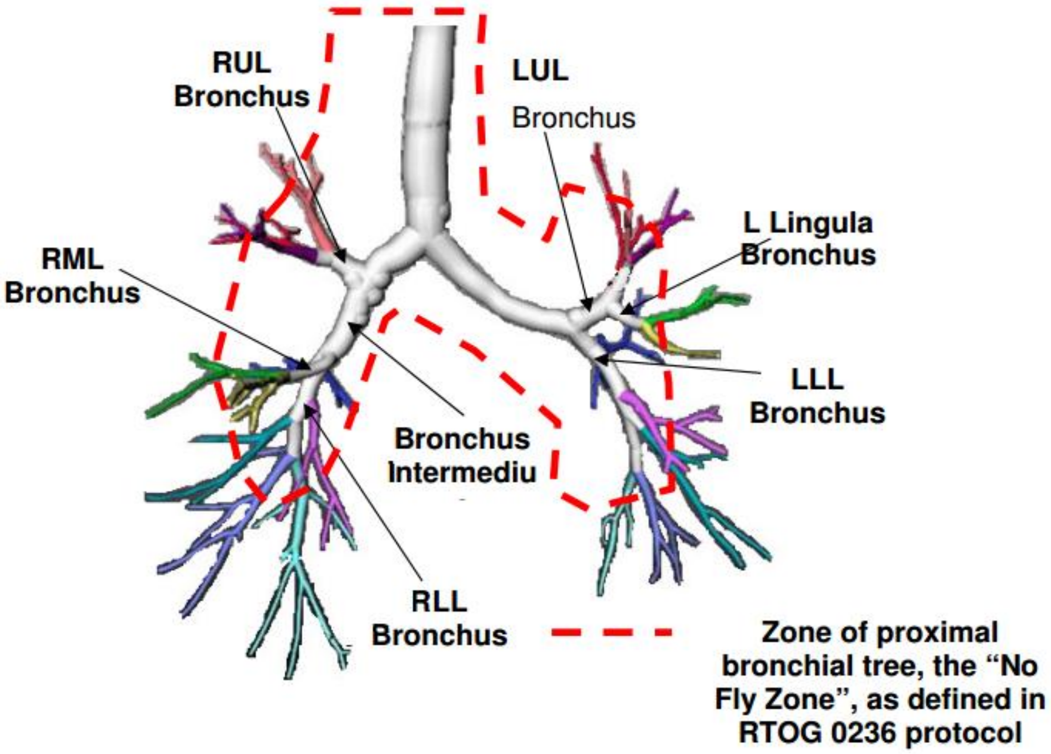

2. Liver metastasis

Specific inclusion criteria for liver metastasis

• Patients may be considered for SABR if they have radiographic liver lesions most consistent with metastases on contrast enhanced CT and/or MRI.

• The patient should not be amenable to elective liver surgery of the metastasis after review of the case by an experienced surgeon.

• Volume of uninvolved liver must be at least 700 cc

Specific exclusion criteria for liver <u>metastasis</u> : Metastasis adjacent to stomach or small bowel

Table 16: Child-Pguh liver score

**Measure**	**1 point**	**2 points**	**3 points**
Total Bilirubin (µmol/l) (mg/dl)	<34 (<2)	34-50 (2-3)	>50 (>3)
Serum albumin (g/l)	>35	28-35	<28
INR	<1.7	1.71-2.20	<2.20
Acites	None	Mild	Severe
Hepatic Encephalopathy	None	Grade 1-2 (or suppressed with medication)	Grade 3 or 4

**Points**	**Class**	**One Year Survival**	**Two Year Survival**
5-6	A	100%	85%
7-9	B	81%	57%
10-15	C	45%	35%

.

18 APPENDIX 2 – NATIONAL CANCER INSTITUTE - COMMON TERMINOLOGY CRITERIA FOR ADVERSE EVENTS

National Cancer Institute* - *Common Terminology Criteria for Adverse Events

(NCI-CTCAE, Version 4.0)

http://ctep.cancer.gov/protocolDevelopment/electronic_applications/ctc.htm#ctc_40

NATIONAL CANCER INSTITUTE

Cancer Therapy Evaluation Program

http://ctep.cancer.gov/

19 APPENDIX 3: LIST OF AUTOIMMUNE DISEASES:

- Acute Disseminated Encephalomyelitis (ADEM)
- Acute necrotizing hemorrhagic leukoencephalitis
- Addison's disease
- Agammaglobulinemia
- Alopecia areata
- Amyloidosis
- Ankylosing spondylitis
- Anti-GBM/Anti-TBM nephritis
- Antiphospholipid syndrome (APS)
- Autoimmune angioedema
- Autoimmune aplastic anemia
- Autoimmune dysautonomia
- Autoimmune hepatitis
- Autoimmune hyperlipidemia
- Autoimmune immunodeficiency
- Autoimmune inner ear disease (AIED)
- Autoimmune myocarditis
- Autoimmune oophoritis
- Autoimmune pancreatitis
- Autoimmune retinopathy
- Autoimmune thrombocytopenic purpura (ATP)
- Autoimmune thyroid disease
- Autoimmune urticaria
- Axonal & neuronal neuropathies
- Balo disease
- Behcet's disease
- Bullous pemphigoid
- Cardiomyopathy
- Castleman disease
- Celiac disease
- Chagas disease
- Chronic fatigue syndrome**
- Chronic inflammatory demyelinating polyneuropathy (CIDP)
- Chronic recurrent multifocal ostomyelitis (CRMO)
- Churg-Strauss syndrome
- Cicatricial pemphigoid/benign mucosal pemphigoid
- Crohn's disease
- Cogans syndrome
- Cold agglutinin disease
- Congenital heart block
- Coxsackie myocarditis
- CREST disease
- Essential mixed cryoglobulinemia
- Demyelinating neuropathies
- Dermatitis herpetiformis
- Dermatomyositis

- Devic’s disease (neuromyelitis optica)
- Discoid lupus
- Dressler’s syndrome
- Endometriosis
- Eosinophilic esophagitis
- Eosinophilic fasciitis
- Erythema nodosum
- Experimental allergic encephalomyelitis
- Evans syndrome
- Fibromyalgia**
- Fibrosing alveolitis
- Giant cell arteritis (temporal arteritis)
- Giant cell myocarditis
- Glomerulonephritis
- Goodpasture’s syndrome
- Granulomatosis with Polyangiitis (GPA) (formerly called Wegener’s Granulomatosis)
- Graves’ disease
- Guillain-Barre syndrome
- Hashimoto’s encephalitis
- Hashimoto’s thyroiditis
- Hemolytic anemia
- Henoch-Schonlein purpura
- Herpes gestationis
- Hypogammaglobulinemia
- Idiopathic thrombocytopenic purpura (ITP)
- IgA nephropathy
- IgG4-related sclerosing disease
- Immunoregulatory lipoproteins
- Inclusion body myositis
- Interstitial cystitis
- Juvenile arthritis
- Juvenile diabetes (Type 1 diabetes)
- Juvenile myositis
- Kawasaki syndrome
- Lambert-Eaton syndrome
- Leukocytoclastic vasculitis
- Lichen planus
- Lichen sclerosus
- Ligneous conjunctivitis
- Linear IgA disease (LAD)
- Lupus (SLE)
- Lyme disease, chronic
- Meniere’s disease
- Microscopic polyangiitis
- Mixed connective tissue disease (MCTD)
- Mooren’s ulcer
- Mucha-Habermann disease
- Multiple sclerosis
- Myasthenia gravis
- Myositis
- Narcolepsy
- Neuromyelitis optica (Devic’s)
- Neutropenia

- Ocular cicatricial pemphigoid
- Optic neuritis
- Palindromic rheumatism
- PANDAS (Pediatric Autoimmune Neuropsychiatric Disorders Associated with Streptococcus)
- Paraneoplastic cerebellar degeneration
- Paroxysmal nocturnal hemoglobinuria (PNH)
- Parry Romberg syndrome
- Parsonnage-Turner syndrome
- Pars planitis (peripheral uveitis)
- Pemphigus
- Peripheral neuropathy
- Perivenous encephalomyelitis
- Pernicious anemia
- POEMS syndrome
- Polyarteritis nodosa
- Type I, II, & III autoimmune polyglandular syndromes
- Polymyalgia rheumatica
- Polymyositis
- Postmyocardial infarction syndrome
- Postpericardiotomy syndrome
- Progesterone dermatitis
- Primary biliary cirrhosis
- Primary sclerosing cholangitis
- Psoriasis
- Psoriatic arthritis
- Idiopathic pulmonary fibrosis
- Pyoderma gangrenosum
- Pure red cell aplasia
- Raynauds phenomenon
- Reactive Arthritis
- Reflex sympathetic dystrophy
- Reiter’s syndrome
- Relapsing polychondritis
- Restless legs syndrome
- Retroperitoneal fibrosis
- Rheumatic fever
- Rheumatoid arthritis
- Sarcoidosis
- Schmidt syndrome
- Scleritis
- Scleroderma
- Sjogren’s syndrome
- Sperm & testicular autoimmunity
- Stiff person syndrome
- Subacute bacterial endocarditis (SBE)
- Susac’s syndrome
- Sympathetic ophthalmia
- Takayasu’s arteritis
- Temporal arteritis/Giant cell arteritis
- Thrombocytopenic purpura (TTP)
- Tolosa-Hunt syndrome
- Transverse myelitis
- Type 1 diabetes
- Ulcerative colitis

- Undifferentiated connective tissue disease (UCTD)
- Uveitis
- Vasculitis
- Vesiculobullous dermatosis
- Vitiligo
- Wegener’s granulomatosis (now termed Granulomatosis with Polyangiitis (GPA)

20 APPENDIX 4: RECIST 1.1 CRITERIA

The criteria below are based on RECIST 1.1. (Eisenhauer EA et al.Eur J Cancer 2009 ;45 :228-247)

Measurability of the disease

Measurable disease: Measurable disease requires the presence of at least one measurable lesion. If the measurable disease is restricted to a solitary lesion, its neoplastic nature should be confirmed by cytology/histology.

Measurable lesions:

- Lesions that can be accurately measured in at least one dimension with longest diameter ≥ 10 mm (by CT scan, MRI, caliper measurement) or ≥ 20 mm (by chest X-ray). Longest diameter will be recorded.
- For a lymph node to be considered pathologically enlarged and measurable, the short axis must be ≥15 mm (by CT scan). The short axis will be recorded.

Non-measurable disease:

- Non-measurable lesions are all other lesions, including small lesions (longest diameter <10 mm with CT scan, MRI or caliper measurement or <20 mm with chest X-ray or pathological lymph nodes with shortest axis ≥10 and <15 mm) as well as truly non-measurable lesions. Lesions considered truly non-measurable include leptomeningeal disease, ascites, pleural or pericardial effusion, inflammatory breast disease, lymphangitic involvement of skin or lung, abdominal masses/abdominal organomegaly identified by physical exam that is not measurable by reproducible imaging techniques.
- New lesions in irradiated fields
- Previously irradiated lesions should not be used as indicator lesions. However, new lesions occurring in previously irradiated fields can be used to assess the anti-tumour response.

Methods of measurement

All measurements must be recorded in metric notation, using a ruler or calipers. All baseline evaluations must be performed as close as possible to the treatment start and within 4 weeks before the beginning of the treatment. If a lesion is considered too small to measure, a default measurement of 5 mm should be applied. If the lesion is not visible, a default measurement of 0mm should be applied.

The same method of assessment and the same technique must be used to characterise each identified and reported lesion at baseline and during follow-up.

Clinical lesions will only be considered measurable when they are superficial (e.g., skin nodules, palpable lymph nodes). In the case of skin lesions, documentation by colour photography including a ruler to estimate the size of the lesion is suggested.

CT and MRI are the best currently available and reproducible methods to measure target lesions selected for response assessment. Conventional CT and MRI should be performed with cuts of 5 mm or less in slice thickness contiguously. Spiral CT should be performed using a 5 mm contiguous reconstruction algorithm. This applies to the chest, abdomen and pelvis.

Ultrasound, endoscopy and laparoscopy should not be used to measure tumour lesions or evaluate tumour response. However, these techniques can be useful to supplement information from other techniques.

Tumour markers alone cannot be used to assess response. If markers are initially above the upper normal limit, they must normalise for a patient to be considered in complete clinical response.

Cytology and histology can be used to differentiate between PR and CR in rare cases (for example, residual lesions in tumour types such as germ cell tumours, where known residual benign tumours can remain).

Baseline Documentation of Target and Non-target Lesions

All measurable lesions up to a maximum of two lesions per organ and five lesions in total, representative of all involved organs should be identified as target lesions and will be recorded, measured and numbered at baseline. The longest diameter will be recorded, except for lymph nodes, which will be measured by their short axis. Target lesions should be selected on the basis of their size (lesions with the longest diameter) and their suitability for accurate repetitive measurements (either by imaging techniques or clinically).

The sum of diameters for all target lesions will be calculated and reported as the baseline sum of diameters. The baseline sum of diameter will be used as reference to further characterize the objective tumour response of the measurable dimension of the disease (Table 1.1).

Table 1.1: Evaluation of target lesions

Complete Response (CR)	Disappearance of all target lesions. Any pathological lymph nodes (whether target or non-target) must have a reduction in short axis to <10mm)
Partial Response (PR)	At least a 30% decrease in the sum of diameters of target lesions, taking as reference the baseline sum diameters
Progression (PD)	At least a 20% increase in the sum of diameters of target lesions, taking as reference the smallest sum on study (this includes the baseline sum if that is the smallest on study). In addition to the relative increase of 20%, the sum must also demonstrate an absolute increase of at least 5 mm. (Note: The appearance of one or more new lesions is also considered progression.)
Stable Disease (SD)	Neither sufficient shrinkage to qualify for PR nor sufficient increase to qualify for PD, taking as reference the smallest sum diameters while on study.

All other lesions (or sites of disease) should be identified as non-target lesions and should also be recorded at baseline. Measurements are not required and these lesions should be followed as “present” or “absent” (Table 1.2).

Table 1.2. : Evaluation of non target lesions

Complete Response (CR)	Disappearance of all non-target lesions and normalisation of tumour marker level. All lymph nodes must be non-pathological in size (<10mm short axis)
Non-CR / Non-PD	Persistence of one or more non-target lesions and/or maintenance of tumour marker level above normal limits
Progression (PD)	Unequivocal progression (see comments below) of existing nontarget lesions. (Note: the appearance of one or more new lesions is also considered progression).<br><br>Although a clear progression of non-target lesions only is exceptional, in such circumstances, the opinion of the treating physician should prevail and the progression status should be confirmed later by the review panel (or study chair).

In some circumstances it may be difficult to distinguish residual disease from normal tissue.

When the evaluation of complete response depends upon this determination, it is recommended that the residual lesion be investigated (fine needle aspirate/biopsy) before confirming the complete response status.

Evaluation of Best Response to Study Treatment

The best response to study treatment (Table 1.3) is the best response recorded from the start of treatment until disease progression or start of further anti-cancer treatment (taking as reference for progressive disease the smallest measurements recorded since the treatment started). In general, the patient's best response assignment will depend on the achievement of both measurements and confirmation criteria.

Patients with a global deterioration of health status requiring discontinuation of treatment without objective evidence of disease progression at that time should be reported as "symptomatic deterioration". Every effort should be made to document the objective progression even after discontinuation of treatment.

Table 1.3. Evaluation of overall best response

**Target lesions**	**Non-target lesions**	**New lesions**	**Overall response**
CR	CR	No	CR
CR	Non-CR / Non-PD	No	PR
CR	Not evaluated	No	PR
PR	Non-PD or not all	No	PR

	evaluated		
SD	Non-PD or not all evaluated	No	SD
Not all evaluated	Non-PD	No	Not evaluated
PD	Any	Yes or No	PD
Any	PD	Yes or No	PD
Any	Any	Yes	PD

When SD is believed to be the best response, follow-up measurements must have met the SD criteria at least once after study entry at a minimum interval of six (6) weeks.

21 APPENDIX 5: mRECIST CRITERIA MODIFIED RESPONSE EVALUATION CRITERIA IN SOLID TUMORS

Conventional response criteria may not be adequate to characterize the anti-tumor activity of immunotherapeutic agents like Atezolizumab, which can produce delayed responses that may be preceded by initial apparent radiological progression, including the appearance of new lesions. Therefore, modified response criteria have been developed that account for the possible appearance of new lesions and allow radiological progression to be confirmed at a subsequent assessment. In this protocol, patients will be permitted to continue study treatment even after modified Response Evaluation Criteria in Solid Tumors (RECIST) criteria for progressive disease are met if the risk/benefit ratio is judged to be favorable.

Modified RECIST is derived from RECIST, Version 1.1 conventions [1] and immune-related response criteria [2] (irRC). When not otherwise specified, RECIST v1.1 conventions will apply.

Modified RECIST and RECIST, Version 1.1: Summary of Changes

	RECIST v1.1	Modified RECIST
New lesions after baseline	Define progression.	New measurable lesions are added into the total tumor burden and followed.
Non-target lesions	May contribute to the designation of overall progression	Contribute only in the assessment of a complete response
Radiographic progression	First instance of □ 20% increase in the sum of diameters or unequivocal progression in non-target disease	Determined only on the basis of measurable disease; must be confirmed by a consecutive assessment □ 4 weeks from the date first documented

<u>DEFINITIONS OF MEASURABLE/NON-MEASURABLE LESIONS</u>

All measurable and non-measurable lesions should be assessed at screening and at the protocol-specified tumor assessment timepoints. Additional assessments may be performed, as clinically indicated for suspicion of progression. The investigator will evaluate response to treatment using modified RECIST.

---

1 Eisenhauer EA, Therasse P, Bogaerts J, et al. New response evaluation criteria in solid tumours: revised RECIST guideline (version 1.1). Eur J Cancer 2009;45:228–47.

2 Topalian SL, Hodi FS, Brahmer JR, et al. Safety, activity, and immune correlates of anti–PD-1 antibody in cancer. N Engl J Med 2012;366:2443–54.

Wolchok JD, Hoos A, O'Day S, et al. Guidelines for the evaluation of immune therapy activity in solid tumors: immune-related response criteria. Clin Can Res 2009;15:7412–20.

MEASURABLE LESIONS

Tumor Lesions. Tumor lesions must be accurately measured in at least one dimension (longest diameter in the plane of measurement is to be recorded) with a minimum size as follows:

1. 10 mm by computed tomography (CT) or magnetic resonance imaging (MRI) scan (CT/MRI scan slice thickness/interval no greater than 5 mm)

2. 10-mm caliper measurement by clinical examination (lesions that cannot be accurately measured with calipers should be recorded as non-measurable)

Malignant Lymph Nodes. To be considered pathologically enlarged and measurable, a lymph node must be ≥ 15 mm in the short axis when assessed by CT scan (CT scan slice thickness recommended to be no greater than 5 mm). At baseline and follow-up, only the short axis will be measured and followed.

NON-MEASURABLE LESIONS

Non-measurable tumor lesions encompass small lesions (longest diameter < 10 mm or pathological lymph nodes with short axis ≥ 10 but < 15 mm), as well as truly non-measurable lesions. Lesions considered truly non-measurable include leptomeningeal disease, ascites, pleural or pericardial effusion, inflammatory breast disease, lymphangitic involvement of skin or lung, peritoneal spread, and abdominal mass/abdominal organomegaly identified by physical examination that is not measurable by reproducible imaging techniques.

SPECIAL CONSIDERATIONS REGARDING LESION MEASURABILITY

Bone lesions, cystic lesions, and lesions previously treated with local therapy require particular comment, as outlined below.

BONE LESIONS

Bone scan, positron emission tomography (PET) scan, or plain films are not considered adequate imaging techniques for measuring bone lesions. However, these techniques can be used to confirm the presence or disappearance of bone lesions.Lytic bone lesions or mixed lytic–blastic lesions, with identifiable soft tissue components, that can be evaluated by cross-sectional imaging techniques such as CT or MRI can be considered as measurable lesions if the soft tissue component meets the definition of measurability described above. Blastic bone lesions are non-measurable.

CYSTIC LESIONS

Lesions that meet the criteria for radiographically defined simple cysts should not be considered as malignant lesions (neither measurable nor non-measurable) since they are, by definition, simple cysts. Cystic lesions thought to represent cystic metastases can be considered as measurable lesions, if they meet the definition of measurability described above. However, if non-cystic lesions are present in the same patient, these are preferred for selection as target lesions.

LESIONS WITH PRIOR LOCAL TREATMENT

Tumor lesions situated in a previously irradiated area or in an area subjected to other loco-regional therapy are usually not considered measurable unless there has been demonstrated

progression in the lesion. Study protocols should detail the conditions under which such lesions would be considered measurable.

<u>TUMOR RESPONSE EVALUATION</u>

DEFINITIONS OF TARGET/NON-TARGET LESIONS

<u>Target Lesions</u>

When more than one measurable lesion is present at baseline, all lesions up to a maximum of five lesions total (and a maximum of two lesions per organ) representative of all involved organs should be identified as target lesions and will be recorded and measured at baseline. This means that, for instances in which patients have only one or two organ sites involved, a maximum of two lesions (one site) and four lesions (two sites), respectively, will be recorded. Other lesions (albeit measurable) in those organs will be recorded as non-measurable lesions (even if the size is > 10 mm by CT scan).

Target lesions should be selected on the basis of their size (lesions with the longest diameter) and be representative of all involved organs, but in addition, should lend themselves to reproducible repeated measurements. It may be the case that, on occasion, the largest lesion does not lend itself to reproducible measurement, in which circumstance, the next largest lesion that can be measured reproducibly should be selected.

Lymph nodes merit special mention since they are normal anatomical structures that may be visible by imaging even if not involved by tumor. As noted above, pathological nodes that are defined as measurable and may be identified as target lesions must meet the criterion of a short axis of ≥ 15 mm by CT scan. Only the short axis of these nodes will contribute to the baseline sum. The short axis of the node is the diameter normally used by radiologists to judge if a node is involved by solid tumor. Nodal size is normally reported as two dimensions in the plane in which the image is obtained (for CT, this is almost always the axial plane; for MRI, the plane of acquisition may be axial, sagittal, or coronal). The smaller of these measures is the short axis. For example, an abdominal node that is reported as being 20 mm x 30 mm has a short axis of 20 mm and qualifies as a malignant, measurable node. In this example, 20 mm should be recorded as the node measurement. All other pathological nodes (those with short axis ≥ 10 mm but < 15 mm) should be considered non-target lesions. Nodes that have a short axis of < 10 mm are considered non-pathological and should not be recorded or followed. Lesions irradiated within 3 weeks prior to Cycle 1, Day 1 may not be counted as target lesions.

<u>Non-Target Lesions</u>

All other lesions (or sites of disease), including pathological lymph nodes, should be identified as non-target lesions and should also be recorded at baseline. Measurements are not required.

It is possible to record multiple non-target lesions involving the same organ as a single item on the Case Report Form (CRF) (e.g., “multiple enlarged pelvic lymph nodes” or “multiple liver metastases”).

After baseline, changes in non-target lesions will contribute only in the assessment of complete response (i.e., a complete response is attained only with the complete disappearance of all tumor lesions, including non-target lesions) and will not be used to assess progressive disease.

New Lesions

During the study, all new lesions identified and recorded after baseline must be assessed at all tumor assessment timepoints. New lesions will also be evaluated for measurability with use of the same criteria applied to prospective target lesions at baseline per RECIST, (e.g., non target lymph node lesions must be >10mm; see note for new lymph node lesions below). Up to a maximum of five new lesions total (and a maximum of two lesions per organ), all with measurements at all timepoints, can be included in the tumor response evaluation. New lesion types that would not qualify as target lesions per RECIST cannot be included in the tumor response evaluation.

New lesions that are not measurable at first appearance but meet measurability criteria at a subsequent timepoint will be measured from that point on and contribute to the sum of longest diameters (SLD), if the maximum number of 5 measurable new lesions being followed has not been reached.

CALCULATION OF SUM OF THE DIAMETERS

A sum of the diameters (longest for non-nodal lesions, short axis for nodal lesions) for all target lesions will be calculated as a measure of tumor burden.

The sum of the diameters is calculated at baseline and at each tumor assessment for the purpose of classification of tumor responses.

Sum of the Diameters at Baseline: The sum of the diameters for all target lesions identified at baseline prior to treatment on Day 1.

Sum of the Diameters at Tumor Assessment: For every on-study tumor assessment collected per protocol or as clinically indicated, the sum of the diameters at tumor assessment will be calculated using tumor imaging scans. All target lesions and all new measurable lesions that have emerged after baseline will contribute to the sum of the diameters at tumor assessment. Hence, each net percentage change in tumor burden per assessment with use of modified RECIST accounts for the size and growth kinetics of both old and new lesions as they appear.

Note: In the case of new lymph nodes, RECIST v1.1 criteria for measurability (equivalent to baseline target lesion selection) will be followed. That is, if at first appearance the short axis of a new lymph node lesion ≥ 15 mm, it will be considered a measureable new lesion and will be tracked and included in the SLD. Thereafter, the lymph node lesion will be measured at subsequent timepoints and measurements will be included in the SLD, even if the short axis diameter decreases to < 15 mm (or even < 10 mm). However, if it subsequently decreases to < 10 mm, and all other lesions are no longer detectable (or have also decreased to a short axis diameter of < 10 mm if lymph nodes), then a response assessment of CR may be assigned.

If at first appearance the short axis of a new lymph node is ≥ 10 mm and < 15 mm, the lymph node will not be considered measurable but will still be considered a new lesion. It will not be included in the SLD unless it subsequently becomes measurable (short axis diameter ≥ 15 mm).

The appearance of new lymph nodes with diameter < 10 mm should not be considered pathological and not considered a new lesion.

RESPONSE CRITERIA

Evaluation of Target Lesions

Complete Response (CR): Disappearance of all target lesions. Lymph nodes that shrink to < 10 mm short axis are considered normal.

Partial Response (PR): At least a 30% decrease in the sum of the diameters of all target and all new measurable lesions, taking as reference the baseline sum of diameters, in the absence of CR.

Note: The appearance of new measurable lesions is factored into the overall tumor burden but does not automatically qualify as progressive disease until the sum of the diameters increases by ≥ 20% when compared with the sum of the diameters at nadir.

Stable Disease (SD): Neither sufficient shrinkage to qualify for PR nor sufficient increase to qualify for PD, taking as reference the smallest sum of the diameters while on study.

Progressive Disease (PD): At least a 20% increase in the sum of diameters of all target and all new measurable lesions, taking as reference the smallest sum on study (nadir SlD; this includes the baseline sum if that is the smallest on study). In addition to the relative increase of 20%, the sum must also demonstrate an absolute increase of at least 5 mm.

Impact of New Lesions on Modified RECIST

New lesions alone do not qualify as progressive disease. However, their contribution to total tumor burden is included in the sum of the diameters, which is used to determine the overall modified RECIST tumor response.

EVALUATION OF BEST OVERALL RESPONSE USING MODIFIED RECIST

TIMEPOINT RESPONSE

It is assumed that at each protocol-specified timepoint, a response assessment occurs. Table 1 provides a summary of the overall response status calculation at each timepoint for patients who have measurable disease at baseline.

MISSING ASSESSMENTS AND NOT EVALUABLE DESIGNATION

When no imaging/measurement is done at all at a particular timepoint, the patient is not evaluable (NE) at that timepoint. If only a subset of lesion measurements are made at an assessment, usually the case is also considered NE at that timepoint, unless a convincing argument can be made that the contribution of the individual missing lesion(s) would not change the assigned time point response. This would be most likely to happen in the case of PD. For example, if a patient had a baseline sum of 50 mm with three measured lesions and at follow-up only two lesions were assessed but those gave a sum of 80 mm, the patient will have achieved PD status, regardless of the contribution of the missing lesion.
